# High evolutionary turnover of satellite families in *Caenorhabditis*

**DOI:** 10.1186/s12862-015-0495-x

**Published:** 2015-10-05

**Authors:** Juan A. Subirana, M. Mar Albà, Xavier Messeguer

**Affiliations:** Department of Computer Science, Universitat Politècnica de Catalunya, Jordi Girona 31, Barcelona, 08034 Spain; Evolutionary Genomics Group, Research Programme on Biomedical Informatics (GRIB) – Hospital del Mar Research Institute (IMIM), Universitat Pompeu Fabra (UPF), Dr. Aiguader 86, Barcelona, 08003 Spain

**Keywords:** Nematodes, Satellite DNA, Tandem repeat sequences, *Caenorhabditis elegans*, Mitosis, DNA elimination

## Abstract

**Background:**

The high density of tandem repeat sequences (satellites) in nematode genomes and the availability of genome sequences from several species in the group offer a unique opportunity to better understand the evolutionary dynamics and the functional role of these sequences. We take advantage of the previously developed SATFIND program to study the satellites in four *Caenorhabditis* species and investigate these questions.

**Methods:**

The identification and comparison of satellites is carried out in three steps. First we find all the satellites present in each species with the SATFIND program. Each satellite is defined by its length, number of repeats, and repeat sequence. Only satellites with at least ten repeats are considered. In the second step we build satellite families with a newly developed alignment program. Satellite families are defined by a consensus sequence and the number of satellites in the family. Finally we compare the consensus sequence of satellite families in different species.

**Results:**

We give a catalog of individual satellites in each species. We have also identified satellite families with a related sequence and compare them in different species. We analyze the turnover of satellites: they increased in size through duplications of fragments of 100-300 bases. It appears that in many cases they have undergone an explosive expansion. In *C. elegans* we have identified a subset of large satellites that have strong affinity for the centromere protein CENP-A. We have also compared our results with those obtained from other species, including one nematode and three mammals.

**Conclusions:**

Most satellite families found in *Caenorhabditis* are species-specific; in particular those with long repeats. A subset of these satellites may facilitate the formation of kinetochores in mitosis. Other satellite families in *C. elegans* are either related to *Helitron* transposons or to meiotic pairing centers.

**Electronic supplementary material:**

The online version of this article (doi:10.1186/s12862-015-0495-x) contains supplementary material, which is available to authorized users.

## Background

Satellites are tandem repeat sequences present in many eukaryotic genomes. The evolution and biological roles of satellites in different species has recently attracted much attention [1, 2]. Previously we reported that the genome of the worm *Caenorhabditis elegans* has a large amount of satellites, which represent about 3 % of the genome [3]. Satellites were originally identified by density gradient centrifugation [4]. Recent definitions based on genomic sequences vary across different studies [1, 2]. Here we define satellites as long tandem repeats with at least ten repeats; with each repeat having a length of 10–200 nucleotides. Satellites with fewer repeats are not included, in order to simplify the analysis and get a clear view of the major satellite families. No limit is placed on the total length of individual satellites.

The large number of satellites in nematodes offers the opportunity to study several intriguing features of satellites in more detail, such as their expansion, transposition and elimination from the genome. These questions are strongly related to other features of genome evolution, such as gene duplication and intron turnover, which are very frequent in *C. elegans* [5–8]. For example, multigene families are subjected to birth and death evolution, with a significant component of neutral change [9, 10].

The satellites of *C. elegans* show a characteristic distribution of repeat sizes, which suggests that different groups or families may play unique roles in the genome. The number and size distribution of satellites is very different from that observed in mammals, which have a much lower number of satellites with long repeats. In this paper we analyze in detail the different families of satellites present in *C. elegans*. We compare them with those present in other related *Caenorhabditis* species. We study in greater detail those satellites which have centromere-like features. As an outside group we study a distant nematode species: *Meloidogyne hapla*. Several features of satellites in different *Meloidogyne* species have been previously studied [11, 12], but no attempt has been made to analyze the different families of satellites in either *Meloidogyne* or in any other nematode species. We have also studied the microsatellite distribution in the *Caenorhabditis* species, which complement previous detailed studies on microsatellites in diverse species [13, 14]. Some microsatellites have been reported to play a role in gene expression regulation [15]; their variability and complex evolution have been reviewed by Ellegren [16].

Our results contribute to the annotation and interpretation of poorly characterized non-coding regions of the genome [17]. We discuss our results in light of the different hypotheses that have been proposed to explain the expansion and elimination of satellites.

## Methods

### Genome sequences

We used the genome sequences available in Wormbase [18]: versions WS201 for *C. elegans* and WS247 for *Caenorhabditis briggsa*e, *Caenorhabditis brenneri*, *Caenorhabditis remanei* and *M. hapla*. The WS247 version of the genome of *C. briggsae* is of higher quality than the version we used in a previous work [3]; whereas the *M. hapla* version is identical. The position of satellites in *C. elegans* is practically the same in the WS201 version of the genome than in the previously employed [3] WS190/ce6, except in chromosome V, where displacements of up to 3 Kb may be found. The use of WS201 was determined by the fact that the CENP-A data we used were obtained from this genome version [19].

### Satellite identification

Repetitive sequences were identified with the program SATFIND, which was developed in order to determine the position of long tandem repeats (satellites). SATFIND is available on-line for general use on our website [20]. The underlying algorithm is described in a previous publication [3]. Its source code has been deposited in Dryad [21]. The program determines the localization of clusters of any short sequence of a prefixed size without internal repetitions and repeated a minimum number of times in regions with a fixed size. In this paper we have used the SATFIND program to identify satellites formed by at least ten repeats of any decamer sequence in 2 Kb long regions. Once a satellite is located, the program continues its search along the genome until no further neighboring repeats are detected. In this way repeats of 10–200 nucleotides repeated at least 10 times can be positioned in the genome, with no upper limit for the number of repeats in the satellite. We have analyzed the statistical significance of satellites by computing the expected number of times a pattern of length L will appear by chance n times in a DNA sequence of length N: RE ~ N · (4^-L^)^n^. For N = 2000, L = 10 and n = 9 it turns out that RE ~ 10^**−**45^; this means that the satellites found using these parameters are far from random.

Most satellites have a regular structure, but there is a significant number which present variations in repeat length and composition. In order to eliminate the most irregular satellites, we have only accepted those which have at least 30 % of their repeats with an identical length. We have analyzed the statistical significance of this choice; given a sequence of length L that is randomly partitioned into n subsequences, the expected number of times that k subsequences will have the same length can be approximated by RE ~ (^n^_k_) · L^-·(k-1).^ For n = 10 and L = 2000, the probability to find three sequences (30 %) with the same length is <10^−3^.

We have further limited our study to satellites with repeats shorter than 200 bases, since there are very few satellites with longer repeats in *Caenorhabditis.* Some repeat sequences were compared with the satellites embedded in the *C. elegans Helitron* sequences obtained from Repbase [22]. *Helitrons* are a special class of DNA transposons, which are associated with different satellite families in *C. elegans*, as we will show below.

### Satellite sequence comparisons

To compare satellites we have used Malig, a progressive multiple sequence alignment algorithm, which we have developed to align satellite repeats and identify families with a related sequence. Its source code has been deposited in Dryad [23]. As a progressive method, Malig first computes the similarity score between all pairs of sequences using a dynamic programming algorithm [24]. The program considers reverse sequences as well, normalizes the alignment score to the maximum possible value and selects the cycle permutation with the highest score. Then the progressive multi-alignment is applied to the matrix of pairwise alignment scores. The process finishes when the score is smaller than a similarity threshold (input parameter) which we set to 0.6.

To calculate the statistical significance of the initial pairwise alignments, we have generated a set of random sequences with the same length distribution than satellites and equivalent GC content (30 % A and T; 20 % G and C) and performed pairwise alignments as described previously. We have used the random sequence pairwise score distribution to set up a score threshold, for which the observed probability in random alignments (p-value) is <10^−4^. As the p-value depends on the length of the alignment, we have used the linear regression (−0.2837’ · length of alignment + 66.0155)/100 to adjust it to different satellite lengths. We have applied this method to the detection of satellites with a common origin in different species.

Each family within a species is characterized by the abbreviated name of the species and three values, eg Cele_Fam_*a_b_c*. The order in the list of families is given by “*a”,* starting with those families with the largest number of members. The second value “*b”* gives the size of the repeat; “*c”* gives the number of members in the family. For simplicity we have omitted *c* throughout the text. Unique satellites appear at the end of the list, as families with a single member. We also define families as “large families” if they have ten or more members. The consensus sequence of the repeat is calculated taking into account the circularly permuted sequence of all repeats. To compare satellites from different species we have used the consensus sequence from each satellite family.

### Quantification of CENP-A affinity

In order to determine the sequence affinity for CENP-A, we used the file 2446-OD00079_HCP3_N2_MXEMB_1.wig, available from modENCODE [25]. Several centromere specification arrays, obtained with different rabbit polyclonal antibodies [19] are available at Wormbase [18]; all of them give a similar distribution of CENP-A affinity. We transformed the logarithm values in the wig file to their natural number value, in order to calculate average affinity values for the regions covered by the satellites. All average affinity values in our work are given as natural numbers. We have arbitrarily divided the satellites in two equal groups, with either a high or low CENP-A affinity. We have defined the limit between the two groups by the value of the median, which is 0.922.

## Results

### General satellite features in *C. elegans*

**Fig. 1:**
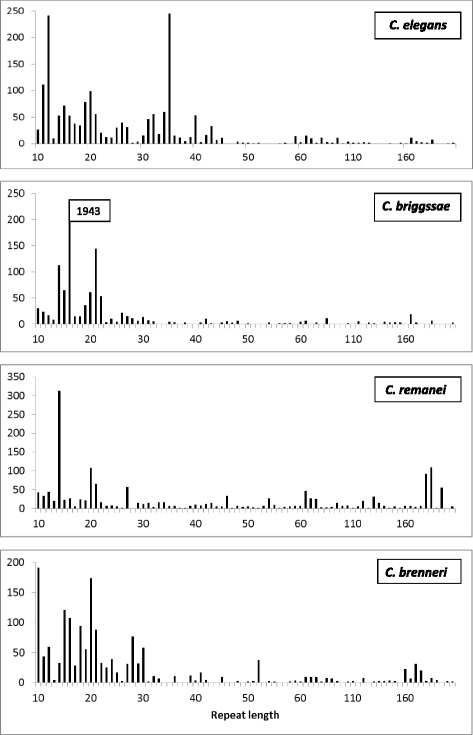
Repeat size distribution of satellites in the genomes of *Caenorhabditis*. The number of satellites found is represented as a function of their repeat size. For repeats longer than 60 bases the data have been merged in bins of 5 bases. The sixteen bases repeat in *C. briggsae* is very abundant; the total number of these satellites is indicated

**Fig. 2:**
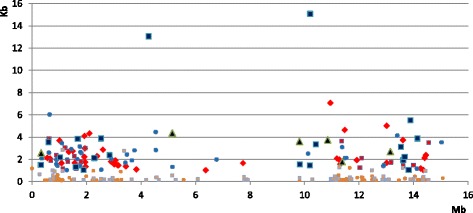
Detailed distribution of satellites in chromosome I of *C. elegans*. The ordinate corresponds to the size of individual satellites. Larger symbols indicate longer repeats: Black triangles, 127–224 bases; dark blue squares, 56–102; red squares, 37–46: red diamonds, 32–36; blue dots, 19–31; orange squares 13–18; orange dots, 11–12. Only a few short satellites are found in the central 4 Mb of the chromosome

We identified 1,779 satellites, using a threshold of a minimum of ten repeats, each with 10–200 bases (Additional file 1). Over half of the satellites are shorter than 1 Kb and only 21 % are longer than 2Kb. A list of the longest satellites is given in Table 1. Satellites may be classified as a function of the size of its repeats, (Fig. 1 and Table 2); the distribution of repeat sizes is clearly non-random. In *C. elegans* there is a negligible amount of satellites with repeat sizes between 50–58 and 110–160 bases. Such distribution suggests that satellites with different repeat sizes might have different origins and/or functions. The distribution of repeat sizes is similar in all chromosomes (Additional file 2: Figure S1); with the exception of the X chromosome, which has fewer satellites. The satellites do not have a uniform distribution throughout chromosomes, they are less frequent in the center (Fig. 2 and Additional file 2: Figure S2). This fact was previously reported when the whole genome sequence was published [26]. Unlike in other species, recombination in nematodes mainly occurs in the gene-poor arms of the chromosomes, which have a higher density of satellites and other types of noncoding DNA [27].

**Table 1:** Properties of satellites longer than 7 Kb in *C. elegans.* Note that most of these satellites have a CENP-A score above the median value (0.922). All these satellites are species-specific; no satellites with a related sequence are found in the other *Caenorhabditis* species. A notable exception is the satellite with repeat 102 found in chromosome IV, which has a single related satellite in all other *Caenorhabditis* species

Chromosome	Position (Start)	Satellite length	Repeat length	Number of repeats	CENP-A score
I	4281488	13061	94	139	1.767
I	10204118	15081	68	223	1.262
I	10945931	7072	32	238	1.326
II	3159311	9176	162	55	1.070
II	9807896	17344	59	292	1.503
II	14322264	9454	185	43	1.339
III	1362848	7149	162	44	1.182
III	7405325	37816	94	414	1.757
III	11428971	7697	43	176	1.294
III	11593322	8828	184	48	1.705
III	11639888	7523	163	35	1.192
III	13074654	11986	59	206	1.297
III	13584176	15068	34	470	2.363
IV	688812	9391	102	92	1.285
IV	6668156	8287	182	46	1.113
IV	6682649	21927	172	125	1.114
IV	6708756	22979	174	130	1.118
IV	8572629	9232	20	402	0.650
V	6176225	6989	117	54	2.042
V	7916946	8052	40	201	0.636
V	13640213	7956	16	356	0.568
V	14584093	6934	162	32	0.999
V	17384209	10276	59	173	0.928
X	4031449	25036	20	1238	0.603
X	7351421	9929	40	248	0.638
X	10361426	7683	25	299	0.612
X	16931765	8401	151	50	1.150

**Table 2:** Distribution of satellites in different species

						Nr of satellites with different repeat sizes/Mb					Nr. satellites Repeat 84
Species	Size Genome (Mb)	CG %	Number of satellites	Satellites/Mb	Sats/Mb >2 Kb	11-20	21-49	50-99	100-150	>150	
*C.elegans*	100.3	35.4	1779	17.7	3.8	8.18	8.18	0.81	0.14	0.35	1
*C.briggsae*	108.4	37.5	2778	25.6	5.4	21.5	3.23	0.40	0.26	0.34	0
*C.remanei*	145.4	38.0	1643	11.3	4.2	4.55	2.48	1.40	0.65	1.97	0
*C.brenneri*	190.4	38.6	1608	8.44	2.2	4.80	2.58	0.56	0.15	0.54	4
*M.hapla*	53.0	27.4	671	12.7	2.0	4.13	5.04	2.55	0.15	0.79	4
*H.sapiens* ^a^	2994.6	40.9	12036	4.0	0.30	1.89	1.74	0.38	0.01	0^a^	141
*M.musculus*	2790.9	41.9	28746	10.3	0.30	8.47	1.57	0.23	0.01	0.02	271
*R.norvegicus*	2902.6	42.0	26799	9.2	0.29	7.23	1.80	0.18	0.01	0.02	114

^a^Alpha satellites have been excluded, as explained in the text

We observe that, as a function of repeat size, no clear pattern of distribution of satellites along the chromosomes is apparent. This is represented well in chromosome I, where repeats of different sizes may occupy any region in the chromosome (Fig. 2). Mutational dynamics of satellites may include frequent insertions and deletions. We have compared the genomic positions of the satellites in *C. elegans* to available copy number variation from twelve strains [28], but found no significant enrichment or depletion, as shown in Additional file 3: Table S1.

### Short repeat satellite families in *C. elegans*

The satellites can be grouped into families, which share a related repeat sequence. In *C. elegans* we have detected a total of 364 families, each with a different number of members (Table 3). Most families are present in all chromosomes, whereas a few of them appear only in one or two chromosomes (Additional file 3: Table S2). In this section we describe the major 25 families of satellites with short repeats (less than 50 bases), which represent 64.2 % of all satellites.

**Table 3:** Satellite families

Species	Nr. satellites	Number of families				
		Total	>9	3-9	2	1
*C.elegans*	1779	364	26	64	57	217
*C.briggsae*	2788	275	24	51	40	160
*C.brenneri*	1643	403	31	82	77	213
*C.remanei*	1608	347	29	71	51	196
*M.hapla*	671	304	7	52	68	177

Satellite families are classified by the number of members in each family. Satellites which have not been aligned with any other satellite in the same species are considered as families with one member

Cele_Fam_1_35 is the largest family of satellites (213 members). It is practically absent in the X chromosome, where only one satellite of this family was found. In the autosomes it is only found in the terminal regions; it is completely absent in a central region of about 7–10 Mb. Its consensus repeat has an internal repeated motif of eight bases and two palindromic regions of thirteen bases. This satellite is only present in *C. elegans*, no related satellites have been detected in the other species we have studied. A combination of this repeat with the related Cele_Fam_9_43 has been previously described as MINISAT1 [22].

Cele_Fam_2_12 corresponds to telomeric sequences, which in this species forms 203 short satellites distributed throughout the genome, as previously described [26]. Interspersed telomere sequences have also been found in other species, such as some fish [29]. Chromosome X is an exception, since telomere sequences only appear at its canonical position: the ends of the chromosome.

Another large group of satellites are associated with *Helitron* transposons, which have been described in many species [30]. This group has 345 members distributed in six families: Cele_Fam_17_20, 3_34 and 20_37 have related sequences, whereas Cele_Fam_4_11, 5_15 and 7_40 are unrelated. Two of these families (Cele_Fam_5_15 and 3_34) had already been described in the pioneering work of Naclerio et al. [31]. Usually *Helitrons* only contain satellites of one or two families, but a larger number may be present. For example the *Helitron Y4_CE* located in chromosome I (starting at position 7868158) contains satellites of Cele_Fam_4_11, 5_15, 3_34 and 7_40. Satellites related to Cele_Fam_7_40 are also found in the other *Caenorhabditis*, as we will describe below.

Another two families, Cele_Fam_26_31 and Cele_Fam_13_32, are associated with terminal repeat sequences, related to the meiotic pairing centers described by various authors [32–34].

Most of the other families in this group are neither related among themselves nor with the other families. No apparent general features can be ascribed to any of them. They have between nine and forty eight members.

Analysis of the sequence of different satellite families revealed that they usually increase in size through the duplication of fragments of about 100–300 bases. We have focused on one family (Cele_Fam_24_19), to illustrate the mechanisms of expansion of satellites (Additional file 3: Tables S4 and S5) In this family all individual satellites present a mixture of repeats (ATTT)_n_AATTAAT, with either three or four ATTT units. This repeat sequence is completely absent in all other members of *Caenorhabditis*. From the distribution of point mutations and repeat sizes, it appears that the ancestral satellite was rather short and contained very few repeats. In a second step it was copied to other locations in the genome. Later the satellites increased in size through the duplication of fragments (Additional file 3: Table S5).

### Centromere-like satellites

The centromeres found in most monocentric species are associated with satellites with relatively long repeats, typically around 170 base pairs [35–38]. In the case of *C. elegans* there are 47 satellites with a similar repeat size, in the range of 151–226 base pairs (Additional file 3: Table S3). Most satellites in this group either have unique sequences or form families with a small number of members; the largest is Cele_Fam_34_163 with seven members.

The centromere is enriched in several proteins, a key one being CENP-A. We used the recently generated mapping of CENP-A on the *C. elegans* genome [18] to determine the affinity of different satellite sequences for this protein. The CENP-A domains were found to be distributed evenly in regions of the genome with low affinity and a very variable size (median 10–12 Kb). About five thousand CENP-A domains were identified, as deduced from the average domain size and genome coverage. Thus the genome may be considered as a mosaic, with about five thousand regions of 10–12 Kb which have affinity for CENP-A and are separated by regions of a similar size with low affinity for CENP-A. In agreement with the results of Gassmann *et al*. [18], we find that satellites are randomly distributed in domains of either low or high affinity for CENP-A. However all the longest satellites (>7 Kb) with a long repeat (>40 bases) have a high affinity for CENP-A (Table 1). Additionally, over 90 % of the long satellites (>2Kb) with repeats longer than 160 bases (Additional file 3: Table S3) are in regions of high CENP-A affinity (p-value = 4.376 · 10^−9^, Fisher’s Exact Test).

We have also compared the position of all the satellites we have detected with the 708 positions with CENP-A affinity reported by Steiner and Henikoff [39]. We found that only two of their positions are found within a satellite. Thus there is no correlation between both results.

A unique feature of the centromere-like satellites is their rather uniform distribution throughout chromosomes (Additional file 3: Table S3), whereas most other repeated regions tend to be located at the ends of chromosomes (Additional file 2: Figure S2). We have also found that all chromosomes have satellites with high CENP-A affinity and repeat size > 150.

### The satellites in *Caenorhabditis*

**Fig. 3:**
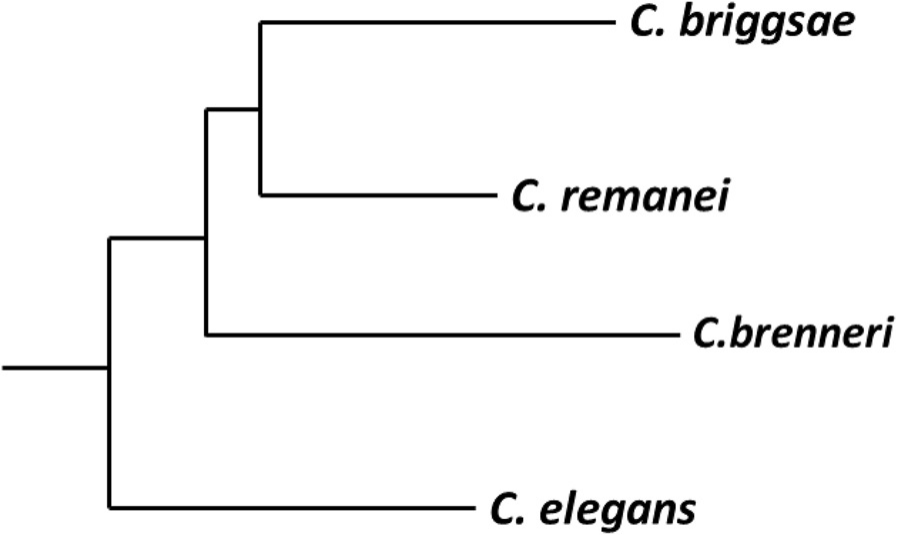
Phylogenetic relationship of the indicated species. Branch lengths were derived from RNA polymerase genes [70]

Next we compared the satellites of *C. elegans* with those present in other *Caenorhabditis*: *C. briggsae* (Cbrig), *C. remanei* (Crema) and *C. brenneri* (Cbren). The evolutionary relationship of these species is given in Fig. 3. The genome data presently available allow a clear view of the main features of satellites, although the genomes have not yet been fully assembled. The distribution of repeat sizes (Fig. 1) shows that all species are rich in satellites. The number of satellites identified is largest in *C. briggsae* and lowest in *M. hapla*, although satellite density is always greater than ten satellites per Mb (Table 2). Satellites with short repeats (11–20 bases) predominate; *C. elegans* stands out with the largest number of satellites with repeats of 30–40 bases, which are not frequent in the other species. The size distribution of the satellites in *C. elegans* is significantly different to the size distribution in the other species (Wilcoxon-Mann–Whitney test p-value < 10^−5^).

We generated satellite families in the different *Caenorhabditis* species. We frequently detected the presence of AAAA (or TTTT) tracts and common palindromes in the consensus sequence of many of these families. A summary of the size distribution of satellite families is given in Table 3. The main features of each species can be summarized as follows:

In *C. briggsae* we have localized 2,788 satellites, 2,352 of which can be grouped in 24 large families with more than 9 members each. This species stands out by having a large number of satellites (1,943) with a consensus repeat of 16 bases, most of which are octamer repeats. The two largest families have a similar consensus repeat AAWYTCAG. There are only two large families with a consensus repeat longer than 30 bases. There are 41 satellites with repeats longer than 150 bases. Surprisingly we only detected two short telomere repeats; apparently these repeats were not positioned in the available genome sequence.

The satellites of *C. brenneri* have a similar size distribution to those of *C. briggsae* (Fig. 1), although the total number of satellites is lower (1,643). This is mainly due to the absence of any large family of octamer repeats. It has 31 large families and only two of them are in the 30–160 bases range. It has 103 satellites with repeats longer than 150 bases.

Satellite families in *C. remanei* are of very different sizes (Fig. 1). It is the species with the largest number of satellites with long repeats, with 292 satellites with repeats longer than 150 bases.

### Comparison of satellite families in *Caenorhabditis*

**Fig. 4:**
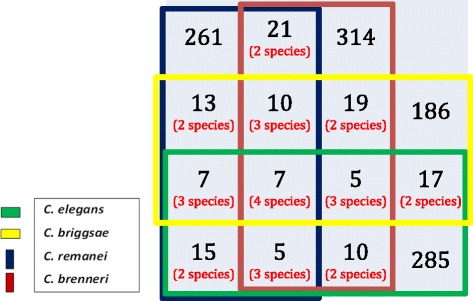
Venn diagram showing species-specific and shared satellite families between and among the four *Caenorhabditis* species studied in this paper

We compared the satellite families from the different *Caenorhabditis* species, using all against all pairwise alignments. We adjusted the score threshold to a random expectation (p-value) < 10^−4^. Once the homologues in different families had been identified, we built super-families, using a progressive multiple alignment approach. Only seven super-families include satellites from all four *Caenorhabditis* species, whereas 1,046 families are species-specific (75.3 % of the total number of families; Fig. 4 and Additional file 2: Figure S3). At the individual species level the number of species-specific families ranges from 67.6 % for *C. briggsae* to 78.3 % for *C. elegans*. Inspection of the results showed that most super-families only involved small families with a few satellites each (Additional file 3: Tables S2 and S6). We found several families with short repeats which had related families in other species, whereas all large families with repeats longer than 22 bases were species- specific, with the exception of two families in *C. brenneri* (10_24 and 3_28). Since there are 38 large families with repeats longer than 22 bases, 94.7 % of them are species-specific.

**Fig. 5:**
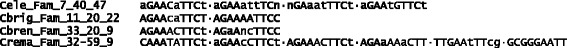
Comparison of *Helitron* related repeats found in different *Caenorhabditis*. The internally repeated palindromic sequence (AGAANNTTCT) is shown in bold. The different motifs are separated by dots

We were surprised that none of the *Helitron* families in *C. elegans* had any related families in the other species. We thus compared the sequence of all satellite families with the standard repeats of *Helitrons* and found that only Cele_Fam_7_40 had related families in the other species (Fig. 5). All of them are approximate multiples of a basic palindromic decamer sequence AGAANNTTCT.

Another peculiar case is the Cele_Fam_11_14 which has an (ACTACAA)_2_ repeat. We detected this motif as one of the most abundant sequences in *C. elegans* [34], where it is mainly found in chromosomes I and II. Strikingly, the distribution of this motif is similar in chromosomes I of *C. elegans* and *C. briggsae* (Additional file 2: Figure S4). However it does not form any satellite in *C. briggsae*. The significance of this evolutionary conservation is not clear.

### Comparison with *M. hapla*

We have compared the satellite families found in *M. hapla* with those of all *Caenorhabditis* (Additional files 1 and 4). We found that only 24 families of *M. hapla* satellites had some relation with *Caenorhabditis* satellite families (Additional file 3: Table S7). All the related *M. hapla* families had very few members; only three of them had more than three members. The longest repeat had 23 bases. Inspection of the sequences shows that most of them have long repeats of As or Ts. No clear evolutionary significance can be attributed to any of the repeats we have found. In summary these observations indicate that, as expected, the set of satellites in this species is largely unrelated to the set in *Caenorhabditis.*

### Microsatellites in *Caenorhabditis*

For comparison we have also studied the microsatellite distribution in the different *Caenorhabditis* species (Additional file 3: Table S8). For reasons given elsewhere [14], we have chosen a minimum length of 24 bases in order to define a microsatellite. We have focused on repeats of 1–3 bases, as the number of microsatellites with longer repeats (4–6 bases) is quite small. As with satellites, the distribution of microsatellites is remarkably different in each species. For example the genomes of *C. briggsae* and *C. brenneri* contain many AG and AAG microsatellites, which are less abundant in the other two species. Thus, at the microsatellite level, these two species appear to be more closely related than any of them to *C. remanei. M. hapla* stands out for its low content in microsatellites. Surprisingly, in spite of its low CG content, it has very few A and AT repeats, but a large amount of random A,T sequences (W_24_ in Additional file 3: Table S8). A similar pattern is found when shorter microsatellites are studied [40].

### Satellites in mammalian genomes

Satellites have been extensively studied in humans [41, 42]. However each study uses different parameters to define satellites. For a fair comparison with the *Caenorhabditis* data, we applied the same algorithm to detect satellites in three different mammalian genomes. In addition, we have not considered the centromeric alpha satellites, mostly restricted to the centromeres. It is clear that the relative number of satellites with longer repeats is significantly lower in the mammalian species, when compared with *C. elegans*, especially for satellites with repeats in the 100–169 size range (Table 2).

We have determined the distribution of repeat sizes in human satellites (Additional file 2: Figure S5). Only a few scattered satellites are found in the size range 100–169 bases. Satellites with repeats 49–50 and 84 bases stand out by having a comparatively higher number of members. The main 49–50 satellite has 85.7 % CG and corresponds to part of the very abundant SVA transposon. The 84 satellite corresponds to the repeated motif of 28 amino acids, typically found in Zn-finger proteins. It is found mainly in chromosome 19 (100 cases). It is also present in mouse and rat, where it had been previously described as MMSAT4 and RNSAT1, which contain two identical 84 repeats [22]. The presence of a large cluster of genes encoding Zinc-finger proteins in those species had already been described by Castresana *et al.* [43].

## Discussion

### Satellites in related species

Our results show that 75.3 % of the satellite families are species-specific. We also find that there is a significant difference when we consider the 38 satellite families which have more than nine members and their repeat size is longer than 22 bases; in this case 94 % of the satellite families are species-specific. A striking example is Cele_Fam_1_35, the largest family in *C. elegans*, which has no related satellites in any other species. The identification of satellites with a common origin has been performed using a rigorous statistical framework and it is improbable that we have missed related satellites.

According to the library hypothesis [44], the ancestor of *Caenorhabditis* should already contain most satellites, which would develop at variable degrees in different species. Although we do find satellites which are conserved across different species (Fig. 4 and Additional file 2: Figure S3), most satellite families are clearly species-specific. It appears therefore that the library hypothesis cannot be generalized to all satellites.

Our results also indicate that the precursor of *Caenorhabditis* probably had many satellites which were lost during evolution, since we have not detected any conserved large satellite family shared by all or some species. This lack of conservation does not allow reconstructing the phylogenetic relationships between the species, except for *C. elegans,* which shares fewer satellites with the other *Caenorhabditis* species (Additional file 2: Figure S3), in agreement with its position in the phylogenetic tree (Figure 3).

### Comparison with mammalian satellites

The distribution of satellites in mammalian species is very different from that found in *Caenorhabditis* and nematodes in general (Table 2 and Additional file 2: Figure S4). The very low frequency of satellites with long repeats is particularly striking; on average there is only one satellite in every 90 Mb of the human genome, compared to one in every 1.15 Mb in *C. elegans*. Probably more satellites with long repeats will be discovered when the heterochromatic regions of acrocentric chromosomes become available. In our study we have excluded the alpha satellite, with a repeat of 171 bases. It occupies several Mb in the centromeric region of each human chromosome, but is practically absent in other chromosomal regions. We have only detected six satellites with this repeat in human genome regions outside the centromeres. It may be that scattered satellites with long repeats are eliminated, because they interfere with the centromeric role of alpha satellites. The wide distribution of satellites with long repeats in *Caenorhabditis* might be related to their holocentric structure, a question which we will discuss in more detail in the next section.

### Centromere-like satellites

There is an ongoing debate on the location of centromeres in holocentric chromosomes. It is generally accepted that centromeres should be found in regions which contain the CENP-A histone. The results of Gassmann et al. [19] suggest that centromeres are randomly positioned on the mitotic chromosomes. Recently Steiner and Henikoff [39] have presented an alternative model. They find strong evidence for localized individual CENP-A proteins, but they find 708 preferred positions, whereas in mitosis there are only 50–100 kinetochores in each half spindle [45, 46]. As described in the results section, the sites of localization reported by the aforementioned authors [39] do not coincide with satellite positions; they may be related to the position of CENP-A in interphase. The details of CENP-A synthesis and deposition on chromatin during the cell cycle are not known in *C. elegans*. The complexity of these processes in other species has been recently reviewed by Catania and Allshire [47]. In particular it has been shown that CENP-A undergoes important changes during the cell cycle [48]. Its deposition on centromeric DNA is also related to transcription [49]. Furthermore, CENP-A plays a general role on chromosome condensation [50] and in double-strand break repair [51]. Thus the studies mentioned above on CENP-A localization are not conclusive, since they have been carried out in whole embryos, not in mitotic cells. Further studies should be done on isolated mitotic cells.

Our results suggest an alternative model for point centromeres: the centromere-like satellites which we have described may accumulate CENP-A and promote kinetochore formation during mitosis. They have sequence features similar to species with monocentric chromosomes [35, 38] and are also found in regions with high CENP-A affinity (Table 1 and Additional file 3: Table S3). Satellites with a similar repeat size have also been found in the other species we have studied (Fig. 1 and Table 2). In favor of the model we suggest, it should be noted that proteins similar to all those involved in mitosis in monocentric species are also found in *C. elegans* [52]. Furthermore holocentric plants are known to have dispersed satellites with a similar repeat size [53] which is 178 base pairs in the case of *Luzula nivea* [54]. Finally it is interesting to note the unique case of holocentric insects, which do not need CENP-A for mitosis [55].

### Origin and expansion of satellites

The sequences of satellite repeats from different families are clearly different. The first step in the birth of a satellite should be one duplication event, so in principle any sequence may become a satellite. However, it is likely that satellite expansion is favored by local sequence features, such as the short A-tracts or palindromes [56], which are frequently found in many satellites.

**Fig. 6:**
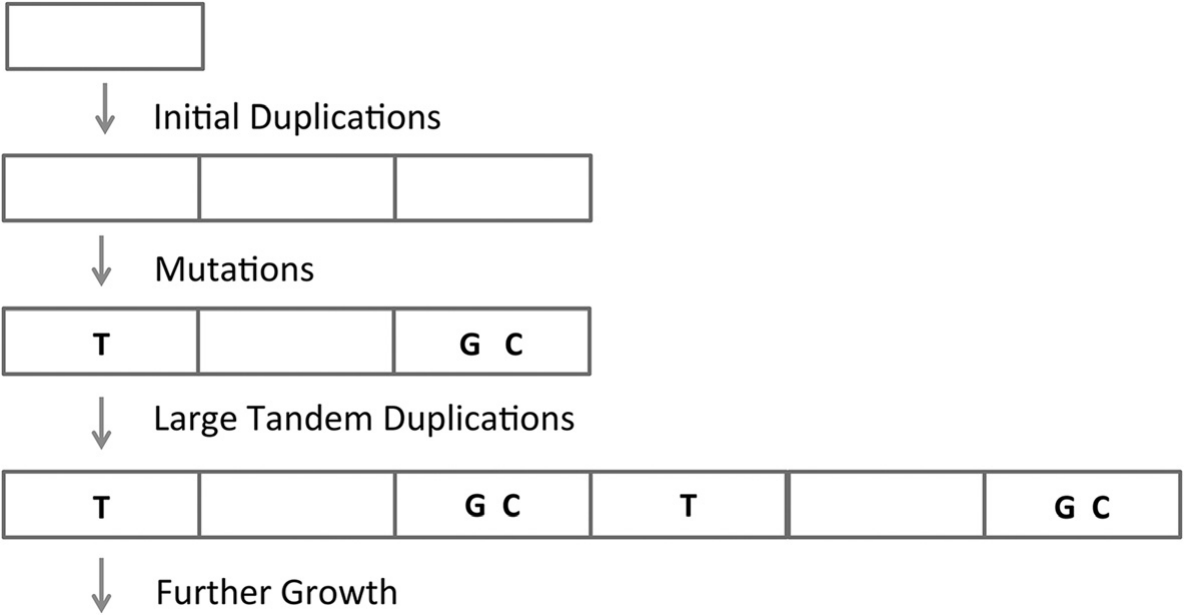
Model for the expansion of satellites. Each repeat is indicated by a rectangle. A relatively small satellite (three repeats in the figure) is transposed to another location. In the course of time it may accumulate some point mutations, indicated by the letters drawn inside the rectangles. Later the satellite increases in size. This process can be followed by the distribution of point mutations (Additional file 3: Table S5)

The satellite expansion process may be different depending on the repeat size. Shorter repeats usually give rise to shorter satellites. They may expand by replication slippage, as it has been suggested for microsatellites [16]. In the case of the Cele_Fam_24_19 (which we have described in detail in Additional file 3: Table S5), it appears that the ancestral satellite was rather short. Later it was copied into other points in the genome and increased in size. Such increases often took place with segments of 100–300 bases in length (Fig. 6). This size corresponds to the minimal size of homology for recombination to occur, as found in several species [57, 58].

Satellites with long repeats should expand by different mechanisms. Repeat expansion may occur at either the mitotic or meiotic level. Errors may take place associated with either DNA unequal crossing-over or duplication, including the repair of double strand breaks [59]. Various models have been discussed by different authors [1, 43, 60]. An alternative mechanism is expansion of satellites through Okazaki fragments, which usually have an approximate size of 200 bases. This possibility has been investigated by Shah et al. [61]; an Okazaki fragment may be duplicated during DNA synthesis. Upon association with the forward strand, a bulge would be formed which could be later repaired. For this process to be effective, the individuality of the repeats should be preserved, so that satellites accurately conserve the size of their repeats. This could be due to some feature of the satellite repeats, such as a palindromic region, which would determine the exact size of the Okazaki fragments involved.

In the case of very long satellites, duplication of a long stretch containing many repeats may take place in a way similar to gene duplication, which is very common in *C. elegans* [8]. Several processes such as unequal crossing-over, DNA transposition and retro-transposition may be involved [62].

### Transposition and turnover

It is clear that some satellites in *C. elegans* have been propagated throughout the genome by *Helitron* transposons, although other transposition mechanisms may also be active. For example, in the case of Cele_Fam_24_19, some satellites are found associated with *Helitron* transposons (Additional file 3: Table S4). Other mechanisms of transposition in *C. elegans* and other species have been reviewed by Huang et al. [63]. The observation that in the silkworm (a holocentric insect) most *Helitron* families experienced a single burst of expansion in the past two million years [64] is of particular interest; if transposons in *Caenorhabditis* have a similar life span, bursts of transposon-associated satellite expansions may explain the large differences in the satellite families present in each *Caenorhabditis* species. Individual families may have appeared on different occasions during the evolution of *Caenorhabditis.*

It would be of great interest to determine the extent to which the satellites vary among different *C. elegans* strains. In this sense full genome sequencing and assembly of different wild-type C. elegans strains [65] will be very helpful, in particular since a whole genome sequence of the Hawaiian strain has been recently released [66]. The results of Maydan et al. [28], which we have analyzed (Additional file 3: Table S1), provide a first step in this direction.

### Satellite elimination

In all *Caenorhabditis* there are many widespread families of satellites. In the case of *C. elegans* there are twelve families with more than thirty members distributed over all chromosomes (Additional file 3: Table S2). Individual satellites may be either eliminated by excision or may accumulate mutations and lose its repetitive nature. The repair of double strand breaks [59] may also contribute to either partial or total elimination of satellites. As an example we present some sequences which may correspond to degraded Cele_Fam_24_19 satellites (shown at the end of Additional file 3: Table S5). An intriguing case is the short motif ACTACAA, found in Cele_Fam_11_14 and as clusters of isolated motifs in other species, as in *C. briggsae* (Additional file 2: Figure S3). It is not clear if this motif either corresponds to degraded ancestral satellites or if it has some unknown function.

Finally we should mention that the elimination of satellites by random drift may not be sufficiently effective to result in the complete disappearance of large satellite families, which may have hundreds of members: an additional mechanism might be required. It is possible that satellite elimination by excision may occur in a concerted manner, similar to the programmed DNA elimination process described in some nematodes and in many other organisms [67]. Unfortunately the molecular mechanisms involved in DNA elimination are not known. It is possible that once the genetic load of some satellites increases, a process of specific DNA elimination is triggered during meiosis. Our data suggest that many ancestral satellites disappeared, while every species developed new families, in a seemingly random fashion. Note that this mechanism differs from the DNA elimination processes previously described, in which there is a selective loss of genomic regions. Elimination of specific satellites may be triggered by particular chromatin structures, resulting from repetitive sequences. A possible mechanism could involve extrachromosomal DNA circles, which have been found to originate from satellite regions of plants [68] and yeast [69]. The presence of palindromic regions would facilitate their formation.

## Conclusions

1. Each *Caenorhabditis* species studied contains a large number of satellites. About 90 % of the satellites in each species can be grouped into families of related satellites with similar repeats. A few of these families contain several hundred related satellites.
2. In *C. elegans* we have defined a subset of large satellites that have strong affinity for CENP-A. These satellites may facilitate the formation of kinetochores in mitosis.
3. Other satellite families in *C. elegans* are either related to *Helitron* transposons or to meiotic pairing centers.
4. The main satellite families found in different *Caenorhabditis* species appear to be unrelated. In particular there are some extremely large families of satellites which are species-specific. They have undergone an explosive expansion, perhaps because they have acquired a yet unknown function.
5. Our analysis of the internal structure of satellites demonstrates that they expand by the duplication of segments of about 100–300 bases.

## Availability of supporting data

The data sets supporting the results of this article are available in its additional files. The source code for the programs used has been deposited in the Dryad Data Repository (http://datadryad.org).

## Additional files

Additional file 1:
**Files containing the description of all satellites and their families in several formats (31 files).** (ZIP 1068 kb) Additional file 2:
**Supplementary Figures.** (PDF 568 kb) Additional file 3:
**Supplementary Tables.** (PDF 802 kb) Additional file 4:
**Satellite families in** ***Caenorhabditis*** **and in** ***M. hapla*** **.** (PDF 268 kb)
