# Supplementary material for: High evolutionary turnover of satellite families in Caenorhabditis
Source: BMC Evol Biol. 2015 Oct 5;15:218. doi: 10.1186/s12862-015-0495-x (PMC4595182; doi:10.1186/s12862-015-0495-x)
Supplement: Additional file 2: — Supplementary Figures. (PDF 568 kb) [file 12862_2015_495_MOESM2_ESM.pdf]

“High evolutionary turnover of satellite families in *Caenorhabditis*”

JA Subirana, MM Albà and X Messeguer

Satellite families

These tables are also available in Additional file 1.
With different formats: Fasta and ASCIII text.

C. elegans

Genome	Family name	Number of sequences	Alignment length	Score	Consensus without gaps
Cele.WS201	Cele.WS201_Fam_1_35_213	213	56	0.455995	CCGATTTGCCGGAAATTTTcAATTcCGGCAAtTTG
Cele.WS201	Cele.WS201_Fam_2_12_203	203	17	0.556588	CTAAGCCTAAGC
Cele.WS201	Cele.WS201_Fam_3_34_124	124	48	0.501228	GAAATTCAAATTTTCtGnGAAAAnaTTTTGGCGG
Cele.WS201	Cele.WS201_Fam_4_11_82	82	14	0.622259	GTACTGTAGGg
Cele.WS201	Cele.WS201_Fam_5_15_65	65	15	0.865855	TCGTGGtGAGACCCa
Cele.WS201	Cele.WS201_Fam_6_19_51	51	30	0.409142	AAAATCgATAAtTTtCngA
Cele.WS201	Cele.WS201_Fam_7_40_47	47	42	0.770906	AATTTTCtCGAaTTTTCtAGAAaGTTCTgGAACATTCCAG
Cele.WS201	Cele.WS201_Fam_8_25_38	38	29	0.698648	TTTACTCTCTGGCTTCaCantaTAT
Cele.WS201	Cele.WS201_Fam_9_43_36	36	51	0.566459	TTgCCGgTTTGCCGATTTGCCGGAAAtTTTcAaTTcCGgCAAt
Cele.WS201	Cele.WS201_Fam_10_19_36	36	21	0.727312	TGttAcACGAAAAATAGAT
Cele.WS201	Cele.WS201_Fam_11_14_33	33	17	0.699941	CAAACTAcAAACTA
Cele.WS201	Cele.WS201_Fam_12_16_29	29	16	0.740353	CTACaTGCCTACaTgC
Cele.WS201	Cele.WS201_Fam_13_32_22	22	36	0.746352	CTACAAACTACAAgTTTTGGGCGCTGCTAAgA
Cele.WS201	Cele.WS201_Fam_14_17_18	18	21	0.577342	CGTAGTAAAAGAgAnnC
Cele.WS201	Cele.WS201_Fam_15_17_17	17	20	0.734804	TTGACTACCTTtGcTCA
Cele.WS201	Cele.WS201_Fam_16_19_15	15	19	0.719298	GcCAAgGCaCtaGAAtTGC
Cele.WS201	Cele.WS201_Fam_17_20_14	14	26	0.551212	TTTGAAtTTTTnAGtcAAAA
Cele.WS201	Cele.WS201_Fam_18_11_14	14	11	0.896104	GCACATTTTTT
Cele.WS201	Cele.WS201_Fam_19_59_13	13	59	0.784442	AAAACCAGTGcAcatTTgAAatTCCATaTTnTCCATAATTCTCaGTTTaAAAAatTTTG
Cele.WS201	Cele.WS201_Fam_20_37_13	13	42	0.710317	aTTTTGGCGGGAATTCAAAtTTtAAtTTTTtnAAaAT
Cele.WS201	Cele.WS201_Fam_21_26_13	13	27	0.730294	cAGAGTCAcTAttTTTGGTGAAnGGt
Cele.WS201	Cele.WS201_Fam_22_22_13	13	25	0.504359	GTAAATCTACAnagaaActccc
Cele.WS201	Cele.WS201_Fam_23_12_13	13	15	0.591738	CCTAtACCTAtA
Cele.WS201	Cele.WS201_Fam_24_19_12	12	23	0.775582	TTTATTTATTTAATTAATA
Cele.WS201	Cele.WS201_Fam_25_18_11	11	21	0.711400	TTAGCAGCGcCCAAAAAa
Cele.WS201	Cele.WS201_Fam_26_31_10	10	32	0.784259	TAAATAtTTAGCAGACCAAagtTgGGTaTGC
Cele.WS201	Cele.WS201_Fam_27_26_9	9	26	0.861823	TTAGACCAtTAGATGAATTTccAAAG
Cele.WS201	Cele.WS201_Fam_28_12_9	9	19	0.412281	cTACcTACCTat
Cele.WS201	Cele.WS201_Fam_29_10_9	9	11	0.717593	GCGgtAGCGG
Cele.WS201	Cele.WS201_Fam_30_10_9	9	10	0.888889	CgATCCATCC
Cele.WS201	Cele.WS201_Fam_31_46_8	8	47	0.692249	ATCCTTcggAgCTGAAGGATCAGAnncAtCAGgAGGTACCCtTntG
Cele.WS201	Cele.WS201_Fam_32_12_8	8	16	0.598586	ATACATATACAt
Cele.WS201	Cele.WS201_Fam_33_12_8	8	15	0.520635	tAAAAAtaAAaA
Cele.WS201	Cele.WS201_Fam_34_163_7	7	168	0.701909	ACTTTTGAATTTTTGAAAAaAaCAGCTTTTgaTnAtATTtTgaAATTTTTtAAAAtAaACTTCCGTACAAGCTAgcATActTTGtTTGGtATtTTCTGGTAtaTTtGactATGCTGAACAgTTTTaTgAAATAAAAAAGAATgaCTcCATATGATTTTCccgT
Cele.WS201	Cele.WS201_Fam_35_63_7	7	68	0.663632	GGTCTGCTAAATcnaAnnTTGTAGTTTGTAGTCTAGCAGACCAaaTTTntTCAAAAccnctTg
Cele.WS201	Cele.WS201_Fam_36_35_7	7	36	0.649471	tTtncaAAACCGGCAATTgtCGaAATTGCCGgAAA
Cele.WS201	Cele.WS201_Fam_37_35_7	7	35	0.691156	TTGCCGGAanTTTTnATTTTCGgCAAaTTGtcnaT
Cele.WS201	Cele.WS201_Fam_38_31_7	7	31	0.918075	TTCTGAAaGAGCTTAAAATTTACTACAAAGT
Cele.WS201	Cele.WS201_Fam_39_26_7	7	26	0.733211	CATTgCGGCgGATTtTagaAAAaTGG
Cele.WS201	Cele.WS201_Fam_40_12_7	7	17	0.539683	GAAATTTTTTtt
Cele.WS201	Cele.WS201_Fam_41_42_6	6	42	0.682540	CcgaAAATtTccAAAACCGGcAATTGCCaAAATTGCCgaTTG
Cele.WS201	Cele.WS201_Fam_42_27_6	6	27	0.665021	AAATcGccaaAATTGaAAATTTCCGGC
Cele.WS201	Cele.WS201_Fam_43_20_6	6	20	0.888889	GGCAGACCTAGCCTACCCTA
Cele.WS201	Cele.WS201_Fam_44_184_5	5	189	0.846208	GAATACAGAACACCAaTTATGtCCGAGAAAAAGATCCnAcTCAGAATAtnAACATAntCgAAttTgtCgGAtGTATAaAGATTCCCGAAGAcaCTTTCCAATTAcCCAAAttGTTCAtATTCTAAAtnAnAtTCTCTTACTaGAACaCTCTTGGCCAAtGTACGCaGCCGAACGTATCATAAGt
Cele.WS201	Cele.WS201_Fam_45_95_5	5	95	0.884912	AGGGCCGCAGGCCcGAAcACTCtTggAAAcTgtGGGCcagCTTTAATCCTATTCCAAGcAgATTGTTATTCgCTCAAGCCTAccAACCAgGTTTG
Cele.WS201	Cele.WS201_Fam_46_22_5	5	24	0.694444	agTACTGTAgggGTACTGTAgn

Cele.WS201	Cele.WS201_Fam_47_19_5	5	22	0.578788	TTTCTaGGCcATCgAtata
Cele.WS201	Cele.WS201_Fam_48_20_5	5	21	0.638095	GTAAATctACAtttgcttCC
Cele.WS201	Cele.WS201_Fam_49_94_4	4	94	0.933806	TCAAAATCTTCTgTTCTCTAAATATTGGGTgCTTTTCGATGCcCTaTGTAGACAAATCAATGGGAAAATcaTCAATTTCTGaAGGCAGTAaTTC
Cele.WS201	Cele.WS201_Fam_50_40_4	4	41	0.796748	TAntTTTTaAtgTGaCCTACTtaAAAAGTAGGTCATGACC
Cele.WS201	Cele.WS201_Fam_51_27_4	4	27	0.720165	AgaCTaCCTAgaCTACCTAgaCTaCct
Cele.WS201	Cele.WS201_Fam_52_25_4	4	25	0.920000	TATTTACTCtAATgTTCTGCCAATt
Cele.WS201	Cele.WS201_Fam_53_20_4	4	24	0.479167	tgnCCTAaAAAAATtgATaa
Cele.WS201	Cele.WS201_Fam_54_22_4	4	24	0.539352	CCACcntaagAAAgTTtCTAGG
Cele.WS201	Cele.WS201_Fam_55_24_4	4	24	0.694444	aaCCTAAGCCTAAgCctgagCcTA
Cele.WS201	Cele.WS201_Fam_56_22_4	4	22	0.560606	AAaTcgcaAAATTTcnaAnAAa
Cele.WS201	Cele.WS201_Fam_57_20_4	4	20	0.697222	CTCAacCaAAAaTcCTcAGC
Cele.WS201	Cele.WS201_Fam_58_18_4	4	18	0.629630	CtAaGccTaaGCCgAaGc
Cele.WS201	Cele.WS201_Fam_59_16_4	4	16	0.729167	TaAGGATCTaAggATC
Cele.WS201	Cele.WS201_Fam_60_16_4	4	16	0.805556	AAtTTTTCaGCaGCaA
Cele.WS201	Cele.WS201_Fam_61_14_4	4	14	0.730159	aCGCTaTacGCTaT
Cele.WS201	Cele.WS201_Fam_62_12_4	4	12	0.814815	CAcGCAACGCga
Cele.WS201	Cele.WS201_Fam_63_11_4	4	11	0.898990	TGGAGCGGTtT
Cele.WS201	Cele.WS201_Fam_64_10_4	4	10	0.866667	ATtcCAGAAA
Cele.WS201	Cele.WS201_Fam_65_174_3	3	183	0.826958	TTAATCAGAAATATATTGAACTTTTGAAACATGTCtCAAcTTtTaTTTTTTTTAGAttACGATGgCtCGTTTTTCAAGCTATGAATAACTTTCAAgTTTTTTGAAATAActGGAACTATTTTTTaaAATGaGtGTgTATATGtACaaACGCATgGCATTCATATACATATAGAT
Cele.WS201	Cele.WS201_Fam_66_168_3	3	172	0.746770	TTTATTTTTGTTTTTtTGtGCCTacaTATAcCTGTtgAAATTGGAAtTCaAgACAAAAACCcCTnTAGCGCgtCTgAAAaTtgaTaTCtaGGTcCAnAAACTTaCCAAtAATATTTtaAAAnAAtCtAGAATCCAAATCTATcAAAAcATTTCCggAATTCCAtAATt
Cele.WS201	Cele.WS201_Fam_67_163_3	3	169	0.722551	ATTTTTGACAtAtcTattCTCTCTTTTTTTTtTaAcATgTAgTTCAGTGAcAactgacTCgAAAATATAaTGcTcGTAATTATTnaTAAgTgTgTgTTGTCTCaGAGCCAngTCTGAAAAaACAGATTTTTTtTcGAAAATTTcAAAAAntTcGTAgAAATTcG
Cele.WS201	Cele.WS201_Fam_68_79_3	3	79	0.909986	gTGAATCTCCtCTCTaCCtAATGAGTcCTGACAGAATACCGGCAGTATTCTGGGGTCTCATTAGGTTtTaGTTTCTTcT
Cele.WS201	Cele.WS201_Fam_69_76_3	3	76	0.918129	AATGCTAGAgGACACTCcTTTGCACGAAGGgAAGTGGGAGAGACTGTGGaGTTGTCGGTGTCTtTGTgCATGCTCg
Cele.WS201	Cele.WS201_Fam_70_66_3	3	68	0.879085	AATCAACCTATTCAGTTGACAATGGTTTTCTTTTAtCAAAtTTtTtAaAATTTtAAAGAAAACACA
Cele.WS201	Cele.WS201_Fam_71_64_3	3	64	0.986111	GTAGTCAAAAAGaCAAAAGTGCGAATGTAGCTATTTTTTCAGTCAGTAGCGAAAAAGACATTCA
Cele.WS201	Cele.WS201_Fam_72_48_3	3	48	0.796296	AATTtAAtTtTTGAtTTgCCCGTAATTTATAttTTTCTGAAGATgagc
Cele.WS201	Cele.WS201_Fam_73_42_3	3	46	0.603865	TTGcCgAAnTTGCcgAAcCTnAAAATtTTcGGCAAccGGCAA
Cele.WS201	Cele.WS201_Fam_74_33_3	3	33	0.734007	CTgAAtATTTtCgACgaAAAATTCaAATTtTCt
Cele.WS201	Cele.WS201_Fam_75_32_3	3	33	0.626263	cCAATTTTTAngCTaAaAaTCnCTtAAAAact
Cele.WS201	Cele.WS201_Fam_76_31_3	3	32	0.812500	AGAGATGAGTTGTCgAGCGAGAcctCAGAAg
Cele.WS201	Cele.WS201_Fam_77_28_3	3	31	0.573477	aTcGATTTTacntTGAAAAtCgGaAATT
Cele.WS201	Cele.WS201_Fam_78_31_3	3	31	0.770609	GGTTACTGTAACTCAAtcctagGATtcTTCG
Cele.WS201	Cele.WS201_Fam_79_30_3	3	30	0.703704	TTTCTAGAAAaatcTGgAAAcTTcTaGAat
Cele.WS201	Cele.WS201_Fam_80_26_3	3	26	0.863248	cAAAACCGGCAAtTGCCGaAAATTCc
Cele.WS201	Cele.WS201_Fam_81_21_3	3	23	0.700483	CTACAaACTACAAAATnaAaA
Cele.WS201	Cele.WS201_Fam_82_18_3	3	22	0.616162	GTcGATTTACGAGnTTTT
Cele.WS201	Cele.WS201_Fam_83_21_3	3	22	0.767677	tGGCAaCTTTCTacCGAAATT
Cele.WS201	Cele.WS201_Fam_84_21_3	3	21	0.751323	AAcTTTtTgGtAAAATtTGAt
Cele.WS201	Cele.WS201_Fam_85_20_3	3	20	0.733333	CGAtAtaGCTAtAAACgAtA
Cele.WS201	Cele.WS201_Fam_86_17_3	3	18	0.660494	AanTAGGTCAagTTAAA
Cele.WS201	Cele.WS201_Fam_87_16_3	3	16	0.722222	TTcCAGGgTtcCAGGg
Cele.WS201	Cele.WS201_Fam_88_16_3	3	16	0.722222	aATTcCGGcAAtCgGC
Cele.WS201	Cele.WS201_Fam_89_14_3	3	15	0.651852	AATTtTaAATTggA
Cele.WS201	Cele.WS201_Fam_90_11_3	3	11	0.717172	AGGcAGGCaaT
Cele.WS201	Cele.WS201_Fam_91_226_2	2	226	0.660767	AAAtAgttCatcTaCAGcccTAaCtTTTaGTGCGTATTTTATTATTAAATGAAAACgACCATTTATAaGCaAAAATAGaTaaATTTacGaAAcTTTGAAAaaTCacAAATCaCTTCAAAccAACTTTTTTagAAAagTCTTTCAGAAACTTTGTaGcAAATTccAAGCTcTTTCaGAAgaTAccAAaAAcATcCCAGTAGGTaCaAGAAGCTcCaaGcAGTTACAG

Cele.WS201	Cele.WS201_Fam_92_168_2	2	168	0.761905	AATTcAaTTTTTTACATACATAtgacTGCaCTTTTTTGCAAAAaAAAAAaTTTGGAATTTTGGGAGAATTTTTATaAAAATGGATTGCTAATTTgTTTTTAGTATTATgGGTACGATTcgGgacCaaGAaAtTCaATTTTcAGCCcCGCTAcAGGcGTTTTgGTCTTc
Cele.WS201	Cele.WS201_Fam_93_86_2	2	86	0.697674	TGTATTTTGAAAGGTCaTcgCAATAggGAAAAGATGGAAGAgttCgaagtTAaAGAaAAaCTATTGccAGACCCCGAAATACTGGC
Cele.WS201	Cele.WS201_Fam_94_69_2	2	69	0.652174	TGTAGTTTGTAGTGTGttAGcCTCAACCAAAAttaattTaTTTTTaaTTcGTTGAGGCTaAgAAAATaT
Cele.WS201	Cele.WS201_Fam_95_69_2	2	69	0.683575	TTTCCGGCAAAccGGCaAATTgccgtaATTgAAAaTTcCaaCAAATCgGCAAATTGCCGGAATTGAAAa
Cele.WS201	Cele.WS201_Fam_96_44_2	2	44	0.681818	AAAtTGGCaAATCGgCAATTTGcCaAAAAtCAAaATTTTcCGGa
Cele.WS201	Cele.WS201_Fam_97_44_2	2	44	0.909091	CTGTTTTCTGAGCATTGTaGaaTTTCGGATTGATGGCCGAAAGG
Cele.WS201	Cele.WS201_Fam_98_43_2	2	43	0.782946	CGAGcTcGGCaaACaGCAAATTcGGCAAAaTCGGCAAATTTGC
Cele.WS201	Cele.WS201_Fam_99_41_2	2	41	1.000000	TCCCGTTTTCCCGTTTTTAAAATTTTCACGGGAACGGGAAT
Cele.WS201	Cele.WS201_Fam_100_39_2	2	39	0.863248	TTATGaAAAAGTGGCTCTAgaGCCACcAGAAATACCAGT
Cele.WS201	Cele.WS201_Fam_101_39_2	2	39	1.000000	GTAATTTATAATTTTTTAAAGATGAATAATAGATTGCCC
Cele.WS201	Cele.WS201_Fam_102_37_2	2	37	0.914414	tTTTTaAAAAATTGATAAAAATATATAAAAGCTGATT
Cele.WS201	Cele.WS201_Fam_103_37_2	2	37	0.747748	TTTTTAGTGAAaaGCCAGTaTTTcagAAATTGGCAAa
Cele.WS201	Cele.WS201_Fam_104_35_2	2	35	0.809524	GTAATAAGAAAATTTCaAACTTTTTTcACGaGTaT
Cele.WS201	Cele.WS201_Fam_105_35_2	2	35	0.809524	CaAGTAGATTcTTTCCGCCGGTTCGATTTTcTCCa
Cele.WS201	Cele.WS201_Fam_106_33_2	2	33	0.601010	AGCAGCCGACaCCTgcCGGGTcGCTaCAgtttt
Cele.WS201	Cele.WS201_Fam_107_33_2	2	33	0.626263	CTACAGTAACCcaACAGTAccacTAaAGTATtt
Cele.WS201	Cele.WS201_Fam_108_33_2	2	33	0.621212	aaCCAATCAGCaaTgAGCTCTgCCcATTTcTcA
Cele.WS201	Cele.WS201_Fam_109_32_2	2	32	0.916667	CGACCTGCGACCTAGCGACCTaGGTCGCaTTG
Cele.WS201	Cele.WS201_Fam_110_31_2	2	31	0.806452	CGATTTTCTgGaaaAAAAAACTAAAAAATTC
Cele.WS201	Cele.WS201_Fam_111_29_2	2	29	0.908046	aaGGATTTCCCACTATGTCAATACATTGT
Cele.WS201	Cele.WS201_Fam_112_28_2	2	28	0.601190	CAAAgaTGCCGAaTTacCCGAGaTcGGa
Cele.WS201	Cele.WS201_Fam_113_28_2	2	28	0.785714	CACTGaCCAAAGAAAAAcAaaACACGTG
Cele.WS201	Cele.WS201_Fam_114_27_2	2	27	0.703704	CCTaCagTGCCTaCCgTGCCTACCgTA
Cele.WS201	Cele.WS201_Fam_115_27_2	2	27	0.703704	TCCTGGgGcTCCTGaTGGTCCcggTGG
Cele.WS201	Cele.WS201_Fam_116_26_2	2	26	0.794872	TGAAATTcCACAAAaTATcGATTTTc
Cele.WS201	Cele.WS201_Fam_117_24_2	2	24	0.625000	TTgaAaTTTTTCAAcGAAAAAAaa
Cele.WS201	Cele.WS201_Fam_118_23_2	2	23	0.652174	TcaaCGGATTTTTaGGcTTAAAA
Cele.WS201	Cele.WS201_Fam_119_23_2	2	23	0.710145	CCgGCGTGGTaTTCAAcGCGTTc
Cele.WS201	Cele.WS201_Fam_120_23_2	2	23	0.681159	GGTGGCCgAaaTTTTTAaAGaTC
Cele.WS201	Cele.WS201_Fam_121_23_2	2	23	0.652174	AGGCATGAGGaAGGCgcaTAacC
Cele.WS201	Cele.WS201_Fam_122_23_2	2	23	0.652174	GTACTGTAGCaaaTCAAGaTaGa
Cele.WS201	Cele.WS201_Fam_123_22_2	2	22	0.734848	TGGcTGAGGCTCTCTagATTTt
Cele.WS201	Cele.WS201_Fam_124_22_2	2	22	0.795455	AATTTTTGaTCAAAAgCGGACt
Cele.WS201	Cele.WS201_Fam_125_21_2	2	21	0.650794	AcAAACTACATTatGcAgACT
Cele.WS201	Cele.WS201_Fam_126_21_2	2	21	0.650794	cAAAAATCgACTaAAAAaaGT
Cele.WS201	Cele.WS201_Fam_127_21_2	2	21	0.690476	TGGCTaAAAaTaGaGAATTTt
Cele.WS201	Cele.WS201_Fam_128_21_2	2	21	0.690476	TATCGATTTTTCaaCAgaTTt
Cele.WS201	Cele.WS201_Fam_129_21_2	2	21	0.873016	AGCAGCCGACACCTCAaaGGT
Cele.WS201	Cele.WS201_Fam_130_21_2	2	21	0.873016	TTTCCTAGTTGATaGCCGAGg
Cele.WS201	Cele.WS201_Fam_131_21_2	2	21	0.746032	AAAaTTGGCTTAAAATGgTca
Cele.WS201	Cele.WS201_Fam_132_21_2	2	21	1.000000	CAACTACCACAACTACCGTAC
Cele.WS201	Cele.WS201_Fam_133_20_2	2	20	0.633333	TTcTaAATTTTaAaGCATaT
Cele.WS201	Cele.WS201_Fam_134_20_2	2	20	0.666667	GGCCTaGAATcTCaCaACaT
Cele.WS201	Cele.WS201_Fam_135_19_2	2	19	0.614035	CCAACTTggGGTacaTAGG
Cele.WS201	Cele.WS201_Fam_136_19_2	2	19	0.798246	AATTTaCCAATTgACCAAa
Cele.WS201	Cele.WS201_Fam_137_18_2	2	18	0.666667	TaAaCCTAAGCTggAGCT
Cele.WS201	Cele.WS201_Fam_138_18_2	2	18	0.629630	aAaTTGCCGGAATTaCga
Cele.WS201	Cele.WS201_Fam_139_16_2	2	16	0.750000	TAaGCaTATAaGCTTA
Cele.WS201	Cele.WS201_Fam_140_15_2	2	15	0.655556	cAaAAaTGAAAATCt
Cele.WS201	Cele.WS201_Fam_141_15_2	2	15	0.733333	GGGGaCAACAaAAaA
Cele.WS201	Cele.WS201_Fam_142_13_2	2	13	0.641026	AaACgCCTATGGc
Cele.WS201	Cele.WS201_Fam_143_12_2	2	12	0.833333	ATTcTGGAATAT
Cele.WS201	Cele.WS201_Fam_144_12_2	2	12	0.680556	TAGCTcAGcTTt
Cele.WS201	Cele.WS201_Fam_145_11_2	2	11	0.696970	GTcGAAAAtTG

Cele.WS201 Cele.WS201_Fam_146_11_2 2 11 0.757576 CAGTTaTCACc
Cele.WS201 Cele.WS201_Fam_147_11_2 2 11 0.757576 TGACAACTTca
Cele.WS201 Cele.WS201_Fam_148_200_1 1 200 0.000000
TGTGGTCCAGAAGGTATGACTGAAAATTCACTTTTTCTGCTTGAACTTGGCAGATTTAAAATTTTTCAAAATGCTGTTTTCAAATTCTGAAAGAACTTAAAATTTGCTACAAAATGGCTACGGGAATTTCTAAAAAAGTTAGTTTCAAGCCAGAAATTAA
TTTTCAAAAATTGGAAAATCTATTAATTTTGTATATAAAT
Cele.WS201 Cele.WS201_Fam_149_185_1 1 185 0.000000
CTGTAGTTTTTTGTCGCATTAATGCAAATTAAACTTCATTCTATAGAAAAATTGTAGAGCATCTTTTTAAAATAAAAATGAATTCAAAATCTTAAAAATGAGCAAAGTTATGAAAGTTTGAAAATTTGAAATTTTCAACAAAAATTTTTTTTGGAAATTT
TGGGTCTCGGAAAGGTGCCGGGGTA
Cele.WS201 Cele.WS201_Fam_150_181_1 1 181 0.000000
TTTTTAACTTTTAAATCCACTAAAACTTGGAATGGTACAGAACATGCAAATTGTAATTTTAAGAACTGAATGTGGTTTCGAATATTCATGGATTGCTTTATTCGAACACTGACTCTGAAATATTTTGCATCCATTTGACTATTAATTGTCATATTATACC
GAACACTACTAACTTTACTGG
Cele.WS201 Cele.WS201_Fam_151_180_1 1 180 0.000000
CAAAATATCTCTAGTTTTTAAAAAACAGTTCAAATTTTGGGTTCACTCTTATTACTTTACAGTGATTGTTACATTGTAGACTCTGTGGACTTGTCCAACTGTCTACGACGGCATGAGTTTAGTAAAAAGGTTGTACCATGAACTGAATCAAATTGGAAAA
TTTGGAAAATCTGCGAGAAT
Cele.WS201 Cele.WS201_Fam_152_178_1 1 178 0.000000
ATTTCGGAATTAGTTTGAGAATAAAAGAATCCATTAGTATTTGAAATAACGTTCAGTTCTCGAAATTAGATCAGAATAGTTTTATAAAATCCCGAGTTTTGTGGTCTTACGAGTTAAAAAAAAAACATCGTAGTTTATTGCTATAAATTGACAATTAACA
ATTAAATGGATACAACAC
Cele.WS201 Cele.WS201_Fam_153_172_1 1 172 0.000000
CAAACATAGGCAACCAAAAATATTTTCCAAGTTTTTATTGATTTGTTGCACATTGAAAAAAACTTTTTTCGGGTTTTTTAAAATGAATATCATAGCTACAGAAACGGTTGTACACTTTTCTGAAAATACAAAAAAATTGCAATTTTTATAGCTAGGAGAC
TTTTTGAATGCC
Cele.WS201 Cele.WS201_Fam_154_161_1 1 161 0.000000
GCGTCGCGGCTAAATCTCAAAATTTGCCTTAATATGTTCAGGAAAGTACCACAAACTATGTTTTTTGAAATCTTCATAAAATTTCCCTTAATTTAAGCCCCATATTAACCTAGTTTGGATAAAAACTAACGAATTTCTAAATTTTCGGCGCGTTGCGTGT
C
Cele.WS201 Cele.WS201_Fam_155_156_1 1 156 0.000000
CGAAAATTTCAGTCATTTCTAATTTTTTTTGTTGCAAAATAAAAAAAAATAATTAAAAATTTATGAGCGTTCTATAATTTTTTTAACATTTATATTCGGACATTGCGACCCACATTTGATAATTTGAAGCAAAAAACTAAAGGACTCTACTTCAAAA
Cele.WS201 Cele.WS201_Fam_156_155_1 1 155 0.000000
TCCCGCTGTGGTCAAAAGTTTTTTGTGTGCTCAGTTCTGTACGACAAATGTGTTAAAATCTTCTGAAAACTCAAAATTCCAAGTTTTCCCAAAAAATGGGTCAAATGGTACAGTGGGATTTTCCACGAATTCTAAGTAGATGGTCGTGAGTTCAAC
Cele.WS201 Cele.WS201_Fam_157_151_1 1 151 0.000000
ATTTCAGGCTGGAATGGGCCGTTTTGGGCCGGTTGTAGGCCCGAATCTTCATGTTTTCTGAATACGCTGCACTTAACATTATTAAGCAAAAACTGAGCGTAGAATGTCCGTTAATTAGAACCCCCAATTGGCTAAATTCTGAAAAAGTTAC
Cele.WS201 Cele.WS201_Fam_158_145_1 1 145 0.000000
GTGGCCTAGAAAAACCTGAGCATGATTTTTCTAGGCCACCAACTTCTAACATTCATAACTTGGCGGAAAATCTACATAATAAAATTTTGCAAAAACCAATTTATTTGTCTAAAAAATTGCAATAAATTTGACCTACTTGATTTTGA
Cele.WS201 Cele.WS201_Fam_159_124_1 1 124 0.000000
AATTTTCAAAGTTTGGAAAATCTATTAATTTTGCATATAATTTTTATAAATTCTGAAAGAACATAAAATTTGCTACAAAATGGCTACGGGAATTTCTAAAAAATTTAGTTTGAAGACAGAAATT
Cele.WS201 Cele.WS201_Fam_160_121_1 1 121 0.000000
AAAAGATAGTTGCTCTTTGAGCAGAAGGTTGCAAACCGCAAGAGCAAACTTGCCAAATGACAAGCCACCCAAGTACTCGTTGCACCTACCTATCATAAGCTCGTAGACTATAATTTCTCTG
Cele.WS201 Cele.WS201_Fam_161_119_1 1 119 0.000000
ACTCCAATACCAACCGATGCTAAAAGATTCTGATCCCAATTATTTCTGAAATAACAGTTTCGGCTTGATGTACCAATGAGAGAGCACAGTGTAAACAAGTTTTCATAGTTCTGACGGCG
Cele.WS201 Cele.WS201_Fam_162_119_1 1 119 0.000000
GTTGCATATTTCGCAATAATTTTTACGGGAACGTAATCTCCACATCACCAGCTTTCATTTAAGTACCCACACGGCAAGGTTTAATTTTTGAAATTTGGTCGCGGATTTTCTCGGCCACC
Cele.WS201 Cele.WS201_Fam_163_117_1 1 117 0.000000
TGTTGGTTCGACAGTAGGTGCATTAGCCTCTGGAACTGGAACATCCTTTTGCTCGACGATTGCAGCTTGTTGTACTTCAGATGTTTCCTTTGATTCCACAGGTGCAAGTTTCTCAAC
Cele.WS201 Cele.WS201_Fam_164_114_1 1 114 0.000000
GTGTTTCAGAATTACATGGAGTCTCACTACTTGCAGAATTTGTGGCTGTAGACGATTGACCTGCTGTAGTTGAATCGCTTGAAACTGTCATAGTGGATGTTCCATCAGACGTTT
Cele.WS201 Cele.WS201_Fam_165_111_1 1 111 0.000000
CTGTTGTTGATCTACTTAGCGTAGTCGTAGATTCTAAAAATTCAGTCTTTAATTTTTATTAATTTTTAAAAAATATTTATTTACCAGTAGTCGATACTGCAGGCGTCGTTA
Cele.WS201 Cele.WS201_Fam_166_108_1 1 108 0.000000
TTTCAGATTTTCAGATTTTCACTTGATTATATAGGTCACGACCCAAGTTATTTTGAAAATTGAGCTACACGGCTGTAACTTTGGAGCTATGGGTAATCAAGTATGAGC
Cele.WS201 Cele.WS201_Fam_167_108_1 1 108 0.000000
AAGTCTTCTTGGTTCTGTGTCTTTGCTTCGAAATGTTGACCATCTCCTTGCAGGATCTTATTGTCAGATGGAACCTTCTTGGCATACCTCTCTCCACTGATGTGAGTG
Cele.WS201 Cele.WS201_Fam_168_102_1 1 102 0.000000
TTCTCCACCAACTGGAACCACCGAAGAGCCTGGATCTTCAGTTGAGACTACACCATATACCGGTGAGACTCCAGTAACTTGGGGACCTCCAGGATCAACAGC
Cele.WS201 Cele.WS201_Fam_169_102_1 1 102 0.000000
TTGCTGCAGCATATCTTTCTCCAGTACCACCTTGATATGTCGAACGATTGATAGTTTGAGAATCAAGAGAATCAGTTGGACGATCAATTCCATCTTTGGGTC
Cele.WS201 Cele.WS201_Fam_170_102_1 1 102 0.000000
TGTACACTTCTCCCAAAATGACTGTAAGAATGGACTCCTTATATGATTTTAAACCCGACTCTGGACATACAAACTTCTCTCAAAAAGAAAATAGACTTCACA
Cele.WS201 Cele.WS201_Fam_171_101_1 1 101 0.000000
GGTTACTGTAGTTCCAAAAGTACGCAAACACTGACTACGGTTCCCTAATACATCAGATATTACGACAAAGTGCAGAAAATCGCTAAAAATGACTTTACTGG

Cele.WS201 Cele.WS201_Fam_172_96_1 1 96 0.000000
GTTGATTTGCGGCACTCTACAGATAGTGGATCTGCTTCTTACCAATCTTCCCGTTACCGCCACGCACTAAAGCTCCAAACTATTTTATTTTTTTGG
Cele.WS201 Cele.WS201_Fam_173_93_1 1 93 0.000000
GGCTTTGGAGTTGCTTTAGCTGATGTTGGCTTGGCAACTGGTTTCTTCTCGTCATCGCTTGAATCCGAAGAGGACTCGGCCTTCTTGGCAACA
Cele.WS201 Cele.WS201_Fam_174_91_1 1 91 0.000000
AATTTTTGAAATTAAATTCTGAAAGCGGTTAAAGTTTGAAACAAATCCCCGAAGAAACTTTTTCAAAAAAGTTAATTTCAAGTCAGATATT
Cele.WS201 Cele.WS201_Fam_175_89_1 1 89 0.000000
AGTATTTTGGGGTCTCTCCCTAGTTGTTAGGTTAACTTTATACTTTTCCTTCTTCTATTTCTTCGAGATGATCGGTCCAGAATACAGCC
Cele.WS201 Cele.WS201_Fam_176_85_1 1 85 0.000000
ATTTTGAAAATTAATTTCTGGCTTGAAACTAACTTTTTCTGAAATTCCCGTAGCCATTTTGTAGCAAATTTCAAGTTCTTTTAGA
Cele.WS201 Cele.WS201_Fam_177_84_1 1 84 0.000000
TAAAAAGTGGTCTAATTTTGTTGAAGACGGGTAATTCATATATGGAGAATTCAGAAAATCTAGGTTTAACCCATCAAAAACTAT
Cele.WS201 Cele.WS201_Fam_178_81_1 1 81 0.000000
TTATTTTTTCGATTTTAGCGAAAAATCAACACATTTTCGATCATTTTTGAACAAAAAATTGTTTTCTCAAAAATTTGACGC
Cele.WS201 Cele.WS201_Fam_179_80_1 1 80 0.000000
ACCGTAACCCTCTAGACAATCGAAAATTCAAATTTTCCGCAATTTTTCGTAAAATTCGGTGTTAGCGGACTTCTGGAGCT
Cele.WS201 Cele.WS201_Fam_180_80_1 1 80 0.000000
AATTTCTCAATAGCGCGCGCTTGCAACATCCGAACGGCGCGAAATTTGAATTTTTGGCCATAAAATAACGAAAATCGGCA
Cele.WS201 Cele.WS201_Fam_181_79_1 1 79 0.000000
ATTTTTTTTTAAATTGATAAAAATATATAAAAGCTGAATTTTTCAAAAATTCAAAAGTATGGGAAAATCATATGGAGTC
Cele.WS201 Cele.WS201_Fam_182_78_1 1 78 0.000000
CGAAGTTGCTGGATCAACGATCACAACAGGCGGATTTGTCTGCACAACCGGATCAGAAGTCGTCGTACTTGGATCTTT
Cele.WS201 Cele.WS201_Fam_183_78_1 1 78 0.000000
TGCAAGGCTCCGATGATGATACCACTTCTAAAGTCGAGGTGTCACAATTTTCGGGTGTCGTAGAACTTGGATCAGTAC
Cele.WS201 Cele.WS201_Fam_184_73_1 1 73 0.000000 ACTTTGTAGTTTGTAGTCTAGCAGACCAAAATTTATTAAAAACTGCAGAAAATTGATGGGTCTGCTAATTAAG
Cele.WS201 Cele.WS201_Fam_185_72_1 1 72 0.000000 TCCATCCAGCTGTGGTTGGTCTCTGGGTGAATCCTCGAGAAGTATCACGAGTAGTACCATTGGCTCCGTTTG
Cele.WS201 Cele.WS201_Fam_186_70_1 1 70 0.000000 AAAAATCGATGTTTTGAAGTTTTTCTCAATAGAGCGCACTTTCAACAGCGAGAAATTTGAATTTTCCACC
Cele.WS201 Cele.WS201_Fam_187_69_1 1 69 0.000000 CCGGAAATTTCTATATTCGGCAAATTACCCGTTTCACATAAATTTTCTTTTCTGCAAATTGTTGGTTTG
Cele.WS201 Cele.WS201_Fam_188_69_1 1 69 0.000000 TTGCCGATTTGCCGGAAATTTACATTTCGGCAAAGTGCCGTTTTTCCGGCAAAACGGCAATTTGCAGGT
Cele.WS201 Cele.WS201_Fam_189_68_1 1 68 0.000000 TTCTAGGCCACCATTCTCCTCCTCGTGACTGTGGGAGTATCTAACCCCACAACACAACTTTTTGGTTT
Cele.WS201 Cele.WS201_Fam_190_67_1 1 67 0.000000 CCATCCAAATTCCAATCGTTGGTGCATCATCTCCAGTCCCGAATCGCCATCTCCAGCAGCTCCCGAA
Cele.WS201 Cele.WS201_Fam_191_65_1 1 65 0.000000 TTTTTTGAAATTCTGTTTTCAAATTCTGAAAGAACATAAAATTTGCTACAAAATGGCTACGGGAA
Cele.WS201 Cele.WS201_Fam_192_64_1 1 64 0.000000 ATATACCAATGAGAGAGCACAGTGTAAACTAGTTTTCATAGTTCTCACGGCGACTCCAATCACT
Cele.WS201 Cele.WS201_Fam_193_62_1 1 62 0.000000 ATTTTCCATCAAAAATCGGCTGTAAAAACCGATTTTTAACTGAAAAAATCACAAATTTCTAC
Cele.WS201 Cele.WS201_Fam_194_62_1 1 62 0.000000 GTAAAAATGTTTGAAATTGTGAATTTTCAACATGAAAATGCCTGAAATTTTGAATTTCCTAT
Cele.WS201 Cele.WS201_Fam_195_60_1 1 60 0.000000 AGAAAATTGCACTTTTTCGGACAATTTTGAATTTTTACTAAAAAAATTGGATATTTTTGA
Cele.WS201 Cele.WS201_Fam_196_60_1 1 60 0.000000 ATTCAGTTGAGCTCGTTATGTCGGTTGAAGACACAATTGGAGTGGTGCTTGAGGAGCTGG
Cele.WS201 Cele.WS201_Fam_197_60_1 1 60 0.000000 CTGCTGATTCCGAGAAGAAGGCCCCAACCAAGAGAGAAGCTGATCCACCAAAGACTGAAT
Cele.WS201 Cele.WS201_Fam_198_57_1 1 57 0.000000 GAACTCAACAAGCTGATCAAGAGCAACACCGTGGATCCCGATCTGCCGGACAGAAGG
Cele.WS201 Cele.WS201_Fam_199_57_1 1 57 0.000000 AAATTTCTAAGACTGTACCATTCTCTCCATTCAAAACCTCATGTCAAACTCTCAATC
Cele.WS201 Cele.WS201_Fam_200_56_1 1 56 0.000000 GAAATTAAAATTTGTGTTTTTGGCATTTTCTAGGCCATTTGGATTAGGTGGCCTAG
Cele.WS201 Cele.WS201_Fam_201_52_1 1 52 0.000000 AAAATCGATAATTTTCGTGTGATTTTACTCTAAAAATTCGGATTTTTCGCTT
Cele.WS201 Cele.WS201_Fam_202_52_1 1 52 0.000000 TCCGACACATCGGAACTTGCCGGCGACACGAAAAATTGTGTCGGCGGTGAAT
Cele.WS201 Cele.WS201_Fam_203_51_1 1 51 0.000000 CAAATCGGCAGACCGGCAATTTGCCGAAATTGAAAATTCCCGGAAAATCGG
Cele.WS201 Cele.WS201_Fam_204_50_1 1 50 0.000000 TGGCCTAGAACCCCGTTTTTCTGTAGCCTAGACTATCATCTAGAAATCAA
Cele.WS201 Cele.WS201_Fam_205_50_1 1 50 0.000000 GGGCGTGTTTTCACATGCCCCGCCCACAAAATTGATGTTTCCACCAAAAT
Cele.WS201 Cele.WS201_Fam_206_49_1 1 49 0.000000 AAAATCAATAATTTTAAATTAGATTATCAATTTTTCTAAATTTTTCCGA
Cele.WS201 Cele.WS201_Fam_207_49_1 1 49 0.000000 GCTACGATATCGTAGCACTTTTTTATTTTTCCATATTCCACCAGAAAAT
Cele.WS201 Cele.WS201_Fam_208_48_1 1 48 0.000000 TGCCTACGTGCCTACATAAGAAGCGTAGGTAGCTTCAAAGGTGTTGCG
Cele.WS201 Cele.WS201_Fam_209_45_1 1 45 0.000000 ACCATCAGGCGGACCACCAGGACCATTTGATCCATCAGGAGCACC
Cele.WS201 Cele.WS201_Fam_210_45_1 1 45 0.000000 ACAACTGAATCAACTGCAGCACCAAGCACATCTACAGAGACACCT
Cele.WS201 Cele.WS201_Fam_211_45_1 1 45 0.000000 CCACCAGCACAGCTTCGAGTACGACAGAGAGCACTTCGACTCCAT
Cele.WS201 Cele.WS201_Fam_212_43_1 1 43 0.000000 AGCAGCCGACAATTGCCGGTTGCCTTAAAATTGAAGTAAATCC
Cele.WS201 Cele.WS201_Fam_213_43_1 1 43 0.000000 CGACAATTTTCGAAGATTTTTTGGTGAAAATTTGGTCATTTTC
Cele.WS201 Cele.WS201_Fam_214_42_1 1 42 0.000000 GGACCAAATGGACCAAATGGGCCATCAGATCCGAATAAGCCA
Cele.WS201 Cele.WS201_Fam_215_42_1 1 42 0.000000 CCTAGAATTTTTGGCATGGTGGCCTTGATTTTAACAAGACGG
Cele.WS201 Cele.WS201_Fam_216_42_1 1 42 0.000000 CGGCAAATCGGCACGTTGCCAAATTGCCGTTTTTAAAATATC

Cele.WS201	Cele.WS201_Fam_217_42_1	1	42	0.000000	AGGTGCTGCTTCCTTCGGAACATTTTCTTGAGTTGGCTCCGA
Cele.WS201	Cele.WS201_Fam_218_40_1	1	40	0.000000	AATTGATTTGTTTAGACTGAAATAAATGTTTTTTTGGAAA
Cele.WS201	Cele.WS201_Fam_219_40_1	1	40	0.000000	GGAATTTTTGATTTTCCGGCTATGAAAACCGAAACAAATC
Cele.WS201	Cele.WS201_Fam_220_40_1	1	40	0.000000	AAAATCCACGTAGATTATCGATTTTTCATAGAATTTTGCA
Cele.WS201	Cele.WS201_Fam_221_40_1	1	40	0.000000	CCAATTTTTTGGGCAAAAAATCAAAAATTCTGAATTTTGG
Cele.WS201	Cele.WS201_Fam_222_40_1	1	40	0.000000	AATGGGCGCCGGTTTCGCTTTGAGCGATTCCTTACCCTTA
Cele.WS201	Cele.WS201_Fam_223_39_1	1	39	0.000000	GAAGAGACCACCACCGCAGCTGCCACCGAAGCCTCGTCG
Cele.WS201	Cele.WS201_Fam_224_39_1	1	39	0.000000	TTTAGTCCCAGTGATATATCTGACACACTTTAGGCCCCG
Cele.WS201	Cele.WS201_Fam_225_39_1	1	39	0.000000	GTAGTCGGAGTAGTCGGAGTAGTTGGAGTAGACTTCATG
Cele.WS201	Cele.WS201_Fam_226_39_1	1	39	0.000000	TGGTACGGTAGTTGTAGTGGTAGTGGAGGTTGTTGTAGG
Cele.WS201	Cele.WS201_Fam_227_39_1	1	39	0.000000	ATTGATACTTTGTCGGGTTTAGATCATCGGCCGAGCTTC
Cele.WS201	Cele.WS201_Fam_228_38_1	1	38	0.000000	TAGGCCATCAGTGAAAACCTATGTGCTCGGTGAAAAGT
Cele.WS201	Cele.WS201_Fam_229_37_1	1	37	0.000000	CAAATTGGCAAATTGGCAAAAAATGTTAAATTCTCGC
Cele.WS201	Cele.WS201_Fam_230_36_1	1	36	0.000000	GCAGGAAATTTAAAAAATGTTGAACAACATTTTTTG
Cele.WS201	Cele.WS201_Fam_231_36_1	1	36	0.000000	GATATTTTCATGTTCGGCAAAAGTACCGATTTGCCA
Cele.WS201	Cele.WS201_Fam_232_36_1	1	36	0.000000	CCTTTGTCACCCTTTGATCCAGGAGTTCCATCGGCC
Cele.WS201	Cele.WS201_Fam_233_36_1	1	36	0.000000	TCCTGGTGGTCCTGGTGGTCCAGCTTGTCCATCGAC
Cele.WS201	Cele.WS201_Fam_234_35_1	1	35	0.000000	TTTGCCGGAAATGTTGAACTCTGAAAATTTTCGGG
Cele.WS201	Cele.WS201_Fam_235_35_1	1	35	0.000000	CGGCAAATTGCCGATTTTCCGAATTTCCCGGAAAT
Cele.WS201	Cele.WS201_Fam_236_35_1	1	35	0.000000	GCCGGCGGCTGCGAGACATGTTTAGCGACGTGTGC
Cele.WS201	Cele.WS201_Fam_237_34_1	1	34	0.000000	CTAAGCCTAAATAGCGAACGCTCGCCACTGACGC
Cele.WS201	Cele.WS201_Fam_238_34_1	1	34	0.000000	GAAAATTCAACTGTTTTGGGGAAATTTTAACCGG
Cele.WS201	Cele.WS201_Fam_239_34_1	1	34	0.000000	CGGCAAATTTCCCAAGCTCGGCAAACCACCAATT
Cele.WS201	Cele.WS201_Fam_240_34_1	1	34	0.000000	AAGTATTTTTGCCGGTTTTTTTGCCGAAATTTCC
Cele.WS201	Cele.WS201_Fam_241_33_1	1	33	0.000000	TTTTAAAATTCGGCAAAGTTAGCAAATTGCCGG
Cele.WS201	Cele.WS201_Fam_242_33_1	1	33	0.000000	ATCCTCCTTGGGATCCTCCAGCATATCCACCGG
Cele.WS201	Cele.WS201_Fam_243_33_1	1	33	0.000000	CTAAGGACAAAATGGGAGACGCCTGGGACACCA
Cele.WS201	Cele.WS201_Fam_244_32_1	1	32	0.000000	CAGAAGCTGTCGAAAACTCAAACTTTCCTCGA
Cele.WS201	Cele.WS201_Fam_245_32_1	1	32	0.000000	AACTCGGCCACCAATATTCTCAAACTTAAGAA
Cele.WS201	Cele.WS201_Fam_246_32_1	1	32	0.000000	GCACCAAATATCACCAATTTAGCACCGATAGA
Cele.WS201	Cele.WS201_Fam_247_32_1	1	32	0.000000	CCAACAGTACCCCCTTAGGACCCCTTAGGACC
Cele.WS201	Cele.WS201_Fam_248_32_1	1	32	0.000000	GGCACCGCCCACCTCTCGCATTTTCGAGAATA
Cele.WS201	Cele.WS201_Fam_249_32_1	1	32	0.000000	AAATTTATGAAAATTTAGGCCCATTTTGCCCA
Cele.WS201	Cele.WS201_Fam_250_32_1	1	32	0.000000	TCTGGACTGGTGTAGTAGAGCTGCTTGGAGGT
Cele.WS201	Cele.WS201_Fam_251_31_1	1	31	0.000000	CTGAAATTCCAATTTTCCACAGTAAAAATGC
Cele.WS201	Cele.WS201_Fam_252_31_1	1	31	0.000000	GTGACGTCACTTTTTTTGCCGAAAACCAAGT
Cele.WS201	Cele.WS201_Fam_253_31_1	1	31	0.000000	GCTGATTGGTCGGCATGGTCTGCTCATCATC
Cele.WS201	Cele.WS201_Fam_254_31_1	1	31	0.000000	TGCGTCGGAAATGCATTCTTATCATCAGCGG
Cele.WS201	Cele.WS201_Fam_255_31_1	1	31	0.000000	TCCGATGCATTGCCGATAATTGCCACCTGGT
Cele.WS201	Cele.WS201_Fam_256_31_1	1	31	0.000000	TCTGAGCTACAGTACTCCTGTCAAGAACAAT
Cele.WS201	Cele.WS201_Fam_257_31_1	1	31	0.000000	GCAGCCGACAGTTCACTAGTCGAAACTACAA
Cele.WS201	Cele.WS201_Fam_258_31_1	1	31	0.000000	AGTCATTTTTGGCCAATTTTTGACTATTTTC
Cele.WS201	Cele.WS201_Fam_259_31_1	1	31	0.000000	TTGGCCCAGAGCTCTCTTGACCTAAGCCCCC
Cele.WS201	Cele.WS201_Fam_260_30_1	1	30	0.000000	AATTTGATTATTTCCGAATTTTTTAGCGAA
Cele.WS201	Cele.WS201_Fam_261_30_1	1	30	0.000000	TGGTGGAACTGGATTCGGCAGTGGAAATGC
Cele.WS201	Cele.WS201_Fam_262_30_1	1	30	0.000000	GAAAAATTGATCTACAGCGAGATTTGGAGT
Cele.WS201	Cele.WS201_Fam_263_30_1	1	30	0.000000	CAGCCCCAGCTCCGGCCGCCGAAGAGACCC
Cele.WS201	Cele.WS201_Fam_264_30_1	1	30	0.000000	CAAACTACAGTAGCACTGTAGTACCATAAC
Cele.WS201	Cele.WS201_Fam_265_30_1	1	30	0.000000	TATGAAAGATCCTTCGATCAAGAAGTCACG
Cele.WS201	Cele.WS201_Fam_266_30_1	1	30	0.000000	AAAATCAAAAATTAGATGAATTTTCGAATA
Cele.WS201	Cele.WS201_Fam_267_30_1	1	30	0.000000	TTTTTCAACTTTTTCAGTTGAAGATTCCAT
Cele.WS201	Cele.WS201_Fam_268_30_1	1	30	0.000000	ATCTACTTTTTCAGATTCCGTCGAAACTTG
Cele.WS201	Cele.WS201_Fam_269_30_1	1	30	0.000000	ACTACCCAACCGGCTCCGGCAACTAGTCCG
Cele.WS201	Cele.WS201_Fam_270_29_1	1	29	0.000000	AAAAATCGATTTTTTCAAAAATTTTCCAG
Cele.WS201	Cele.WS201_Fam_271_27_1	1	27	0.000000	GGCCAAATCTGGTGTTCAAATTTTTTA
Cele.WS201	Cele.WS201_Fam_272_27_1	1	27	0.000000	GCTGCTGGAGGATCAACTTCAACAACT
Cele.WS201	Cele.WS201_Fam_273_27_1	1	27	0.000000	TATTATAAATATAATATTACCTATTAT

Cele.WS201	Cele.WS201_Fam_274_26_1	1	26	0.000000	AAAATTAATAGATTTTCCAAACTTTG
Cele.WS201	Cele.WS201_Fam_275_26_1	1	26	0.000000	ATCTACAGTACCCCTATTCTAGAGTG
Cele.WS201	Cele.WS201_Fam_276_26_1	1	26	0.000000	CGTAGTAATAAATCCAACCTGCCATT
Cele.WS201	Cele.WS201_Fam_277_26_1	1	26	0.000000	ATTTACGGAGCTTCAAGTTAATGTTG
Cele.WS201	Cele.WS201_Fam_278_26_1	1	26	0.000000	TGTCAGTGCAAAATGTTTTATTATTT
Cele.WS201	Cele.WS201_Fam_279_26_1	1	26	0.000000	AAGTTCCAAAATTCTAAAACATCCAA
Cele.WS201	Cele.WS201_Fam_280_24_1	1	24	0.000000	AAGTAGGTCATGACCTAATTTTTT
Cele.WS201	Cele.WS201_Fam_281_24_1	1	24	0.000000	TTTAATATACGCTATACATAAGTA
Cele.WS201	Cele.WS201_Fam_282_24_1	1	24	0.000000	GTGCTCCGTGAGCATTGGGAATTT
Cele.WS201	Cele.WS201_Fam_283_24_1	1	24	0.000000	AAAAACCGGCAAAAAAATCTTTTG
Cele.WS201	Cele.WS201_Fam_284_24_1	1	24	0.000000	GAGTCTACCACAGAATCAACTACG
Cele.WS201	Cele.WS201_Fam_285_24_1	1	24	0.000000	TTTTTTGGAAAAAAATATATTGAT
Cele.WS201	Cele.WS201_Fam_286_24_1	1	24	0.000000	TTTGAGTTTATCATCCTTATCTTT
Cele.WS201	Cele.WS201_Fam_287_23_1	1	23	0.000000	GGACTAGAAATACTGAACGTGGT
Cele.WS201	Cele.WS201_Fam_288_23_1	1	23	0.000000	TGGCCTAACTTTTCCTTATTTGG
Cele.WS201	Cele.WS201_Fam_289_23_1	1	23	0.000000	CGCTGTAAGACCATTTGCTGTAT
Cele.WS201	Cele.WS201_Fam_290_22_1	1	22	0.000000	GCATGGCCTGACGGTCCTGAGG
Cele.WS201	Cele.WS201_Fam_291_22_1	1	22	0.000000	ACTACAGTAATTCTACGTCGCT
Cele.WS201	Cele.WS201_Fam_292_22_1	1	22	0.000000	TTTAAGCCTAGCAGGGGTCCAA
Cele.WS201	Cele.WS201_Fam_293_22_1	1	22	0.000000	CCTTCCAAGAGCCTCACATGAT
Cele.WS201	Cele.WS201_Fam_294_22_1	1	22	0.000000	TCTGGGGTAGGGCTGCCTAGGA
Cele.WS201	Cele.WS201_Fam_295_22_1	1	22	0.000000	AATCTTAAAATGCACATTTTTT
Cele.WS201	Cele.WS201_Fam_296_21_1	1	21	0.000000	TGGCCTCGATTCTGTACAGGG
Cele.WS201	Cele.WS201_Fam_297_21_1	1	21	0.000000	TCTCCTGGTAAATTTAAAAAT
Cele.WS201	Cele.WS201_Fam_298_21_1	1	21	0.000000	CAAACCTTTTTGGCAGATTTT
Cele.WS201	Cele.WS201_Fam_299_21_1	1	21	0.000000	ACCTGGCCAGATCGGTAGGCA
Cele.WS201	Cele.WS201_Fam_300_21_1	1	21	0.000000	GCATAAAAACGTAAATTTTAG
Cele.WS201	Cele.WS201_Fam_301_21_1	1	21	0.000000	GCAATTTTCCCGTGCAGATCG
Cele.WS201	Cele.WS201_Fam_302_21_1	1	21	0.000000	GCAGCGCCGGTCTCATCGAGC
Cele.WS201	Cele.WS201_Fam_303_21_1	1	21	0.000000	AAAATAGCTTAAAATTGATTA
Cele.WS201	Cele.WS201_Fam_304_21_1	1	21	0.000000	CTAGATTTTTTTGGCGTTGAC
Cele.WS201	Cele.WS201_Fam_305_21_1	1	21	0.000000	CTAACATTTTTCAATTTTCGT
Cele.WS201	Cele.WS201_Fam_306_21_1	1	21	0.000000	CTAATTAGCGTCGCTTTCGTT
Cele.WS201	Cele.WS201_Fam_307_21_1	1	21	0.000000	AAAAGCCTAAAAAACCCCAAA
Cele.WS201	Cele.WS201_Fam_308_21_1	1	21	0.000000	GATCTTCCAGCTACACCGTCT
Cele.WS201	Cele.WS201_Fam_309_21_1	1	21	0.000000	TCAAATTAGATTACCTTTTTT
Cele.WS201	Cele.WS201_Fam_310_21_1	1	21	0.000000	TTTGCACTGACCAACACGAGT
Cele.WS201	Cele.WS201_Fam_311_21_1	1	21	0.000000	TGAAAGATTGGTCAGTGTCTA
Cele.WS201	Cele.WS201_Fam_312_20_1	1	20	0.000000	TTCTAGGCCGTAGTAAACAT
Cele.WS201	Cele.WS201_Fam_313_20_1	1	20	0.000000	TGGCAAAAATGGCAGAAAAA
Cele.WS201	Cele.WS201_Fam_314_20_1	1	20	0.000000	TAAATCTACACAGGGTGGTG
Cele.WS201	Cele.WS201_Fam_315_20_1	1	20	0.000000	TGTCGATTTACGGCGATCGA
Cele.WS201	Cele.WS201_Fam_316_20_1	1	20	0.000000	TATTGATTTTTACCGGATGT
Cele.WS201	Cele.WS201_Fam_317_20_1	1	20	0.000000	GGAAATTTTCCCGATTAGCC
Cele.WS201	Cele.WS201_Fam_318_20_1	1	20	0.000000	GCTCGAGCTCAAACCCCGAC
Cele.WS201	Cele.WS201_Fam_319_20_1	1	20	0.000000	AAGGTTCTAGTAAATTCCAG
Cele.WS201	Cele.WS201_Fam_320_20_1	1	20	0.000000	TTCTGGAATTTTTCCTGAGC
Cele.WS201	Cele.WS201_Fam_321_19_1	1	19	0.000000	AAAATTCTGAAAACTCGAG
Cele.WS201	Cele.WS201_Fam_322_19_1	1	19	0.000000	TGGTCAGTGCAACTGCAAC
Cele.WS201	Cele.WS201_Fam_323_18_1	1	18	0.000000	TTGTAGATCAACGGGGTC
Cele.WS201	Cele.WS201_Fam_324_18_1	1	18	0.000000	GGCCTAGGATCCATCACT
Cele.WS201	Cele.WS201_Fam_325_18_1	1	18	0.000000	TGCTCAAAGTGCTCGAGG
Cele.WS201	Cele.WS201_Fam_326_18_1	1	18	0.000000	ATAGTTAGGCTACCTGTA
Cele.WS201	Cele.WS201_Fam_327_18_1	1	18	0.000000	ACTACTGTATCGCAAGAT
Cele.WS201	Cele.WS201_Fam_328_18_1	1	18	0.000000	ATATAATGTATATAATGC
Cele.WS201	Cele.WS201_Fam_329_18_1	1	18	0.000000	CATTGTCACGTTCTTCTC
Cele.WS201	Cele.WS201_Fam_330_18_1	1	18	0.000000	TTTTGTCAGTTCAATAGT

Cele.WS201	Cele.WS201_Fam_331_17_1	1	17	0.000000	CGGCGGAAAAATCTACT
Cele.WS201	Cele.WS201_Fam_332_17_1	1	17	0.000000	AAAATCAAACCGGAGAC
Cele.WS201	Cele.WS201_Fam_333_17_1	1	17	0.000000	TAACCTGACTGCTATAT
Cele.WS201	Cele.WS201_Fam_334_17_1	1	17	0.000000	GAATTAGAAAACTTTGA
Cele.WS201	Cele.WS201_Fam_335_17_1	1	17	0.000000	TTGCAAGAAAATCTGCA
Cele.WS201	Cele.WS201_Fam_336_17_1	1	17	0.000000	TAGTTTTGCACCTCTAA
Cele.WS201	Cele.WS201_Fam_337_16_1	1	16	0.000000	TTTGGAATGTCGGAAC
Cele.WS201	Cele.WS201_Fam_338_16_1	1	16	0.000000	TGATCTGATGATCTCA
Cele.WS201	Cele.WS201_Fam_339_16_1	1	16	0.000000	GTACTGTAGGGAGAGG
Cele.WS201	Cele.WS201_Fam_340_16_1	1	16	0.000000	CGGCAAATCGGCAGAC
Cele.WS201	Cele.WS201_Fam_341_16_1	1	16	0.000000	ACCGCAACCCTACCCT
Cele.WS201	Cele.WS201_Fam_342_16_1	1	16	0.000000	CTCCTAGGCTCCTAGG
Cele.WS201	Cele.WS201_Fam_343_16_1	1	16	0.000000	CTTAGATCTCAATTCA
Cele.WS201	Cele.WS201_Fam_344_16_1	1	16	0.000000	GGACCAAAGGGCCAAA
Cele.WS201	Cele.WS201_Fam_345_15_1	1	15	0.000000	CGATGCTCCACCCGG
Cele.WS201	Cele.WS201_Fam_346_15_1	1	15	0.000000	GAACCACCAAAGATT
Cele.WS201	Cele.WS201_Fam_347_15_1	1	15	0.000000	AAAAGGGGATCTGCG
Cele.WS201	Cele.WS201_Fam_348_14_1	1	14	0.000000	GAAGGATCGGGCCG
Cele.WS201	Cele.WS201_Fam_349_14_1	1	14	0.000000	GGACTAAGGACTAG
Cele.WS201	Cele.WS201_Fam_350_13_1	1	13	0.000000	GCTGCCGCCCGCC
Cele.WS201	Cele.WS201_Fam_351_13_1	1	13	0.000000	CGTAGGCCATATA
Cele.WS201	Cele.WS201_Fam_352_12_1	1	12	0.000000	ACCAGGACCAGG
Cele.WS201	Cele.WS201_Fam_353_12_1	1	12	0.000000	AGTTATATAGTA
Cele.WS201	Cele.WS201_Fam_354_12_1	1	12	0.000000	AGAAAGAGGAGA
Cele.WS201	Cele.WS201_Fam_355_12_1	1	12	0.000000	CCTCCTCCACAT
Cele.WS201	Cele.WS201_Fam_356_12_1	1	12	0.000000	GCTGGAGCTGGA
Cele.WS201	Cele.WS201_Fam_357_12_1	1	12	0.000000	CATTGACTTGAG
Cele.WS201	Cele.WS201_Fam_358_11_1	1	11	0.000000	GCTCTAAAAGT
Cele.WS201	Cele.WS201_Fam_359_11_1	1	11	0.000000	CGGATAATTAA
Cele.WS201	Cele.WS201_Fam_360_11_1	1	11	0.000000	AGATACACTTC
Cele.WS201	Cele.WS201_Fam_361_11_1	1	11	0.000000	GTTGCGATTGC
Cele.WS201	Cele.WS201_Fam_362_11_1	1	11	0.000000	ATCGGTGCTAC
Cele.WS201	Cele.WS201_Fam_363_10_1	1	10	0.000000	TGGGGAAAAA
Cele.WS201	Cele.WS201_Fam_364_10_1	1	10	0.000000	TATCTATATC

C. briggsae

Genome	Family name	Number of sequences	Alignment length	Score	Consensus without gaps
Cbrig.WS247	Cbrig.WS247_Fam_1_16_1253	1253	33	0.401617	AAATTCAgAAATTCaG
Cbrig.WS247	Cbrig.WS247_Fam_2_16_557	557	16	0.854772	ATTCTgAGATTCTGAG
Cbrig.WS247	Cbrig.WS247_Fam_3_22_70	70	29	0.566840	GACCTAgaAAAAAtTAGGTCAt
Cbrig.WS247	Cbrig.WS247_Fam_4_15_56	56	19	0.531203	CgGAATcCngGAATC
Cbrig.WS247	Cbrig.WS247_Fam_5_21_47	47	25	0.595954	CATTTTTCATGatTTTTggnc
Cbrig.WS247	Cbrig.WS247_Fam_6_14_46	46	17	0.690386	GGTCCTGGGTCCTG
Cbrig.WS247	Cbrig.WS247_Fam_7_16_40	40	17	0.680581	GTTCTaAAAtGTCGcg
Cbrig.WS247	Cbrig.WS247_Fam_8_20_31	31	22	0.753014	AGCCTCAGTAAAAAtTCCTA
Cbrig.WS247	Cbrig.WS247_Fam_9_15_31	31	17	0.717521	CGGATCcAGGTCACC
Cbrig.WS247	Cbrig.WS247_Fam_10_14_28	28	14	0.870213	TCCTTaTTCCTTAT
Cbrig.WS247	Cbrig.WS247_Fam_11_20_22	22	20	0.780087	CTGGAATTTTCTAGAAtgTT
Cbrig.WS247	Cbrig.WS247_Fam_12_10_19	19	10	0.887719	AAaTTCcAGA
Cbrig.WS247	Cbrig.WS247_Fam_13_21_16	16	22	0.708712	GATTCCACCTccACCAGCTCc
Cbrig.WS247	Cbrig.WS247_Fam_14_15_16	16	16	0.673090	aAaCCaGaCAtTTCG
Cbrig.WS247	Cbrig.WS247_Fam_15_163_15	15	165	0.714613	GAaAAATtcGaAAAAtCgGtTTTtGAgCCCcAAAATCgaaATTTTtGCTTCcAACTTTTTTCTgAatgAaCgAaTTCACgCAaATctTTtTGTAAAAnAttAGtCAcnaTnATagTttTGTTcTAAnattTGcTTGTtCTGtCTTTTCaCAAAAcTcGtaTGT
Cbrig.WS247	Cbrig.WS247_Fam_16_19_14	14	19	0.971467	AAATTCTGGTCCTTAAAAA
Cbrig.WS247	Cbrig.WS247_Fam_17_29_12	12	31	0.643043	TGACCTAatTTTTnGnAtTTTCTAGGtCA
Cbrig.WS247	Cbrig.WS247_Fam_18_26_12	12	26	0.746698	CCTAGATTTcaATTTTtAGGTCATGA
Cbrig.WS247	Cbrig.WS247_Fam_19_14_12	12	18	0.641274	TGgAATCTGgAATC
Cbrig.WS247	Cbrig.WS247_Fam_20_14_12	12	17	0.605912	CAGCTCCAGagCTC
Cbrig.WS247	Cbrig.WS247_Fam_21_16_12	12	16	0.727273	GATCcAGGGATCcAgG
Cbrig.WS247	Cbrig.WS247_Fam_22_13_11	11	18	0.506229	GGCCCAgGCCCnn
Cbrig.WS247	Cbrig.WS247_Fam_23_81_10	10	81	0.965798	TGAGAGGATTGTTGTGAAAATTAATAAAGAcCCTTtTTCGTTGTTTTTGCATcAATTCTCCCCAGAGTTTGAAGGAACCAG
Cbrig.WS247	Cbrig.WS247_Fam_24_21_10	10	26	0.428917	aaGCanaTTTaGGcCnATTTt
Cbrig.WS247	Cbrig.WS247_Fam_25_13_9	9	13	0.692308	ctgACGGaCggAC
Cbrig.WS247	Cbrig.WS247_Fam_26_182_7	7	184	0.914941	CATGTCATAATTGTGCTAGAAAGATGCTTTTTAACCACCATGTTGAAAGATTCACTGAAGaGCGATGTCTAGAATaGTCCTCATTTTGAGCGTTTTTTAGGTCAAAATTACCGATTTTCTGTGAATTTTTTCAaGTATTCAACGGAAAgATgGCCGTGTCACTaTATTtTAAcCAGATTAAg
Cbrig.WS247	Cbrig.WS247_Fam_27_16_7	7	16	0.781746	tAgTTGCGAAATGTCA
Cbrig.WS247	Cbrig.WS247_Fam_28_15_7	7	15	0.932275	AAGATTTTCTTTAgG
Cbrig.WS247	Cbrig.WS247_Fam_29_30_6	6	30	0.715556	GGTGaTAGTTGAAgcTGgAGtAGGTTCAGA
Cbrig.WS247	Cbrig.WS247_Fam_30_19_6	6	19	0.700585	TGaCGCATTTTcaGtGCcT
Cbrig.WS247	Cbrig.WS247_Fam_31_16_5	5	16	0.783333	AAGcCCggAAGcCCgg
Cbrig.WS247	Cbrig.WS247_Fam_32_14_5	5	15	0.740000	aCTTCcGACTTCcG
Cbrig.WS247	Cbrig.WS247_Fam_33_11_5	5	12	0.705556	CTAtTTTAaGc
Cbrig.WS247	Cbrig.WS247_Fam_34_11_5	5	12	0.800000	tTACCgGTAAT
Cbrig.WS247	Cbrig.WS247_Fam_35_146_4	4	146	0.969559	TCATATTTCCACCCGTAAGGTTTGGATGCAAAcTTCGTTGTTTTTCTCCGGTATCGGGTTGGAAAAACTATGAGGGGTCTCAATCcTGTGTAcAGACTAcGAACCTTTGCTGTCGGCGTCTcAGTTGTATTGATCCGGCaGTACT
Cbrig.WS247	Cbrig.WS247_Fam_36_60_4	4	60	0.922222	CaACATCaTCAGCTGAGACCTCGACAATcGCCACCTCGACCGCTGAGACtActACAGTTC
Cbrig.WS247	Cbrig.WS247_Fam_37_46_4	4	47	0.686761	TTTCTaGGtCATtTTgAAgTggTGgCCTAAcTTTtgCaAaTTtggT
Cbrig.WS247	Cbrig.WS247_Fam_38_27_4	4	30	0.749074	ATAGaTACCATAGaTACCATAGaTACC
Cbrig.WS247	Cbrig.WS247_Fam_39_19_4	4	23	0.681159	tAAGCCTAAGCCTaAGgGt
Cbrig.WS247	Cbrig.WS247_Fam_40_22_4	4	22	0.654040	aAAaTtAgCcGAAAATcaTgAA
Cbrig.WS247	Cbrig.WS247_Fam_41_21_4	4	21	0.735450	ATTTTtgACccAAAATtagTC
Cbrig.WS247	Cbrig.WS247_Fam_42_21_4	4	21	0.904762	ATTTACCgTAATTccATTTTG
Cbrig.WS247	Cbrig.WS247_Fam_43_21_4	4	21	0.820106	TAAGCcTATGCCAcAAaaAGc
Cbrig.WS247	Cbrig.WS247_Fam_44_16_4	4	18	0.660494	GATCcaAGATCCtAaG
Cbrig.WS247	Cbrig.WS247_Fam_45_16_4	4	16	0.743056	CTGGGtttCTGaaTCT

Cbrig.WS247 Cbrig.WS247_Fam_46_254_3 3 255 0.939869
TTTTTTTGATTTTTTGTCaTAGAAACGTTcTACATTCAATTTCAAGTTACGTCCCAAAATTTCAGGCCCAAATCTCTTATTTGAACAAAGTTATTAAGGATTgTTCTAAGCaTgTACTTAAGCCAAAATTCGGTTTcAGGCTTTtGTCCATAACTTCGTA
TCGAGAAGAGATCTCGATCTGATATtcGAAttcGGTACCTaAAtTTGAATGTaAAACGTTTTCaTAACAAAAAATCTCAAAAAATTGACAAACA
Cbrig.WS247 Cbrig.WS247_Fam_47_167_3 3 167 0.946773
TGACAGATTTTTGAtAGCcCttACAGaAACCGAGATaTATGGAACAGACGGCCGTCTTCCGTCACACAGAGAAACTTTGAGCTGAATTTGAGgGGTTCTtATGCGAGTTAGCAAAAATACGTCAACTGAAgAAATGTTAACAACACTTGTGCTAAGATTT
TATtAGT
Cbrig.WS247 Cbrig.WS247_Fam_48_154_3 3 154 0.919192
AAAATgtTTGAAgTCAGTAGGGGAGCATTTTATtAGTTgACAAAACATTTTTaTcTtTCACAGGAACGGAGATAAtTGaACTTGAAAAAAcCGAAATATTCAAGTTcATTTATTCcCTATTCTGGTGAAAGTTATCAATAAATAGTCAAcAAGT
Cbrig.WS247 Cbrig.WS247_Fam_49_139_3 3 139 0.968026
ATTTCTCAATTTCGAACTGATTTTCTATAAAACaGCCCAATTTTTCTGTTCCAAACTTATCCGAAATcCAAATTTCCTGTATATCgTAgCTGATGAGCGGAAATTTGGGCAGAATTgAAAATTAGAAAACTGTGTTGTG
Cbrig.WS247 Cbrig.WS247_Fam_50_111_3 3 111 0.855856
GAAgACTCGCCaACaTAgTTttggAaCTTgCaCGTTCcAAtATTtgCCATTTTGGTCAGAAATTGGCTCAGAAATCGGCTGGAGCAATGTgTCAaCTGGGTgTAACAGAAG
Cbrig.WS247 Cbrig.WS247_Fam_51_111_3 3 111 0.975976
AAATGGAAAAGAAGACTTGCTGAAAGGACCATTGAgCTTTTaGGCCCAAAAATTGTCCGATTTTCCTCAAAATTAAACtTTAAGATTACGGCAGCAGTGTGTCAATGGAGA
Cbrig.WS247 Cbrig.WS247_Fam_52_58_3 3 59 0.905838 AAAATtaAAAAATCGCATTCCAATTTTCAAATTTCTGAATTTCtCTtCtAAAAATCAT
Cbrig.WS247 Cbrig.WS247_Fam_53_47_3 3 50 0.593333 tTACTGTAGTTTTngGATtCanTtTTCaaAnTTGAAAAtAntGAGGa
Cbrig.WS247 Cbrig.WS247_Fam_54_42_3 3 42 0.957672 GACTCGGTCGAtGTGGAGGTGGATTCAGTCGAGGTACTcGTG
Cbrig.WS247 Cbrig.WS247_Fam_55_31_3 3 33 0.764310 GnGACACTTcGAAgCCaGTCAtTTCGAAACC
Cbrig.WS247 Cbrig.WS247_Fam_56_30_3 3 30 0.881481 ACTGGAGCAGCcTCGACTGGTTTCTcTTca
Cbrig.WS247 Cbrig.WS247_Fam_57_24_3 3 28 0.603175 TTTAgaATTTCAGaATTTCtGAaT
Cbrig.WS247 Cbrig.WS247_Fam_58_27_3 3 27 0.967078 AACCCCATGAACCCCATGAACCCCATt
Cbrig.WS247 Cbrig.WS247_Fam_59_24_3 3 26 0.752137 AATTCcCGCCaAAATatTTTTTTG
Cbrig.WS247 Cbrig.WS247_Fam_60_26_3 3 26 0.743590 AGTAcTCtTtGAaaTTCtgAAACTAC
Cbrig.WS247 Cbrig.WS247_Fam_61_21_3 3 24 0.620370 AgtTGAAnGGATTACTGTAGn
Cbrig.WS247 Cbrig.WS247_Fam_62_24_3 3 24 0.703704 TCCTTCTTGGccTTntCnTCaGCt
Cbrig.WS247 Cbrig.WS247_Fam_63_21_3 3 23 0.550725 TTTtaAGTcgtTTTnGACTAt
Cbrig.WS247 Cbrig.WS247_Fam_64_21_3 3 22 0.601010 AgGcCATTTcAaaCCnATTTc
Cbrig.WS247 Cbrig.WS247_Fam_65_21_3 3 22 0.702020 TCCgAAaTCAGtcAAAAATgG
Cbrig.WS247 Cbrig.WS247_Fam_66_21_3 3 21 0.798942 TTTgTAGccCATTTTCAGTca
Cbrig.WS247 Cbrig.WS247_Fam_67_19_3 3 20 0.694444 GTTTtAGAaCTTTtggGAC
Cbrig.WS247 Cbrig.WS247_Fam_68_19_3 3 19 0.883041 TAGAGGTCCcaAAAAAAAT
Cbrig.WS247 Cbrig.WS247_Fam_69_16_3 3 18 0.648148 GAcCCATAGAccCATg
Cbrig.WS247 Cbrig.WS247_Fam_70_16_3 3 17 0.718954 ACCTAGTTaCCgTAtA
Cbrig.WS247 Cbrig.WS247_Fam_71_17_3 3 17 0.790850 TCTCTTttACTACcaAG
Cbrig.WS247 Cbrig.WS247_Fam_72_14_3 3 14 0.746032 TAtAATaTAtAATa
Cbrig.WS247 Cbrig.WS247_Fam_73_10_3 3 11 0.767677 CTTCAGaATC
Cbrig.WS247 Cbrig.WS247_Fam_74_11_3 3 11 0.757576 CtTGcCCTGtT
Cbrig.WS247 Cbrig.WS247_Fam_75_10_3 3 10 0.733333 AGcCtAGCtT
Cbrig.WS247 Cbrig.WS247_Fam_76_127_2 2 127 0.968504
AACTTTTCACACCCCATTTTcCACTTTTTAATTTTTCCCTTTCAATTTTTCATCCTaATTTTTTCAATTTCATTTTTTTGTcCTACATTTTTCATTTCCAAATTTTTGTGCATATTTTTTCATTCCT
Cbrig.WS247 Cbrig.WS247_Fam_77_122_2 2 122 0.945355
CATCAAATCACTaAAAATAGACAGCAAGAATTACTGTAGCCGAACAATTTCGATCTaATGGGCTCTGTAGTCGCTTCAGcTGACGCACTAGCCTAAAAaGTAAATTTTGAGCTCGTCAGAGc
Cbrig.WS247 Cbrig.WS247_Fam_78_83_2 2 83 1.000000
CTCCGCCCACTTTTCAGAAAGTGGGCGTGGTCTTGAAAATTCGAGATTGGGCGAAGCTTAAAACTAGCTCAAACTCGAATAGG
Cbrig.WS247 Cbrig.WS247_Fam_79_64_2 2 64 0.830729 TTTGGAATTTTaAGAGAACACATGACGCCTGAGAATGGCTAAATTaTTGAGATTGAGtttggaa
Cbrig.WS247 Cbrig.WS247_Fam_80_53_2 2 53 0.685535 ACGACAAAATGCAcaAAAaTTTaaATTTTTccGaAATTTTGCATaCAgATTTG
Cbrig.WS247 Cbrig.WS247_Fam_81_48_2 2 48 1.000000 TTTTAGAAATTTTTGGTTCTGAAATGTCCTATTTTTAAAAATGTGACG
Cbrig.WS247 Cbrig.WS247_Fam_82_48_2 2 48 0.888889 TCGTGCTCGAcGATGGTACTTCacTAGTTGAaCTGGATGGTTCTGCTG
Cbrig.WS247 Cbrig.WS247_Fam_83_45_2 2 45 0.762963 AcGGAaCCTTCCACCAcAACcACTGAAgCTcCaACCACCACCACc
Cbrig.WS247 Cbrig.WS247_Fam_84_41_2 2 41 1.000000 GACGACAAAATGCACAAAATTGAAACTAATACGCCAGAGTT
Cbrig.WS247 Cbrig.WS247_Fam_85_36_2 2 36 0.703704 cCCTACAGTAATcCTTTTGATTTTcaAtTaATTcTT
Cbrig.WS247 Cbrig.WS247_Fam_86_36_2 2 36 0.888889 TCCTGGTGGTCCTGGTGGTCCaGaTTGTCCGTCGAc
Cbrig.WS247 Cbrig.WS247_Fam_87_34_2 2 34 0.607843 TGAAATTCtGaAAAtTaTGaAAAcTCAAAtAaTC
Cbrig.WS247 Cbrig.WS247_Fam_88_32_2 2 32 0.833333 CgACATTTTAGAACcGCGACaTTTCAaAACCG
Cbrig.WS247 Cbrig.WS247_Fam_89_30_2 2 30 0.733333 TTCTAGAACCacCTaTTccAGaTCTGGACG
Cbrig.WS247 Cbrig.WS247_Fam_90_27_2 2 27 1.000000 GGAGCCCCAGGACCACAAGGAGATGCC
Cbrig.WS247 Cbrig.WS247_Fam_91_26_2 2 26 0.897436 GAAACTACAGTAAcCCCAGGaTCCTA

Cbrig.WS247 Cbrig.WS247_Fam_92_23_2 2 23 0.659420 gGTaCCgAAAaaGGCTCAAAATG
Cbrig.WS247 Cbrig.WS247_Fam_93_22_2 2 22 0.636364 GCCTAACcTTTgcTTaTTAaTG
Cbrig.WS247 Cbrig.WS247_Fam_94_21_2 2 21 0.746032 AAATTcaTcATTTTcAGGTCG
Cbrig.WS247 Cbrig.WS247_Fam_95_21_2 2 21 0.682540 ACATGGcTCCGTcGgGGgCTc
Cbrig.WS247 Cbrig.WS247_Fam_96_21_2 2 21 1.000000 TGGCCTAGAAAATAGGACTAG
Cbrig.WS247 Cbrig.WS247_Fam_97_20_2 2 20 0.725000 CGTTCCGTGATTTTaTTttt
Cbrig.WS247 Cbrig.WS247_Fam_98_20_2 2 20 0.600000 AACGGCCAAATcgTTaCgaT
Cbrig.WS247 Cbrig.WS247_Fam_99_20_2 2 20 0.933333 TGAGCCTACTTTTTGAGGaC
Cbrig.WS247 Cbrig.WS247_Fam_100_18_2 2 18 0.611111 GGaTTAcTGTAGTcATac
Cbrig.WS247 Cbrig.WS247_Fam_101_18_2 2 18 0.703704 CATAGaTaCCAaAgGTAC
Cbrig.WS247 Cbrig.WS247_Fam_102_16_2 2 16 0.708333 TgAGaTTTTTTaATTC
Cbrig.WS247 Cbrig.WS247_Fam_103_16_2 2 16 0.677083 aATCaGAAATaTTGAa
Cbrig.WS247 Cbrig.WS247_Fam_104_16_2 2 16 0.625000 cTCTGGAaCtCAGAAa
Cbrig.WS247 Cbrig.WS247_Fam_105_16_2 2 16 1.000000 GAAATTCGATAACAGA
Cbrig.WS247 Cbrig.WS247_Fam_106_16_2 2 16 0.750000 TCCaAGATTCCGAaaC
Cbrig.WS247 Cbrig.WS247_Fam_107_12_2 2 12 0.722222 AAAAAtTCGAcA
Cbrig.WS247 Cbrig.WS247_Fam_108_12_2 2 12 1.000000 AAGCCTAAGCCT
Cbrig.WS247 Cbrig.WS247_Fam_109_12_2 2 12 0.777778 CATCTTCAcaTT
Cbrig.WS247 Cbrig.WS247_Fam_110_12_2 2 12 0.888889 GaAAACGGAAAC
Cbrig.WS247 Cbrig.WS247_Fam_111_11_2 2 11 0.636364 ATGGTAagCcT
Cbrig.WS247 Cbrig.WS247_Fam_112_11_2 2 11 0.757576 ATTTTTCAGcc
Cbrig.WS247 Cbrig.WS247_Fam_113_11_2 2 11 0.757576 GACCaGCGcCT
Cbrig.WS247 Cbrig.WS247_Fam_114_10_2 2 10 0.600000 CgAGAaCCAa
Cbrig.WS247 Cbrig.WS247_Fam_115_10_2 2 10 0.600000 TTCTAGacCa
Cbrig.WS247 Cbrig.WS247_Fam_116_200_1 1 200 0.000000
AAATCTTTCAAAATCTAGGACCTACTCAAAAAGGAGAGGAAGATTCTTAAGATTTTCAAAGAATTCAGAAGACAGGTTCCAATCTTCAATACACTTTCGAACACAGCTAAACCTTTGACTGACCGAAATATCAGTCAGCCATAGCCGTCGGGGCTCGAAA
GTGTAAAGGAAACCGGGAAATTGTCAAAAGAATACTAAGA
Cbrig.WS247 Cbrig.WS247_Fam_117_164_1 1 164 0.000000
AAACAATTTTGAAATGAAGGAATGGTTTAGTTCATTTTATTAAAAAATAATTTATTTGAAAACGGAACAGTATGAAAACGCAGTTTGCAAAAATAAAAAAATCGCATTCCAATTTTCAAATTTCTGAATTTCTTTTCTAAAAATCATAAAATTCAAAAAA
AAAC
Cbrig.WS247 Cbrig.WS247_Fam_118_163_1 1 163 0.000000
TCAGAAAAATATTGGAAGCGAAAATAGCGATTTGGACGATCAAAAACTAATTTTTCGAATATTTAGCATATGAATTTTCGAAAAAGCCAGATCAAACAGATCTTTAAACAACATTATGACTGCGTTAAACGTTTTACAGAAATATTTGCGTGAATTCGTT
AAT
Cbrig.WS247 Cbrig.WS247_Fam_119_163_1 1 163 0.000000
GAGATCTGCATGTTCTGCCGTTTTATAAAATTCATATGAGAAAAATTTGAAAAATCTGTTTCTGACCCCCAAAATCGTCATTTTCGTTTCCCACATTTTTCAGAATGAACGAATTCACTCGAATCGTTTTGTAAAATGTTAATTACTACTATCCGTCAAA
TCT
Cbrig.WS247 Cbrig.WS247_Fam_120_161_1 1 161 0.000000
TGACGTCTTCTGGATCTGATGGAGCGCAATTGCAATGACTTAAATTTCAAAACACTGTAACTTCTTTTCTAGTGGACCAAATTTTTTGATTTTTACACCATTAAAGAGGATTTTTTTTTGAATTTTAGCTGGCAATTCAAAATTTCAACAAATTCTATTT
T
Cbrig.WS247 Cbrig.WS247_Fam_121_156_1 1 156 0.000000
GTGATGACCTAATTGAAAAAAAGCAAAGTTTGACAAATTTGACTTTGGAGCTCTTGTGCGACCGCTCTTTTACAATTTTTTCAGCTTACGACAGTCGCTAACTTAATTTTAATAGCTAGCTTCAGAAAATAGAACCTATGTAATTTCAAATTGCCA
Cbrig.WS247 Cbrig.WS247_Fam_122_145_1 1 145 0.000000
GGATTACTGTAGTTTTGTCTGTTCTACTAAAACTACGGTATCTCAAGCTACAGTAATCCTAGAAACATGAAAAATACACCAATCGACTCGGTTCGTTGTTAACTTTCGATTGAATACACTCTTGATTATATTTGAGAAAGTTTTT
Cbrig.WS247 Cbrig.WS247_Fam_123_145_1 1 145 0.000000
TTCTAGGCCATCAACCTAAAAATGGTCATAACTTGGCTGAAAATTGAGATTTTGAGCTGAAACTGAATCCAATAGGTTGCTGAGATCATCCCTGTCATTTTAAACCTATTTACGTCCTGGTGGCATAGAAACCCAACATTTGCAT
Cbrig.WS247 Cbrig.WS247_Fam_124_141_1 1 141 0.000000
GTGTTTGCGTACTTTTGGAACTACAGTAGCCCGAAAAAAAGAATTTCACGGAATCCTATTGCTATGGGTATCAAAATGTCAGAAATTTCATATAAAATCCAGTTATTATGTTTTGAAAATCAAAAAATTGCGCTAGGCGTG
Cbrig.WS247 Cbrig.WS247_Fam_125_140_1 1 140 0.000000
GATTCTATAGGAATGAAACATACTCTGGCTCCTCGTTACTGTTGGTAGTGGTTCCGCGTGATTTCACGTTTTTTAGAATAGAAGCTGTTTTGCGTGGGAGTGGGTTTGGAGTCATTCTAAAATTGAAAGGTTTTCTTGTA
Cbrig.WS247 Cbrig.WS247_Fam_126_136_1 1 136 0.000000
GAATTCTCAGAAAATCATTCAAAATCTAGGACCTACTTAGGAAAGGGGAGGAAGATTCTGAATATTTTAAAAGAATTCAGAAGACAATTCTCAATCTTCAATACCCTTTCGAACACAGCTAAACCTTTGACTGACT
Cbrig.WS247 Cbrig.WS247_Fam_127_131_1 1 131 0.000000
AGTCAAAAATCAAGCTGAAAAGTCTTCTTTTTCTAGCGGTGTTTGACTTAGCTGCCATATGGGTTGAAGACTATCCTCACGAGCTGAGATGGGAACTTTTTCGCCCAATTTCTTGAAAAAATCTCTGTAAA
Cbrig.WS247 Cbrig.WS247_Fam_128_122_1 1 122 0.000000
AATTTCAGGCCAGTGGAATGTGCCAACTGACAGTAGCAGGACCTGTGTAACTGGGAAGAAGACTCGATGGCAATTTGACCTTCCAATTGAGCTTAATAGCCTGAAATTCAAGGAAGATAGCT
Cbrig.WS247 Cbrig.WS247_Fam_129_119_1 1 119 0.000000
AAATTATTTTCACGTCTTCATTTTTAAGTTTTTTGTGAATATCTAGGTCAAAAATTGAAATTCTGGATTTTTAAAAACACGTTCGGAATGCACTATTTTTTTCTGAATCCATTAATATAA

Cbrig.WS247 Cbrig.WS247_Fam_130_108_1 1 108 0.000000
GGAGAACGATATGCCAAGAAAGTCCCAGCTGACAACAAGATTCTCCAAGGTGATGGCCAACAGTTCGAAGCCAAAACACAGAATCAAGAGGACTTCACTCACATCAGT
Cbrig.WS247 Cbrig.WS247_Fam_131_102_1 1 102 0.000000
TGGTCTTGGTCCGAATCCCGATGGTCCCTGTCCACGTTCTCCACGTCCTCCCGGTCCACGACGATCTGGGAAGTCCGAGTCAGGTCCAAAGTCTGGTCCTTG
Cbrig.WS247 Cbrig.WS247_Fam_132_102_1 1 102 0.000000
AATTGATCGTCCTACTGAAGGTCTAGACTCAACCACCATCAACAGATCCACGTATCAAGGAGGCACTGGTGACAGATTCCAAGCTTCAAGACCAAAAGATGC
Cbrig.WS247 Cbrig.WS247_Fam_133_99_1 1 99 0.000000
TCCGATGAAGAATCGGCCTTCTTAGCAGCTGTAGACTTTGGAGTAACCTTTGCTGGAGTTGTTTTAGCCGCAGGCTTTTTCTCGTCGTCAGAAGAATCG
Cbrig.WS247 Cbrig.WS247_Fam_134_88_1 1 88 0.000000
TTTTTCAGAATGAACGAATTCACTTGAATTATTTTGTAAAATGTTAATTACTACTATCCGTCAAATCTGAGATCTGCATGTTCTGCCG
Cbrig.WS247 Cbrig.WS247_Fam_135_73_1 1 73 0.000000 ACAAACTACATAGGAAACATTAGCAGACACATCATGCGCGTATTAGAAATGCGTCAGTTGGGTCTGTTAGACT
Cbrig.WS247 Cbrig.WS247_Fam_136_72_1 1 72 0.000000 CAAGGAAGTCACCTTTGAACCACGTAACGTTCGTAGACGGAGCTTCGGTTCCCCTACACCCGACCACTAGTT
Cbrig.WS247 Cbrig.WS247_Fam_137_72_1 1 72 0.000000 GATCCACAGTAACCAAATCTTCTTCTTTATCCGCTGTCCCATCAACCACACAGGCAACATCTAGTTCTAAGG
Cbrig.WS247 Cbrig.WS247_Fam_138_66_1 1 66 0.000000 ATGGTACGTCGCCGACTACTGACCGTACGCCAAGAGCACACGAGAAAATGATGTCTGCCACCGTTG
Cbrig.WS247 Cbrig.WS247_Fam_139_65_1 1 65 0.000000 CTGAGAATTCGTTTGACATTGTCTCGGTTTCCTTTACTCTTTCAAGCCCCGACGGCTATGGCTGA
Cbrig.WS247 Cbrig.WS247_Fam_140_64_1 1 64 0.000000 TGTCTAATTGGGAGACGACGAGAGGAACGACGGGGGGACGGTGGGACGGATGGAGGAGAGATGG
Cbrig.WS247 Cbrig.WS247_Fam_141_63_1 1 63 0.000000 GAGACTCCAGAGACTCCAGAAACATCAGAGACCACCTTGACTCCTCCAACTGGAACTACAGCC
Cbrig.WS247 Cbrig.WS247_Fam_142_63_1 1 63 0.000000 CTGGACGCTTCTTCTCCTCCTCAGCTCCTTGGGTTCCCTTTGGCCGATCCTCAGCAGCTCTAG
Cbrig.WS247 Cbrig.WS247_Fam_143_63_1 1 63 0.000000 TCCTTGAGTCTTGGCTCCAACTTGGGCGGCAGCTCTGGCTGGGCGGTGTTGTTGAGCATCAGC
Cbrig.WS247 Cbrig.WS247_Fam_144_61_1 1 61 0.000000 GATTACTGTAGATTGGGTCAAAAGAGTCATTTTAAGTCAAGAATCGAAATTTTTGGATCAG
Cbrig.WS247 Cbrig.WS247_Fam_145_60_1 1 60 0.000000 ACGGAACCATGTCTGAATTCCACGTCTTCCGAAACTACCACAGAACCCTGTGAGACCACG
Cbrig.WS247 Cbrig.WS247_Fam_146_57_1 1 57 0.000000 TTTCCGAATTTCTGAATTTCAGGATTTCAAGGATTTCAGGACTTCAGGACTTCAGGA
Cbrig.WS247 Cbrig.WS247_Fam_147_56_1 1 56 0.000000 CCAGAAAACGCATCAGATGAATATCAGGTCATTTCTAGGCCATTTCTACATGGTGG
Cbrig.WS247 Cbrig.WS247_Fam_148_56_1 1 56 0.000000 TTCTAGGCCACCGTAGTCGATGGTCCAGAAATTGTCAGAAATTCACATGGTGGGAT
Cbrig.WS247 Cbrig.WS247_Fam_149_54_1 1 54 0.000000 TCTGGAGAAGAGGCTACCACTGCTGCCGCCACTGAAGCCACAGTCGCCGAAGCA
Cbrig.WS247 Cbrig.WS247_Fam_150_54_1 1 54 0.000000 TGTGCTAGAAAGATGCTTTTTAACCACCATGTTGAAAGATTCACTGAAGTGCGA
Cbrig.WS247 Cbrig.WS247_Fam_151_54_1 1 54 0.000000 TTTTTTAATTTTTGAACACTGCGTTTTCATACTGTTCCGTTCTCAAATGAATTA
Cbrig.WS247 Cbrig.WS247_Fam_152_51_1 1 51 0.000000 TTCTTCAGATCCTCCGACTGGTGCTTGTGAAGATGCATCGGATGGATTTGG
Cbrig.WS247 Cbrig.WS247_Fam_153_48_1 1 48 0.000000 TGGCCTAGAAAATGCAAAAATCTGGAAAATTAGACAATTTTGAGTGGG
Cbrig.WS247 Cbrig.WS247_Fam_154_48_1 1 48 0.000000 TCAGTTGTAACAAGTTCTCCGTCTGGTCCAATGGCAAATCCAGTGGGA
Cbrig.WS247 Cbrig.WS247_Fam_155_48_1 1 48 0.000000 TTTCTAGGCCATCGGGCAAAATAACATGTGGTGGCCTAGAAATCCAAG
Cbrig.WS247 Cbrig.WS247_Fam_156_47_1 1 47 0.000000 TTTCTAGGCCACGAAAAGTTTGATGGACTAGGTTTTCGATTTTAAGA
Cbrig.WS247 Cbrig.WS247_Fam_157_46_1 1 46 0.000000 TTTCTAGGCCGTATGCTCTTCATGGCCTGGGAATCCCGTTTTTGGG
Cbrig.WS247 Cbrig.WS247_Fam_158_45_1 1 45 0.000000 ACACCAGCAGGCCCGTCTGGACCAACCATGACGACAACAAAACCT
Cbrig.WS247 Cbrig.WS247_Fam_159_43_1 1 43 0.000000 GTCAAAAATCCGTAATTTTGATCCGAAATTTCATATTTTTTAT
Cbrig.WS247 Cbrig.WS247_Fam_160_43_1 1 43 0.000000 AATTTGGCTGAAATTGCTAGAAAAATAGGATAAAAATGGCGGA
Cbrig.WS247 Cbrig.WS247_Fam_161_42_1 1 42 0.000000 GAGAGCATTTTTTAAATTTCCAAAAGATGCAATTTCTCAATA
Cbrig.WS247 Cbrig.WS247_Fam_162_42_1 1 42 0.000000 ATTTTTGACCCAATTATGACCATTTATAGCCAAATTTGAGTC
Cbrig.WS247 Cbrig.WS247_Fam_163_42_1 1 42 0.000000 GGTCATTTTTTGACTATTTTAGGCCTATTTTTGGACCAATTA
Cbrig.WS247 Cbrig.WS247_Fam_164_42_1 1 42 0.000000 TAGTGATTTTCGGCTCAAAAATCCGCTTTTTGGGGTCAAAAT
Cbrig.WS247 Cbrig.WS247_Fam_165_42_1 1 42 0.000000 AAATGACTATTTTCAGCCTAAAACTGGCTATTTTAAGCCTGA
Cbrig.WS247 Cbrig.WS247_Fam_166_42_1 1 42 0.000000 GCTTAAAATGACCAATTTTGGATCTAATTTTGCTCATTTTTG
Cbrig.WS247 Cbrig.WS247_Fam_167_42_1 1 42 0.000000 TTTTGGACCACTTTGCATGAATTTTGAACCGTTTTCCATGAA
Cbrig.WS247 Cbrig.WS247_Fam_168_41_1 1 41 0.000000 CGGAAAATCGAGTTCTCGAATTTTTTCTCGACTTTTTTTTC
Cbrig.WS247 Cbrig.WS247_Fam_169_39_1 1 39 0.000000 TGCCACGTGGCTGCTGCTGGTGCTGCTGGTTCTGATAAT
Cbrig.WS247 Cbrig.WS247_Fam_170_38_1 1 38 0.000000 TGGTACCATAGGTTTTCAATAGTTCCATGGAGTTTAAA
Cbrig.WS247 Cbrig.WS247_Fam_171_38_1 1 38 0.000000 CGCAAAGGCACCAAAAATGCGTCAAGACACTAGAAAAG
Cbrig.WS247 Cbrig.WS247_Fam_172_38_1 1 38 0.000000 AGCCGATCGGCGATCGGCTAGTGTGGTTCGGATGTGTC
Cbrig.WS247 Cbrig.WS247_Fam_173_37_1 1 37 0.000000 GGACAACTGAGAAAAATTTGTTTTCACCAAAACAGAT
Cbrig.WS247 Cbrig.WS247_Fam_174_36_1 1 36 0.000000 TACTGTAGAAAAAAGGGAATTTTCAAAATCAAGAAC
Cbrig.WS247 Cbrig.WS247_Fam_175_36_1 1 36 0.000000 GGAGCTCCAGGAGCCCCAGGACAAGGATCAGGAGCC
Cbrig.WS247 Cbrig.WS247_Fam_176_35_1 1 35 0.000000 TACAGTAATCCTTTGGAATTGGTAAATAAAAAATC
Cbrig.WS247 Cbrig.WS247_Fam_177_35_1 1 35 0.000000 TGGCCTAGAAAACCAAATATTGCAATTCTCAGCCA
Cbrig.WS247 Cbrig.WS247_Fam_178_35_1 1 35 0.000000 GGGCCCTTCTATTACAGGGGCCTTTCAATTAGAGG
Cbrig.WS247 Cbrig.WS247_Fam_179_34_1 1 34 0.000000 ACTCGGAATAAGTAGTACCAATTCGATACTACTT
Cbrig.WS247 Cbrig.WS247_Fam_180_32_1 1 32 0.000000 ATTCTCAGATTCTCAGATTCTGAAGTTGCGGA
Cbrig.WS247 Cbrig.WS247_Fam_181_32_1 1 32 0.000000 AGCCATTTTTGACCTATTTTAGCCAATTTGC

Cbrig.WS247	Cbrig.WS247_Fam_182_31_1	1	31	0.000000	ATTCCGGATTCCGGATTCTGTCCATTTTTTC
Cbrig.WS247	Cbrig.WS247_Fam_183_31_1	1	31	0.000000	GTTGCAAAATGCCTGGTTCTATAACGTCACG
Cbrig.WS247	Cbrig.WS247_Fam_184_31_1	1	31	0.000000	TTTCAGCGATCCTCAGCTATTTTTAGGCATG
Cbrig.WS247	Cbrig.WS247_Fam_185_31_1	1	31	0.000000	AGCGATTTTTCTAGGAATTTTGAGGTTTTTA
Cbrig.WS247	Cbrig.WS247_Fam_186_31_1	1	31	0.000000	AAAATCATGATTTTTAGGTTAAAATTGACCG
Cbrig.WS247	Cbrig.WS247_Fam_187_31_1	1	31	0.000000	GCAGTAGCAAATTCGGTGTTACTACTGCTTA
Cbrig.WS247	Cbrig.WS247_Fam_188_30_1	1	30	0.000000	CTATAAACTACAGAATCTAGACTACAGAAG
Cbrig.WS247	Cbrig.WS247_Fam_189_30_1	1	30	0.000000	TCTAATAGCTCAAAAAATGGGTCCCAATGT
Cbrig.WS247	Cbrig.WS247_Fam_190_30_1	1	30	0.000000	CTGGAACTTTCTAGAACGTTGTGGACTTTT
Cbrig.WS247	Cbrig.WS247_Fam_191_29_1	1	29	0.000000	GATCCAGGTCACCCGGTCAAGGTCACCCG
Cbrig.WS247	Cbrig.WS247_Fam_192_29_1	1	29	0.000000	GAATCGGAGAATCGGAGCTGGTGAAGGTG
Cbrig.WS247	Cbrig.WS247_Fam_193_29_1	1	29	0.000000	AAAATTAGGTCACGAAATAGAAATACGAG
Cbrig.WS247	Cbrig.WS247_Fam_194_29_1	1	29	0.000000	GATGCCAGTGATATACTCCCGTATATCCT
Cbrig.WS247	Cbrig.WS247_Fam_195_28_1	1	28	0.000000	TACAGTAATCCAAGAAATTAGTTAAAAC
Cbrig.WS247	Cbrig.WS247_Fam_196_28_1	1	28	0.000000	GAAAAATTGGGGGTCAAAAAATCGAAGT
Cbrig.WS247	Cbrig.WS247_Fam_197_28_1	1	28	0.000000	GGATTCCGGACCCAGAAGTCCGGAGTCA
Cbrig.WS247	Cbrig.WS247_Fam_198_27_1	1	27	0.000000	GTGGTAGTCTCCTCGACATATGGAGTC
Cbrig.WS247	Cbrig.WS247_Fam_199_27_1	1	27	0.000000	TTCCACGTCTTCCACGTCTTCCACGTT
Cbrig.WS247	Cbrig.WS247_Fam_200_27_1	1	27	0.000000	CCGAGGATCCCATGGATCTCAAGGATC
Cbrig.WS247	Cbrig.WS247_Fam_201_27_1	1	27	0.000000	TACAGTGCTTACAGTGCTTACAGTGCT
Cbrig.WS247	Cbrig.WS247_Fam_202_27_1	1	27	0.000000	TATTTTTGAGGCACGTAACGTGCCTTG
Cbrig.WS247	Cbrig.WS247_Fam_203_27_1	1	27	0.000000	CCACCATTTCCTCCATTTCCGCCATGG
Cbrig.WS247	Cbrig.WS247_Fam_204_27_1	1	27	0.000000	CAAGTACCGTACTTTTGATCTCTGAAA
Cbrig.WS247	Cbrig.WS247_Fam_205_26_1	1	26	0.000000	ACCTAATTTTACAAAATCGAGGTCCG
Cbrig.WS247	Cbrig.WS247_Fam_206_26_1	1	26	0.000000	GAATACTACAATAGTCCTAATATCCA
Cbrig.WS247	Cbrig.WS247_Fam_207_26_1	1	26	0.000000	GACAATATCAACGAAGGAGATGAGCC
Cbrig.WS247	Cbrig.WS247_Fam_208_25_1	1	25	0.000000	CATAGCTGACATTGCTGACCTAGAC
Cbrig.WS247	Cbrig.WS247_Fam_209_25_1	1	25	0.000000	ATTGAAATCTTGTTCATTGCCTAGA
Cbrig.WS247	Cbrig.WS247_Fam_210_25_1	1	25	0.000000	TCTGAATCTCTGAATTACCTATTAT
Cbrig.WS247	Cbrig.WS247_Fam_211_24_1	1	24	0.000000	TATCAGAATCTCATAGATTGAAGA
Cbrig.WS247	Cbrig.WS247_Fam_212_24_1	1	24	0.000000	CAAAAGGGGTCGATTTTGAAAATT
Cbrig.WS247	Cbrig.WS247_Fam_213_24_1	1	24	0.000000	TCCTCCTTGGGATGTTCCTCCGAA
Cbrig.WS247	Cbrig.WS247_Fam_214_24_1	1	24	0.000000	GATGTACCCTGGAATCCTTTCCCT
Cbrig.WS247	Cbrig.WS247_Fam_215_23_1	1	23	0.000000	GCCTAGAAAACTCCTGAATTGGT
Cbrig.WS247	Cbrig.WS247_Fam_216_23_1	1	23	0.000000	TGGCCTAAAAACCCTGTTTTCAA
Cbrig.WS247	Cbrig.WS247_Fam_217_22_1	1	22	0.000000	AAATAAGCCTAAATTAGGTCAA
Cbrig.WS247	Cbrig.WS247_Fam_218_22_1	1	22	0.000000	GGTATCATTTTCAATAAATTAT
Cbrig.WS247	Cbrig.WS247_Fam_219_22_1	1	22	0.000000	ACCATATGATACATAGAGATGA
Cbrig.WS247	Cbrig.WS247_Fam_220_22_1	1	22	0.000000	AAAATGGACCTGAAGGAGGGCA
Cbrig.WS247	Cbrig.WS247_Fam_221_21_1	1	21	0.000000	AAGTTAGGCCACGACTTTCGG
Cbrig.WS247	Cbrig.WS247_Fam_222_21_1	1	21	0.000000	GGTTACTGTAGCTTTTTTCTG
Cbrig.WS247	Cbrig.WS247_Fam_223_21_1	1	21	0.000000	GGGATTCTAGGTCATGGTATT
Cbrig.WS247	Cbrig.WS247_Fam_224_21_1	1	21	0.000000	CATGGGCAAGTACAGGCAAGC
Cbrig.WS247	Cbrig.WS247_Fam_225_21_1	1	21	0.000000	TCTAGCTTAAAATAGGCCTTT
Cbrig.WS247	Cbrig.WS247_Fam_226_21_1	1	21	0.000000	CTACAAACTACAACGCCTAGG
Cbrig.WS247	Cbrig.WS247_Fam_227_21_1	1	21	0.000000	AAGGTACCATTGTATCATTGA
Cbrig.WS247	Cbrig.WS247_Fam_228_21_1	1	21	0.000000	TCCGGATCCCGGATGCCTGTG
Cbrig.WS247	Cbrig.WS247_Fam_229_21_1	1	21	0.000000	GATTCGGTCGAGGTACTGGTG
Cbrig.WS247	Cbrig.WS247_Fam_230_21_1	1	21	0.000000	ACTACGGAACCTTCCACTATC
Cbrig.WS247	Cbrig.WS247_Fam_231_21_1	1	21	0.000000	ATTTTGGCTCTTGAAACCCTG
Cbrig.WS247	Cbrig.WS247_Fam_232_21_1	1	21	0.000000	AAAATAGGTCATGCCATTTAA
Cbrig.WS247	Cbrig.WS247_Fam_233_20_1	1	20	0.000000	CTGACCGACCACCGATCAAT
Cbrig.WS247	Cbrig.WS247_Fam_234_20_1	1	20	0.000000	TTTCTAGGCCATCAGTGAGG
Cbrig.WS247	Cbrig.WS247_Fam_235_20_1	1	20	0.000000	AAATTCAGAAAATTGACGGC
Cbrig.WS247	Cbrig.WS247_Fam_236_20_1	1	20	0.000000	GTCTATTAGAAGAGATGCAG
Cbrig.WS247	Cbrig.WS247_Fam_237_20_1	1	20	0.000000	AAGATGGTACCTCAGCTGGG
Cbrig.WS247	Cbrig.WS247_Fam_238_20_1	1	20	0.000000	AGCATGATCCCATCCCTCTC

Cbrig.WS247	Cbrig.WS247_Fam_239_19_1	1	19	0.000000	ACCATTGAAGTGCCATGGA
Cbrig.WS247	Cbrig.WS247_Fam_240_19_1	1	19	0.000000	TACTGTAGTTCAAAAAGAC
Cbrig.WS247	Cbrig.WS247_Fam_241_19_1	1	19	0.000000	AAATGTGTCAAGGAATCAA
Cbrig.WS247	Cbrig.WS247_Fam_242_19_1	1	19	0.000000	AAACCACGAGAGTCCCAAA
Cbrig.WS247	Cbrig.WS247_Fam_243_19_1	1	19	0.000000	TTAGTCCTAACCGCAAGAG
Cbrig.WS247	Cbrig.WS247_Fam_244_18_1	1	18	0.000000	TTTTGAAAAAAAAAATTT
Cbrig.WS247	Cbrig.WS247_Fam_245_18_1	1	18	0.000000	CAGAAGACGTCAAGGATT
Cbrig.WS247	Cbrig.WS247_Fam_246_18_1	1	18	0.000000	CAGATGGTTCCGGTTCAT
Cbrig.WS247	Cbrig.WS247_Fam_247_18_1	1	18	0.000000	GGGTCTCTGGTGACCCGG
Cbrig.WS247	Cbrig.WS247_Fam_248_17_1	1	17	0.000000	AGGGTACATCGGATCAG
Cbrig.WS247	Cbrig.WS247_Fam_249_17_1	1	17	0.000000	TGTTTTGTTCTAATATC
Cbrig.WS247	Cbrig.WS247_Fam_250_16_1	1	16	0.000000	GTAAAATTTTAGGCTT
Cbrig.WS247	Cbrig.WS247_Fam_251_16_1	1	16	0.000000	GATTCTGGATTTTAAG
Cbrig.WS247	Cbrig.WS247_Fam_252_16_1	1	16	0.000000	CTCGCTGTCTCGCTGT
Cbrig.WS247	Cbrig.WS247_Fam_253_16_1	1	16	0.000000	TTTGCAACTAGGAAAA
Cbrig.WS247	Cbrig.WS247_Fam_254_16_1	1	16	0.000000	CTATAACGCTATAACG
Cbrig.WS247	Cbrig.WS247_Fam_255_16_1	1	16	0.000000	TTAATGGGTTAATGTT
Cbrig.WS247	Cbrig.WS247_Fam_256_16_1	1	16	0.000000	TGGTCCTCTGGTCCAC
Cbrig.WS247	Cbrig.WS247_Fam_257_16_1	1	16	0.000000	AAGCCCGAAAGTTTGG
Cbrig.WS247	Cbrig.WS247_Fam_258_16_1	1	16	0.000000	TTCATAGGGCGACGAG
Cbrig.WS247	Cbrig.WS247_Fam_259_16_1	1	16	0.000000	CAGTTCTATTCCAGAG
Cbrig.WS247	Cbrig.WS247_Fam_260_15_1	1	15	0.000000	TGTAAGCTATGAGCT
Cbrig.WS247	Cbrig.WS247_Fam_261_15_1	1	15	0.000000	CATCCTCCGCTGGAT
Cbrig.WS247	Cbrig.WS247_Fam_262_14_1	1	14	0.000000	AAGTGAAAAAATAT
Cbrig.WS247	Cbrig.WS247_Fam_263_14_1	1	14	0.000000	TTTTTGTGCTATAA
Cbrig.WS247	Cbrig.WS247_Fam_264_14_1	1	14	0.000000	ACCGCAGACCTAAG
Cbrig.WS247	Cbrig.WS247_Fam_265_14_1	1	14	0.000000	CTCGGGGCTCGGGG
Cbrig.WS247	Cbrig.WS247_Fam_266_14_1	1	14	0.000000	TACGAACTACATAC
Cbrig.WS247	Cbrig.WS247_Fam_267_12_1	1	12	0.000000	GGATGGACGGAT
Cbrig.WS247	Cbrig.WS247_Fam_268_12_1	1	12	0.000000	TGGTGGTGGAGG
Cbrig.WS247	Cbrig.WS247_Fam_269_12_1	1	12	0.000000	GCCCTAGCCCTA
Cbrig.WS247	Cbrig.WS247_Fam_270_12_1	1	12	0.000000	ATGGGACCCATC
Cbrig.WS247	Cbrig.WS247_Fam_271_11_1	1	11	0.000000	GCGAGCGACGG
Cbrig.WS247	Cbrig.WS247_Fam_272_11_1	1	11	0.000000	TTTGGGCAAGT
Cbrig.WS247	Cbrig.WS247_Fam_273_11_1	1	11	0.000000	AGCCGGCCGAC
Cbrig.WS247	Cbrig.WS247_Fam_274_10_1	1	10	0.000000	TCGAGTCTCG
Cbrig.WS247	Cbrig.WS247_Fam_275_10_1	1	10	0.000000	GTGTGTGGGT

C. brenneri

Genome	Family name	Number of sequences	Alignment length	Score	Consensus without gaps
Cbren.WS247	Cbren.WS247_Fam_1_10_97	97	11	0.805295	TaGAAGTTTC
Cbren.WS247	Cbren.WS247_Fam_2_18_65	65	18	0.935835	AATTCCCATACCCAAAAT
Cbren.WS247	Cbren.WS247_Fam_3_28_64	64	39	0.524242	ntTTTtGAAttTTTGAATTCCCgCCAAA
Cbren.WS247	Cbren.WS247_Fam_4_15_62	62	18	0.695169	GGTCCCCGATTTTTc
Cbren.WS247	Cbren.WS247_Fam_5_20_44	44	26	0.552136	tTTTTTgGGtCCtCgATTTt
Cbren.WS247	Cbren.WS247_Fam_6_20_42	42	23	0.627161	GGCCTAACTTTtcaAAaTAn
Cbren.WS247	Cbren.WS247_Fam_7_52_38	38	55	0.764619	TCCATTACCTGggtAAATGgCAgCAGGTAGCTATACGTTGCccTtTcCCTAA
Cbren.WS247	Cbren.WS247_Fam_8_12_37	37	12	0.700701	GCttAGGCTtAG
Cbren.WS247	Cbren.WS247_Fam_9_10_36	36	14	0.600340	GCCTTAGACA
Cbren.WS247	Cbren.WS247_Fam_10_24_31	31	32	0.634543	AGGTCATGACCTAGAAAAAAAATT
Cbren.WS247	Cbren.WS247_Fam_11_10_27	27	11	0.707589	CCGGTCTGGT
Cbren.WS247	Cbren.WS247_Fam_12_20_26	26	21	0.721074	AAAATCACGGTCCCAattnt
Cbren.WS247	Cbren.WS247_Fam_13_30_25	25	30	0.913185	ATCGGGGACCgAAAaATcGtGGCCCGAAAA
Cbren.WS247	Cbren.WS247_Fam_14_20_23	23	27	0.459450	CTTaGCCTCTACAAAnnnnc
Cbren.WS247	Cbren.WS247_Fam_15_16_22	22	17	0.715559	ATCTGGAcATCcGGAc
Cbren.WS247	Cbren.WS247_Fam_16_15_20	20	17	0.697110	GAAACCgGACATTTc
Cbren.WS247	Cbren.WS247_Fam_17_16_20	20	17	0.714190	TCtGtATGTCTGTaTG
Cbren.WS247	Cbren.WS247_Fam_18_161_18	18	163	0.770988	TCCAACTtTTAGAAAAATCgAATtTTTTCAGTAgAnTCGAAAaTTcAAGattTTTTTgCATTGtCCTGGCGaCagCtaGaATcGTTtGaAcTGagCATATtaTGcATTGCcAGanTAaTTCTGACCAtTTGACAtCAATTttgAGtGtGCcCCactCAGAT
Cbren.WS247	Cbren.WS247_Fam_19_174_17	17	176	0.865948	AAAATCCAAAAATATGAcCttTTTtaTCCTGATATTTTaGGATTTTGGTCTATTTCTGTCCGCCCTGAAAAAGTTTGAGCTTCTAATGGTCCAGActtGTGaTAATTTGTTTCAATACGTTGTAGTTCATCATTTtGTGCTGtaTTCTCGATTGAAAAaTtaATTTTTTAGTCA
Cbren.WS247	Cbren.WS247_Fam_20_15_17	17	15	0.870588	ATGTCCCAGAGGGAA
Cbren.WS247	Cbren.WS247_Fam_21_14_15	15	14	0.916553	GGAAGTCGGAAGtc
Cbren.WS247	Cbren.WS247_Fam_22_169_14	14	174	0.901899	AAACTATAAACCACATTCCTGTTTGCTGGCTGCCTGTACTACACGTATATCTTGATTTTTGCATTCCCGGCTGCAACAGAAGAACAAGACGTTTGTGAGGGCTCGGATGGTGTTTAAaTTGCTTCAaAGACCGTGTCGGAATCGTgATAaCCttTGTAGACCGATATCG
Cbren.WS247	Cbren.WS247_Fam_23_10_14	14	14	0.645474	TCTCTCTCTC
Cbren.WS247	Cbren.WS247_Fam_24_12_14	14	13	0.656805	AAGcgGAAgCgG
Cbren.WS247	Cbren.WS247_Fam_25_12_14	14	13	0.700197	aCAGACAgACAg
Cbren.WS247	Cbren.WS247_Fam_26_28_13	13	32	0.631410	TgTCCGGATGTCCatcTGTgTGTCTGTA
Cbren.WS247	Cbren.WS247_Fam_27_15_13	13	15	0.811966	GCAGCGTTcAAAatc
Cbren.WS247	Cbren.WS247_Fam_28_19_12	12	24	0.494739	TTTaGGCcGtTcGggCCGA
Cbren.WS247	Cbren.WS247_Fam_29_20_11	11	20	0.729091	aTGGCCTAGAAtTcCAAaTg
Cbren.WS247	Cbren.WS247_Fam_30_16_11	11	18	0.758249	CTCTCAgGCTCTCAGG
Cbren.WS247	Cbren.WS247_Fam_31_10_10	10	10	0.760000	GACcAgcCTA
Cbren.WS247	Cbren.WS247_Fam_32_41_9	9	41	0.904246	AAGTAACCGTTGAAATTTTCTgggAACCCGGAAAATTTCCa
Cbren.WS247	Cbren.WS247_Fam_33_20_9	9	22	0.650673	AACTTCtAGaAncTTCCAGA
Cbren.WS247	Cbren.WS247_Fam_34_19_9	9	19	0.822612	ATCGGCCcATttTgcCTAA
Cbren.WS247	Cbren.WS247_Fam_35_17_9	9	18	0.847737	CTCATTGACTaCATTTA
Cbren.WS247	Cbren.WS247_Fam_36_15_9	9	17	0.572985	CCGGaCCgGAcCAGg
Cbren.WS247	Cbren.WS247_Fam_37_16_9	9	16	0.763889	CTGtCTaTCTGtCTaT
Cbren.WS247	Cbren.WS247_Fam_38_169_8	8	169	0.950690	GTTcCACCACAATATGAAAAGATGTAACACGGTGTTTTtaACGCATTGGACACAATCCGAACCCtCATTAACATCCCATTCTGATGATTCACTTGCAGATGCAAAAATCAAGAGAAATCAGTAGTGCAGGCAGCGAGCAGACaGAAATGTCAAATAAAGTTtTGATATC
Cbren.WS247	Cbren.WS247_Fam_39_30_8	8	30	0.888095	ATACATCTGCATGTATTAggGAaGTCgCTA
Cbren.WS247	Cbren.WS247_Fam_40_29_8	8	29	0.857553	aACGCTGCGTTCCAGCGTtcaAAAGTTTc
Cbren.WS247	Cbren.WS247_Fam_41_23_8	8	24	0.644841	TACAGTaATCCtAGAnnaAaAAC

Cbren.WS247	Cbren.WS247_Fam_42_184_7	7	200	0.823492	GGCATCCAACCGGTCCGCACGTGTGCTCTTCcGGTCAAGGATCTCTGACAAGATAGATCCAGTGACcTTCCGAAGCACGTTGCCTGACAGATCGCGGTGGTTGGGCATCCaaAgTGTCTCATTGCTGTgTGGTGTAGGCgCACCtCAGACTATGAGCCTTCGACTnaTcCCTCTTgGGtcAGTC
Cbren.WS247	Cbren.WS247_Fam_43_165_7	7	168	0.858466	GAGTTTTTGAAattGGGAAGGGTTTGGATGGGAGTACTGTcTAaGAGCTgAtGTngAaATCAAGAACAACCAAGTCACATACGCCAAAAAGGAGTGTTTCCACTCGATCCTGCCGCCTAGCTAACTGGATTATTCTTGgGAAAAAAGAAGCTCGGAAAgAAGCTA
Cbren.WS247	Cbren.WS247_Fam_44_58_7	7	65	0.726252	CAGAAaTaTTnGAAAAAGTGACCAAAAATATTTTGGTCATTTTTtTcCAtAAAAACCT
Cbren.WS247	Cbren.WS247_Fam_45_41_7	7	41	0.947348	AAGTCAGATAGGACTTTTGTTCAGGAAGGCCTaTTTGACCA
Cbren.WS247	Cbren.WS247_Fam_46_27_7	7	27	0.934156	ACACACGCACTGTCGATtTTTAGGGGC
Cbren.WS247	Cbren.WS247_Fam_47_18_7	7	18	0.774250	AAATTCCgAAAAtTCtGA
Cbren.WS247	Cbren.WS247_Fam_48_16_7	7	16	0.861111	AAATCTGGCCGAgAGg
Cbren.WS247	Cbren.WS247_Fam_49_189_6	6	190	0.859123	ACGAAGTTGGTTGcTGGTTTTATTGAAAGGCTCTGTTTTCTAGGaCCGGAGTGTCGATCAAAAAAGTAAAATATGGGAAATCCAAACGGTGGATTATCTTCTGATTGACCTGAaaAacCTCtTGTCaAtTTCCCATTCTGAATGGaTCCAAATTGaCTGTGTAATGCATCATAATTCCTGACGAAACTG
Cbren.WS247	Cbren.WS247_Fam_50_45_6	6	46	0.792271	CATCGACCTcCACTTCTGAtgCTCCAAcCACcACAACCaAGaAAC
Cbren.WS247	Cbren.WS247_Fam_51_33_6	6	33	0.876094	CAGCTTGTTCGAcTGGtTTCTCCTCGACTGGAG
Cbren.WS247	Cbren.WS247_Fam_52_24_6	6	24	0.642593	CATCcGGacAgACAGACAtcngGA
Cbren.WS247	Cbren.WS247_Fam_53_22_6	6	24	0.562500	GaGCCaAAAaTTAGaGaTTTTT
Cbren.WS247	Cbren.WS247_Fam_54_21_6	6	23	0.617391	TGaTGGCCTAGatTttgAAAA
Cbren.WS247	Cbren.WS247_Fam_55_22_6	6	23	0.712077	AGGCCATTTTTcaaGGtTTTCT
Cbren.WS247	Cbren.WS247_Fam_56_21_6	6	21	0.915344	ATACCATAGGTGGAAGATCGG
Cbren.WS247	Cbren.WS247_Fam_57_19_6	6	20	0.709444	TAGCAGTTGATCAcCCCaa
Cbren.WS247	Cbren.WS247_Fam_58_16_6	6	19	0.607602	cGGAAGCCcGGAAGCC
Cbren.WS247	Cbren.WS247_Fam_59_18_6	6	18	0.871605	GCCTcAgAAAAGATATCG
Cbren.WS247	Cbren.WS247_Fam_60_15_6	6	15	0.718518	aaTAGCAGTTgAGAc
Cbren.WS247	Cbren.WS247_Fam_61_10_6	6	14	0.528571	AAAAAAAtCG
Cbren.WS247	Cbren.WS247_Fam_62_62_5	5	62	0.873118	ACGACTAGAGACCgAAGTCTagAGCCTGTAGTCTGTTAGcAAggnAaCCAatATgAAGACCT
Cbren.WS247	Cbren.WS247_Fam_63_42_5	5	42	0.876190	TTAGGCTTAGGCTTAGGTGaCAaCaaAAtCTTAGGCTtCaaC
Cbren.WS247	Cbren.WS247_Fam_64_24_5	5	32	0.596875	nncGTGGGAAaATGCCCAGAGGCA
Cbren.WS247	Cbren.WS247_Fam_65_27_5	5	28	0.838095	AAcACCAGAntAGTTATAACATaGGGt
Cbren.WS247	Cbren.WS247_Fam_66_20_5	5	24	0.591667	aGCCAAAAATgggTTTTcaG
Cbren.WS247	Cbren.WS247_Fam_67_19_5	5	19	0.845614	GTGTCCTTTCAACAttTan
Cbren.WS247	Cbren.WS247_Fam_68_11_5	5	11	0.903030	TCGAtAAATTa
Cbren.WS247	Cbren.WS247_Fam_69_84_4	4	86	0.886305	GTTTTGTCTGGTTCTTGGCTaTaGATTGCTTGTCaATCcGTtTGTCTCCCAGaGAACTACAGCTTATCCAAcCCCGGAGaGGTT
Cbren.WS247	Cbren.WS247_Fam_70_75_4	4	75	0.925926	tCCACGATTGCGTTTCCAACGTaGACTTTTaACTGATTCACcTCAGTTGAgAcTTTGCCTGGAAaGGAGCGATCA
Cbren.WS247	Cbren.WS247_Fam_71_51_4	4	52	0.820513	AAccGACGTAGCTTCcGAAGAAGCAGATcAGTCCTAaAgGGgtaAAGTtAT
Cbren.WS247	Cbren.WS247_Fam_72_30_4	4	32	0.664062	ccTAATACCTTTTTgGGTATcAgcgagtCt
Cbren.WS247	Cbren.WS247_Fam_73_30_4	4	31	0.767921	GTtCTTAGaTCcAAATTTAGAgtTAgtGGA
Cbren.WS247	Cbren.WS247_Fam_74_28_4	4	28	0.849206	gGATGTCTGTTAGaATATCTgAaTaTCC
Cbren.WS247	Cbren.WS247_Fam_75_27_4	4	27	0.818930	GGACCAcccGGAccACCAGGACcACCA
Cbren.WS247	Cbren.WS247_Fam_76_25_4	4	26	0.655983	TgGTCCGGtCcgAtCCgGtCtGgcC
Cbren.WS247	Cbren.WS247_Fam_77_23_4	4	24	0.856481	aAaCCATGACCTTTTTGTTTCTa
Cbren.WS247	Cbren.WS247_Fam_78_21_4	4	23	0.589372	TTAGGCTTAGGtgaccAcAcc
Cbren.WS247	Cbren.WS247_Fam_79_19_4	4	22	0.622475	acCCGGGACCtAaAAAaAT
Cbren.WS247	Cbren.WS247_Fam_80_21_4	4	21	0.669312	tGGTGtCtGGCGCcAgAGggt
Cbren.WS247	Cbren.WS247_Fam_81_20_4	4	21	0.701058	GGTCcgaTCCGGcCTgGCCt
Cbren.WS247	Cbren.WS247_Fam_82_20_4	4	20	0.844444	tAGCATAGGGCCaAAATaTa
Cbren.WS247	Cbren.WS247_Fam_83_18_4	4	19	0.769006	TGGCGCTTAGgAtagATT
Cbren.WS247	Cbren.WS247_Fam_84_14_4	4	18	0.708333	TGTAGTTTGTAGTT
Cbren.WS247	Cbren.WS247_Fam_85_14_4	4	17	0.549020	GCTAcAGTAacCct
Cbren.WS247	Cbren.WS247_Fam_86_16_4	4	16	0.875000	ACCATGAcCTTTtTtA
Cbren.WS247	Cbren.WS247_Fam_87_11_4	4	11	1.000000	ACTCATGGTGG
Cbren.WS247	Cbren.WS247_Fam_88_10_4	4	10	0.866667	CGATTtGGgC
Cbren.WS247	Cbren.WS247_Fam_89_10_4	4	10	0.777778	GaCaGTTtCA

Cbren.WS247 Cbren.WS247_Fam_90_170_3 3 170 0.937255
TCTCCAATCTTTTCGTGaGATTTTTTGtATaCTCACTCAcAAGACCATAATTCTGCTCATACTACCTGTTTTtCTCCTaTTTTTTGCcTTTggAGATGTTCCAGTATTTCATGGGTGAAGTGTGTGGGAGaGCATTACTAACCATCATTTTCGACATTTT
CGAATGAAat
Cbren.WS247 Cbren.WS247_Fam_91_160_3 3 160 0.955556
ATGGACaTCGGCTCTATTccTTCTAGGACATTAaAGAGCATAATTTTCCAAGTCgcAATCCAAAATTTCATGAACCTACGTGCAGTTTCAGcATCGGCCGAAACGAATTTGTCTGTAACAGaCGTCAAAACCAACTGAGAAAAATTCAAATATTGAAAAC
Cbren.WS247 Cbren.WS247_Fam_92_144_3 3 148 0.713964
tcCatAanTcAAAAatcAAAAATcTTcAaAACCTGGTCATGATTATACTTTTtTGAAcCGTCTCGAaaTGCTGAaTTCAAAgATACCaaTTTcAtaATTTTagGTTCAAAACTagCcGaAAaACGTngAAAttCgAaTTTTaAt
Cbren.WS247 Cbren.WS247_Fam_93_77_3 3 77 0.763348
AAGCCAAAaGTAGAAcCTGGAAAAgCAACCGcgnnggggggGGgGGGTGGTCACcCTTTCCtGatttACTCTCGACCG
Cbren.WS247 Cbren.WS247_Fam_94_69_3 3 70 0.785714 CCTCTACAAAATgcCTGCCGCccctGcGCgACAGTTTGTAGAGGcTAAgGCtACAAACTACaAAccTTG
Cbren.WS247 Cbren.WS247_Fam_95_36_3 3 36 0.888889 GACTCGGTcGAGGTGGATTCCGTTGAtGTaCTGGTn
Cbren.WS247 Cbren.WS247_Fam_96_32_3 3 32 0.888889 ATAGCAGTTGaTcCATAGCAGAAgAGCCAAAt
Cbren.WS247 Cbren.WS247_Fam_97_29_3 3 30 0.855556 GACATTGgCCcTGAATTCAAATAGAAATt
Cbren.WS247 Cbren.WS247_Fam_98_30_3 3 30 0.881481 AAaTCTATGCGCTTTAGAacCACAAACtCA
Cbren.WS247 Cbren.WS247_Fam_99_27_3 3 27 0.934156 AaAGACTACAAAgACTACAAAGACTAC
Cbren.WS247 Cbren.WS247_Fam_100_27_3 3 27 0.868313 AAAATTCtGaAAATTCTGaAAATTCcG
Cbren.WS247 Cbren.WS247_Fam_101_22_3 3 23 0.753623 TGTAGCTACAtGnaGAGgGAAA
Cbren.WS247 Cbren.WS247_Fam_102_23_3 3 23 0.806763 ATTGGgCAACTTaCCTAGcTcaG
Cbren.WS247 Cbren.WS247_Fam_103_22_3 3 22 0.737374 AcCtGTCCTGgAGAGGacACCa
Cbren.WS247 Cbren.WS247_Fam_104_21_3 3 22 0.727273 AGCCTGAagGGCCTcAgTgCC
Cbren.WS247 Cbren.WS247_Fam_105_20_3 3 20 0.600000 GTcTGTctgTCtgTgTacCT
Cbren.WS247 Cbren.WS247_Fam_106_20_3 3 20 0.711111 GGCAanTTGCCaGagAAAaC
Cbren.WS247 Cbren.WS247_Fam_107_20_3 3 20 0.755556 AAGCcGCTTCtAgAtGnTCT
Cbren.WS247 Cbren.WS247_Fam_108_20_3 3 20 0.733333 AcAAATTTcaGATtTGAggC
Cbren.WS247 Cbren.WS247_Fam_109_16_3 3 16 1.000000 GTAGTGACGTAGGGAC
Cbren.WS247 Cbren.WS247_Fam_110_16_3 3 16 0.777778 TTCTATTTcGacaCAG
Cbren.WS247 Cbren.WS247_Fam_111_15_3 3 15 0.644444 TtcTaaTtCGGCCgG
Cbren.WS247 Cbren.WS247_Fam_112_14_3 3 14 0.873016 ATCGGAAtTGGTgA
Cbren.WS247 Cbren.WS247_Fam_113_11_3 3 11 0.838384 AAAtCGTCTAt
Cbren.WS247 Cbren.WS247_Fam_114_199_2 2 199 0.789782
CAGAGATCAGTATGGTCaAATCaAACAAACATAaAAACAaAGaATCAAATTAAAATAgaGaAGGaGTGAGAcaTAcGAATGGATTTCaCTaGAACTTGcaGGcAATTTTAAaAAGAAACTCTATcCATCTGTaTGcTCcAAaAGACCaCTGAAACCGTcG
AATGGACCTCCGAAGAaGCGCAGAcCCCCGAAGGaaAag
Cbren.WS247 Cbren.WS247_Fam_115_192_2 2 192 0.618056
TTGTCAACTAATaAAaTGcTaAGtAgATaAaaTTGTActCAaTTTGTcAGTTGAtCAaTTTTTcTGTATCTaTcAaGGAAACcGAGATaTaGAaCATCAATcCTTCCgTaTCGCTaTccaggCaGCAAGTGCGCTCTGcTGctTTTcTgTTcAGAcGcTc
aTATCTCaaTcAGGAAaaACGACATCaaAAAG
Cbren.WS247 Cbren.WS247_Fam_116_174_2 2 174 0.674330
cTTCGAcTGTTgTCtTCcGCTCATGCTATATAGACCTGGACATCAAtGaGAGcATTCgTaacCAGCCCCTcgTaaGAACTccgGTTCTcgCCCGTTGGcGGTTTATTCTCGTTCAGGaTCTAACAGCTTCtAGaTGaATCAcAAAGaagaTaTGcTTcAC
TGTcTGATcTCgAa
Cbren.WS247 Cbren.WS247_Fam_117_134_2 2 134 0.900497
TTTTTGCTTTTTTTCTTTTcGCAGAAAATGATTCAAAAAGTGTTcCTaTgTGTTcccATaGCTCAGTGGCTAAAAGCATTGGCAATTaTTTGAACCTAACAGTCGCAAGTTCAAACCcCACCGGTGGCAAATCT
Cbren.WS247 Cbren.WS247_Fam_118_128_2 2 128 0.787760
ATAGTCGGCGAGCGCCTGGAGCATTcCaTcCGACaTaATAcTCcGAACCTCATCCGGcCTCGCCGGCTATGACACaTAaGCaTAGgTGGACCATcCAAGAaCATAGGATCCAACcAcGaTAaGTGtGa
Cbren.WS247 Cbren.WS247_Fam_119_121_2 2 121 0.867769
TATAGCCGATTTCAGTTAACTATCCGATCATTCTTCGGCTACTcCAAcTTATTcTaAAAaTGAATCATgGCAGCACaCTaCAGGTTGTATaTaGGAAAATTTCACCGTTaTTTAAGAATTT
Cbren.WS247 Cbren.WS247_Fam_120_104_2 2 104 0.963141
tTTGGATGCCCAACCACCGCGATCTGTCAGGCAACGTGCTTCGGAAGGTCACTGGATCTATCTTGTCAGAGATCCTTGAcCGAAAGAGCACAcGTGCGGACCGG
Cbren.WS247 Cbren.WS247_Fam_121_95_2 2 95 0.663158
ATTGATCAACTgaCTCCAaTTaCTGAACaAgTCAaacaGGAaGcGAgACATCCaAACaTGTTGAaGAgATaCcAACaACTTCAAGCCAaGATaTG
Cbren.WS247 Cbren.WS247_Fam_122_90_2 2 90 0.888889
TGCTTCCAGCGTGCTGAGCTTgGGGGGAAaGTgTAGTCAGTTGGCTCCCGGGATTTTACTAAGCCTaTTTCTTAAAGGGTGTATCCcaCa
Cbren.WS247 Cbren.WS247_Fam_123_87_2 2 87 0.931035
GCCAGAAAAAACAACTGTGGAGGGGaGaGGGaTAGTTGTACTTTTCTaGAGACTTTTTCGATCGAAATACATCAATCTAGAGTCCGA
Cbren.WS247 Cbren.WS247_Fam_124_65_2 2 65 0.753846 AGTCGCAGCTATaCTcTACCCCGcCTgCacGGCGAAGTGGTaACACCCCgaGGTTCGAAcCcACa
Cbren.WS247 Cbren.WS247_Fam_125_54_2 2 54 0.901235 TTCaGCTCAGTGcTgGTGGTTCCCTCCGaTGTTGTCGTTGGGACGGTAGACGTC
Cbren.WS247 Cbren.WS247_Fam_126_45_2 2 45 0.659259 GCATAAGGCGaCGcCCTaAAaTTaaGAcGGGACTTTagGACAcTc
Cbren.WS247 Cbren.WS247_Fam_127_39_2 2 39 1.000000 TCTGTTCCTCTTAAAGGATCTGACTACTTTAGAATTTTG
Cbren.WS247 Cbren.WS247_Fam_128_36_2 2 36 0.629630 GGAcaacCaGGAcCACCAGGACCACaAGGAGacGaC

Cbren.WS247	Cbren.WS247_Fam_129_32_2	2	32	0.833333	GCACTTgCAGATgTCaAGaTTATCAATAGAGC
Cbren.WS247	Cbren.WS247_Fam_130_32_2	2	32	0.958333	AAAACGaTTTTCCAGGAAAATCATTTTCTCGG
Cbren.WS247	Cbren.WS247_Fam_131_31_2	2	31	0.811828	aGACTACAAACTACAAAAACTGAGaCTaaGA
Cbren.WS247	Cbren.WS247_Fam_132_30_2	2	30	0.622222	GCCaATTTaAGCCgAacgTaTTCAATTcGG
Cbren.WS247	Cbren.WS247_Fam_133_30_2	2	30	0.600000	TTGTTGGaACcGTcGTaGTTTcAacgGTaG
Cbren.WS247	Cbren.WS247_Fam_134_30_2	2	30	0.733333	CCTGCaCCAGCaCCAGCaGCaGAaGAGACa
Cbren.WS247	Cbren.WS247_Fam_135_29_2	2	29	0.862069	AGAGTCACCAGAGTCACCcCaGAGTCAgT
Cbren.WS247	Cbren.WS247_Fam_136_29_2	2	29	0.770115	CGGGCCAATTcGGGCCATTTTaaaCCAAc
Cbren.WS247	Cbren.WS247_Fam_137_29_2	2	29	0.724138	TTTCTAGGCCAcCaAATCAAaAaTTTgaG
Cbren.WS247	Cbren.WS247_Fam_138_28_2	2	28	0.761905	TTGCgCGTAAcTTaCGAAATaTTgGAAT
Cbren.WS247	Cbren.WS247_Fam_139_27_2	2	27	0.802469	CTTCCGGGCCTTCcGGGCcTTCcGcGC
Cbren.WS247	Cbren.WS247_Fam_140_26_2	2	26	0.602564	AcCTACAGTAATCCtAAcTgTaGGtg
Cbren.WS247	Cbren.WS247_Fam_141_25_2	2	25	0.633333	CCGGATGTCCAgGaATCcGaATgTc
Cbren.WS247	Cbren.WS247_Fam_142_24_2	2	24	0.618056	GGCAAcTTGCCGaaGGTcTaGaTg
Cbren.WS247	Cbren.WS247_Fam_143_24_2	2	24	1.000000	CTTGGCTTCAGTTTCAGCCTTGGT
Cbren.WS247	Cbren.WS247_Fam_144_24_2	2	24	0.777778	TCCTTCTTGGCCTTggCaTCAGCc
Cbren.WS247	Cbren.WS247_Fam_145_24_2	2	24	0.722222	CATAcATCCTaTTTTCaGTccTGC
Cbren.WS247	Cbren.WS247_Fam_146_24_2	2	24	1.000000	CACCGGACTTACAGCCGTACTTAC
Cbren.WS247	Cbren.WS247_Fam_147_24_2	2	24	0.944444	TACACACTTTTTATTCAaGAGGAC
Cbren.WS247	Cbren.WS247_Fam_148_23_2	2	23	0.652174	AAAATCtgGAaTTTTTCgGTaTG
Cbren.WS247	Cbren.WS247_Fam_149_23_2	2	23	0.884058	AAGCCTAAAAAGGCCTAGTcTaC
Cbren.WS247	Cbren.WS247_Fam_150_23_2	2	23	1.000000	GCGCTACGATGCTCAACAACGAC
Cbren.WS247	Cbren.WS247_Fam_151_22_2	2	22	0.613636	GTTcTAgGGCaTTTCtAGAGCt
Cbren.WS247	Cbren.WS247_Fam_152_22_2	2	22	0.666667	TTaGCgTcAAAAcTCaCCTATT
Cbren.WS247	Cbren.WS247_Fam_153_21_2	2	21	0.619048	AAtAcTATGAGttaTTTCCGA
Cbren.WS247	Cbren.WS247_Fam_154_21_2	2	21	0.650794	GaTaTGAGcCTCAGGCTTaCa
Cbren.WS247	Cbren.WS247_Fam_155_21_2	2	21	0.746032	GTAGATCAAAACaTAgGCTaG
Cbren.WS247	Cbren.WS247_Fam_156_21_2	2	21	0.658730	GGaGcAAAAAATaGAaTTTCc
Cbren.WS247	Cbren.WS247_Fam_157_21_2	2	21	0.619048	gAaGCTGaTCTaAGGCTGgCT
Cbren.WS247	Cbren.WS247_Fam_158_21_2	2	21	0.809524	TCAACTGCTATAGcTGTTGca
Cbren.WS247	Cbren.WS247_Fam_159_21_2	2	21	0.873016	GCCCCCCACCATCCAAcATCa
Cbren.WS247	Cbren.WS247_Fam_160_21_2	2	21	0.873016	AAAAATGAACGGGTCaAAaAA
Cbren.WS247	Cbren.WS247_Fam_161_21_2	2	21	0.619048	TAAAAaGGATAAAaaGcGaGa
Cbren.WS247	Cbren.WS247_Fam_162_21_2	2	21	0.619048	GGACTAGaaTTcCaGAacGAT
Cbren.WS247	Cbren.WS247_Fam_163_20_2	2	20	0.633333	GGCCcaAAACGGcCTgTAaC
Cbren.WS247	Cbren.WS247_Fam_164_20_2	2	20	0.800000	TCaACTGCTATTCCtTAGAC
Cbren.WS247	Cbren.WS247_Fam_165_20_2	2	20	0.666667	CCAAaTGaGcCGATTcTGaA
Cbren.WS247	Cbren.WS247_Fam_166_20_2	2	20	0.666667	CTCAacTaCTATaTTTTTGa
Cbren.WS247	Cbren.WS247_Fam_167_20_2	2	20	0.933333	GCCTGACAGACCTGAGcACA
Cbren.WS247	Cbren.WS247_Fam_168_20_2	2	20	1.000000	GCTCACTGCCGCCCCGTTGC
Cbren.WS247	Cbren.WS247_Fam_169_20_2	2	20	1.000000	ATGACCGAGTGTTTGACCGA
Cbren.WS247	Cbren.WS247_Fam_170_20_2	2	20	0.866667	TTCTTGTGATaAAcCACAGG
Cbren.WS247	Cbren.WS247_Fam_171_19_2	2	19	0.666667	TGTCaATTTcTcCATCTag
Cbren.WS247	Cbren.WS247_Fam_172_19_2	2	19	0.649123	TTTCcAGcCCAGATaTGaa
Cbren.WS247	Cbren.WS247_Fam_173_18_2	2	18	0.666667	TaGGCTTgaAGGGaGCCT
Cbren.WS247	Cbren.WS247_Fam_174_18_2	2	18	1.000000	ATGCCACACCATCAATGG
Cbren.WS247	Cbren.WS247_Fam_175_18_2	2	18	0.703704	ATACTaCTcCgGcACCAA
Cbren.WS247	Cbren.WS247_Fam_176_18_2	2	18	0.777778	AGGCTAGTCAGGcTaaTC
Cbren.WS247	Cbren.WS247_Fam_177_17_2	2	17	0.921569	ATTGACAAAGTTGGGTa
Cbren.WS247	Cbren.WS247_Fam_178_16_2	2	16	0.635417	TCCGGtTATGaAAcGa
Cbren.WS247	Cbren.WS247_Fam_179_16_2	2	16	0.750000	CCcCAGGACCCcAGaA
Cbren.WS247	Cbren.WS247_Fam_180_14_2	2	14	0.690476	ctCGCAACtCGCAA
Cbren.WS247	Cbren.WS247_Fam_181_14_2	2	14	0.809524	TCaATAaTCAATAA
Cbren.WS247	Cbren.WS247_Fam_182_13_2	2	13	0.692308	TaaAAGTTAAAcG
Cbren.WS247	Cbren.WS247_Fam_183_12_2	2	12	0.833333	TgTTTGACCGAG
Cbren.WS247	Cbren.WS247_Fam_184_12_2	2	12	0.611111	aCATTTTCgCaA
Cbren.WS247	Cbren.WS247_Fam_185_12_2	2	12	0.777778	TAGGTaTAGGTa

Cbren.WS247 Cbren.WS247_Fam_186_11_2 2 11 0.757576 CaTcGCCTAAC
Cbren.WS247 Cbren.WS247_Fam_187_11_2 2 11 0.636364 AAAaAGGCcCa
Cbren.WS247 Cbren.WS247_Fam_188_10_2 2 10 0.733333 CTTCcACGGc
Cbren.WS247 Cbren.WS247_Fam_189_10_2 2 10 0.600000 CCCcGGaAaA
Cbren.WS247 Cbren.WS247_Fam_190_10_2 2 10 1.000000 TTTTTGAAAT
Cbren.WS247 Cbren.WS247_Fam_191_231_1 1 231 0.000000
ACACACCGTGCTGAAAACCTTCAAGATGTTCCACTTTCTCATTCTGTCACATCTTATCCATCTGAATTCAGCTACGAGAAACTGCCAGAGCACGTTGTTCCAGTTGTTGTGGAGGAAACCGAGAAGAAGGATGAGCCATCATTGAAATCCAAGATTACCG
GACTCTTCAAGAAGTCATCTGCTCATGCTGACTATCCAATCTCTGAACAATACGATGGACCATTGGAATCA
Cbren.WS247 Cbren.WS247_Fam_192_218_1 1 218 0.000000
GGACTTGAAATCAGGGAATTATTGATTTAGTTTTTAGAAGCTTCTCAATTTTAACAGAATGATCAGATCAAATGATTCGTATAGACAGTGCATGTAAAGACGCTGGTCGTTGCTCAAAAAGCCCCGGCCCCCCGTCGGGAAGAGACAGAATTCACATACA
ACTGACTAATAAACCGCTCAATACAGACCGCCCCATTAAAATTATAAAAGGATGAAAG
Cbren.WS247 Cbren.WS247_Fam_193_180_1 1 180 0.000000
TTTTTCTTAAAATGAAGCAATCGCTAAGATAATTCGGGGATATTTTCCCCAGTGGCTGTAGTGTCAAAAAAACATAATTTGAGCCCTGAGAAATGCCTAGTTAGGGCTGTGCAGCCAGGAATGCAAAAACGAAAATCCCAGTAGAAGTGGGATATAGGGT
AAAATTTTGATGAATCACAA
Cbren.WS247 Cbren.WS247_Fam_194_176_1 1 176 0.000000
TATAAACCTGCCTCTCATACCTAAAATTTGGCGGATTGGCAATAAGAATTGTATGTTTTTACTATCGGCATAATTATTACAACGATTTGACTAAGTTTCAGAAAACTTCAGCAAAGAATTGGAAAATTTTTTGGTTTAGGAGGCGACATTTTTGTCGAAA
ATTGGACAAATGTGAA
Cbren.WS247 Cbren.WS247_Fam_195_172_1 1 172 0.000000
GCTATCTCACAGGTAAAATCAATTTTTATGCATATAAGTTGCCATTCCAACAGCTCGTCTGCCCAAGGAATTCTTACAATCCGCCTCAGAATAGTTTCAAGAACCCTCAAAAAAGCCAAAAAGTGATGGTCACGGAATGGCGATTCCGAGTGTTTCATGT
TAAATAAAACAG
Cbren.WS247 Cbren.WS247_Fam_196_171_1 1 171 0.000000
AGAGCGAGATTTGTGAGAAATTCCGAGAAATGAGCCTTCGAAACACGTATACCTGATTTTTGAGAAAATTTCGCAAAAACTGAATTTTGTTCGTTCAATATAGTTTAATCGGTATTGATCTGTTTCGATAACGCTAACGTTGGCTTCTCCGATCGTGTTC
GAACTGAATCG
Cbren.WS247 Cbren.WS247_Fam_197_170_1 1 170 0.000000
TGAAACTGAACAAATGATTGATACTAGGAGCAGAACAAAGATCTCAATAACAATACTATTTCAAAAAACTCCCAAAAATTTAGAATTTTAGACCCTAAAATGACCGACGTCTGAGCATTCAACATCTCTTCAACGCATTGTCCAACAGTGCTACTTGGAG
CTTGAAATGC
Cbren.WS247 Cbren.WS247_Fam_198_168_1 1 168 0.000000
GTACTCTCACTTCTTCTGAAGTCGTCAAATAACATCCAGAACTGAACAGTTGCTCTAAATTGTTTGGTAATCACCTGATTCTCTCAACAAAGCCGTATCAGAACGGCATCGTGTGGTTTTCCAAGAAAAAGTTCTTGAGGTGTATATTTACTGATGATCC
TCCCTCAA
Cbren.WS247 Cbren.WS247_Fam_199_163_1 1 163 0.000000
TAGCATGAGCGGAAGGGACAATCAGAGATTAACGAATACTGAAACACATCTTCTTTGTGATTCAGATGAAGCTGTTAGCACTCGAACGAAAATAGACCCCCACCGTATATGACCGAAGTCAAAGGAAGTGATACGAATATTCTCTTTCTTGTTCGAGTCT
TTA
Cbren.WS247 Cbren.WS247_Fam_200_162_1 1 162 0.000000
TTTCGCTCATGAAAAACTAAAATGGTCCCGTATCGGCTGGAATTTTCATCTGAATTTATGTTTTGGGGTTGGTTGTCGGCTTCTGATAAGCAACTAATATATTTCGGCCTAAAATCTAAACATTGTCATTTCAGAAAAAAGTTTTGAACCGTCCATCAAT
AT
Cbren.WS247 Cbren.WS247_Fam_201_162_1 1 162 0.000000
CCATCAATTGCCCGAAAAATCGAATCGGAAATTCAAAGTATTTGAACATTTTTCGAATTTTTCTGGAAATCTGAAAAAAACTGATATGGCTGAAACTTGAATCAATGTCACTAGAACTTATTACACTGATACTTAATACAACTATTTTGAGTGGATACAA
AG
Cbren.WS247 Cbren.WS247_Fam_202_160_1 1 160 0.000000
TCTAACAGCTTTCCATGAATCACAAATCTCTGTGCTTCACTATTCGGTGTCATCCTTCGAATGTTCCTTCCGCTCATGCTAAATAGACCTGGATATAAGAGTATTCGTACTGCCTCATTGTGATCGGGCACGCGGTTAGATGGTTTACTCTTGTCCGGGC
Cbren.WS247 Cbren.WS247_Fam_203_160_1 1 160 0.000000
TAGTATGTTCAGTTACAATTCCAAGATGCAAATCGAGGTTCAAAAAGTTTCTCCGTTTTTCTTGATTTCACAAATGTTGAACTTGATCATGACTAAAAATACGTATAGACTGAAAGATTTTCTCTGAAAGCTCAAAGAAACGTAGTTTTAGACACATTTA
Cbren.WS247 Cbren.WS247_Fam_204_151_1 1 151 0.000000
GTGATGGTCCAGTAGGAGAAAGTGTTCTTCTCCTTCTAATCGAACTGTTTTTTTGATCTACTGAGAAGAGAACGAAAGAAAAGGTGGACGAGAGGAAGAGTATAAAATTGGAAGTTTGGACGGAAACAGAAAAAAAAATCAATAAAAAGAG
Cbren.WS247 Cbren.WS247_Fam_205_150_1 1 150 0.000000
ATCTACAGTAACCCAAAAACCAAAAAATCTTTGAAAGCTGCAGAGATTTCTCAAATTTGATACATCTAAAATCGAGGATTTCATGTTCGTTAGTTTCATATAGATCACAAACAAAATCTAAAAGATAGGACCTAAACGAGCTATCCTGTC
Cbren.WS247 Cbren.WS247_Fam_206_146_1 1 146 0.000000
AAAAAAATTCTAATTTTTTGTGCAAAGGATAGCAAATGTGCTGAGGAAGCTTAGAACGAAAAAATTTTTTGAAAAATCTGTCTCTGAACCCTATTATAAGGGCATCCTCTATTAGAAGGGCACCCACCGGTTTCAGGACCGCCCCC
Cbren.WS247 Cbren.WS247_Fam_207_144_1 1 144 0.000000
GGGTCTCGCCACGAAAATCTAAAAACTGTCATATCTCAGTCAGTTTTGGTCAGATTTAGATGAAAACAGTACCGTTGGAGTTAGGCTGAAGAGACGCTTCGAATGAGTATAACGATGACTAGGTTTTGACATATTTATATTTTC
Cbren.WS247 Cbren.WS247_Fam_208_143_1 1 143 0.000000
GGGTTACGGTAGTTTTTGTGATTTCGTGACGGACCCAAAAATTAAAAAAATTCCAAACCCGACCTTGTGGGTAGTTTTCTGTGCCGCCTTCACGTGCTGATTCCAAATCTATCAAAACTATCGAGATCGAGTGAAAATTGATC
Cbren.WS247 Cbren.WS247_Fam_209_140_1 1 140 0.000000
TGAGAAGATGTTGGATACGGAAAAAACAGTGCATTTCAGGTCGACAATGCGGAAATCTCTCAAATTTCCTATGTTTCATTCTCTCAGTAGACCATTAACTTGCGCTCAATTGTTTTCTCTTGTTCACCTAGAGAACGCAT
Cbren.WS247 Cbren.WS247_Fam_210_138_1 1 138 0.000000
GTTCAGAAAAGTACCCACAAGACGAGGTTTCAAATGTATCTGTTATAATCACTCATTTTGTGACTTCTAATAATTATTTATGGAATTCAAAAAAACCATGTCTTGTAGGTTATCATTAGAACCGTATCGACGTGCCGT

```
Cbren.WS247	Cbren.WS247_Fam_211_138_1	1	138	0.000000
CTAGGCCACGGCCGCGAAAAGTTAGGCCACCAATCTTCGAAATTGCACAGTTTGGGTGAAAATTGGGCTGGCGACTCGATTTTTGAACAGTTTCGGCCGGTTTGACCTGGGCTTTCAGGAAAAAACAAACATGGAATT
Cbren.WS247	Cbren.WS247_Fam_212_131_1	1	131	0.000000
AGCCACGTGCCGTCAAGTTATCAAGCCTCCCGGAAAGGCTGATACCTTGACAACCAACCCATCTAGTAGCATTCGATGAGTCCGCGACTATCTTGTTCTTACCTCAGAACTTTTGGCATAGATACACAGAC
Cbren.WS247	Cbren.WS247_Fam_213_122_1	1	122	0.000000
AGTTCGGGGGTAGCCAGCAAATCTATAGGAAGATCAACCAGCCCACTAGGTACCCGCATCTTGACTGTCCAAAAGGGTGAGAGGGGGGAAAAAGTGTTCTAAGTTACTAGAGAAGTGACAGC
Cbren.WS247	Cbren.WS247_Fam_214_120_1	1	120	0.000000
GATCCAAGAGTAGAGTAAGAAGCTGGTGAATTGCTCGATACCGTTGTTTCTTGTTCAGTAGTACATGGCTCGGTGGTAGTCTCTTCTGAAGTTGCCGTACTCGATGAAGAGGCATCAGTT
Cbren.WS247	Cbren.WS247_Fam_215_120_1	1	120	0.000000
TGTTCAGATGAACCTTCAATAGGCTCGAGAAGTGGGGATGACCGTTCGTAAGTGACTTCATGACCAAACGACTGTTCGAAGTCAGCTCCTGATGTTGCACTTGACCGATCTTCTTCTAGA
Cbren.WS247	Cbren.WS247_Fam_216_120_1	1	120	0.000000
CTGACTTTTTCCCCTAATTTTAATAAATTGGTGGTTCGAAAGTAGATGCTTCTGTGAGTGGATTGTGAAGTTTCCATCCCTCACTAGAGCCTAAGAGTCTGAAAATCTTCATCTGAAAAG
Cbren.WS247	Cbren.WS247_Fam_217_119_1	1	119	0.000000
TTTACTGGATTTTAGGAATGTCTAGCCGGAATTCAGCTCAATTTCGATCCAATTCCAACTATTTCAACCTTTCCTTCGATCAAAACATGCTGGGTCACTAATGTTGTCTCTAGGCAAAA
Cbren.WS247	Cbren.WS247_Fam_218_117_1	1	117	0.000000
GTAATTTTTTTGGTGTTTTATGGTTATTTTATAAAAACGTGCAAGATCATATGAGACAGTTCCGAGTACCAATCAAAAATCTGCGAAATTAAAGGAGACTTCGTTCTTTTTGAAACT
Cbren.WS247	Cbren.WS247_Fam_219_116_1	1	116	0.000000
TAGCAGAAGAAGGACAGACGAAGGCTGTTAGAACCCGAACGAAAGCGACCATCCACCGTGCATAACCGTAGTTAAATATGAGTGGTCCGAACACTTTCATTATGCTCAGGTCTTTA
Cbren.WS247	Cbren.WS247_Fam_220_110_1	1	110	0.000000
TCTCACTAAAAAGTAAGATTATAGGCTACCAAAATATGATTTCTGAACAGAATCACTTCGAGTTCTTTGGTGGTCATGGGTGGATTAGTCCCAGGACATACCTATAATTT
Cbren.WS247	Cbren.WS247_Fam_221_109_1	1	109	0.000000
TGGTGGCCTAGAACTATATCTGGCGGACATCTAAAATCAAGAAAATCAAAATGAGCCTCAACCCAAGATTCTAGCTCAACTTTGAAACGATTTACGACGTTTTGAAGTT
Cbren.WS247	Cbren.WS247_Fam_222_106_1	1	106	0.000000
TGCTAAATAGACCTGATAGGAGTATTCGTGCTGCTGCCCTGGATTTCGGTCCTATGCGGTAGAGGTCTATTCTTTTGTGGGTTCTAACAGCTTCCGCTTCTTCCTA
Cbren.WS247	Cbren.WS247_Fam_223_96_1	1	96	0.000000
TCGACCTGGCGGTTGAGCTGAGATCTACAACTGTACCTCATCCCCCCCCCCCTTTAGGGCGGTTGTATTCTCTTCTTGGCCATGGGTTGATGCATT
Cbren.WS247	Cbren.WS247_Fam_224_93_1	1	93	0.000000
TCAGATGATTCATCTGACGACGAAAAGAAACCAGCTATTAAGCCAACGCCGAAAGCAACTCCAGTCGCTGCCAAAAAGAAGGCCGAATCTTCC
Cbren.WS247	Cbren.WS247_Fam_225_90_1	1	90	0.000000
GCCGGTATGACACAATAGCAGAGCTAGATTAACTAGAAAAAGCGTATATATTGATTATATAGAAAAAGGGTCCAGACAATCACGAATGAT
Cbren.WS247	Cbren.WS247_Fam_226_90_1	1	90	0.000000
GTCTTCCACTGGAACTACTGGACAGTCGATGCATGAATTCGTGACTGGAAACTGGAATAGTGCTCCATCGATTGGTATGGACTTTGGAAT
Cbren.WS247	Cbren.WS247_Fam_227_86_1	1	86	0.000000
AATTTTTCAATCGAGAATACAGCACAAAATGATGAACTACAACGTATTGGATCAAATTATCACAAGTCTGGACCATTAGAAGCTCA
Cbren.WS247	Cbren.WS247_Fam_228_84_1	1	84	0.000000
CAGTCGAGGTGGACTCAGTCTGAGATGTGGTAGACTCAGGAGTAGTAGACGTCTGTTCAGTTGTCGAGGTTTCCGTAGTCGATT
Cbren.WS247	Cbren.WS247_Fam_229_83_1	1	83	0.000000
CGCATTGGTTTTTTCGAATGACGACAAATGGATTTGGCTAAAATCTTCGCCACATCCATCGCTATTCGACAAAATATCGTACC
Cbren.WS247	Cbren.WS247_Fam_230_81_1	1	81	0.000000
TCACAAGGCTCTGTCGTAGTCTCGCAAGGTTCCGTGGTAGTCTCGGACAACGTGGAGTTCAGGCATGGCTCCGTTGATGTT
Cbren.WS247	Cbren.WS247_Fam_231_81_1	1	81	0.000000
ACTGGAACTCCAGTTACCGACGAAACAACATTGTCAACCGATGAAACTCCATATGGCACAAGATCCACAGCCACTGCTCCA
Cbren.WS247	Cbren.WS247_Fam_232_74_1	1	74	0.000000
GCCTCTACAAAGTCATCGGCAGGCAAAAAAGATGCAGGAGAGTTTGTAGAGGCCAAGACTACAAACTAAAAGCT
Cbren.WS247	Cbren.WS247_Fam_233_74_1	1	74	0.000000
CATCTTGACTAGGAAGCCGCCTTAGCTGCTAAGACACCATCATATTTAGGGCGTCGCCTTAATTGTTAAGACAT
Cbren.WS247	Cbren.WS247_Fam_234_74_1	1	74	0.000000
TAGGCTCCGCCCATTTTTTTTTTCAATATTTCAGTTTTGGGGCTGAAAATCAGTGTCTAAGGGTTGGGGGTAAC
Cbren.WS247	Cbren.WS247_Fam_235_74_1	1	74	0.000000
GTACGCAAACACACTTTACACGAAATTGCAGAAAATTTGGTTTTGGTACGAATTGTCGTGGGATCTAGCGCCGG
Cbren.WS247	Cbren.WS247_Fam_236_72_1	1	72	0.000000	TTTCAGCCTTGGTCTTGGCTTCGGCGTCCTTCTTGGTCTTTTCATCAGCCTCCTTCATGGCCTTGGCTTCAG
Cbren.WS247	Cbren.WS247_Fam_237_69_1	1	69	0.000000	CAGGTTCTGGAGTAGATTGTTGAGTCTTGGACATGCTTTCTGTTGAACTCTTCCAACCAATTCGATGAA
Cbren.WS247	Cbren.WS247_Fam_238_69_1	1	69	0.000000	TCAAATACTGTATTTCTCTGGAGGCAAGGATTCGATGACTTCATTCCGACCATATCCGAATTTGAATCA
Cbren.WS247	Cbren.WS247_Fam_239_69_1	1	69	0.000000	AGTTGGATACGAAACTGTCCAAAGATTGTGTCGTGAAAAGTTCACCGAGCCAAAGTCAAATTGGACCCA
Cbren.WS247	Cbren.WS247_Fam_240_69_1	1	69	0.000000	AAGTTCTCGAATCTGGCACTTGTGATCTGCTGACGAAGAAGACATCCACCACTTGCCTTCCGAAAGCAA
Cbren.WS247	Cbren.WS247_Fam_241_68_1	1	68	0.000000	CCCTGCCCCAGATTCTGTCTGGGATTATCCGGGGATTTACGCTGAAGACGAGAAACAACTATTCGGTG
Cbren.WS247	Cbren.WS247_Fam_242_63_1	1	63	0.000000	CCATCATCGGTGGATAACCCTGGAATGGTGGATTCTGAGCATCCTGACGTTGTTGCGGGGGCA
```

Cbren.WS247	Cbren.WS247_Fam_243_63_1	1	63	0.000000	GACGCAGAGAAACGAAAATGCCTACGGGTCTGCGCTCTCTACTAGTAGTGACAGCTATTGCGA
Cbren.WS247	Cbren.WS247_Fam_244_58_1	1	58	0.000000	CTAGGTCACCAAATCTAGAAAGTTAGGCCGATGGACTAATTTTCCAGGATTTGGATTT
Cbren.WS247	Cbren.WS247_Fam_245_57_1	1	57	0.000000	TAGACTCTGTCGATTGAGTACTGAAGGTTGAATCAGAGATTGTAGTATCAGTGAATG
Cbren.WS247	Cbren.WS247_Fam_246_56_1	1	56	0.000000	GGGAATTCAAAATTTCGAAACTTTTGGTGGAAATTTAAAACTTCGAAACTTTTGGC
Cbren.WS247	Cbren.WS247_Fam_247_55_1	1	55	0.000000	TCTGCGCTCTCCCATACTTACCGGAGAGAGCGCAGACAGCTAGACGCTCTGGCCG
Cbren.WS247	Cbren.WS247_Fam_248_55_1	1	55	0.000000	GATAATGAAACAAACATGCCTTTGATTATACTCATTCAAACCGTTATGATCAGGA
Cbren.WS247	Cbren.WS247_Fam_249_54_1	1	54	0.000000	TTTGGTGGTGATCCATCCAGATCGATAACCTCTCTTGGGTTGAGCTGGAGCAAG
Cbren.WS247	Cbren.WS247_Fam_250_51_1	1	51	0.000000	AAGAAGATCGGGTGGTCGACAAGGTCATCGGTTCGCTAGACTGGACTGGTG
Cbren.WS247	Cbren.WS247_Fam_251_49_1	1	49	0.000000	AAACTAGGCAATCAAACTAGTTTTGTGGTTTCTAGGCCATCGGTTGGAA
Cbren.WS247	Cbren.WS247_Fam_252_48_1	1	48	0.000000	ACACTCCTCGCCATCTCCAGCGAAGAACGAGTCCGACTGCTGGACGCG
Cbren.WS247	Cbren.WS247_Fam_253_48_1	1	48	0.000000	ATGGCTCGGGATGGAAGGAGGAGGACGACAACAACGACTGGATCATGG
Cbren.WS247	Cbren.WS247_Fam_254_48_1	1	48	0.000000	TCCTTTTTGGCCTTGGCCTCAGTATCAGCCTTGGTCTTTGCTTCAGCG
Cbren.WS247	Cbren.WS247_Fam_255_47_1	1	47	0.000000	GTTTCTCTGCGCTCTAACTCCATGACGCAGAGAAGTCAGACACTCTC
Cbren.WS247	Cbren.WS247_Fam_256_45_1	1	45	0.000000	GGACCACCAGGACCTCAAGGAGGACGTGGACAAGGCCCAGACTTC
Cbren.WS247	Cbren.WS247_Fam_257_45_1	1	45	0.000000	CCTGACCAGAACAGGCCAGGCAGGGCTGGTCCAGGCTAGTCCAGG
Cbren.WS247	Cbren.WS247_Fam_258_43_1	1	43	0.000000	TTTGGTTTTTAGGCCTAAACTTTCGAATTTTTGGGACTAAACT
Cbren.WS247	Cbren.WS247_Fam_259_41_1	1	41	0.000000	CTGACGATTGCTCAGTATTGCACCTAAAAACACTCGTTTTT
Cbren.WS247	Cbren.WS247_Fam_260_40_1	1	40	0.000000	TTTCCGTTTCCGTTTCCGGCGGAAATATTTTTCTGGAAAT
Cbren.WS247	Cbren.WS247_Fam_261_40_1	1	40	0.000000	GTAGTGACGTAGGGACGTAGGCACGTAGGCTCGTAGGCAT
Cbren.WS247	Cbren.WS247_Fam_262_40_1	1	40	0.000000	TGGGCGGGGCTTAGACTGCAACTCCGCTCCTTTTCGAATG
Cbren.WS247	Cbren.WS247_Fam_263_40_1	1	40	0.000000	GGATGTCCGGATGTCCAGGAATCTGTCTGTCTGTACTCCA
Cbren.WS247	Cbren.WS247_Fam_264_39_1	1	39	0.000000	GATGCTTCAGTGACGGCTGCGGTGGTGGTCTCTTCTCCT
Cbren.WS247	Cbren.WS247_Fam_265_39_1	1	39	0.000000	GATGTGCAGGTTCCTATCACTGCAGTAACTATATAGTTA
Cbren.WS247	Cbren.WS247_Fam_266_39_1	1	39	0.000000	TTCCAGAAAGTTCCAGAACCTCCAGGAAAGCTCCAAAAG
Cbren.WS247	Cbren.WS247_Fam_267_39_1	1	39	0.000000	TCCGGATATCCGGATATCAAGATAACAAAAGTCTGTGTG
Cbren.WS247	Cbren.WS247_Fam_268_39_1	1	39	0.000000	CAGGCCTATTCGGGCTGCTCCAGGACAATTCAAACTAAA
Cbren.WS247	Cbren.WS247_Fam_269_39_1	1	39	0.000000	GGTTACTAACCTGCAAGATTTTTTTCTGATTTTCTAGCA
Cbren.WS247	Cbren.WS247_Fam_270_37_1	1	37	0.000000	GACACCACCCTCTGGCGCCCGTCTCCACCCTCAGCGA
Cbren.WS247	Cbren.WS247_Fam_271_36_1	1	36	0.000000	CCGCCCACTTCAATCTGAATCGAAGTTTGTTAGGCT
Cbren.WS247	Cbren.WS247_Fam_272_36_1	1	36	0.000000	GCGCTCTATTGAGAATATTGGGAAATTCGAGAAAAT
Cbren.WS247	Cbren.WS247_Fam_273_36_1	1	36	0.000000	GTCCGGATGTCCTATTGCCTGTTTTTCTGTCTGTAT
Cbren.WS247	Cbren.WS247_Fam_274_36_1	1	36	0.000000	ATTGACAATTTCCAGAAAACCATGCAAAAGCGCTCT
Cbren.WS247	Cbren.WS247_Fam_275_36_1	1	36	0.000000	CCGAGCCGACTACCACCACTGTCCCGACGACTACTA
Cbren.WS247	Cbren.WS247_Fam_276_36_1	1	36	0.000000	TCCGGATGTCCGGATGTCTGTCTGTGGGTAGGTTTA
Cbren.WS247	Cbren.WS247_Fam_277_35_1	1	35	0.000000	TACTGTAGCTGGCTTCCTGCCTATCCTACCAGGAT
Cbren.WS247	Cbren.WS247_Fam_278_34_1	1	34	0.000000	TTTGTTCCCGGAACGAAAATATAGACTGCTAAAA
Cbren.WS247	Cbren.WS247_Fam_279_33_1	1	33	0.000000	CTATTTTCTCAAGCGCTACTGGGTGTTCGACTG
Cbren.WS247	Cbren.WS247_Fam_280_32_1	1	32	0.000000	ATGGTTTTTACAAATGAAAAAACATAAAACTC
Cbren.WS247	Cbren.WS247_Fam_281_32_1	1	32	0.000000	GGCCTAGGATTTTTCTAGGCCACCGGGACCAT
Cbren.WS247	Cbren.WS247_Fam_282_32_1	1	32	0.000000	GAAACCGCGACATTTCGTAACCGAGACATTCC
Cbren.WS247	Cbren.WS247_Fam_283_31_1	1	31	0.000000	AGGGTCTAGGGTCCAGGGTAGCCTAGGTCCC
Cbren.WS247	Cbren.WS247_Fam_284_31_1	1	31	0.000000	TTTATTCGAGTGCAAAGTGAGTTCATATATT
Cbren.WS247	Cbren.WS247_Fam_285_30_1	1	30	0.000000	TAAGCCTAAGATTATGAAGTAGTTCAAGCG
Cbren.WS247	Cbren.WS247_Fam_286_30_1	1	30	0.000000	GTCGTTGGAACAGTAGTGGTAGCCACAGTT
Cbren.WS247	Cbren.WS247_Fam_287_30_1	1	30	0.000000	CCGCTTGATGAAGAAGTTTCTGAGACTGAG
Cbren.WS247	Cbren.WS247_Fam_288_30_1	1	30	0.000000	CCTAAGCCTAAGCCTAAGCTTCAGTCGTGA
Cbren.WS247	Cbren.WS247_Fam_289_30_1	1	30	0.000000	TAAAAAGTGACTCCTCCATGTAAGTGACCA
Cbren.WS247	Cbren.WS247_Fam_290_30_1	1	30	0.000000	TTTGGGAATGATTAGGTGCCGAGGGAAAGT
Cbren.WS247	Cbren.WS247_Fam_291_30_1	1	30	0.000000	AAGTTGCCCAATGCTCGGCAAGCAAAAGAC
Cbren.WS247	Cbren.WS247_Fam_292_30_1	1	30	0.000000	TAAGCCTAAATCTATACCTAAGCCGGGGTC
Cbren.WS247	Cbren.WS247_Fam_293_29_1	1	29	0.000000	GCCTAAACAGCCTAAATCGGCCCAAATCG
Cbren.WS247	Cbren.WS247_Fam_294_29_1	1	29	0.000000	GCAGACCAGACGGTAAAAATTGGAGCTAA
Cbren.WS247	Cbren.WS247_Fam_295_29_1	1	29	0.000000	TTTGGGTCTGCTTATTATTTCCCATACCG
Cbren.WS247	Cbren.WS247_Fam_296_29_1	1	29	0.000000	TCAACTGCTATGAAATCTTCTGCTATATC
Cbren.WS247	Cbren.WS247_Fam_297_29_1	1	29	0.000000	TCTGGAAGTTCTGGAAGGTCCTAGAAGTT
Cbren.WS247	Cbren.WS247_Fam_298_29_1	1	29	0.000000	AAGTGCCGCTTCCAGGCGTCATGATCCAA
Cbren.WS247	Cbren.WS247_Fam_299_28_1	1	28	0.000000	TCAAATTCCTTGCTGGAACTTTAAAATT

Cbren.WS247	Cbren.WS247_Fam_300_28_1	1	28	0.000000	AAAAAGCTGAAATTTTGGAGGAAACTGG
Cbren.WS247	Cbren.WS247_Fam_301_28_1	1	28	0.000000	AGAAAAAAAGAGAATCAAATTAAAATAG
Cbren.WS247	Cbren.WS247_Fam_302_28_1	1	28	0.000000	AGGTGGCTCCACTCCTCACAGGTCCCCA
Cbren.WS247	Cbren.WS247_Fam_303_27_1	1	27	0.000000	ACTGATTTTCGAGAAAATTTCGCAAAA
Cbren.WS247	Cbren.WS247_Fam_304_27_1	1	27	0.000000	TCCTGAACCACCTGGAGTTCCTGGACT
Cbren.WS247	Cbren.WS247_Fam_305_27_1	1	27	0.000000	AAGTCCGATCCGGTCTATTTCGTCCCA
Cbren.WS247	Cbren.WS247_Fam_306_25_1	1	25	0.000000	TTTTCGAAAATTTTTCGGACCAAAT
Cbren.WS247	Cbren.WS247_Fam_307_24_1	1	24	0.000000	CCAGAAAAAAAAGAATTCCGTAAG
Cbren.WS247	Cbren.WS247_Fam_308_24_1	1	24	0.000000	TAGGACTAAAACACCCGGCTGTTT
Cbren.WS247	Cbren.WS247_Fam_309_24_1	1	24	0.000000	ATGGCTCATGGTGGTCAAAGTCTG
Cbren.WS247	Cbren.WS247_Fam_310_24_1	1	24	0.000000	ACATTCGGACAACTGCATATTTGG
Cbren.WS247	Cbren.WS247_Fam_311_24_1	1	24	0.000000	AACCGATGCTCTAAGATCTCCGGC
Cbren.WS247	Cbren.WS247_Fam_312_24_1	1	24	0.000000	TTACGACCGGAATCCCACCGGGAA
Cbren.WS247	Cbren.WS247_Fam_313_24_1	1	24	0.000000	AAATCTGAAAAATCTGGCGTTGCG
Cbren.WS247	Cbren.WS247_Fam_314_24_1	1	24	0.000000	AGTTGGCGGAATTCCTGGTTGTCC
Cbren.WS247	Cbren.WS247_Fam_315_24_1	1	24	0.000000	AGCAATAGCAGTTGATCAGCCTGT
Cbren.WS247	Cbren.WS247_Fam_316_24_1	1	24	0.000000	GATATCTGGACGCTCGAAAATTGA
Cbren.WS247	Cbren.WS247_Fam_317_23_1	1	23	0.000000	GGCAGCTTGCCCACTTTCCTTCC
Cbren.WS247	Cbren.WS247_Fam_318_22_1	1	22	0.000000	TTTTCAGGCTGTTGTCTGCGTT
Cbren.WS247	Cbren.WS247_Fam_319_22_1	1	22	0.000000	ATGGAATTTTTGAACACAAATT
Cbren.WS247	Cbren.WS247_Fam_320_22_1	1	22	0.000000	CCGCCCACTTTTGGGTTTAGCT
Cbren.WS247	Cbren.WS247_Fam_321_22_1	1	22	0.000000	GATTTCGGGACAGTTTCCTGGG
Cbren.WS247	Cbren.WS247_Fam_322_22_1	1	22	0.000000	TTGGAGCTGCCTAGGCTACAAA
Cbren.WS247	Cbren.WS247_Fam_323_22_1	1	22	0.000000	TCCCCCGGCGAATCGAAATCAG
Cbren.WS247	Cbren.WS247_Fam_324_22_1	1	22	0.000000	TTTTCATGGATTTTTTCCGAAC
Cbren.WS247	Cbren.WS247_Fam_325_22_1	1	22	0.000000	GAGCCCAAATTCAAGCTTTTTT
Cbren.WS247	Cbren.WS247_Fam_326_22_1	1	22	0.000000	ACCCGGGTAGGGACGAGTCGAG
Cbren.WS247	Cbren.WS247_Fam_327_21_1	1	21	0.000000	TTCCAGTAGAAGAGGCAGTGC
Cbren.WS247	Cbren.WS247_Fam_328_21_1	1	21	0.000000	TGAGGTTAAGGAGCCTAAGCT
Cbren.WS247	Cbren.WS247_Fam_329_21_1	1	21	0.000000	GCAGGTTGCCGGAAAAGAGGA
Cbren.WS247	Cbren.WS247_Fam_330_21_1	1	21	0.000000	TACAGTAATCCTAGAGGGTCA
Cbren.WS247	Cbren.WS247_Fam_331_21_1	1	21	0.000000	TTAGGCTTAGTAACATAAGAT
Cbren.WS247	Cbren.WS247_Fam_332_21_1	1	21	0.000000	GAGCAACCGGCTCCTGCACCT
Cbren.WS247	Cbren.WS247_Fam_333_21_1	1	21	0.000000	TCTACAGTAATCTCCATTTTT
Cbren.WS247	Cbren.WS247_Fam_334_21_1	1	21	0.000000	CAGGAAGAAGGAACTCAAGAG
Cbren.WS247	Cbren.WS247_Fam_335_21_1	1	21	0.000000	CAAATTTTTTTCGGGATTTTT
Cbren.WS247	Cbren.WS247_Fam_336_21_1	1	21	0.000000	AGACAAACAGACACCTATTAT
Cbren.WS247	Cbren.WS247_Fam_337_20_1	1	20	0.000000	CATTGCACCTCCATTTTGAC
Cbren.WS247	Cbren.WS247_Fam_338_20_1	1	20	0.000000	CAAGAGTTTCTATGAAGATG
Cbren.WS247	Cbren.WS247_Fam_339_20_1	1	20	0.000000	TGGTCTAGAATCCCAAAAAG
Cbren.WS247	Cbren.WS247_Fam_340_20_1	1	20	0.000000	TTCTAGGCGATTCCCTGGAA
Cbren.WS247	Cbren.WS247_Fam_341_20_1	1	20	0.000000	CTGAAAATGTCTTAATTACG
Cbren.WS247	Cbren.WS247_Fam_342_20_1	1	20	0.000000	GCTGCTAGAACCAGTCTGGC
Cbren.WS247	Cbren.WS247_Fam_343_20_1	1	20	0.000000	TGTAGAGGTTAAGGACTGTC
Cbren.WS247	Cbren.WS247_Fam_344_20_1	1	20	0.000000	GCCTTCAAAGTAGTCCATGC
Cbren.WS247	Cbren.WS247_Fam_345_20_1	1	20	0.000000	TCTCAACTGCTTTAGGCCGC
Cbren.WS247	Cbren.WS247_Fam_346_20_1	1	20	0.000000	AAGCCTTAGCCGACTTTCCC
Cbren.WS247	Cbren.WS247_Fam_347_20_1	1	20	0.000000	CTGCTTAGGTAAATAGAGGG
Cbren.WS247	Cbren.WS247_Fam_348_20_1	1	20	0.000000	AGGGGGCCCATCTTAAGAGA
Cbren.WS247	Cbren.WS247_Fam_349_20_1	1	20	0.000000	CATAAAATGCTTTTGCAGGT
Cbren.WS247	Cbren.WS247_Fam_350_20_1	1	20	0.000000	TAAGCAGCTTTGAGGGGTCC
Cbren.WS247	Cbren.WS247_Fam_351_20_1	1	20	0.000000	AATTTGAATTTAGAATGGTA
Cbren.WS247	Cbren.WS247_Fam_352_20_1	1	20	0.000000	TACAGTAAGCTCAAAAAAGC
Cbren.WS247	Cbren.WS247_Fam_353_20_1	1	20	0.000000	AGCGGCTTAGAAAGGTTAGG
Cbren.WS247	Cbren.WS247_Fam_354_20_1	1	20	0.000000	AGACTAGATCAGGCCAGATT
Cbren.WS247	Cbren.WS247_Fam_355_20_1	1	20	0.000000	GCGGGCCTATGCCGAAGCTG
Cbren.WS247	Cbren.WS247_Fam_356_20_1	1	20	0.000000	GGCAAGCTGCTTAACTGTTG

Cbren.WS247	Cbren.WS247_Fam_357_19_1	1	19	0.000000	AATTGAAAATGCTCCAGCA
Cbren.WS247	Cbren.WS247_Fam_358_19_1	1	19	0.000000	GAAATGCGCCAAGACACAA
Cbren.WS247	Cbren.WS247_Fam_359_19_1	1	19	0.000000	TGACGCGTTTTCGGTGGGT
Cbren.WS247	Cbren.WS247_Fam_360_19_1	1	19	0.000000	AGAAGCGTTTTGTGCGTCC
Cbren.WS247	Cbren.WS247_Fam_361_19_1	1	19	0.000000	TCCTTTTTTCCCATCCGTT
Cbren.WS247	Cbren.WS247_Fam_362_19_1	1	19	0.000000	CAAATCTAGGTTCTTGCCG
Cbren.WS247	Cbren.WS247_Fam_363_19_1	1	19	0.000000	AAGGGACCGTTCAAGTTTG
Cbren.WS247	Cbren.WS247_Fam_364_19_1	1	19	0.000000	AGTGGTCCCATGGGAGTTT
Cbren.WS247	Cbren.WS247_Fam_365_19_1	1	19	0.000000	GCCTAAACCCAAGGGTAAG
Cbren.WS247	Cbren.WS247_Fam_366_19_1	1	19	0.000000	TCAAAGGACCCTATAATTG
Cbren.WS247	Cbren.WS247_Fam_367_18_1	1	18	0.000000	AGCTGAGCCAAAGGTAGT
Cbren.WS247	Cbren.WS247_Fam_368_18_1	1	18	0.000000	TGTGACAAAAAAGAGGAG
Cbren.WS247	Cbren.WS247_Fam_369_18_1	1	18	0.000000	GGCACTGGCGTCTTTAGC
Cbren.WS247	Cbren.WS247_Fam_370_18_1	1	18	0.000000	TCTTTTACTTTTACTGAT
Cbren.WS247	Cbren.WS247_Fam_371_18_1	1	18	0.000000	CCGAGGCGACGACTACCA
Cbren.WS247	Cbren.WS247_Fam_372_18_1	1	18	0.000000	GGCAAGTTGCCCAAACGA
Cbren.WS247	Cbren.WS247_Fam_373_18_1	1	18	0.000000	ATTCGTCCTCCTTTTCTC
Cbren.WS247	Cbren.WS247_Fam_374_18_1	1	18	0.000000	TTTTCGAAAATGCCCATT
Cbren.WS247	Cbren.WS247_Fam_375_18_1	1	18	0.000000	CACGAGATGCAGAAGCTC
Cbren.WS247	Cbren.WS247_Fam_376_18_1	1	18	0.000000	CCACGTCCACTGTTTCCA
Cbren.WS247	Cbren.WS247_Fam_377_17_1	1	17	0.000000	AAATTTCAAAACTTTTT
Cbren.WS247	Cbren.WS247_Fam_378_16_1	1	16	0.000000	GACAACCGGATTATCG
Cbren.WS247	Cbren.WS247_Fam_379_16_1	1	16	0.000000	ATCTTTGACTTCCAGT
Cbren.WS247	Cbren.WS247_Fam_380_16_1	1	16	0.000000	ACCCAGGTACTCATAT
Cbren.WS247	Cbren.WS247_Fam_381_16_1	1	16	0.000000	TAGGTTTAGGAGAGTT
Cbren.WS247	Cbren.WS247_Fam_382_16_1	1	16	0.000000	AAACGGAAAAACGGAA
Cbren.WS247	Cbren.WS247_Fam_383_16_1	1	16	0.000000	GAAAGTGAGAGAGAGG
Cbren.WS247	Cbren.WS247_Fam_384_16_1	1	16	0.000000	AGTGCAATCGTGCTCA
Cbren.WS247	Cbren.WS247_Fam_385_16_1	1	16	0.000000	GAAATCTGACGTATAT
Cbren.WS247	Cbren.WS247_Fam_386_15_1	1	15	0.000000	TCAGTTGTCGAGGTT
Cbren.WS247	Cbren.WS247_Fam_387_15_1	1	15	0.000000	TCCGGCGCGTAGGGT
Cbren.WS247	Cbren.WS247_Fam_388_15_1	1	15	0.000000	CGTCAGGCTGAGGTC
Cbren.WS247	Cbren.WS247_Fam_389_15_1	1	15	0.000000	TCAGGCTTAGGCTTT
Cbren.WS247	Cbren.WS247_Fam_390_15_1	1	15	0.000000	GGAGGCGGAGGATGT
Cbren.WS247	Cbren.WS247_Fam_391_15_1	1	15	0.000000	AGCTGCAGCACGAGC
Cbren.WS247	Cbren.WS247_Fam_392_15_1	1	15	0.000000	CTACTACAACTACGC
Cbren.WS247	Cbren.WS247_Fam_393_15_1	1	15	0.000000	CGAAAAATTTTTTTT
Cbren.WS247	Cbren.WS247_Fam_394_14_1	1	14	0.000000	GGAGGTTTTTCCCG
Cbren.WS247	Cbren.WS247_Fam_395_14_1	1	14	0.000000	CTGCCTAGCCCTAA
Cbren.WS247	Cbren.WS247_Fam_396_14_1	1	14	0.000000	ATTCTACATTTATA
Cbren.WS247	Cbren.WS247_Fam_397_12_1	1	12	0.000000	TGACCTTGACCT
Cbren.WS247	Cbren.WS247_Fam_398_12_1	1	12	0.000000	CTGTACCTGGGC
Cbren.WS247	Cbren.WS247_Fam_399_12_1	1	12	0.000000	CCTTGCCGTCTC
Cbren.WS247	Cbren.WS247_Fam_400_12_1	1	12	0.000000	GCTCCGGCTCCG
Cbren.WS247	Cbren.WS247_Fam_401_11_1	1	11	0.000000	CCTCTACTAAC
Cbren.WS247	Cbren.WS247_Fam_402_11_1	1	11	0.000000	AGCCTTCAAAT
Cbren.WS247	Cbren.WS247_Fam_403_10_1	1	10	0.000000	ACAAAACGCA

C. remanei

Genome	Family name	Number of sequences	Alignment length	Score	Consensus without gaps
Crema.WS247	Crema.WS247_Fam_1_14_208	208	14	0.817522	TTCCGacTTCCGaC
Crema.WS247	Crema.WS247_Fam_2_184_98	98	187	0.896850	TTCACAAAAGTCTAtATGAGAAnTTCCAAaACTGTGCTTTcAAcGTGTATTGTCTCTAACTTTTTTTCTGGATGAGATATCTGAAAACAGTAAAgtGcaTTCGAattGGCAaTGGATtgTCTACTaCTGTGCCAAtTTTCAGCCAATAacGTTCAAAcTGAACAAGGCCGCACACGCTTGAAGT
Crema.WS247	Crema.WS247_Fam_3_180_79	79	186	0.866553	TTTTGAGATTTGGTCCAAGTGGaCTTTTCTGCCAAtACATTTTTtATCAGAACTCAGTCAGTTTTGgACGGATCncagAGATtTTTATACCATTCGAAAGCtCTCGTTTTTCTCTAtAAAAATGATcTAATTTaAAAAAATTTTGTTCAAAAATAAAGTTGTAGTGACATTTCCGTCAAT
Crema.WS247	Crema.WS247_Fam_4_14_69	69	14	0.873300	GAAgAAGGAAGAAG
Crema.WS247	Crema.WS247_Fam_5_54_47	47	70	0.607405	aTtATAATTTTaTTGATTTCTAgATttAAAAAAGTCCTGTTTTTAGGAaTTTcA
Crema.WS247	Crema.WS247_Fam_6_46_33	33	46	0.876098	TCAGAAAATGTCCGACAGACATTTTCGAgTACTTCnnAAnATGTAT
Crema.WS247	Crema.WS247_Fam_7_12_32	32	14	0.605415	TTtTCGAaAgAT
Crema.WS247	Crema.WS247_Fam_8_192_31	31	196	0.874109	TTCAGCTTCATTTTTCACCAAATTCAAAATAACTCATTTCATTCAGATccTACtAnAAAAAGaAAACCACCAATCGAAAGAACTCGttGATTTACATCAAGTTCTATGGTTaATCTnTTTTCTCATCTTCTTaTTtCATTTTTATGATCAAAAAACtGTCCGCGAGAAATcaATTTGACATTTTCGCCACTT
Crema.WS247	Crema.WS247_Fam_9_20_29	29	20	0.774713	TTAGACGTCCCaaAAAAcAn
Crema.WS247	Crema.WS247_Fam_10_27_28	28	31	0.609234	gGAAGTCGGAAGTaAAaTTcctaAtCc
Crema.WS247	Crema.WS247_Fam_11_20_27	27	21	0.748971	CAAGTATCCAAAAnTtAnac
Crema.WS247	Crema.WS247_Fam_12_130_26	26	131	0.949485	CAACACTCGAGTTGGGTgCTTGTCAAATAAAGTTaATTTTTAGgATTATATCATTATAAATAGGTAATTATTAACtTTGTTTAgaTGATAGGTAAGCCAACTGGCAGTGTTGGGCTCCTTTTGGAGTTGC
Crema.WS247	Crema.WS247_Fam_13_14_26	26	17	0.680995	TCCGGaTTCCGGAT
Crema.WS247	Crema.WS247_Fam_14_119_19	19	121	0.802394	aTCCACGTCATAAAAAGtTAtGaGCcGTTTTCTAAAAtGTGAcGACGTGAAgGATtTTTTGATATCaTcGGAtTCaGaAAAAaTAGAGGATTCCAAACGTGTnGTCcgTTtCtGGATTG
Crema.WS247	Crema.WS247_Fam_15_21_19	19	21	0.834029	CTGAGGCTAACTGaGatTTTG
Crema.WS247	Crema.WS247_Fam_16_27_17	17	27	0.860203	CGCTAgAATCGnCTGGGGTGCCTGCGG
Crema.WS247	Crema.WS247_Fam_17_64_16	16	64	0.873003	AGATGATCGAGAACATATGTAtTTTCATTCTCTcTcTcTTTCtTTTTTCTCTCATCTCATTccG
Crema.WS247	Crema.WS247_Fam_18_10_16	16	10	0.918889	CTTCTAGAAA
Crema.WS247	Crema.WS247_Fam_19_192_15	15	193	0.902015	GTCCGATGAAAACGAAATGAGTTGCGTGTGATTTACTGAAtTTCAgCATTCAATTTGGGCGAAAatTAGaAAATGAAGTCTtGCACAGCGTTTTTCGATTGTgAAACAAGAAAATAAGAAaAAGTGGAcATTCTATCCATAGAACTAgaACnAACTCAACGAGATCTTTCTAATGGTaTTTTCAGTATTAcT
Crema.WS247	Crema.WS247_Fam_20_70_15	15	70	0.914376	TGAGGTGAGAGAAAGAAAATgATgAgAAaAAGAGAtaAAAATaAAGGAGTTGTTCTCcATCACCTCCTAT
Crema.WS247	Crema.WS247_Fam_21_61_15	15	63	0.822424	CTGACTGCTTCGGCTGgCTGAGAAAAGACTCTaAAACcCCgTCCCatAGAgTACTGTGTAC
Crema.WS247	Crema.WS247_Fam_22_12_15	15	17	0.577591	TaAGCCTAAGcC
Crema.WS247	Crema.WS247_Fam_23_71_14	14	76	0.751350	gGnGCTGAGCntAgGGGCGGgGcgAGAGCAAGCTcCaGGGtGtCCTgTTTtaAgGTAATCACTACTTCcAG
Crema.WS247	Crema.WS247_Fam_24_92_13	13	100	0.767009	agTTCTTATCAAAAAGATAAGaaAATaaAaAcATAGGCCAATTgACACGTCTGTCGTGGAGaaTTtAgTTtCTCCCCTGCAGATATccTTAG
Crema.WS247	Crema.WS247_Fam_25_43_13	13	43	0.875174	CAGaAGAAaTGAAAAACGATCAcTGaTcGTTTTTGAGATTCTg
Crema.WS247	Crema.WS247_Fam_26_14_13	13	14	0.985348	AGGGTCTAGGGTCT
Crema.WS247	Crema.WS247_Fam_27_11_13	13	12	0.799145	TTaCCGGtAAA
Crema.WS247	Crema.WS247_Fam_28_34_11	11	35	0.706493	GAAGTCGGAAGTCGGAAGTaAAaTTTnttAtcCG
Crema.WS247	Crema.WS247_Fam_29_180_10	10	189	0.880854	AGCAGTTTTCaATGGTACGGAAACcCAGgAAAACAGGGCACGAGAGCGTcATCCTTtTGGcccATAATAGTATGAaACATGTCAGTTAAAATAgTcTAAACGGTGCCGCTTtCAGCTcTCTAcAATTGAGGTCATTTGACCTTTCGAATTTCCAGTAAATCTAAGCAATACaGTGAAAAGT
Crema.WS247	Crema.WS247_Fam_30_133_9	9	136	0.726511	TGGTAAATCCCGAAGGAcCCaACCCACCAgcnTCCGCCAAATaACntATCtTgACTgaACGTcCctAAcaAaCTAnnTACTgAgAATcaAtncaaAAAGTGAAcagcctTcGAcAAGcATcCAACtTcAGCTA
Crema.WS247	Crema.WS247_Fam_31_66_9	9	66	0.971942	ATTTTCAGATtTCCTCTTTCTTTCTTTTTTTCTGATCTTCTGATATCCTCGAAAAAGTGCGTCGCAC
Crema.WS247	Crema.WS247_Fam_32_59_9	9	62	0.710872	cTTCtAGAAaCTtCtAGAnAAAcTTTTGAanTTcggCGGGAATTCAAATATTCtaGAAc
Crema.WS247	Crema.WS247_Fam_33_15_9	9	17	0.711329	ATTTCgAAACCGGAC
Crema.WS247	Crema.WS247_Fam_34_16_9	9	16	0.914352	ATCGGGTGCCCAAAAn
Crema.WS247	Crema.WS247_Fam_35_10_9	9	10	0.837037	tTAATTTTtA
Crema.WS247	Crema.WS247_Fam_36_161_8	8	168	0.882370	AGCGTGTTTTGgTTAGTATTTGTGCAAAGTTTCAAGTCATTTGACCCAGGAATCTCTCTGATCGCAGTGATTCAGACTAATTGGTAGAgGCCAAAACTGGGCATTTCCCATAAcTTTTTTTCTAGAAGAGCTATCGACAAACGCTAACTtGCATaAGAACA

Crema.WS247	Crema.WS247_Fam_37_132_8	8	138	0.681073	tagtAGTTGTTgAgTTTGTgTcGgGATCGGtGAATCATcaAGtAGCGTTGGctnngcgttctTcgnancgCGCCAATAcTCAATTTGgTTCaTtCGAAAACGgTACAaTTTAGAGTTGGGTCACTATaAGT
Crema.WS247	Crema.WS247_Fam_38_21_8	8	22	0.658820	TTCAACAAgTTGTGtTTnTnT
Crema.WS247	Crema.WS247_Fam_39_20_8	8	22	0.584957	TTTCTAGGCCatCantGagn
Crema.WS247	Crema.WS247_Fam_40_20_8	8	21	0.740646	TTGGTCTGCTAGCTCtcAnA
Crema.WS247	Crema.WS247_Fam_41_95_7	7	95	0.894403	ATTTCATTTTTcTGATCAAAAAACTGTctGCGAGAAAcGAATTTcACgTTTTCGCCACTTTTgAGCTTCATTTTTgACgAAATTCAAAATAtCTC
Crema.WS247	Crema.WS247_Fam_42_19_7	7	19	0.839599	GGCCTCCGATTtTtttATG
Crema.WS247	Crema.WS247_Fam_43_13_7	7	14	0.853741	AAAAAAAaTTTGA
Crema.WS247	Crema.WS247_Fam_44_183_6	6	189	0.751088	AtAATTTTanTgATTTCTAaccCGGGAGGATTCAAACCCGCGTCTACTGAAGTAaaCcTCAGCCCGGATAGTATAGACTCcttCAcCcTCTGatCGaagtgTGTCTAACCGGtaCCGACACAGATAAGAATGGTGGGGGAAGTTAATTtcAtaaAAAaTAcCCTGTTTtTaGgaATTTTaATt
Crema.WS247	Crema.WS247_Fam_45_181_6	6	181	0.824800	ATTCTTTCTTGTGTCGGATCCTGTAAGACTCGGTCAGTTACGCTGCGAGTAGTCTTACCTAtGGGCTGTGTTTACTCACGGTAGACGCGGGATTGAATCCaCCCGGGaTAGAATATTTTAGAAATTTCATAAAAAGTATCAAAAAACAGtAtaatttcgtttctttatgacttccaacacc
Crema.WS247	Crema.WS247_Fam_46_50_6	6	53	0.787421	TTCATaAAAAATATCCAAAAAcAGGaTAATTTCTTTTCgTATATCAGAAT
Crema.WS247	Crema.WS247_Fam_47_27_6	6	35	0.690159	ACTTTTTAGGCTTTcAGGCCGGCCCGG
Crema.WS247	Crema.WS247_Fam_48_21_6	6	23	0.547343	GATTTACGGaAgcttTGTGTn
Crema.WS247	Crema.WS247_Fam_49_194_5	5	194	0.953265	TCTCTAAATATCTCTGTTCGTATTAcAGATACAGAAAAACGGTTTtCACCATTCGAaCGAGCATAttTTcTTACATCAGATTCTATGGATTTCTAATTTccTcAcCCGTTTAGTTTTTATTtaAAACCAAAAAAGTGCTCATTTCCACGCGACATCaTCATTTCCtAgGTTTTCCATACCTCGAAATATACAAA
Crema.WS247	Crema.WS247_Fam_50_191_5	5	192	0.916667	TTTCTCTATCAATATGACCTAAttTTATAAAATTTCcgTgaAAAATAAAGCCGTAATAAAGGATGGCCGAtGACgTAAAAGTTGGTCCATTTCTTgGGTTTGTCAAACGTTTTTTCAACACATTTtCATTcGTAACTGTGTCAgTTTTGGTcgGAGCGCAAAGATTTTTATAcCaATCGAAAGagctngTt
Crema.WS247	Crema.WS247_Fam_51_136_5	5	136	0.873529	AGTGAATTTAGtCACAGGcACTCaCtGTTAGAGAACAGGATAATTCCGAAGAACcgcCCTGAACATCACATTGGAGaAACCCgTGTTnCaAaATGTAattTatTgactGAcTATCTAaTTaAtAcCGAACTACAca
Crema.WS247	Crema.WS247_Fam_52_103_5	5	106	0.821069	TCGCGGTCtaTCAtCATGCCTTcTCCaTAAAATTATaAAAATTaTAaAaaaTAcaTTAAgTTTATTAGggACAGGAgtTGGAGTacCaTGaGACgGAtTGCAT
Crema.WS247	Crema.WS247_Fam_53_62_5	5	66	0.783838	CCaACCCATCAaGTCTTCgctTtCtCtGGCCAAAAaTACgAGCGAGTTGGGAGTAAATACTC
Crema.WS247	Crema.WS247_Fam_54_45_5	5	45	0.976296	GgCACTCTTTTACAGAGATTTTTGAAAATGGtTTAGAAAATGAGT
Crema.WS247	Crema.WS247_Fam_55_44_5	5	44	0.927273	AAAATGgGTAgTACCCATTTTCnAATCGAAACaAGAAATCTgTG
Crema.WS247	Crema.WS247_Fam_56_29_5	5	35	0.733333	TCCGGATTCCgGAAAATgAAAAttTTcAT
Crema.WS247	Crema.WS247_Fam_57_29_5	5	30	0.724444	tACGAATTTAtAaTcgAAAtTCtcAAAAa
Crema.WS247	Crema.WS247_Fam_58_19_5	5	25	0.572000	TTGGTCTGCTAntGTGaTt
Crema.WS247	Crema.WS247_Fam_59_16_5	5	18	0.683333	tGTCgcAGTTGCGAAn
Crema.WS247	Crema.WS247_Fam_60_14_5	5	14	0.723810	attACAcTGACaaA
Crema.WS247	Crema.WS247_Fam_61_12_5	5	12	0.788889	GctGGaGCTGGa
Crema.WS247	Crema.WS247_Fam_62_222_4	4	223	0.811535	TTCTgAtTAGACaACTACCTGATGCTGTCATCGGtGTCACATGCcGTAAccAACCATTCAgATTTGGGTTttTCgAAAATacAAAcTGCATTgAGAAATTGCgTAtttggctcGTgAGagcacAAACTAcATAATTcTaATTTTAATCCATTTCCTCCagATaAATTACAGCCAGGCCAAAGCAGCAACTCGATAGATTTACTCAttccaatcagattactc
Crema.WS247	Crema.WS247_Fam_63_179_4	4	179	0.939168	CACGTAATAAATAgAATTCGGGTCAGGACGTGTAACGCTTTcTCATGATGATAGGGATgAACAATACGAACAAAATGGTGgTGGCGgTTTTGGGTTATCTATCAcGAACCTCGAGaTCCAGGCGCTCAAAgTTTCCGTCCgTTctCAGATTTCcTTCAaAAAgtGCATTTGCAAACTGA
Crema.WS247	Crema.WS247_Fam_64_136_4	4	137	0.783861	cTCTaagTcTcaGgaAGCagTTcTATTCtCtgtGAGTCTGTCTGTGTGTCtGTCCaTCCGGATGTCCtaTTTCTTtcCGGagACAGTTCAGACGAaAtgaTgAGAAggAGaAATCcgTCcTTTcGTgTGTACCaTc
Crema.WS247	Crema.WS247_Fam_65_73_4	4	74	0.759009	AAAACAAAAAaGtTTTGcCAcgAGTGGGgaTCGAACcCTGGTTGTcAGATTgGtACCAAAAagtCaaaaacaa
Crema.WS247	Crema.WS247_Fam_66_74_4	4	74	0.951952	AAACTGTCTTGATGgGGGCTGAGGTCAGGTGTGCGGGTTcGGGCCTaTaGGAACAGCTTGCATTATGACCcTAG
Crema.WS247	Crema.WS247_Fam_67_65_4	4	65	0.952137	TCATATTTATTCCAtTTTCACTTaTTCATTTTCATTCTAATCTATGAAAAAGAGTaTCCaCTTGT
Crema.WS247	Crema.WS247_Fam_68_41_4	4	42	0.844577	AAAAcGGGATAATTTTGaacAATTTTGAATTAAATCTctcA
Crema.WS247	Crema.WS247_Fam_69_34_4	4	34	0.845588	TAAAAACAGaAcATTTTCTATaAaATTGAgTTCc
Crema.WS247	Crema.WS247_Fam_70_33_4	4	33	0.973064	ATATCACTATTGaTCTTAATTACTCGCTAGAAC
Crema.WS247	Crema.WS247_Fam_71_21_4	4	23	0.638889	CGTAAATCgACattaGAAcnT
Crema.WS247	Crema.WS247_Fam_72_23_4	4	23	0.623188	AAAaTAGtcaAntTTTTGGnTga
Crema.WS247	Crema.WS247_Fam_73_21_4	4	22	0.679293	TTgGgtCaTGACCTAaTTaaT
Crema.WS247	Crema.WS247_Fam_74_20_4	4	21	0.566138	tTTgGTCAgTGTAancATtt
Crema.WS247	Crema.WS247_Fam_75_11_4	4	11	0.681818	TTTCAGcaAnT

Crema.WS247 Crema.WS247_Fam_76_10_4 4 11 0.659091 ATtTCAgcCg
Crema.WS247 Crema.WS247_Fam_77_10_4 4 10 0.666667 AaGAtaCTGa
Crema.WS247 Crema.WS247_Fam_78_174_3 3 174 0.943806
TGgTATAAAAATCTTCaAGATCCGACAAAAACTGAGAAcGTTATAGAAGAAACCGTTTTGGTAGTaAAGTTGGACGATCACTCAAAGTATGGCCATCTCTCATTGGAGCTTCATTTTttATGAAAATTtTTTCAATCCAGGTcAtTTTGATAGAGGAGAA
TaCGGGCTTTCtGA
Crema.WS247 Crema.WS247_Fam_79_171_3 3 173 0.822094
TGTCATTTTCAGgtTTCTTTTTtaCAAaAATATcAATTTTACGTgaCtAGAAgTaAAAAAACATGTgAAaATTACAgATTTATGTCAAAAAGCAATCtAAAACAGtCAaAATCgACcAAATTtcGCnAACGCGcCAaaGGTGCGCCAGtcTcTctGCCTA
CTgCTGAAAAA
Crema.WS247 Crema.WS247_Fam_80_159_3 3 163 0.804363
GCATTCGATCCCCATgTTCCCACcGGTTATAGGGTTCtTCtatGtgAGGAGCAGTAGGCTATCTaAGAATATGCtGCCGGATGGATTCGTTTGCCtATTCatTCAGgTTTCTGaaTGAATCCTtaTAAATtcTccAgtTTtTaaAaGTCCCATTAAcCT
Crema.WS247 Crema.WS247_Fam_81_99_3 3 99 0.955107
CTGAATCAAGCTCCACtGAACCATGTGTCACTGAAACAGAAGAGACTTCTTCAaCTACTTCTTCAGTCACCGAgTCtAgCATTTCTACAGAATCTGTTT
Crema.WS247 Crema.WS247_Fam_82_77_3 3 78 0.762108
GGCTAGCAGGttTagGAtATTCtGanTtTCTGAaACtAGCGAaACCACGTTCGGgAGGTCcgtGACTgTAtGACACA
Crema.WS247 Crema.WS247_Fam_83_60_3 3 60 0.851852 GGTGGATTCTGACCCgGgATTCAgaTATCCGTcACCTGATGTCgcTTGAttCCTTAGAAg
Crema.WS247 Crema.WS247_Fam_84_40_3 3 40 0.933333 AAACTGAGACtCATTGACAGATAACCATATGAAaTGtCAG
Crema.WS247 Crema.WS247_Fam_85_35_3 3 35 0.758730 CATAgACGGTaAGTtTATgaATgAgtAAcAAAACC
Crema.WS247 Crema.WS247_Fam_86_31_3 3 31 0.971326 GTAAGaAAATTAGAAATCCATAGAATCTGAT
Crema.WS247 Crema.WS247_Fam_87_30_3 3 30 0.881481 AGGATCAGTCCAcGAgGaGCCCACaGCCGG
Crema.WS247 Crema.WS247_Fam_88_22_3 3 23 0.782609 tCTACAGTATcCCAgAATGaTA
Crema.WS247 Crema.WS247_Fam_89_22_3 3 22 0.616162 GATTTACngCaTnntcTGTGTC
Crema.WS247 Crema.WS247_Fam_90_21_3 3 22 0.666667 ntGTGGCCtAACTTTTntgAT
Crema.WS247 Crema.WS247_Fam_91_19_3 3 21 0.640212 AAAAATcGatGAAAtTTAn
Crema.WS247 Crema.WS247_Fam_92_20_3 3 21 0.645503 AnTcGAAAATtgAcTTTTTg
Crema.WS247 Crema.WS247_Fam_93_21_3 3 21 0.703704 GCTnAAAATncnCATTTTTGa
Crema.WS247 Crema.WS247_Fam_94_20_3 3 20 1.000000 GCACTTTTCTGTATTCTTGT
Crema.WS247 Crema.WS247_Fam_95_18_3 3 19 0.771930 TCAcTTGCTATATTtGtC
Crema.WS247 Crema.WS247_Fam_96_18_3 3 19 0.818713 cGAAAAAAAATCcCGGAA
Crema.WS247 Crema.WS247_Fam_97_16_3 3 16 0.944444 GGCCTAGAAAaCCGGT
Crema.WS247 Crema.WS247_Fam_98_14_3 3 15 0.651852 ATTnTGAATcCTgA
Crema.WS247 Crema.WS247_Fam_99_15_3 3 15 0.851852 GTAACcTCTGGAGTn
Crema.WS247 Crema.WS247_Fam_100_13_3 3 13 1.000000 ACATTTTCTGAAT
Crema.WS247 Crema.WS247_Fam_101_183_2 2 183 0.948998
AAATCTAATTTTCGGCCGTAAATTGGTGCTTCTGGTCATTTCTTTCGTCTTCTCTAAACAATACAGCTTTGTaGCTATGCGCAAATGAAAGAaCGTGTTcGTTTCcACTAATACAATTacTTATCCTGATTATAACTGTCATTCTaTGACTCAGACAACT
CAATGAGACACAAAAACGGCTGA
Crema.WS247 Crema.WS247_Fam_102_159_2 2 159 0.895178
AAGATGATCAGTTCTTAGTAACGCCGAGGCACGTTCGGAACATCCATACGACCCGAAAAgTGAGTATGAAGaTACAAATAcCTACAGaaAACTAAACGATTTATTTcAAaATGAAAaaGaATCAACAAATgGTTTTTTGTTGAaTGAAAACTCGTTGGG
Crema.WS247 Crema.WS247_Fam_103_153_2 2 153 0.642702
TTTaCccAaTTTcgTTGCaTTTcAGTTGTCGCCGCcGCaAAATATTCAaATTTTcCGTgcAAcaGCaAaTTTCcaATTTTCTGcCaTTTTCGTCaACTTTTCaCAGTaTTTcTTgTTGaaTTTAGaCTaAAaTCCgaaTAgAAAATCaATaAA
Crema.WS247 Crema.WS247_Fam_104_88_2 2 88 0.628788
TcAAAATTCttaTTAACtTaATTAGGgAcAGGAaTTGGAGgaaCATGaGacAgGACTGCACTCGCgGTCCTCCaaaCATGCCtTcTCC
Crema.WS247 Crema.WS247_Fam_105_75_2 2 75 0.928889
GAACTGCTTTGaACACTTGACGAAGTTTCTGTCGTGGAACTTTCGAGAGTTGTaGAaGTcACTGGGGTAGATTCA
Crema.WS247 Crema.WS247_Fam_106_64_2 2 64 0.898438 AAAAATGAAGCTGAAAAGTGGCGAAAATGTCAAaaTGATTTCTCGCGGACaGTTTTTTGATCat
Crema.WS247 Crema.WS247_Fam_107_50_2 2 50 0.840000 CTTCGACGGGaAGTCAACaGAAGATaTGGGTAGAAGTaCCTACCCCAAGa
Crema.WS247 Crema.WS247_Fam_108_49_2 2 49 0.717687 TGGCCTAAaAACaTCAAAATTgGGAcTTCTAaGCCATCAAcacCCaAGg
Crema.WS247 Crema.WS247_Fam_109_44_2 2 44 0.655303 taCAGTAACCCGgATCCcAAaAACccAAAAcTTGGATCaTaGgT
Crema.WS247 Crema.WS247_Fam_110_42_2 2 42 0.603175 CAGCGCTAAAAaccaTGaaGTcGaAcTTTgAACGCTGCTaAa
Crema.WS247 Crema.WS247_Fam_111_42_2 2 42 0.682540 CGCTGCTAAAaAGCGcTAAAATccAAaaTAccAaAAaTTTAG
Crema.WS247 Crema.WS247_Fam_112_41_2 2 41 1.000000 TTTTTTGGGGAAAAATTAGACTTTTTGAGGGAAAAAATGCC
Crema.WS247 Crema.WS247_Fam_113_39_2 2 39 0.850427 TTcCCTTAGTTGGATGATGGaCTCACCCaCGAGACCTGt
Crema.WS247 Crema.WS247_Fam_114_39_2 2 39 0.863248 GCTTCAGTGACGGCaaCAGTTGTGGTCTCCTCgCCGGAa
Crema.WS247 Crema.WS247_Fam_115_37_2 2 37 0.711712 ATcCTGGATCCAGtTACAGTaACCCcCaAgATCTaAG
Crema.WS247 Crema.WS247_Fam_116_35_2 2 35 0.619048 ACGCTGCGAGCTCACTAgCaacCTGcTaTcTTTaG
Crema.WS247 Crema.WS247_Fam_117_33_2 2 33 0.883838 AGGTAGTCTaCTATTTTCGGTTTTTcTCATTTt
Crema.WS247 Crema.WS247_Fam_118_32_2 2 32 0.750000 CAGCGCTAAAacCGgaaTTTAGCGCTGGcTAG
Crema.WS247 Crema.WS247_Fam_119_31_2 2 31 0.806452 AAGAAAGAATGGTgGaGaAAGTCATaAAGAA

Crema.WS247	Crema.WS247_Fam_120_31_2	2	31	0.741935	TGaCGCGTTTTTCAACTccCTGGGaTaCCgT
Crema.WS247	Crema.WS247_Fam_121_31_2	2	31	0.827957	AAAAcGGGATAATTTTGAAcTAAATcTTAcA
Crema.WS247	Crema.WS247_Fam_122_30_2	2	30	0.666667	AAaAAGGATCAaAAGAAACCcGccgaAGAG
Crema.WS247	Crema.WS247_Fam_123_26_2	2	26	0.769231	GGAAaAtTTTGAaTTCCCGCCAAAAc
Crema.WS247	Crema.WS247_Fam_124_24_2	2	24	0.618056	gTCgGAAAttTTcCGAATcTTTTT
Crema.WS247	Crema.WS247_Fam_125_22_2	2	22	0.621212	CTTAGGCTTAgcAaATCTTagg
Crema.WS247	Crema.WS247_Fam_126_22_2	2	22	0.704545	CcAGaTTTAcAGCCTAAAATTa
Crema.WS247	Crema.WS247_Fam_127_22_2	2	22	0.643939	aACTCGCAGCGTcAAaTaaTAa
Crema.WS247	Crema.WS247_Fam_128_22_2	2	22	0.878788	GCACTAGTCGTGTGCATTaTcA
Crema.WS247	Crema.WS247_Fam_129_22_2	2	22	0.818182	aAAGCCTaAAAaGTCCGGCCTA
Crema.WS247	Crema.WS247_Fam_130_21_2	2	21	0.603175	AAaAACaACACcAaTTCCAac
Crema.WS247	Crema.WS247_Fam_131_21_2	2	21	0.714286	AAAaTaGGCTgAAAATTGcTC
Crema.WS247	Crema.WS247_Fam_132_21_2	2	21	0.650794	GAAAAtATCAAaaTTTaGAcT
Crema.WS247	Crema.WS247_Fam_133_21_2	2	21	0.777778	TAAATCTACATcTCAgCcCCG
Crema.WS247	Crema.WS247_Fam_134_21_2	2	21	0.619048	TcaTTTTagGCCaAATTTaAG
Crema.WS247	Crema.WS247_Fam_135_20_2	2	20	0.666667	GaTCaAAAAaCcTCGTTTTg
Crema.WS247	Crema.WS247_Fam_136_19_2	2	19	1.000000	TTCAAAGGACCTCGATATT
Crema.WS247	Crema.WS247_Fam_137_19_2	2	19	0.789474	caATGCACCATATTTGaGC
Crema.WS247	Crema.WS247_Fam_138_18_2	2	18	0.925926	AAAGATTCAAAaTTTTTC
Crema.WS247	Crema.WS247_Fam_139_18_2	2	18	0.629630	AAAccCaTAAAAacCTTA
Crema.WS247	Crema.WS247_Fam_140_18_2	2	18	0.851852	CTCATTTACTACCGaTTc
Crema.WS247	Crema.WS247_Fam_141_17_2	2	17	0.607843	AATTcCtaGAcTTTCGG
Crema.WS247	Crema.WS247_Fam_142_17_2	2	17	1.000000	TCAACTACAAAAATGTC
Crema.WS247	Crema.WS247_Fam_143_16_2	2	16	0.625000	tGACAGACAcACAgac
Crema.WS247	Crema.WS247_Fam_144_16_2	2	16	0.666667	AAtCTGACtAATATAG
Crema.WS247	Crema.WS247_Fam_145_16_2	2	16	0.666667	AaAGCCTaAaAGCCTa
Crema.WS247	Crema.WS247_Fam_146_14_2	2	14	0.714286	TcTGTAaTcTGTAG
Crema.WS247	Crema.WS247_Fam_147_14_2	2	14	0.904762	CAGGCGCGGAGaTC
Crema.WS247	Crema.WS247_Fam_148_12_2	2	12	0.694444	cCGGGCCTgGCt
Crema.WS247	Crema.WS247_Fam_149_12_2	2	12	0.611111	TaAcAATAAaAA
Crema.WS247	Crema.WS247_Fam_150_12_2	2	12	1.000000	TCCCGAGCATAA
Crema.WS247	Crema.WS247_Fam_151_11_2	2	11	0.893939	CTATTTTAGGg
Crema.WS247	Crema.WS247_Fam_152_231_1	1	231	0.000000	GATAGTCGAGATGAGATGGTGACTTCTTGAACAATCCAGTAATCTTCGATTTCAACGATGGCTCGTCCTTCTTCTCAGTTTCTTCCACCACAACTGGAACAACATGTTCTGGTAGCTTCTCATAGCTGAACTCGGATGGATAAGCGTTGACTTCGGTTGTGAGAGGAATGTCGTGAATGTTGGAAGCACGAGATGTCGAATCGAGTGGTCCGTCGTATTGTTCGGAGATTG
Crema.WS247	Crema.WS247_Fam_153_171_1	1	171	0.000000	GTCGTAACCGCATAGGTAACCCAGGAAACCTGGAGAAATGCTATAAAACGCGGGCTGAGGTTTACTTCAGTAGAAATGAGTTTGAATCTTCTCGGTCTAGAAAACATAAGAAATAAAAGGAATTGGGATACTTTCTATTCATCTGACTTCTTCCCACCATTCTTACTCAGT
Crema.WS247	Crema.WS247_Fam_154_170_1	1	170	0.000000	AATCATCTCGACTAGATCAACGGAATCGAGGTACGGTGTCAAAACCTCACAATCACCTGAGATTCAGATAAATGCGGCAAAGTCCTGCACGTCGATTCAACCAATCAATTCGATGTTTCTATCGTATAACAATGGGAAAGAGTCCAACTGATTAGAGAGGAGATTGGGAC
Crema.WS247	Crema.WS247_Fam_155_160_1	1	160	0.000000	ACGACAAAATGCACAGTAACTCCAGGGAAACGTTTCTAGTGTCAAAAACCACTCATAAATCCAAATTTCCTGTTAAAATTTACCTCAGGCTTCGTTTTTTGCCAGATAACTAGTCATTTTGGGTTTCGGTAGCTCCAATACTGTGCATACACCATATCTT
Crema.WS247	Crema.WS247_Fam_156_157_1	1	157	0.000000	TGGCCTAGAAAACCAAATTTAAGAAAACTAGTCCCCGGGTCATTTTTGACAGTTTTAGCTAGTTTTTTTGTGGTTTTTCTTGTTGAAAATTGCGTTTTTCATGATTTTTAGCCAATCAAACAAGAAAAAACAGCCGGGGGACTAGTTTTTCGACACG
Crema.WS247	Crema.WS247_Fam_157_153_1	1	153	0.000000	GAATACGATCTTAGAGCTTATTAGTTCTTATGTTTTGAATTGGAAGAATGGAAATTGAGATTGAAGTACTAGACTTACTGTTGAGATTGAACACAAAATTGGATTTGAGATTTTGAGTTTGGTAACGAGGTTTGAGAGTTGATTTTGATATTT
Crema.WS247	Crema.WS247_Fam_158_146_1	1	146	0.000000	GTGGCCGAGTTTTTCACTTGTTGGCCTAGGAAACCAAATTTTGGGAAAACTAGTCCCCGAGCTCCGATTTTAATGAAAATAACGAATTTGAGCTGAAAAGTAATGGGAAATTCGAAAAAATCGCGGAAATCCGATTAGAAAAGGTG
Crema.WS247	Crema.WS247_Fam_159_141_1	1	141	0.000000	CTCCTAAACGCATGAAACAGTAGACTGCAAGGAGCCTCTGATCCGGAGCAGTTCTAGTCACCACGCGTGCGAAGACCTCTCGTAAGAGGTTCTCCGACTTCGCGAACATGGTTCGCATTCCTTCAGTGCGGGCAGAACCAT
Crema.WS247	Crema.WS247_Fam_160_134_1	1	134	0.000000	ATTCACAAATTTGAATAAATTCTAAGAAACACCGTTTCTCATAAACTTTTGAAATGATAGTTAATTGACTGAAGGCGGTGGGTCGCCGCTTCGGCTGGTACCGTAACCTGATGTTGATTGGTTATCATTAGTGA
Crema.WS247	Crema.WS247_Fam_161_134_1	1	134	0.000000	GACTAGTTTTTTTCATAATTGGAATTTCTAGGCCACGTGTCGGAAAACTCGGCCACGTACCACATTTGGTGGTTTCCTTGCTTTTTGGCTCTAGAAACGATGAAAATGTCAAAAACTGACTTTCTGAGATTGGG
Crema.WS247	Crema.WS247_Fam_162_130_1	1	130	0.000000	CGTGTACTCCTCGTGGAAAAGTCGAAAATTGGAATTTGAAATTTTGAGTCGATTTTTTTCGGTTCGACGCTAAAAACTAGTAGTAACCAGTCCGAATTCCATGAAAATAATCCATTTTGAAAATTCATGC

Crema.WS247 Crema.WS247_Fam_163_128_1 1 128 0.000000
ATCAAGCAATTCAAATTTCAGCAAGAATCGGAATAAGATAATTCAAATCGAAAGACAATCAGAAATACAATAAGTGTTCCTTCTGGCTAGCATGCTAGCACCCACAACACTATACTGCACCTCATCTC
Crema.WS247 Crema.WS247_Fam_164_127_1 1 127 0.000000
GATCCTCTTACGAGAGGTCTACGCACGCGTGTTAATCAGACAACTCCGGATCAGAGGCTTCGGCCGTTGTTCCATGCGTATAGGAGTCAGTTCTGCACGCACAGAAGATATACAAAGAACAGACGGA
Crema.WS247 Crema.WS247_Fam_165_121_1 1 121 0.000000
CTTCTGACACCCGAATCGTTCACATTTTTCCGACTTTTACGAAGTTTTCTTTCCAAAAGCTACTCAATCCCCGAAAAATCGATCTGGAAGGAGGATCATGGGCGAATTTTCGGTTTCCCAG
Crema.WS247 Crema.WS247_Fam_166_116_1 1 116 0.000000
TAGAGACAATACACCTTGAAAGCACAGTCTTGGAATTTCTCATTTAGACTTTTGTGAAACATCAAGCGTGTGCGGCCTTGTTTAGTTTGAACGTTATTGGCTGAAAATTGGCACAG
Crema.WS247 Crema.WS247_Fam_167_114_1 1 114 0.000000
ATCATTTTTGTAGTTGACATCTTTATTTAGTTCTAGAGGGAATAAGATAGGGACGCTTAACTGCCAATGACTAGAAGATAAAAATGATTCTGAAACATTGGACATGTCTCCTTC
Crema.WS247 Crema.WS247_Fam_168_107_1 1 107 0.000000
TTATAGAAGAAACCGTTTTGGTAGTAAAGTTGGACGATCACTGAAAGTGTGGCCATCTCTCATTGGAGCTTCATTTTCAATGAAAATTTTTTTAATCTAGGTCATTT
Crema.WS247 Crema.WS247_Fam_169_103_1 1 103 0.000000
CTCAAAACTTCCAGATTTCTACACTTTTTCATTCAAAAAACCCGTTTTTTTATTCCAATTTCGGCGAAAATCGCTAAATATTCATCCAAAATGGCCATTTTTC
Crema.WS247 Crema.WS247_Fam_170_102_1 1 102 0.000000
AAAAATCACCTTTTTTGAATCAAAAATGGTCTAAAATCGAGGAGAATCGATTGAAAACGACTTGAAATCGCTTAAGGAAGAATTTTTGACTGGAAAAAAGCG
Crema.WS247 Crema.WS247_Fam_171_102_1 1 102 0.000000
CGTATCAAGGAGGTACTGGAGAGAGATATGCCGCTACGAGACCGAAGGATACGATTGATCGTCCAACTGATTCCCTTGACTCCACCACAATCAATCGGTCAA
Crema.WS247 Crema.WS247_Fam_172_99_1 1 99 0.000000
CCAATCCCGAACCATCCGATTATCGATCGTCGCCTTTTCCTTCCCATCCCAAACCATCCGAATTTCAATCGTCGTTGTCGTCGTTCTTCTTATTCTCCA
Crema.WS247 Crema.WS247_Fam_173_96_1 1 96 0.000000
ACTCCAAAAGTAGCTCCTAAGAAGAAGGAATCATCGTCCGACGACTCCTCGGACGATGAGAAAAAAACGGTTTCGAAGCCAACACCAGTCAAGACC
Crema.WS247 Crema.WS247_Fam_174_81_1 1 81 0.000000
GTCCATCTGTGAGTCTGAGTGTCCAGATGTCCAACAAATGCGGATTTTCGGTCAAAAACAGCCAAATTGAACAATTTTCAA
Crema.WS247 Crema.WS247_Fam_175_81_1 1 81 0.000000
GTTCAGGAGTCGTTTCACAGGACTCCACAGTGGTCTCACATGGTTCCGTTGGAGTAACTGGTGACGTAGAATTCTCGCATT
Crema.WS247 Crema.WS247_Fam_176_73_1 1 73 0.000000 AAGATATATCCCGTTCCCAGCTATCCTCCGTTTGCTGACTAAAATGTCTGCTGGCTTCGGTTTGCTAGTATAC
Crema.WS247 Crema.WS247_Fam_177_71_1 1 71 0.000000 TTTCTAGGCCACCAAAACTGTTATTTTCAGCCAAAAATAACCTGATTTTCGGCTAGTGGCCTAACTTTGGG
Crema.WS247 Crema.WS247_Fam_178_70_1 1 70 0.000000 TGAATGAGTAATAGAACCCATAGACGGTAAGCACATGACTGTCTAACAAAACCCATATACGGTAAATAAA
Crema.WS247 Crema.WS247_Fam_179_66_1 1 66 0.000000 GAGTACTGTAGGTGGAGTCGGAGACAGAAGACACTGAGGATCCTGATGTTGTTGAATCTGCTGATA
Crema.WS247 Crema.WS247_Fam_180_63_1 1 63 0.000000 TCGGATGGGTTTGGTCTAGATAAAAGTGTCACCCGATTCTTATTCTTACCTCAGGATTCATCT
Crema.WS247 Crema.WS247_Fam_181_63_1 1 63 0.000000 TTTTTAGGTGAAAAAAATGCGATTTTCAGCCATTTTCGAAAGAAAAATCTCACAAAACGTCAA
Crema.WS247 Crema.WS247_Fam_182_63_1 1 63 0.000000 TTTTTGTTTTGTTTTCTATGGTTCCGAGGGTTGTGGATTCGATCCCCACCCACACCAAAACTT
Crema.WS247 Crema.WS247_Fam_183_63_1 1 63 0.000000 AATCTGGCGTGCTTTTAACGCGGTTTCAGCTATGAAAAGCCCGAAATTTCGTGTTTTTCTTCA
Crema.WS247 Crema.WS247_Fam_184_62_1 1 62 0.000000 TGGCGTGTCTTTGGCGCGTTTTTAAAGTGGAAAATGGTCGTATTTAGCAAAATTTCAAAATT
Crema.WS247 Crema.WS247_Fam_185_60_1 1 60 0.000000 AGCTTAGAAAAGTTTTTTCGTTGACAGACTTAACCGGTGCATGTTCACTGCCGAGTAGGC
Crema.WS247 Crema.WS247_Fam_186_60_1 1 60 0.000000 TTTCTAAGCTGCCTATATGGCAGTGAACAAAATTGATGATCAATAAGCAACTGGCAATTA
Crema.WS247 Crema.WS247_Fam_187_59_1 1 59 0.000000 CGTAAATCTACACAAGGATCGGGATACTGTAGCGGTGAATTGGCTTGTCGATTTACGCT
Crema.WS247 Crema.WS247_Fam_188_58_1 1 58 0.000000 AGGCGCGCCAGCTATCTAGCAATCAATTTTTCAGAAAAATTCTTTAGAAACGCGTGAA
Crema.WS247 Crema.WS247_Fam_189_57_1 1 57 0.000000 TGACCTAGAAACTTCAACTTGGAAAAGTTAGGCCACTGTCAATTCTCTTGGGATTGG
Crema.WS247 Crema.WS247_Fam_190_54_1 1 54 0.000000 GGATCCCGAGGAGCTCAGAACTTGAGGAGGAGTCCTAAAGAAGCCCAGAGGAGC
Crema.WS247 Crema.WS247_Fam_191_54_1 1 54 0.000000 ACTACAGAAAATGAACATGTTCAAATTCTGTAGTTTAAGGAGAAAGCCACGTGG
Crema.WS247 Crema.WS247_Fam_192_51_1 1 51 0.000000 AGGAGCCCCACTTGCAGCCGAGGAAGGTGGTGAAGGAGGCAACGAAGGAGC
Crema.WS247 Crema.WS247_Fam_193_50_1 1 50 0.000000 ACGCGTTTTTCTCGTAAAAATCACCAAAAATTGTGTCTAGGGCGCCTCCG
Crema.WS247 Crema.WS247_Fam_194_50_1 1 50 0.000000 GACGCGCTTTTTATCAGATAAAACTGATTTTTTGAATCTGGCGTACCTTC
Crema.WS247 Crema.WS247_Fam_195_48_1 1 48 0.000000 TGCTTGCTTCGGCCTTTCGGCCTAGGCTTGCTGCCAATTACATCGACC
Crema.WS247 Crema.WS247_Fam_196_48_1 1 48 0.000000 GGTGGCCTAGAAATCCAGAAATGCGGAAAACTAGGCCACGGTCTATTT
Crema.WS247 Crema.WS247_Fam_197_48_1 1 48 0.000000 GAGGACAAGGACCAGACTTCGGACCGTCCGGTGATTTACCAGACCGTA
Crema.WS247 Crema.WS247_Fam_198_48_1 1 48 0.000000 TCAGCTTCCTTCTTGGTCTTGGCTTCAAGATCCTTCTTAGCCTTGTCA
Crema.WS247 Crema.WS247_Fam_199_47_1 1 47 0.000000 ACGCGACCGCAACGCGTGCGAGTTATGGCGCCAAGTTAGCAGCCGCG
Crema.WS247 Crema.WS247_Fam_200_47_1 1 47 0.000000 TGGTGCGCCTTAGGCGCTCTAGTGGGAGAGGAGGGGAGGCACAGGGT
Crema.WS247 Crema.WS247_Fam_201_46_1 1 46 0.000000 TTTAGGCCAATTTTGACCGGGGGACTAGTTTTCCTGAAATTTGGGT
Crema.WS247 Crema.WS247_Fam_202_46_1 1 46 0.000000 TCGAAAATGGTTGATAACAATTCGTTTCATTATTTTCACTTACATC
Crema.WS247 Crema.WS247_Fam_203_43_1 1 43 0.000000 GGTTACTGTACTCAGGATCCGAAATTTTGGATTTTCCAAACAA
Crema.WS247 Crema.WS247_Fam_204_42_1 1 42 0.000000 ACTGGCTCCAGTCGCGGCTCTTCCTCCTTGCGAAGTCGAAGC
Crema.WS247 Crema.WS247_Fam_205_42_1 1 42 0.000000 GACAAGCTGGAATTCCAATTAACCTCATCCCACCAGTTCCAA
Crema.WS247 Crema.WS247_Fam_206_42_1 1 42 0.000000 CCACGGAATCTACCAGTACATCGACCGAGTCTACCAGTACTT

Crema.WS247	Crema.WS247_Fam_207_42_1	1	42	0.000000	CGGAGAGCGGGATGGGGCCGGATTCTGACGATCCTGAGGTTG
Crema.WS247	Crema.WS247_Fam_208_42_1	1	42	0.000000	GACGCGTTTTACATAGAAATAGTGGTTATCTGGGGCACCTTC
Crema.WS247	Crema.WS247_Fam_209_42_1	1	42	0.000000	AAAAATGTCTGAGATCCGTCCAAAACTGACTGAGTTCTGATG
Crema.WS247	Crema.WS247_Fam_210_42_1	1	42	0.000000	CGCGTTTTGATGACTTTTGAGAATGATCTGGCGTGCTGTTGG
Crema.WS247	Crema.WS247_Fam_211_42_1	1	42	0.000000	GGAATAAAATCGCGAGATCGGAACCCCAGAGTACTGAAAAAG
Crema.WS247	Crema.WS247_Fam_212_41_1	1	41	0.000000	TCGGAGAATCGGATATCCGACTTTTTCTCCGAAAAGAAAAG
Crema.WS247	Crema.WS247_Fam_213_41_1	1	41	0.000000	AAAAACGCGTCACAGGTGCCCCCGATTCACCTTTTTTGTCA
Crema.WS247	Crema.WS247_Fam_214_40_1	1	40	0.000000	TCCGGATGTCCACGAGTTTGTCTGTGTGTCTGTCTGTTTG
Crema.WS247	Crema.WS247_Fam_215_40_1	1	40	0.000000	CCCCCCCGAAAAAAAAATTTTCGGGAACAAAGCGGAGACA
Crema.WS247	Crema.WS247_Fam_216_40_1	1	40	0.000000	TTTAGCGCTGACTAGCAGCGCTAAAAACGAAAATTGGACT
Crema.WS247	Crema.WS247_Fam_217_40_1	1	40	0.000000	ACACGCCAGATTTCGGGTTTTCAGAAAAAACGCGCCAAAA
Crema.WS247	Crema.WS247_Fam_218_40_1	1	40	0.000000	TTTGATATTTTGAGTTTGGTAACGAGGTTTGAGAGTTGAT
Crema.WS247	Crema.WS247_Fam_219_40_1	1	40	0.000000	ATTTTTGGCTCAAAATCAGAACTTCTAGCTCACAAACAAG
Crema.WS247	Crema.WS247_Fam_220_40_1	1	40	0.000000	ATTTTGGTCTGAAAATTGGATTTTTGACCTAAAAATCACC
Crema.WS247	Crema.WS247_Fam_221_39_1	1	39	0.000000	ACATTACTTCAATCATCATTTGATCCTTTGCCCTTAGTT
Crema.WS247	Crema.WS247_Fam_222_39_1	1	39	0.000000	AGACTACTGTAGGTTACGGCAATTGCTACAGTAACCCGG
Crema.WS247	Crema.WS247_Fam_223_38_1	1	38	0.000000	AAACCCTGGAGCTTGCTTTAGCCCCGCCCATAGGCTTC
Crema.WS247	Crema.WS247_Fam_224_38_1	1	38	0.000000	ACCTTTGACGCGCTTTCTACTTTTTGACGATCTGGCGC
Crema.WS247	Crema.WS247_Fam_225_37_1	1	37	0.000000	TTAGGCTCCGCCTTCTTGAAAAAGAGGGTGGGGCTAA
Crema.WS247	Crema.WS247_Fam_226_36_1	1	36	0.000000	GGGTACTGTAGCCTCAGGATCCTAATTTTTGGATTG
Crema.WS247	Crema.WS247_Fam_227_36_1	1	36	0.000000	ACCAAGGAGGCCGAGACTCCAAAAGTGAAGCGCGAC
Crema.WS247	Crema.WS247_Fam_228_36_1	1	36	0.000000	GCTACAGTACCACATATCCTATGTATTTCGGCTCCT
Crema.WS247	Crema.WS247_Fam_229_36_1	1	36	0.000000	TTTCCGTCAATTTTTGAGATTTGGTCCAAGTGGTCT
Crema.WS247	Crema.WS247_Fam_230_35_1	1	35	0.000000	TCTGATTTGACGTTACGATATTGAGAAGAGAAATT
Crema.WS247	Crema.WS247_Fam_231_34_1	1	34	0.000000	GAGATGAAAATGAAAATGGTTGTCAATCATTTTC
Crema.WS247	Crema.WS247_Fam_232_34_1	1	34	0.000000	GAAATTTTTGTAGCCGGGTAATCATGCTGAGTGA
Crema.WS247	Crema.WS247_Fam_233_33_1	1	33	0.000000	GGGGCGGAGCTTCAGTACTACTATAAGCCTAAG
Crema.WS247	Crema.WS247_Fam_234_33_1	1	33	0.000000	CAGTAGAGCGCAATTGCACAATTCGAAAATTAG
Crema.WS247	Crema.WS247_Fam_235_33_1	1	33	0.000000	CAAGGAGGATCCGGAGGATACGCTGGAGCTGGA
Crema.WS247	Crema.WS247_Fam_236_33_1	1	33	0.000000	GAGTTCCCTCAGTTTGAGACAATGTTGCTCCAG
Crema.WS247	Crema.WS247_Fam_237_33_1	1	33	0.000000	AGCAGCTTGTTCGGCTGGAATCTCTTGAACTGG
Crema.WS247	Crema.WS247_Fam_238_32_1	1	32	0.000000	TCTGTCTGTCTGTGTGTCTGTCATTCCCGATG
Crema.WS247	Crema.WS247_Fam_239_31_1	1	31	0.000000	GGACCCAACTCGAGTGTTGGCAACTCCAAAA
Crema.WS247	Crema.WS247_Fam_240_31_1	1	31	0.000000	GGGCGGAGTTTGTGCCAAAATACGGAAAAAT
Crema.WS247	Crema.WS247_Fam_241_31_1	1	31	0.000000	TGTCATTTCTGACTGAAATTAGCTCAGAATT
Crema.WS247	Crema.WS247_Fam_242_31_1	1	31	0.000000	TACGCTGCGAGTTTGCAAGCATTTTGAAAAA
Crema.WS247	Crema.WS247_Fam_243_31_1	1	31	0.000000	TTTTCATCGATTTTTGATGGGAAAATCGGGG
Crema.WS247	Crema.WS247_Fam_244_31_1	1	31	0.000000	TTTCAGCTTTTTTGACTCAAACTTTCTCGGT
Crema.WS247	Crema.WS247_Fam_245_31_1	1	31	0.000000	AAACTTGAAAAAATACCATTTTTGAGCCCAA
Crema.WS247	Crema.WS247_Fam_246_30_1	1	30	0.000000	ATGAAATTATCATGTTTTTGGATATTTTTA
Crema.WS247	Crema.WS247_Fam_247_30_1	1	30	0.000000	CGAGGAGCTCAGAATCTGAGGAGGAGAGCA
Crema.WS247	Crema.WS247_Fam_248_30_1	1	30	0.000000	TATTGGTTAAAATAGGTTAATAAACAGTAA
Crema.WS247	Crema.WS247_Fam_249_30_1	1	30	0.000000	AGTCCTACAGTAACCCAGAACTTGAGGAGG
Crema.WS247	Crema.WS247_Fam_250_30_1	1	30	0.000000	CTCCAGAATCCTCCAGAAGACGTCACGACA
Crema.WS247	Crema.WS247_Fam_251_30_1	1	30	0.000000	CTGGAGCTGGTGCTGGAGTCTCTTCGGCTA
Crema.WS247	Crema.WS247_Fam_252_29_1	1	29	0.000000	TCCGCCCACTTTTAAGGTTCTTTTTATGA
Crema.WS247	Crema.WS247_Fam_253_29_1	1	29	0.000000	AGCTGCTTAATTACTATCCAATCGTATGG
Crema.WS247	Crema.WS247_Fam_254_29_1	1	29	0.000000	TCCGGTTCCGGATTCCGGATGAATTTTTT
Crema.WS247	Crema.WS247_Fam_255_29_1	1	29	0.000000	TTAAGCAGCTGCACATGGATGAATGCTAA
Crema.WS247	Crema.WS247_Fam_256_29_1	1	29	0.000000	GTTTCAAAACGTCCTTTTCTCAAAAAACG
Crema.WS247	Crema.WS247_Fam_257_29_1	1	29	0.000000	GTGGATTTACGGAGCTAAATGTCTTATAT
Crema.WS247	Crema.WS247_Fam_258_27_1	1	27	0.000000	GGAGCCCCAGGACCAGCTGGACCAAAG
Crema.WS247	Crema.WS247_Fam_259_27_1	1	27	0.000000	GTACCAGGAGTACCACCTCCTCCAGTA
Crema.WS247	Crema.WS247_Fam_260_27_1	1	27	0.000000	TGATAGCGATGATAGCGATGATAGCGA
Crema.WS247	Crema.WS247_Fam_261_27_1	1	27	0.000000	TTCTCATTAGAGCGCACGTGCCATCGG
Crema.WS247	Crema.WS247_Fam_262_27_1	1	27	0.000000	TACAGTAACCCAGTCAAGATTTAGAAC
Crema.WS247	Crema.WS247_Fam_263_27_1	1	27	0.000000	TGTATGGTCCTGTGGTTCCACCAGTGG

Crema.WS247	Crema.WS247_Fam_264_27_1	1	27	0.000000	GAAATCCGGCGCCCCGGGGCCATCCTC
Crema.WS247	Crema.WS247_Fam_265_26_1	1	26	0.000000	AACTTTTTCGAAAAGTTTAGACCGAA
Crema.WS247	Crema.WS247_Fam_266_25_1	1	25	0.000000	TCTTTCTCTCTTTTTTTCATTATTT
Crema.WS247	Crema.WS247_Fam_267_25_1	1	25	0.000000	TTCCGACTTCCGGGCGTTTTTTCAC
Crema.WS247	Crema.WS247_Fam_268_25_1	1	25	0.000000	ATCTAGGCTCCGCCCACTTAGAGAC
Crema.WS247	Crema.WS247_Fam_269_25_1	1	25	0.000000	TTTCTCTTCCACGGCCACCACGGGA
Crema.WS247	Crema.WS247_Fam_270_24_1	1	24	0.000000	AGTCTCGAAGACCAAGTCTCGCCA
Crema.WS247	Crema.WS247_Fam_271_24_1	1	24	0.000000	GCCTACAACCGTTACAACTACTGA
Crema.WS247	Crema.WS247_Fam_272_24_1	1	24	0.000000	ACGGAAGCTCCGACGACTTCAACT
Crema.WS247	Crema.WS247_Fam_273_24_1	1	24	0.000000	AAGAAGAGGAGGGAGAGCAGAAGA
Crema.WS247	Crema.WS247_Fam_274_24_1	1	24	0.000000	GATCCTAGAGACTACTGTAACTCC
Crema.WS247	Crema.WS247_Fam_275_24_1	1	24	0.000000	ATGTACCCTTTGATCATCTCACAG
Crema.WS247	Crema.WS247_Fam_276_24_1	1	24	0.000000	TCCTTCTTGTTCTTGGCTGCGAGA
Crema.WS247	Crema.WS247_Fam_277_24_1	1	24	0.000000	TCCTTCTTAGCCTTGTCATCAGCC
Crema.WS247	Crema.WS247_Fam_278_23_1	1	23	0.000000	CACAAGTTGTGTTTTTGACTATC
Crema.WS247	Crema.WS247_Fam_279_23_1	1	23	0.000000	AGCTCCGCCCACTATTGACCCTT
Crema.WS247	Crema.WS247_Fam_280_23_1	1	23	0.000000	TTTAGGCTTAGAAGCTCCGCCTC
Crema.WS247	Crema.WS247_Fam_281_23_1	1	23	0.000000	TCTAAAAATTTACAGAAAATTCA
Crema.WS247	Crema.WS247_Fam_282_23_1	1	23	0.000000	TTTTGAGTTGATTTTAATCATGA
Crema.WS247	Crema.WS247_Fam_283_22_1	1	22	0.000000	TACTGTAGTGAATCCCAAGGAA
Crema.WS247	Crema.WS247_Fam_284_22_1	1	22	0.000000	TTACACTGACAATCACCGAAGC
Crema.WS247	Crema.WS247_Fam_285_22_1	1	22	0.000000	AAACTGTTGTCCCCTTGAGTAT
Crema.WS247	Crema.WS247_Fam_286_22_1	1	22	0.000000	TCAATGCACCATATAGTAATGG
Crema.WS247	Crema.WS247_Fam_287_21_1	1	21	0.000000	CGTTTTCAGCTCAAATTTTCT
Crema.WS247	Crema.WS247_Fam_288_21_1	1	21	0.000000	CATGAATTTTTAGTCGAAAAA
Crema.WS247	Crema.WS247_Fam_289_21_1	1	21	0.000000	AGTTCGGCCACCAGGAGAGAA
Crema.WS247	Crema.WS247_Fam_290_21_1	1	21	0.000000	AAAGTGACGTCATTTCAGAGA
Crema.WS247	Crema.WS247_Fam_291_21_1	1	21	0.000000	AGGTGAAAAATTCAAATTTAT
Crema.WS247	Crema.WS247_Fam_292_21_1	1	21	0.000000	TACAGTGACAATTTCAAAAGT
Crema.WS247	Crema.WS247_Fam_293_21_1	1	21	0.000000	ACCAGTACCTCGACTGAATCC
Crema.WS247	Crema.WS247_Fam_294_21_1	1	21	0.000000	AAGATTACTGTAGATGACCAG
Crema.WS247	Crema.WS247_Fam_295_21_1	1	21	0.000000	GCGTCGCAGTTTTCGCTAGAC
Crema.WS247	Crema.WS247_Fam_296_21_1	1	21	0.000000	CTGGAACCCCTGATTCTGAGC
Crema.WS247	Crema.WS247_Fam_297_21_1	1	21	0.000000	TAAGCAGCTCCCAACGAGCCC
Crema.WS247	Crema.WS247_Fam_298_21_1	1	21	0.000000	CACAAGTTGTAGCCAATAAAA
Crema.WS247	Crema.WS247_Fam_299_21_1	1	21	0.000000	GGCTGAGCTTGTTTCGAAAAG
Crema.WS247	Crema.WS247_Fam_300_21_1	1	21	0.000000	ACTTCTGGTACTGGACCATCT
Crema.WS247	Crema.WS247_Fam_301_20_1	1	20	0.000000	TGGAAAGTTCTTTCGAAATT
Crema.WS247	Crema.WS247_Fam_302_20_1	1	20	0.000000	GTCGATTTACGGAGCCTCGG
Crema.WS247	Crema.WS247_Fam_303_20_1	1	20	0.000000	ACTCAACTTTTGGCAATGCG
Crema.WS247	Crema.WS247_Fam_304_20_1	1	20	0.000000	ATAGGTCCCCACATCCAATA
Crema.WS247	Crema.WS247_Fam_305_20_1	1	20	0.000000	GGTGGCCTAGTTTCCCAATA
Crema.WS247	Crema.WS247_Fam_306_20_1	1	20	0.000000	ACGCCTAAAATGAGCCTGAA
Crema.WS247	Crema.WS247_Fam_307_20_1	1	20	0.000000	GGAAAAAGCTTAAATTTGAT
Crema.WS247	Crema.WS247_Fam_308_20_1	1	20	0.000000	TGTCCGCTTTTTCGAAGATG
Crema.WS247	Crema.WS247_Fam_309_20_1	1	20	0.000000	CAGTAATCTAGGAATATCTA
Crema.WS247	Crema.WS247_Fam_310_20_1	1	20	0.000000	TTCATTCGCTGCGGCGTCAT
Crema.WS247	Crema.WS247_Fam_311_20_1	1	20	0.000000	CATCAGACAGATTCCTCGGC
Crema.WS247	Crema.WS247_Fam_312_20_1	1	20	0.000000	TGCGTCAAAAAGTACGCCGC
Crema.WS247	Crema.WS247_Fam_313_20_1	1	20	0.000000	ACCTCAAAAATTGGGCTGAG
Crema.WS247	Crema.WS247_Fam_314_20_1	1	20	0.000000	GCTCACTAGAGCGTCAAAAA
Crema.WS247	Crema.WS247_Fam_315_19_1	1	19	0.000000	ACGGGGGTTTTAAGAATAG
Crema.WS247	Crema.WS247_Fam_316_19_1	1	19	0.000000	GAACGGTCCCAAGGAATTA
Crema.WS247	Crema.WS247_Fam_317_19_1	1	19	0.000000	GAATGGTCCCAACCGCAAA
Crema.WS247	Crema.WS247_Fam_318_19_1	1	19	0.000000	ATTCCTCCTTTTTTTTCTC
Crema.WS247	Crema.WS247_Fam_319_19_1	1	19	0.000000	TAGAGTACCATTACATCAC
Crema.WS247	Crema.WS247_Fam_320_19_1	1	19	0.000000	TCTTCTTAACTCTTCTTCC

Crema.WS247	Crema.WS247_Fam_321_18_1	1	18	0.000000	GGATACGGTGGATATGGA
Crema.WS247	Crema.WS247_Fam_322_18_1	1	18	0.000000	CCTTCCAATCCAGGAACT
Crema.WS247	Crema.WS247_Fam_323_18_1	1	18	0.000000	CTCTCCTGTTTTTAGTCC
Crema.WS247	Crema.WS247_Fam_324_18_1	1	18	0.000000	GATTTCCAGGACGATCAG
Crema.WS247	Crema.WS247_Fam_325_18_1	1	18	0.000000	GGCGGAGCTTGTTGAGGG
Crema.WS247	Crema.WS247_Fam_326_18_1	1	18	0.000000	GCCAAGTACAGTGACCAC
Crema.WS247	Crema.WS247_Fam_327_18_1	1	18	0.000000	TAGAATCGCTAGCATCGC
Crema.WS247	Crema.WS247_Fam_328_17_1	1	17	0.000000	TCGAGTCTCGTCTGGCC
Crema.WS247	Crema.WS247_Fam_329_17_1	1	17	0.000000	CTCTTTGACTACACTTG
Crema.WS247	Crema.WS247_Fam_330_16_1	1	16	0.000000	TTAGGCTTAGCTGATC
Crema.WS247	Crema.WS247_Fam_331_16_1	1	16	0.000000	TGCGAGTTTTTGGGTT
Crema.WS247	Crema.WS247_Fam_332_16_1	1	16	0.000000	CGGGTTTGAATCCTCC
Crema.WS247	Crema.WS247_Fam_333_16_1	1	16	0.000000	TCGGAGGGTCGGAGGA
Crema.WS247	Crema.WS247_Fam_334_15_1	1	15	0.000000	TATCAGTTATCACTT
Crema.WS247	Crema.WS247_Fam_335_15_1	1	15	0.000000	CAGTGCAACCGAAAT
Crema.WS247	Crema.WS247_Fam_336_15_1	1	15	0.000000	CATTTCGGCACCAAC
Crema.WS247	Crema.WS247_Fam_337_15_1	1	15	0.000000	AATCTGAATCTTCAG
Crema.WS247	Crema.WS247_Fam_338_15_1	1	15	0.000000	GATTACTGTATCTGG
Crema.WS247	Crema.WS247_Fam_339_15_1	1	15	0.000000	GATAAGGAAGCAAAT
Crema.WS247	Crema.WS247_Fam_340_15_1	1	15	0.000000	CGAAAAATTTTGAGG
Crema.WS247	Crema.WS247_Fam_341_14_1	1	14	0.000000	AAGTTAGGCCACCA
Crema.WS247	Crema.WS247_Fam_342_14_1	1	14	0.000000	CCCATTCTAATTGT
Crema.WS247	Crema.WS247_Fam_343_11_1	1	11	0.000000	TATCGTAATCC
Crema.WS247	Crema.WS247_Fam_344_11_1	1	11	0.000000	GACCACGATTC
Crema.WS247	Crema.WS247_Fam_345_10_1	1	10	0.000000	CGAGACTCAA
Crema.WS247	Crema.WS247_Fam_346_10_1	1	10	0.000000	ATTCTTGTGC
Crema.WS247	Crema.WS247_Fam_347_10_1	1	10	0.000000	TCGCTATCTT

M. hapla

Genome	Family name	Number of sequences	Alignment length	Score	Consensus without gaps
Mhapla.WS247	Mhapla.WS247_Fam_1_17_42	42	18	0.747688	GAATAnTGAAGCAgGtg
Mhapla.WS247	Mhapla.WS247_Fam_2_24_30	30	24	0.947893	CAGTCCGTTCAGTGtCgTCAACTT
Mhapla.WS247	Mhapla.WS247_Fam_3_19_17	17	24	0.721763	TGTCAATCAAACaTGTACc
Mhapla.WS247	Mhapla.WS247_Fam_4_14_16	16	24	0.348322	ATTAAAatTAAAna
Mhapla.WS247	Mhapla.WS247_Fam_5_39_13	13	42	0.781237	TGCGTGCTGGACTGCACtaAAGGCAGCAGCAtCaACAnA
Mhapla.WS247	Mhapla.WS247_Fam_6_21_13	13	31	0.466915	TTCAcaCTACcnnATGTTCCc
Mhapla.WS247	Mhapla.WS247_Fam_7_16_11	11	16	0.863636	CCAAGgTTTAAAAgGC
Mhapla.WS247	Mhapla.WS247_Fam_8_182_9	9	183	0.811374	ATAGGCCTATATTGACAGAAAAAATtAAGAAATTTTATCGTTTAGAAACGGatTTATaGGGCCTAGAanTTTTcAAtnCaaAAATTTTCaTTgaAactTCAGAGTCCTCTGACTCAAAAGGCAATATCTCTGCCAAAAATGCggAAAccTaTnTTGAAAATtTgagAtATTTTAgAnAAGAT
Mhapla.WS247	Mhapla.WS247_Fam_9_61_8	8	62	0.802035	CTGCATTAtgGGATGGATGCaAaTCTCGGGCCTTCGGCCCTCgAACCaCACTCATTTACTT
Mhapla.WS247	Mhapla.WS247_Fam_10_12_8	8	13	0.673077	aAaaTTaTTTTA
Mhapla.WS247	Mhapla.WS247_Fam_11_169_7	7	177	0.716348	GaCCTATAAAAATGCTTTGAGacGATAaAATTTCTTtATTTTTTCTtcAAAGaTgGATtAGAGTCTTATcTACaatATcCCaAAAtTTTgAGAtAGgttTcCGCATgTTTGGatGAGATATaGcCTTTAGAGTCTGaTGtatcagaAAtttTCTccgAAAAActTcTAG
Mhapla.WS247	Mhapla.WS247_Fam_12_62_7	7	88	0.509109	aAAAATCAATAAATCGATATATcGATTTTTcAAAtTTGTaAAAaTTTTTcaAAAAATCgATA
Mhapla.WS247	Mhapla.WS247_Fam_13_55_7	7	57	0.788917	CTGGCTAGATGGAGgGCTTcgTnGTGCTcCAGTATGGACAGgGGGATTtATTGCn
Mhapla.WS247	Mhapla.WS247_Fam_14_37_7	7	37	0.747748	TTcGgACTAgaAAgTAGGCTGGAagGAACATGTTCCC
Mhapla.WS247	Mhapla.WS247_Fam_15_180_6	6	185	0.814114	TTCAGAGTTACCTGACTCTAAAGGcCATAACTCAGCCAAAAATGCTAAAATCTCCTTGAAATTTTGaGATATCTTAGATAAGATGTAgAtCTATATTGATACAAAAAATCAAGAAAtGTTATCGATTTAAAACATTTTTATAGGCCaTAGAAgTTTTgATAggAGaAGAACATaTtTAAT
Mhapla.WS247	Mhapla.WS247_Fam_16_21_6	6	21	0.919577	ATATTCTGGTCAGTTCTGaTC
Mhapla.WS247	Mhapla.WS247_Fam_17_14_6	6	18	0.588272	naAATTCTGATTTT
Mhapla.WS247	Mhapla.WS247_Fam_18_177_5	5	184	0.770833	TCTAAaGGcCATAACTCATCCAAAAATATtAATATCTCCTTGAAATTTtGgGACATCTTAGAtAAGATaTAgAtCTATaTTtAagCcAAAAATaaagAAgctgAGacATaCcGaAAAaTTTTTATAGGGCCTAgAatTTTTaaTAgGAGaatTCATAtAatttnaaggtctcnagac
Mhapla.WS247	Mhapla.WS247_Fam_19_20_5	5	25	0.433333	AAtnTtctAAAntATAAtaa
Mhapla.WS247	Mhapla.WS247_Fam_20_20_5	5	20	0.853333	AaTTgTAAGaATTTaTAAAT
Mhapla.WS247	Mhapla.WS247_Fam_21_180_4	4	186	0.742234	aAaATTTCGAGGTAaccaGACTCTAaaaTCAATATCaCgAAAATTCCtCCTACgAtTTtaaTgtAAaTTTGGGATATtGTAGgtAAGAcATTcaTccATCTATaaACAAAAAATtAagAAATTTCACTTTCAAAaTTTtGATTTaTaGGggTTTGAAgaTtTtATTAGGCtaAAAtaatt
Mhapla.WS247	Mhapla.WS247_Fam_22_177_4	4	177	0.893283	CgTAAATaGGCACAAACaTaCTAATaAaCGTAACTTAAAGCGTTATAACGCgAaTTCTAGCGTTTAaATcTCtTAGtAAATTgGgGATATtTTAGAgAAGACTaTAAtcAGaATTTATTCAAAAAATTAgaAAGATCtAAACCaCCAAaTTTTTGTAATCGTTACtGAAGTTTtTAG
Mhapla.WS247	Mhapla.WS247_Fam_23_158_4	4	158	0.992968	AAAATTTTTCGTTCATTTCAAAATGTCAAAAATTTCATGAAATTCCATTCGAtTGAAATTTTTTAAAATTTATTAGAATCAAATTATTTTATGATATTTATTTTGCCAATGAGTTCGGCAAGATAATTCTATAAGAATTTCCTAGAATTTCAAAGAAA
Mhapla.WS247	Mhapla.WS247_Fam_24_88_4	4	88	0.929293	GAGGAAAACCAtTGAATTCTaTTTTATCaGaAATATgTAGTCTAGAACGTTAAAATTTAaTCGAAATTTTTAGAaaCTGAAGACAAAT
Mhapla.WS247	Mhapla.WS247_Fam_25_59_4	4	61	0.819672	GCAGCCCTACTGACGCTTTTGTCAGGGGCaTaATTCaTGGTGGcttgTgcTCAATGGTG
Mhapla.WS247	Mhapla.WS247_Fam_26_40_4	4	40	0.966667	TTAATCATTATCtTCTATTCAACGGATAATAGGGcTAATT
Mhapla.WS247	Mhapla.WS247_Fam_27_31_4	4	32	0.828125	TTCATTATTCaaCtTGTTTCacTTgCCTTGC
Mhapla.WS247	Mhapla.WS247_Fam_28_32_4	4	32	0.958333	AGgGaCAGGACTCTAATGGTTTTTAATCTTAA
Mhapla.WS247	Mhapla.WS247_Fam_29_23_4	4	24	0.817130	AAAGCAACCGAgGCtCCCGGgAc
Mhapla.WS247	Mhapla.WS247_Fam_30_19_4	4	22	0.545455	AAgTTTATTTttaTAaTTc
Mhapla.WS247	Mhapla.WS247_Fam_31_18_4	4	18	0.702160	tTagagAGaGGGTAAtTA
Mhapla.WS247	Mhapla.WS247_Fam_32_11_4	4	14	0.551587	AAAnAAcACAA
Mhapla.WS247	Mhapla.WS247_Fam_33_11_4	4	11	0.898990	AGAATTACTCt
Mhapla.WS247	Mhapla.WS247_Fam_34_78_3	3	87	0.837803	TCGTCCAAATTGTTATAGAAGAAGCCTtTCTTCGACAAGTTGTGGAGTATTATGTCGTCGATTTcTCAAGCTTcAGAC
Mhapla.WS247	Mhapla.WS247_Fam_35_86_3	3	86	0.886305	CATAAATGAACAATAATATAAAATtaAtTgGCcTAgTCtTATGAATTATcTTACAAAAATAATATCTTCTAATAAATTAaAAGtCg

Mhapla.WS247 Mhapla.WS247_Fam_36_84_3 3 84 1.000000
CCTCGAAGATGGGATTTGTTTATAGGAATCCATTCGATTTTGGGGTTTTATATTAAAAATCATCCATGAAATAACCGGTAAAAC
Mhapla.WS247 Mhapla.WS247_Fam_37_74_3 3 74 0.963964
ATGATtAAGATAGATTAGATATtTTAGGgCCAAATAAATAGAAAGAGATTAGAATGATAATGACCATAAGAAGA
Mhapla.WS247 Mhapla.WS247_Fam_38_68_3 3 69 0.906602 TTAGAGTCTtATCTACAATATCCCAAAATTTTGAgggAGGTTTCCGCATGTTTGGAaGAGATATAgCC
Mhapla.WS247 Mhapla.WS247_Fam_39_62_3 3 68 0.846405 TAATTTGAAAACAAACTTTAACACATcCTcTAATAATAAATTACAATTCCGtAAAGCATTTT
Mhapla.WS247 Mhapla.WS247_Fam_40_64_3 3 64 0.798611 TGTATATCAATTATCAATATATTTTTaaATTaaAGGTATAAATATCAATGTAtgttatcaaaaa
Mhapla.WS247 Mhapla.WS247_Fam_41_56_3 3 56 0.984127 CAAATaTATTATGGCATTTTGGCCTCTTCGGTGGCCATAAAAATTTTTCGTTTTGC
Mhapla.WS247 Mhapla.WS247_Fam_42_34_3 3 34 0.947712 TGCCCCATGCCTCAATCGGcTTGCgGGTCTTTCG
Mhapla.WS247 Mhapla.WS247_Fam_43_28_3 3 28 0.611111 AATaAatAcAAtAATCAgaaTTaATTta
Mhapla.WS247 Mhapla.WS247_Fam_44_21_3 3 27 0.588477 AaGGTGctGTTGTtGTgGTAG
Mhapla.WS247 Mhapla.WS247_Fam_45_26_3 3 26 0.803419 AtTgACGTGGCgCAGGGCTCCaGCag
Mhapla.WS247 Mhapla.WS247_Fam_46_26_3 3 26 0.965812 AAAAATCAGAATTTTTTCtGATTTTA
Mhapla.WS247 Mhapla.WS247_Fam_47_22_3 3 22 0.712121 TATTTTTGTATTTTTntAantt
Mhapla.WS247 Mhapla.WS247_Fam_48_22_3 3 22 0.656566 GGGATAAGtGGgAAnGnTAagg
Mhapla.WS247 Mhapla.WS247_Fam_49_22_3 3 22 0.878788 ATACTGTAgAttTAAAAGCCGG
Mhapla.WS247 Mhapla.WS247_Fam_50_18_3 3 20 0.727778 ntTTTTATAGGCCTAGAA
Mhapla.WS247 Mhapla.WS247_Fam_51_20_3 3 20 1.000000 AAGTTGCGTTATAACGCTTT
Mhapla.WS247 Mhapla.WS247_Fam_52_18_3 3 18 0.901235 GCCACCATTGAAcCACAa
Mhapla.WS247 Mhapla.WS247_Fam_53_18_3 3 18 0.901235 CCCATCtgAACGCTGAAA
Mhapla.WS247 Mhapla.WS247_Fam_54_16_3 3 17 0.650327 TTTTAATTTnAttCTg
Mhapla.WS247 Mhapla.WS247_Fam_55_16_3 3 17 0.647059 ATtGTAaAAcTTaTTt
Mhapla.WS247 Mhapla.WS247_Fam_56_15_3 3 16 0.701389 AAACAaGTTTTnAAa
Mhapla.WS247 Mhapla.WS247_Fam_57_15_3 3 16 0.729167 AtAaTTTTAAgCTTT
Mhapla.WS247 Mhapla.WS247_Fam_58_16_3 3 16 1.000000 TTAAGATAAAGCCATT
Mhapla.WS247 Mhapla.WS247_Fam_59_11_3 3 11 0.848485 AtAATTTAATt
Mhapla.WS247 Mhapla.WS247_Fam_60_95_2 2 95 0.901754
ATCCCATCGAATAAAAGGGaaaATTgATCGATGAATGAAGTTCCATGTATGAATTAGTATGACCTTCATgATCAACTTTTaCATTTCTAGAAATa
Mhapla.WS247 Mhapla.WS247_Fam_61_94_2 2 94 0.702128
AGATTTTATTAATTAAAATTATATTTTTAAAATAATACGAGGTAATAAATAAAAATACTAAACACTTTATaattttattttcgagggtaaattt
Mhapla.WS247 Mhapla.WS247_Fam_62_89_2 2 89 1.000000
GAATAGACGATAAAATTGCAGTCGTTAAGTAGTAGATTTAAAATAATTTGTCTCATAGAACTAATGATTAGTACTAATGATAAATTTAC
Mhapla.WS247 Mhapla.WS247_Fam_63_78_2 2 78 0.965812
TTAATAATAAGTTAAACATTAGAAGCCTAGTTAATATATTTACTTTTTTCTGATTCTGTTTCATTAGATACTGGtTAAc
Mhapla.WS247 Mhapla.WS247_Fam_64_76_2 2 76 0.929825
CAGTGCaAATGCcTACCTAGGATTTCTAGATTTTTaATCTATGACAATTCTTTCAATTATTAGGACATATATAGTG
Mhapla.WS247 Mhapla.WS247_Fam_65_76_2 2 76 0.929825
GTTTATACAATTCATATATATTTgGTAGAATTAGATCTCTATATAGAACAaATCATaACTTGTTAGCTTCaACTTC
Mhapla.WS247 Mhapla.WS247_Fam_66_72_2 2 72 0.953704 CAAGTGATATAATAAAATTAAGTGAGATAaATGAGGGTTCTTAAGcCCAAGAGCTAAGCAAGACTCGTTTTT
Mhapla.WS247 Mhapla.WS247_Fam_67_67_2 2 67 1.000000 AGAATAATTTCGACAAGCAATAAGTGCTAACCTAAAGATAATGGCACTTTCTGATTTATCAAAAATC
Mhapla.WS247 Mhapla.WS247_Fam_68_66_2 2 66 1.000000 TAATTTTTTACAATTTCTGCCGTAGCAATACTTAAGTTTATTTTTTCTTCGACAAGTTTACAATTG
Mhapla.WS247 Mhapla.WS247_Fam_69_62_2 2 62 0.956989 AAATTTTAGATAAaACGTTACATAAACGCGAAATCTATTCGCgAAACTTTGATTACTAACCT
Mhapla.WS247 Mhapla.WS247_Fam_70_62_2 2 62 0.935484 CGCAAGGAGGACGAAGACAAATGACGCTTCTAAATGATGaATAAAAGaCGTTATAATTAGGa
Mhapla.WS247 Mhapla.WS247_Fam_71_60_2 2 60 0.822222 GGCTTCGGCCAACTGCCAGCAgGGGGGttcTTCTTTCTCTcTTcCCCCCTGCAAGCAGaT
Mhapla.WS247 Mhapla.WS247_Fam_72_60_2 2 60 0.955556 GATGGTTCAGCAGAAGTTCCTcTCTTCTTGTCCaTGCTCCGAGAACGAGAAGACTTTTTT
Mhapla.WS247 Mhapla.WS247_Fam_73_57_2 2 57 0.859649 TATAAgGCACTGTAGaAAATcTTCTACTAACTTAAACGtTTACCgGTTTGCCATGGC
Mhapla.WS247 Mhapla.WS247_Fam_74_54_2 2 54 1.000000 AATATCTGGCTTTCAGCCTTTTTATGAAGCTGCTTCCTCTTCTGGAAGCTTCAA
Mhapla.WS247 Mhapla.WS247_Fam_75_54_2 2 54 1.000000 ATACATAGACCCTCAGGAATCTATGTATTTTCGGTATATTCTAACGATTACAAA
Mhapla.WS247 Mhapla.WS247_Fam_76_53_2 2 53 1.000000 TTTTATATAATTAAAGATTTTCTTTCTATATTTAAAATATATTTAAGTTTAGA
Mhapla.WS247 Mhapla.WS247_Fam_77_48_2 2 48 0.833333 CGAAAGCTTATGCGTGCCAaAATCAATaCcTGCTAAAATCAAAaaCTa
Mhapla.WS247 Mhapla.WS247_Fam_78_48_2 2 48 0.916667 TTCACTTGCCTTGCTTCATTATTCCaCCTGTTTCATaATTCAACTTGc
Mhapla.WS247 Mhapla.WS247_Fam_79_42_2 2 42 0.603175 TTTACAAATTGTAAAAaGTTTTaAttaAAgcTTTaaTTATaa
Mhapla.WS247 Mhapla.WS247_Fam_80_42_2 2 42 0.888889 CAGTAATTTTGAAGCATGCTTcAAaTTTTTGTAAACATGaTT
Mhapla.WS247 Mhapla.WS247_Fam_81_42_2 2 42 1.000000 GAAAATAATATATAATCAGTTAGTACAAAATTCGTACTAACA
Mhapla.WS247 Mhapla.WS247_Fam_82_41_2 2 41 1.000000 TCTTAATTTTAATTTATTAAAATAAAAACATATCTTCTCTC
Mhapla.WS247 Mhapla.WS247_Fam_83_40_2 2 40 0.608333 TAATTaagAAATAtaTTCCGGAAAACaCATaCTaAaATtt
Mhapla.WS247 Mhapla.WS247_Fam_84_40_2 2 40 0.800000 CCTATaTTGAaAATCTGagATATTTTAGaTAAGATATAGg

Mhapla.WS247	Mhapla.WS247_Fam_85_37_2	2	37	0.639640	ATTCTGaTTTTaatGtTTTAAaAaaTGTTTTaATTTA
Mhapla.WS247	Mhapla.WS247_Fam_86_37_2	2	37	0.639640	GAAAAcaTTATTaTAtTTAAATaAAAaAaaTTTTaCG
Mhapla.WS247	Mhapla.WS247_Fam_87_36_2	2	36	0.611111	AAATCAGAATTTCaTgCAaACAgTAaTgAAttaAgA
Mhapla.WS247	Mhapla.WS247_Fam_88_36_2	2	36	0.962963	TTAACTTTTCcGGAAAATACATTCTGAAATTAATCT
Mhapla.WS247	Mhapla.WS247_Fam_89_33_2	2	33	0.621212	tTTTGcTAtTTTTcTATTTTTcACAAAaCaGAa
Mhapla.WS247	Mhapla.WS247_Fam_90_33_2	2	33	1.000000	ATCAGAATTAAAACAAACTGTAGAAAAATAAAT
Mhapla.WS247	Mhapla.WS247_Fam_91_32_2	2	32	0.656250	ttTTCCGGAAAaTaaTTaAAaaTAATTCTGAC
Mhapla.WS247	Mhapla.WS247_Fam_92_32_2	2	32	0.666667	aTACAAACcTTCCGGaAAATATTaTaaTTAAa
Mhapla.WS247	Mhapla.WS247_Fam_93_32_2	2	32	1.000000	TTACCGAACGAACGGGCAGGAAGAAATCTCCC
Mhapla.WS247	Mhapla.WS247_Fam_94_31_2	2	31	0.612903	TACAAaTTGTAAAAaaaTTTaTTAaTAAaac
Mhapla.WS247	Mhapla.WS247_Fam_95_30_2	2	30	1.000000	ATAATTTAAAAAATTTACTAATTTTTTAA
Mhapla.WS247	Mhapla.WS247_Fam_96_29_2	2	29	0.655172	AAgTTTTAcAAATAAATaaTTTTTAggAA
Mhapla.WS247	Mhapla.WS247_Fam_97_28_2	2	28	0.678571	AAAAtACAAAAATACaAATAAaCTgGga
Mhapla.WS247	Mhapla.WS247_Fam_98_27_2	2	27	0.703704	TTGAcaTTTTGTaATATTTTgGccTAT
Mhapla.WS247	Mhapla.WS247_Fam_99_26_2	2	26	0.679487	TTAAATAAACTcGAAgcAAAAcTTtg
Mhapla.WS247	Mhapla.WS247_Fam_100_25_2	2	25	0.600000	TTTTcagTAATTaaGCTTTaAATGa
Mhapla.WS247	Mhapla.WS247_Fam_101_24_2	2	24	0.625000	TTACAAaAATATTTaaTTAcaAaa
Mhapla.WS247	Mhapla.WS247_Fam_102_24_2	2	24	0.888889	GGGTCACAaTATCCaGAGAAACCC
Mhapla.WS247	Mhapla.WS247_Fam_103_23_2	2	23	0.666667	aaAATTcTGAAAAAcTTgGGAAa
Mhapla.WS247	Mhapla.WS247_Fam_104_23_2	2	23	0.688406	ATTCTgATTTTcGgTTTTAATca
Mhapla.WS247	Mhapla.WS247_Fam_105_23_2	2	23	0.688406	aAGAAtaAAATTTTAGAAaAAAt
Mhapla.WS247	Mhapla.WS247_Fam_106_22_2	2	22	0.689394	aaATCAGAATTaTTTaAGAAGa
Mhapla.WS247	Mhapla.WS247_Fam_107_22_2	2	22	0.742424	TTcTGAtTTTTAAAAATTAAaa
Mhapla.WS247	Mhapla.WS247_Fam_108_22_2	2	22	0.636364	TTgTTTTATTTAtTTAAaaaAA
Mhapla.WS247	Mhapla.WS247_Fam_109_22_2	2	22	0.727273	AaGGAACATCCatCTAGTCTGa
Mhapla.WS247	Mhapla.WS247_Fam_110_22_2	2	22	0.613636	gGAAGCATGCTTCaAaaTTaCg
Mhapla.WS247	Mhapla.WS247_Fam_111_22_2	2	22	0.939394	GGCCTcCGGCCCTCGACTTCTC
Mhapla.WS247	Mhapla.WS247_Fam_112_21_2	2	21	0.698413	AATTCTGATgTTTTTaAaCaa
Mhapla.WS247	Mhapla.WS247_Fam_113_20_2	2	20	0.641667	CAAcaGAAaATAcAAGACTa
Mhapla.WS247	Mhapla.WS247_Fam_114_19_2	2	19	0.684211	TATTTTTGaAgaAAAgAAT
Mhapla.WS247	Mhapla.WS247_Fam_115_19_2	2	19	1.000000	AAATGTTTCCGGAAAAAAG
Mhapla.WS247	Mhapla.WS247_Fam_116_18_2	2	18	0.611111	TAAACTTGAAaAcTgAag
Mhapla.WS247	Mhapla.WS247_Fam_117_18_2	2	18	0.666667	AaTTAtTTAaTAAACaAG
Mhapla.WS247	Mhapla.WS247_Fam_118_18_2	2	18	0.851852	CTACACCaCCAaCGGGAT
Mhapla.WS247	Mhapla.WS247_Fam_119_16_2	2	16	0.708333	TaTTATAAaGAGTGTc
Mhapla.WS247	Mhapla.WS247_Fam_120_16_2	2	16	0.833333	TATAGTGAACTCcCGa
Mhapla.WS247	Mhapla.WS247_Fam_121_15_2	2	15	0.733333	AAAcTGGTACaaGAC
Mhapla.WS247	Mhapla.WS247_Fam_122_14_2	2	14	0.666667	TAGgTAAGGcAAcT
Mhapla.WS247	Mhapla.WS247_Fam_123_14_2	2	14	1.000000	ACTCTGAATCGGCT
Mhapla.WS247	Mhapla.WS247_Fam_124_13_2	2	13	1.000000	TTCGCGAAACCAA
Mhapla.WS247	Mhapla.WS247_Fam_125_12_2	2	12	1.000000	TACCGTACCGTA
Mhapla.WS247	Mhapla.WS247_Fam_126_11_2	2	11	1.000000	ATAACTTTCGC
Mhapla.WS247	Mhapla.WS247_Fam_127_10_2	2	10	0.733333	TATTTTAaTg
Mhapla.WS247	Mhapla.WS247_Fam_128_181_1	1	181	0.000000	TTTCTTTATTTTTTTATACAATATAGATCTATGTCTTATCTAACATATCCCAAAATATCTAGGAGATCGTTCGCATTTTAAGCCCCGAAATTTTTCATTTTGAGTCTGATGACTCTGAAAGATAAAAATTTTCTCCTGTTAAAAACTCTAACCCCTATAACTCAAAATTTTGAAGTTGAAA
Mhapla.WS247	Mhapla.WS247_Fam_129_179_1	1	179	0.000000	TCATAGGACTTCGGCCACACCCATTAAGTTTTGAGAAGCAATAAGAAGCTTGGTAAGCCTTTTTAGAGTCAAATATGATTAGATCTCGTGTGTATGATTTGGTATAAGAACTAGAACCGCCACCGGATCCCACAAGCTAAAGTAGTGTTTCTCTATTTAGAAGCTCCAAATATGAAAAC
Mhapla.WS247	Mhapla.WS247_Fam_130_177_1	1	177	0.000000	ACAAAAAATTAGAAAAATTTAATTTAGAAAATCGTTCCTAGAGGAGTCGGAATTTTCATTAATTTTAGAGCTTTATAATTAAAGTCAACAGACTCTAAGGGTCATTTCTCTAAAAATTGACGTCAGATCACCTAATAAATTTTGGATATTGTAGATAAGATGTATTCACATCTTTAC
Mhapla.WS247	Mhapla.WS247_Fam_131_168_1	1	168	0.000000	CCCAAGTTTTGTAAGGGAGGACGAAGTCCCTATGAGTTTTTTTGTTTTCCATCAACGCTACTTTAGCAATCAGGATCCGGGTTGCATTTCTCTTTCTTATGCTCAGTCATACACACCAAACTTATCATATTGGACTCAAGGAACACTACACAAGCTTTTTATGGCTTC
Mhapla.WS247	Mhapla.WS247_Fam_132_141_1	1	141	0.000000	AATTATAATTTAATTTTGCTAATACAATTAAATATATACTTAATATCGCTTAATTTAAATGCAAATTTATCAATATAAATTTGCATTTAAATTAACCAAATACCTATTCTAATTCTTATTATCTTTTAATTTTATTAGCAA

```
Mhapla.WS247        Mhapla.WS247_Fam_133_137_1        1        137    0.000000
TTTAACATTTGTTATTTACTTTAAATATAAATTATAAAAGTCTATTAAAATAAGAATATCGAATAAATTTTAGGTTTCTGTTCCTCATTTAGGTTATGGAATCTTCTGTATGTCGAGAATATTACTATTTTTATAGA
Mhapla.WS247        Mhapla.WS247_Fam_134_135_1        1        135    0.000000
TAATTATTTTATCTTTCTAAAACCTATGATATCAACTTATTTTTCTTCGTCCTACTTATCTTCCGAACGAATAATAAACTCTTCCATTCTCTTCATCATCCTCTTCCATCAACATCTCTTCACTATTTCTACTGT
Mhapla.WS247        Mhapla.WS247_Fam_135_120_1        1        120    0.000000
AAAATAATAAAAATAATAATAAAGAGCACACGAATAATAAAATGTTTATAATTGCTTAAGAATAAGATATTTTCTGACTAACATAACGATTTAATATTTATTCGATTTAATTATAATTAT
Mhapla.WS247        Mhapla.WS247_Fam_136_111_1        1        111    0.000000
CATCATCAGAACTATCTTCAGAAGAAGATGGTTTTCGCTTCTTTGTCGCAGGCAAAGCGTTCGCAGAAGTCTTGGTAGTAGCCTTGGCCGTAGAGTTTTTAGATGCCAAAC
Mhapla.WS247        Mhapla.WS247_Fam_137_104_1        1        104    0.000000
TGCTGAGCTACGAGCAGGCAAGGCCATGCGAGTTGCCCCGCCGACTAAGCCACGCTCGAAGGCTCGGGACCGGGAAGGAGACGCGGGTAAAACCGAAACAAGCG
Mhapla.WS247        Mhapla.WS247_Fam_138_101_1        1        101    0.000000
TTAGAGTCTGGTGTATTAGAAATTTTCTCCTGTTATACTTCTAGGATCTATAAAAATGCTTTGAGACGATAGAATTTCTTTATTTTTTCTTCAAAGATGGA
Mhapla.WS247        Mhapla.WS247_Fam_139_97_1        1        97    0.000000
GAGAACTGGCTTGATAAAACAAAACAATTTGTTGGATAATAAAATACGAATACAACTCTAATCCTTCCAAATTCATGATTAATCCAAGCTTTTCGAA
Mhapla.WS247        Mhapla.WS247_Fam_140_97_1        1        97    0.000000
TTTACTTTATCTCTCATTTTACCATGATATAGGTTTAAATAATTCAAAATACTTTTCTAAATATATAATCTAAATAAATCTTTATTATTAATACCTA
Mhapla.WS247        Mhapla.WS247_Fam_141_93_1        1        93    0.000000
CTTCAGGACCAACTGGAGCACCAACAGCAACTCCAGCTTCAACTGAATCAACAGGCTCTCCTGCTCCATCTACTTCAGCTGAAACTGGCTCAC
Mhapla.WS247        Mhapla.WS247_Fam_142_92_1        1        92    0.000000
TTCAAAATTTATAATAAAATTTTTCAAAAGACATAAAATCTTGCCTACAAAAAAATTTTTGAATTTTTATTTCTTTATTTTAAATCAGAATT
Mhapla.WS247        Mhapla.WS247_Fam_143_90_1        1        90    0.000000
AAACAAATTAGCTAAAATCAAGTTTAGCTTACAAAAAAATAAGGGACACTCCGAAAAAAGGCAGTTAATTAGAGAATTTTTTAGTATTAAA
Mhapla.WS247        Mhapla.WS247_Fam_144_84_1        1        84    0.000000
ATATTATTTTTTAGAATATAGTGCTAATGCACTATCAGAACCACTATTTTTCTTCTATCTTTTTCAATTTTTGTATTCTCTCTA
Mhapla.WS247        Mhapla.WS247_Fam_145_82_1        1        82    0.000000
TTAATAAATAAACGACTAGATTTGTCTGATGGAAACTCGAAACTAATTAGTTTCGTAAAGTTTCGAATAAGAAATAATCAAA
Mhapla.WS247        Mhapla.WS247_Fam_146_81_1        1        81    0.000000
CTCTAGAGGGAACTCCCTAAATAGTGAAACTCTCCATATTATGAACTTCTTTTAAAGTTCCCATGCCTCAACTAGAGGGGA
Mhapla.WS247        Mhapla.WS247_Fam_147_79_1        1        79    0.000000
AATTGGATATCCCTACCCCTATATTCTTAGGGTTACAAATCCCTAATTTAGGTGACTTTTGAGTATTATGCCCCTTACA
Mhapla.WS247        Mhapla.WS247_Fam_148_78_1        1        78    0.000000
TCCATCTACACAAAAGACAGGAGGAGTGGCATCGAACAAGCCCCCTGCAGGATTGCCATCTACCGGACGGCCAGAAAC
Mhapla.WS247        Mhapla.WS247_Fam_149_77_1        1        77    0.000000
ATTTTTTTAGGGTTAAATCCCGAATTTTTCATTTTTATTCTGATTTATTAAAATCAGAATTTTTTATTTTATTTAAA
Mhapla.WS247        Mhapla.WS247_Fam_150_76_1        1        76    0.000000
GATTTATTAAGAATTAGCCTACACCAATAAAATTTATTTTTTATTATAACATTATTTTCAATTCGGAAGTTTGATA
Mhapla.WS247        Mhapla.WS247_Fam_151_76_1        1        76    0.000000
ATTAATTCCAAAAGGATATATCCAAATAAATGATTCGGAACAAAATTTGAAAAAATTTTACGTCTCAAAAACCAGA
Mhapla.WS247        Mhapla.WS247_Fam_152_75_1        1        75    0.000000
AGAAACGATTCTTTAGGTAGTTTATTGTGGGATAATAAAAGAAATGTTAGATAGTTTGAGATTAAAGAAGAAAAA
Mhapla.WS247        Mhapla.WS247_Fam_153_74_1        1        74    0.000000
TTTGTCGTTTATGTAGAGAGTGTGTGAATTAGAGAGTAAAAGGAATCAATTTTCTTGCGTGGATTCTGAGAGGT
Mhapla.WS247        Mhapla.WS247_Fam_154_74_1        1        74    0.000000
CCCTAATCTCGGTAAAACAATCCCATTTCTTTACCTACGATTGGGTCTCTATAGGTATCAAGGGTTACAATGGT
Mhapla.WS247        Mhapla.WS247_Fam_155_74_1        1        74    0.000000
CGAAATTTAACTAACGAGAAAATGATATAATGCAACAGAAGACAAATCCTACAACTACATTAGGAATCATTCGC
Mhapla.WS247        Mhapla.WS247_Fam_156_73_1        1        73    0.000000
GTGAAGATCATCTTATAGCCTCTCTAAGGTCGCATCTGGATAATTTATATTGATCATTTCTCGTCTTTTTATT
Mhapla.WS247        Mhapla.WS247_Fam_157_72_1        1        72    0.000000        TGGAGTTGAAGGAGTATTTGGTCCAATTCTTGTCGTTCGAGGTTCTAAAGTGGTCGAGGATGTTCCGTTTGC
Mhapla.WS247        Mhapla.WS247_Fam_158_72_1        1        72    0.000000        ACAAAACTTCGCTAAATGCTGACCATCAATTTGAAAAATCAAGCAACATAATACGAATCAACCAAATAAACA
Mhapla.WS247        Mhapla.WS247_Fam_159_71_1        1        71    0.000000        TCTTTAAATTGAAAACTTTTTTTATTCTCACCGCGCCTTTTTTGGTCTCTCCAGTTTCTTCTGATTCGACC
Mhapla.WS247        Mhapla.WS247_Fam_160_69_1        1        69    0.000000        AAATAAACTCATTTGATCGGTCAATTCTTTTTTCAATTCATCTTTGATCTCCTTTTTCAGTTGTTGTCC
Mhapla.WS247        Mhapla.WS247_Fam_161_68_1        1        68    0.000000        AAATAATTTTCTAAAAAAATTGAAAAAAATTTCGAGGATTTATTGGATCTATCAAAAAATGGAATTAA
Mhapla.WS247        Mhapla.WS247_Fam_162_68_1        1        68    0.000000        GGGATTTCGCACTAATGTAATACTAGTATCAAAGTGAGACGAATAAACATCTTTTGGATAGATGAGAG
Mhapla.WS247        Mhapla.WS247_Fam_163_67_1        1        67    0.000000        AAATACAATAGCCCGAACTGCTATAGATACAGTCGGGCTTATTTACGGGTATACTGTACATATCAAG
Mhapla.WS247        Mhapla.WS247_Fam_164_67_1        1        67    0.000000        TATTAGTTTAGAGATGTAATGCCATATAATAGGATTTAAGGACAAGACACTGAAAAATAGAATAGCG
Mhapla.WS247        Mhapla.WS247_Fam_165_66_1        1        66    0.000000        GTCAAACAATATAATGATACTAAATGTCATAAATTAATCCATAAATCGTTGCAATTCTGTGGAAAC
```

Mhapla.WS247	Mhapla.WS247_Fam_166_65_1	1	65	0.000000	TATTTTCATATATTTATATTTTTTAAATTTTAATTTTTTAATTAAATAAATAATATTTTTTTTAT
Mhapla.WS247	Mhapla.WS247_Fam_167_64_1	1	64	0.000000	AGAATTACAACTAAAGTTGGGATCCCCTAAACTAGAGGGTAAACCCTTTGACAAATAATCCTTT
Mhapla.WS247	Mhapla.WS247_Fam_168_64_1	1	64	0.000000	AAGAATAATTTACACGAATATCTTCGAATGTCATGATATGACATAATAACATTTGCACGATTTT
Mhapla.WS247	Mhapla.WS247_Fam_169_64_1	1	64	0.000000	ATTTATTTTTGGACAATCTAGATAAATTCTTTTGATTCTATCTGCTTATCTGTCGAAGTCCTTT
Mhapla.WS247	Mhapla.WS247_Fam_170_63_1	1	63	0.000000	TAAAATTTTGAGGTAACCATACTCTAACGGCAATTTCTCGAAAATTCCGCCTACGATTTTCTT
Mhapla.WS247	Mhapla.WS247_Fam_171_63_1	1	63	0.000000	TTACATACAGGGGGGCCTGCGCTGCGTTTTTGGTGCCAATTCTTGGCATGACCTCTTAAATTT
Mhapla.WS247	Mhapla.WS247_Fam_172_63_1	1	63	0.000000	TTGTTCGTCTTTCAATGTTTATTTTTCTTCTGAATTTTGAAGAGTTTTCTTTATCTATTGTTA
Mhapla.WS247	Mhapla.WS247_Fam_173_62_1	1	62	0.000000	TCGAAGAAAAACAACCTGGATGTATTGTCAATAGCAATAGATAACAAAAAATCGTCAAAAAT
Mhapla.WS247	Mhapla.WS247_Fam_174_62_1	1	62	0.000000	GATAAAAAATGGCAAAAATCAGAATTAAAATAAAGGTTATCGATACTCTAATAGTCCTGCTC
Mhapla.WS247	Mhapla.WS247_Fam_175_58_1	1	58	0.000000	TTTCCGGAAAAGTTTAAATGTTTTTTGTGTTTAAAAAGTTTTATAATTTTCAGAATTT
Mhapla.WS247	Mhapla.WS247_Fam_176_58_1	1	58	0.000000	AATTAATGTAAAAGAATTTAAAATAAAATTTCAGAGTTCCGAAAATAATATTTTCTGA
Mhapla.WS247	Mhapla.WS247_Fam_177_57_1	1	57	0.000000	TATTCTATTACTTGAGAATATGACATAAATCAAATTATGGAGTCAGTGCATATGTCA
Mhapla.WS247	Mhapla.WS247_Fam_178_56_1	1	56	0.000000	GTTTTGCCGTCCGTCTACCAAAGCTTTGTTCTATTCGGACAGCTTTTCACGTTACC
Mhapla.WS247	Mhapla.WS247_Fam_179_56_1	1	56	0.000000	TATATTTAAAAAAATAATCAGAATTTTACTTAATATTTTATTAATAAATTGGCTTT
Mhapla.WS247	Mhapla.WS247_Fam_180_55_1	1	55	0.000000	TATGGCAATGATCTAGAATGGTTATACCTCTTTTAATACACTGCCATTCCCGGTC
Mhapla.WS247	Mhapla.WS247_Fam_181_54_1	1	54	0.000000	ATACTAACCCTGGAAACTGAGAGGCCTTTCGAATAGAACTAATCCTCCTATATG
Mhapla.WS247	Mhapla.WS247_Fam_182_54_1	1	54	0.000000	AATATTTTCCGGAAGATGTTTTTATTGTTTTCCGGACAATTAATATTTATATTA
Mhapla.WS247	Mhapla.WS247_Fam_183_53_1	1	53	0.000000	AAATTTTAATAGAAGTTTCCGGAAAAAAATATAACTTTACAAATATATAATTAC
Mhapla.WS247	Mhapla.WS247_Fam_184_51_1	1	51	0.000000	AGGACAAGGACATTCTTCAAGTGAATCGAAGGAACACACACCAAAACCTTC
Mhapla.WS247	Mhapla.WS247_Fam_185_50_1	1	50	0.000000	AGAAACGATTAAATAAATTTATTCGTGAATTTTCTTATTAGAAGTCGATG
Mhapla.WS247	Mhapla.WS247_Fam_186_49_1	1	49	0.000000	TAAACATGTTTTTAACATCAAAACAAGTTTTAATAATCAGAATTTATTC
Mhapla.WS247	Mhapla.WS247_Fam_187_48_1	1	48	0.000000	CTTTATAAAAAACACACCTTATAACCCCCTCCCTTTACAAGCCACTTC
Mhapla.WS247	Mhapla.WS247_Fam_188_48_1	1	48	0.000000	GAATTAGAAGCTAAAAAGAAGTCTGAACCAGAAGCAAAGAAAAAAGCT
Mhapla.WS247	Mhapla.WS247_Fam_189_48_1	1	48	0.000000	CTTCCTCCTATCCTTCCACTTCCCTCCATTCTTCTCTCCATTCCACCT
Mhapla.WS247	Mhapla.WS247_Fam_190_48_1	1	48	0.000000	AGGACAATTCACCGTTCGACTTTTCACCTAATAGGATATTTCACCTAT
Mhapla.WS247	Mhapla.WS247_Fam_191_46_1	1	46	0.000000	TCAAAATTTTATGTTTTCTCGCCTAAAAAATTCTTACCCCTATAAA
Mhapla.WS247	Mhapla.WS247_Fam_192_46_1	1	46	0.000000	GGGATGTTCATTCCATCTGGGATGAGAGTTTACTCTCATTCCATCT
Mhapla.WS247	Mhapla.WS247_Fam_193_45_1	1	45	0.000000	AATTCTGATTTTTAACTGTATTTTTGTATTAGATTTTTAATTAAA
Mhapla.WS247	Mhapla.WS247_Fam_194_45_1	1	45	0.000000	AATAATTTAATTATGTTTGGATAAAATTCTGAAATGTATTTTCCG
Mhapla.WS247	Mhapla.WS247_Fam_195_45_1	1	45	0.000000	TTATTAATTATTATATTGCATTATTATTCTAATTAACATATTAGT
Mhapla.WS247	Mhapla.WS247_Fam_196_44_1	1	44	0.000000	AAGGTAATCCCCCAAACTCCATGACGTTTGTCGTAGGAGTCCTC
Mhapla.WS247	Mhapla.WS247_Fam_197_42_1	1	42	0.000000	GAAAAAGCCTTCTGGAGTTTCAAGTGAATCGAAAGAACATAC
Mhapla.WS247	Mhapla.WS247_Fam_198_41_1	1	41	0.000000	CGGGAAACGGTAATTTCGGGAAATTACAAATTCCCGAATAT
Mhapla.WS247	Mhapla.WS247_Fam_199_41_1	1	41	0.000000	GCCCCCGACCTAAGTCAGTAAGGCTGCCACCATTGCATCAA
Mhapla.WS247	Mhapla.WS247_Fam_200_41_1	1	41	0.000000	AATTCCGGAAAGTTGTGATTTATTTTACAATTTGCGAATAG
Mhapla.WS247	Mhapla.WS247_Fam_201_41_1	1	41	0.000000	ATAGAGGGTTATTATAGCGAGAGTTTACATTAGTTTCTTAA
Mhapla.WS247	Mhapla.WS247_Fam_202_40_1	1	40	0.000000	CAGAATTTAATATAAACATTAAAATTGAAGAAGTAAAAAT
Mhapla.WS247	Mhapla.WS247_Fam_203_40_1	1	40	0.000000	AAACTTAAGTTTGCGTTTTCTGATTTAGTAAATAATTTTA
Mhapla.WS247	Mhapla.WS247_Fam_204_40_1	1	40	0.000000	AAATTTTGAGTTATAGGGGTTTGAAATTTTTTATAGGCGA
Mhapla.WS247	Mhapla.WS247_Fam_205_40_1	1	40	0.000000	CCGAGACGGCAAAGGTATTGACACATCTACTAACAAGTAT
Mhapla.WS247	Mhapla.WS247_Fam_206_39_1	1	39	0.000000	CCTTCAGCCCATGTCCCCTCCACACTACACTCCATGTTC
Mhapla.WS247	Mhapla.WS247_Fam_207_39_1	1	39	0.000000	AGGGTATTTAATAAATACCCTATTTCTGCTAATAGAAAT
Mhapla.WS247	Mhapla.WS247_Fam_208_39_1	1	39	0.000000	CCAACTACAACCCCGGCTGAAGAGACTACCACACCAGCT
Mhapla.WS247	Mhapla.WS247_Fam_209_39_1	1	39	0.000000	AAAGTTTCAGAATAAGTTGGAAATTGTGTTTTAAAGGAA
Mhapla.WS247	Mhapla.WS247_Fam_210_37_1	1	37	0.000000	TTTTCCGGAAGATTAATATTCATTGTTTTACATTTAA
Mhapla.WS247	Mhapla.WS247_Fam_211_37_1	1	37	0.000000	AAGTAAATCAGACTTCTAGACTGTCAAAATAAACTTA
Mhapla.WS247	Mhapla.WS247_Fam_212_36_1	1	36	0.000000	GCTAGGTAATAATTTTATTAAGTTAGGTATTATTAA
Mhapla.WS247	Mhapla.WS247_Fam_213_36_1	1	36	0.000000	CGTCGTAGCTGGGCTTGTAGCTCGGTTTGTAACCTT
Mhapla.WS247	Mhapla.WS247_Fam_214_35_1	1	35	0.000000	AAAAATCCAATTACTCTAAATTTACAGTAAGTACA
Mhapla.WS247	Mhapla.WS247_Fam_215_35_1	1	35	0.000000	AAAAAAGTCCCTACTTGACTTTATGTCAGGGGCAA
Mhapla.WS247	Mhapla.WS247_Fam_216_35_1	1	35	0.000000	TTATTTAAAAATGTATTTCATTTACAAATTCAAGT
Mhapla.WS247	Mhapla.WS247_Fam_217_34_1	1	34	0.000000	TTTACAAATCATATATTCTGATTTAAATTATAAT
Mhapla.WS247	Mhapla.WS247_Fam_218_33_1	1	33	0.000000	TATAAGCAAGTTTTAATTTAAATAGGGAACGTT
Mhapla.WS247	Mhapla.WS247_Fam_219_33_1	1	33	0.000000	CTGGTCCATCAACACCTGCTCCAACAGGAACTC
Mhapla.WS247	Mhapla.WS247_Fam_220_32_1	1	32	0.000000	TTCCAACTTTCCTGGAATATTTATTTTTACAA
Mhapla.WS247	Mhapla.WS247_Fam_221_32_1	1	32	0.000000	GGGCATTAGTTATTCGAGATGGCAATAAAGGG
Mhapla.WS247	Mhapla.WS247_Fam_222_32_1	1	32	0.000000	GGGTAATTATTTTTTCAACCTCTTGCTGGAGG

Mhapla.WS247	Mhapla.WS247_Fam_223_32_1	1	32	0.000000	AACAATTTTTCTGATTATTTTTTTTAATTTCC
Mhapla.WS247	Mhapla.WS247_Fam_224_31_1	1	31	0.000000	TAATTGTAATTTTGTTTTCCGGATCTTTTAA
Mhapla.WS247	Mhapla.WS247_Fam_225_31_1	1	31	0.000000	AACCGAAAACCGGGGGTCCAAAAATCGGACT
Mhapla.WS247	Mhapla.WS247_Fam_226_31_1	1	31	0.000000	TGATTTGTTCTGGTTGATTCTGGTACAGTTC
Mhapla.WS247	Mhapla.WS247_Fam_227_30_1	1	30	0.000000	TTTCCGGAAATCGTACAAAAATTTTTGTAC
Mhapla.WS247	Mhapla.WS247_Fam_228_30_1	1	30	0.000000	TCCGGAAAACTTAAAAGAAAATTAGATATT
Mhapla.WS247	Mhapla.WS247_Fam_229_30_1	1	30	0.000000	GATGGATTTTGAGTCGTTGGAGCTGCTGTC
Mhapla.WS247	Mhapla.WS247_Fam_230_30_1	1	30	0.000000	TAAACTTAAAATGAAGCTTATTTTCAAGTT
Mhapla.WS247	Mhapla.WS247_Fam_231_30_1	1	30	0.000000	TCCTTTAACTTCTTCTGTAGTTCCCTCTTT
Mhapla.WS247	Mhapla.WS247_Fam_232_30_1	1	30	0.000000	GTTTATTTAAAATCAGAATTTGTAATTCAG
Mhapla.WS247	Mhapla.WS247_Fam_233_30_1	1	30	0.000000	AATTCTGATTTCCGGAATAAGTTTATTTGA
Mhapla.WS247	Mhapla.WS247_Fam_234_30_1	1	30	0.000000	CTAGCACAGCTGGCACATCAACTGAAGCAC
Mhapla.WS247	Mhapla.WS247_Fam_235_30_1	1	30	0.000000	AATAATTTTCCGGAAAGGTAAATAAATGTA
Mhapla.WS247	Mhapla.WS247_Fam_236_30_1	1	30	0.000000	AGTGGACTTAGCAGCCTTGGAAATGGCCTT
Mhapla.WS247	Mhapla.WS247_Fam_237_30_1	1	30	0.000000	GAGGAAGATGAATCTGGGGTCAGTGAAGAC
Mhapla.WS247	Mhapla.WS247_Fam_238_29_1	1	29	0.000000	AAATACATTTCTTTCTGAAAATTTAAATA
Mhapla.WS247	Mhapla.WS247_Fam_239_29_1	1	29	0.000000	AAAAAACAGAATTTTGGGGGAAGAGAGCC
Mhapla.WS247	Mhapla.WS247_Fam_240_29_1	1	29	0.000000	ATATTTTTACAATTCAAGTTTATGTTGTA
Mhapla.WS247	Mhapla.WS247_Fam_241_28_1	1	28	0.000000	TTTATAAGCCTCTATTTAATAAATACTT
Mhapla.WS247	Mhapla.WS247_Fam_242_27_1	1	27	0.000000	ATTTGGTCACTTAAGGGAAATATACTC
Mhapla.WS247	Mhapla.WS247_Fam_243_27_1	1	27	0.000000	CAAAACGCCAATACCCCCAAAAATCGA
Mhapla.WS247	Mhapla.WS247_Fam_244_27_1	1	27	0.000000	AAATAAACTTGGATTCATAATTACAAT
Mhapla.WS247	Mhapla.WS247_Fam_245_27_1	1	27	0.000000	AAATCCAGCATCAGTATATGGCGCCCA
Mhapla.WS247	Mhapla.WS247_Fam_246_27_1	1	27	0.000000	TCATAATTTACTAATGATTAGTACTAA
Mhapla.WS247	Mhapla.WS247_Fam_247_27_1	1	27	0.000000	AAAATGCCAATAAGGCAAAAAACACTA
Mhapla.WS247	Mhapla.WS247_Fam_248_27_1	1	27	0.000000	AGTGCAAAAACCGTGGATTCGTCCCAT
Mhapla.WS247	Mhapla.WS247_Fam_249_26_1	1	26	0.000000	AAAATGGACAAATTGCCATAAGGGTA
Mhapla.WS247	Mhapla.WS247_Fam_250_26_1	1	26	0.000000	AAACAAGTTTATTTATTCAAAAAGAC
Mhapla.WS247	Mhapla.WS247_Fam_251_26_1	1	26	0.000000	TCTACAGTATTGCTATCCCTTTCTTT
Mhapla.WS247	Mhapla.WS247_Fam_252_26_1	1	26	0.000000	ATTTCGCCATAAAGAACAAAATTTAC
Mhapla.WS247	Mhapla.WS247_Fam_253_25_1	1	25	0.000000	TAAAAGAATTGGACAATCCCCTGTA
Mhapla.WS247	Mhapla.WS247_Fam_254_25_1	1	25	0.000000	TTTCCACAAGTTCAACAAGTTCGGT
Mhapla.WS247	Mhapla.WS247_Fam_255_24_1	1	24	0.000000	ATCCAAGTTTAATATATTATATTC
Mhapla.WS247	Mhapla.WS247_Fam_256_24_1	1	24	0.000000	CCAGGGCTCCCAAAGATAAAGCAT
Mhapla.WS247	Mhapla.WS247_Fam_257_24_1	1	24	0.000000	GCGGAGGAGGAGGCGGATGCAGCA
Mhapla.WS247	Mhapla.WS247_Fam_258_23_1	1	23	0.000000	GAGAACCGAGGTCACCATATTTT
Mhapla.WS247	Mhapla.WS247_Fam_259_23_1	1	23	0.000000	AATTCCGGAAAGAATTTAATATA
Mhapla.WS247	Mhapla.WS247_Fam_260_23_1	1	23	0.000000	GGAATATTTACGAAAAGTTTTCC
Mhapla.WS247	Mhapla.WS247_Fam_261_22_1	1	22	0.000000	AAATAAAATTTCAGAGTTCCGA
Mhapla.WS247	Mhapla.WS247_Fam_262_22_1	1	22	0.000000	TTTAAACTTAATAAATTTAAAG
Mhapla.WS247	Mhapla.WS247_Fam_263_22_1	1	22	0.000000	CCAGGTTTTTTGTCGCAAATTG
Mhapla.WS247	Mhapla.WS247_Fam_264_21_1	1	21	0.000000	CAAATTGTAAACCTTAGATTA
Mhapla.WS247	Mhapla.WS247_Fam_265_21_1	1	21	0.000000	ACCAGAAGTGGTGCAAGCCCA
Mhapla.WS247	Mhapla.WS247_Fam_266_21_1	1	21	0.000000	CCTCCGCATCCACCACCGCCG
Mhapla.WS247	Mhapla.WS247_Fam_267_21_1	1	21	0.000000	AATTTGTAAAAAGTTTTCCAT
Mhapla.WS247	Mhapla.WS247_Fam_268_21_1	1	21	0.000000	AAATTGTTGCCTAAATTGTCT
Mhapla.WS247	Mhapla.WS247_Fam_269_21_1	1	21	0.000000	AGACTCAAGTGACCCTAGATC
Mhapla.WS247	Mhapla.WS247_Fam_270_21_1	1	21	0.000000	AATAATATACAGATGATATAC
Mhapla.WS247	Mhapla.WS247_Fam_271_20_1	1	20	0.000000	AATTGTAAAACATGCGGGAA
Mhapla.WS247	Mhapla.WS247_Fam_272_20_1	1	20	0.000000	TACTCTTATGACTTGATATC
Mhapla.WS247	Mhapla.WS247_Fam_273_20_1	1	20	0.000000	TATTTTACTGTAAGAGAAAA
Mhapla.WS247	Mhapla.WS247_Fam_274_19_1	1	19	0.000000	CCATCCCAAATTAGGAGTT
Mhapla.WS247	Mhapla.WS247_Fam_275_19_1	1	19	0.000000	CCGTTCCATTTTAGGGCAA
Mhapla.WS247	Mhapla.WS247_Fam_276_19_1	1	19	0.000000	TTTTATTATTCGTGCGCTC
Mhapla.WS247	Mhapla.WS247_Fam_277_18_1	1	18	0.000000	AATACAAGCCCAATCCCC
Mhapla.WS247	Mhapla.WS247_Fam_278_18_1	1	18	0.000000	GTTGGTGTTCCTTGGTGT
Mhapla.WS247	Mhapla.WS247_Fam_279_18_1	1	18	0.000000	TATCTCACTTAATTTTAT

Mhapla.WS247	Mhapla.WS247_Fam_280_17_1	1	17	0.000000	AAGAGCAAAAAAGGCGG
Mhapla.WS247	Mhapla.WS247_Fam_281_17_1	1	17	0.000000	TTTGAGGGGATAAATAC
Mhapla.WS247	Mhapla.WS247_Fam_282_16_1	1	16	0.000000	TTTCCCGAAATTCCCG
Mhapla.WS247	Mhapla.WS247_Fam_283_16_1	1	16	0.000000	TGTTCCTTCCACACTT
Mhapla.WS247	Mhapla.WS247_Fam_284_16_1	1	16	0.000000	TTGGATGTTTTGTTAT
Mhapla.WS247	Mhapla.WS247_Fam_285_16_1	1	16	0.000000	CTTCAATTTTTCTTTG
Mhapla.WS247	Mhapla.WS247_Fam_286_15_1	1	15	0.000000	CAAAGTGGGCTGCGT
Mhapla.WS247	Mhapla.WS247_Fam_287_15_1	1	15	0.000000	CGCCTAGTTGTCTCG
Mhapla.WS247	Mhapla.WS247_Fam_288_15_1	1	15	0.000000	AAAGCGCAGTGGAAG
Mhapla.WS247	Mhapla.WS247_Fam_289_15_1	1	15	0.000000	CCAACCTACCGCTGC
Mhapla.WS247	Mhapla.WS247_Fam_290_15_1	1	15	0.000000	AATTTTAACTCAAAT
Mhapla.WS247	Mhapla.WS247_Fam_291_15_1	1	15	0.000000	GACTTTTGAGGATCC
Mhapla.WS247	Mhapla.WS247_Fam_292_15_1	1	15	0.000000	TCGACTTTTTTATTA
Mhapla.WS247	Mhapla.WS247_Fam_293_14_1	1	14	0.000000	GCCAACTGAGTGTT
Mhapla.WS247	Mhapla.WS247_Fam_294_14_1	1	14	0.000000	ATTTACAATTTGTA
Mhapla.WS247	Mhapla.WS247_Fam_295_14_1	1	14	0.000000	TATATAGGTAGTAA
Mhapla.WS247	Mhapla.WS247_Fam_296_14_1	1	14	0.000000	ACTAGAGCGCTCCC
Mhapla.WS247	Mhapla.WS247_Fam_297_13_1	1	13	0.000000	TTTACATCGTACT
Mhapla.WS247	Mhapla.WS247_Fam_298_13_1	1	13	0.000000	TAACTCCCTCATA
Mhapla.WS247	Mhapla.WS247_Fam_299_13_1	1	13	0.000000	AATTTTTGGCATT
Mhapla.WS247	Mhapla.WS247_Fam_300_11_1	1	11	0.000000	TTACTTCGAAT
Mhapla.WS247	Mhapla.WS247_Fam_301_11_1	1	11	0.000000	AATCGATAAAA
Mhapla.WS247	Mhapla.WS247_Fam_302_11_1	1	11	0.000000	CAGAATATAAC
Mhapla.WS247	Mhapla.WS247_Fam_303_10_1	1	10	0.000000	TTTTTCGGAA
Mhapla.WS247	Mhapla.WS247_Fam_304_10_1	1	10	0.000000	GCAAATATTT
