# Supplementary material for: High evolutionary turnover of satellite families in Caenorhabditis
Source: BMC Evol Biol. 2015 Oct 5;15:218. doi: 10.1186/s12862-015-0495-x (PMC4595182; doi:10.1186/s12862-015-0495-x)
Supplement: Additional file 3: — Supplementary Tables. (PDF 802 kb) [file 12862_2015_495_MOESM3_ESM.pdf]

“High evolutionary turnover of satellite families in *Caenorhabditis*”

JA Subirana, MM Albà and X Messeguer

Supplementary Figures

Figure S1. Distribution of repeat sequences in each chromosome of *C. elegans.*

Figure S2. Size of satellites, as a function of their position in each chromosome.

Figure S3. Shared satellite families between pairs of *Caenorhabditis* species.

Figure S4. Distribution of the $(ACTACAA)_2$ sequence in chromosome I of *C. elegans* and *C. briggsae*.

Figure S5. Histogram of human satellites.

Figure S1. Distribution of repeat sequences in each chromosome of *C. elegans*. It is clear that all chromosomes have a similar distribution, with the exception of Chromosome X. Satellites longer than 80 bases have been merged into bins of 20 bases.

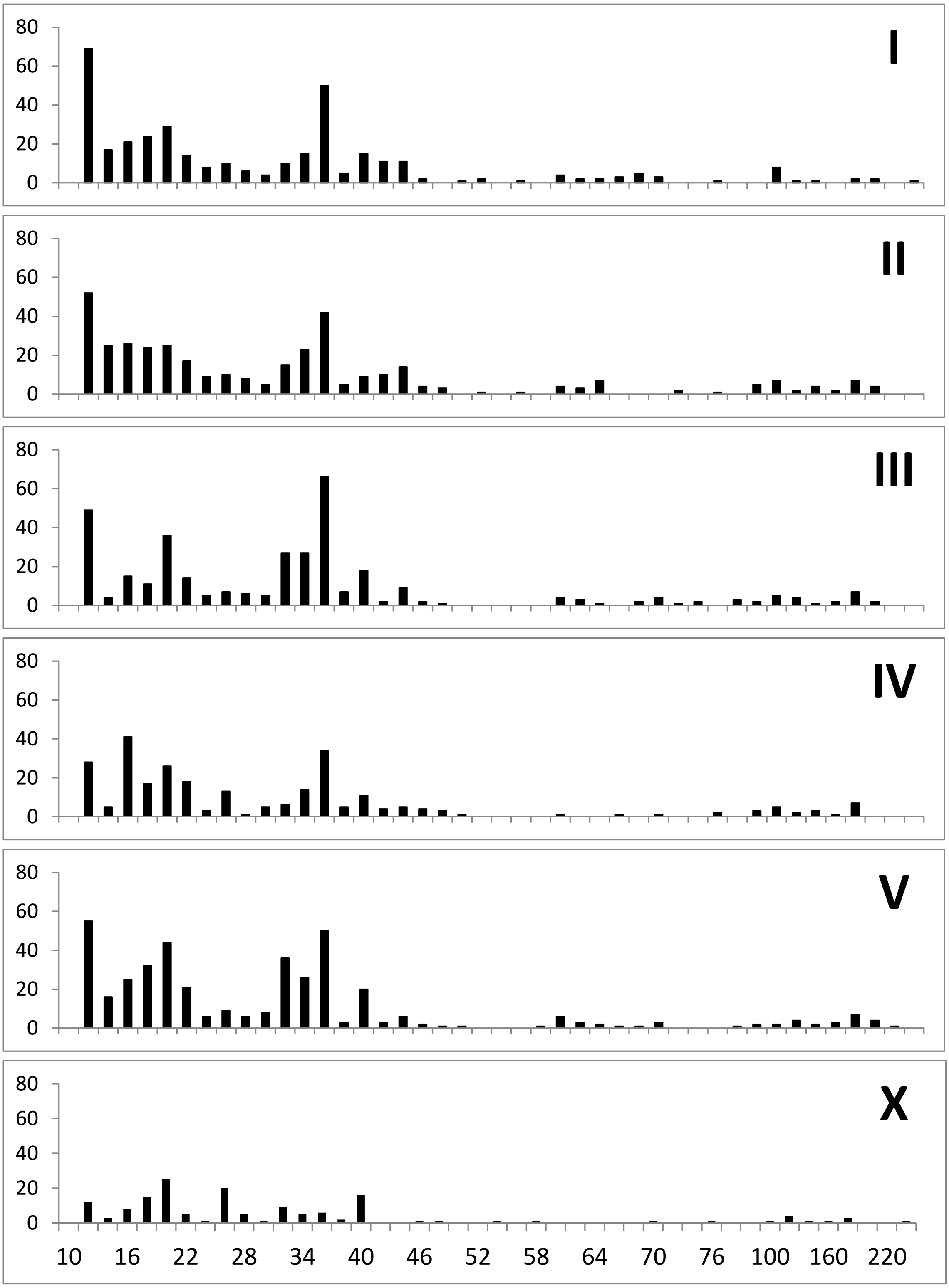

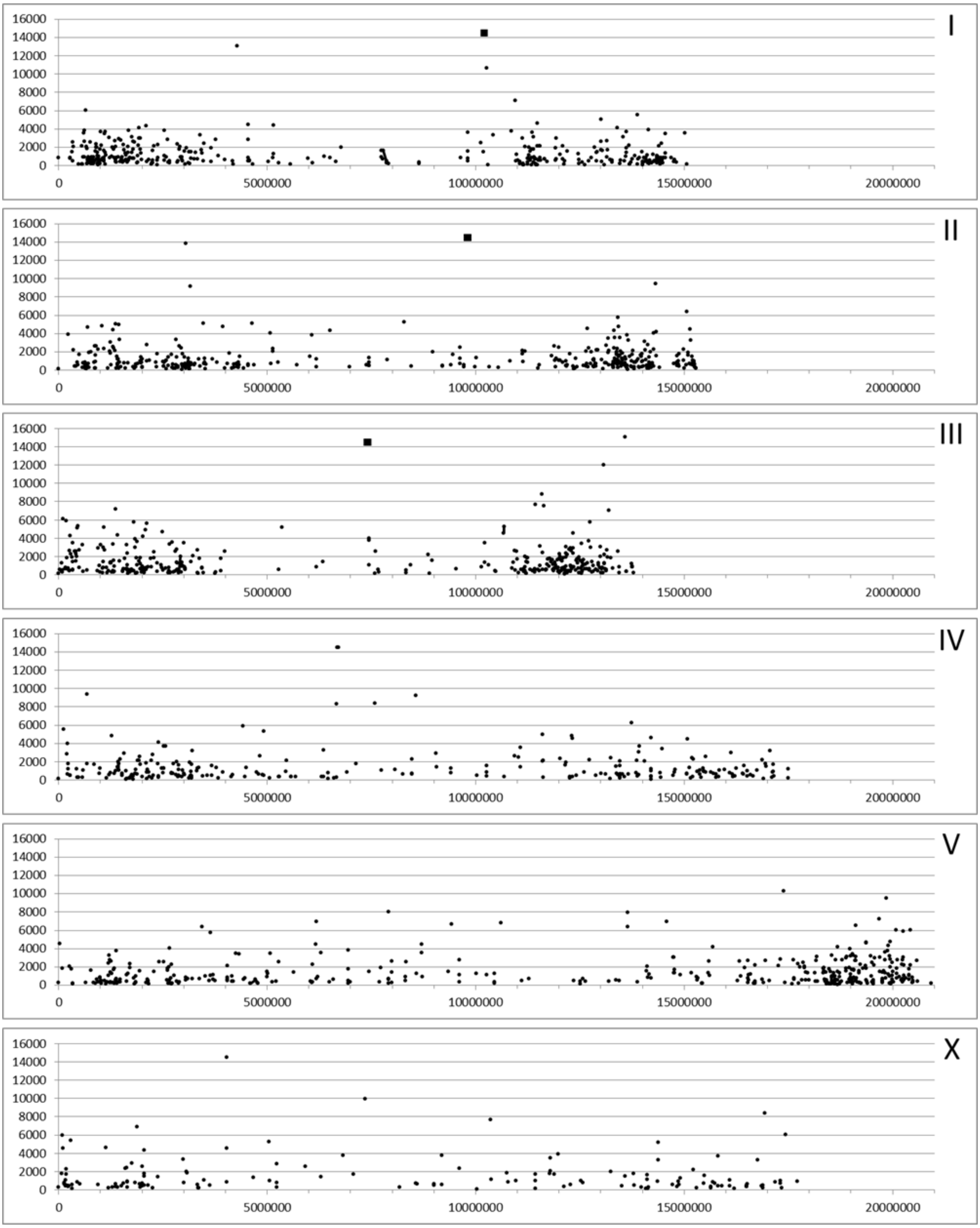

Figure S2. Size of satellites, as a function of their position in each chromosome. A few satellites are indicated by a square: they are longer than what is indicated in the figure. A more detailed presentation for chromosome I is given in Figure 2.

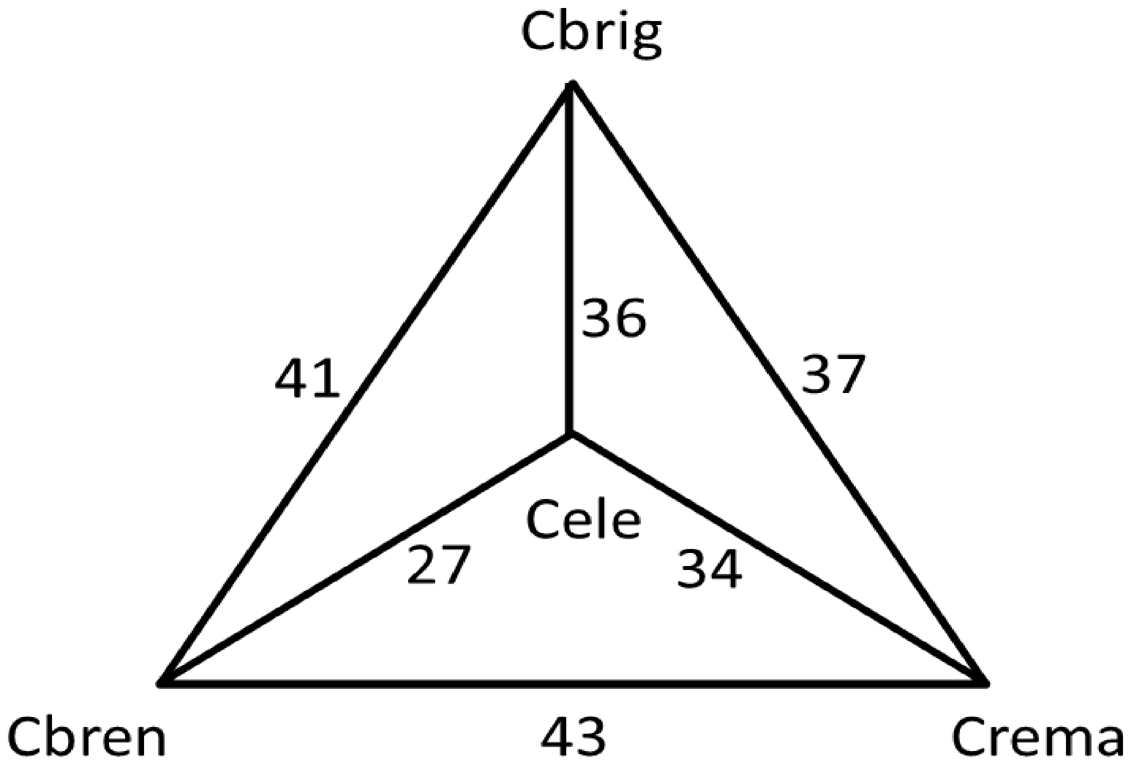

Figure S3. Shared satellite families between pairs of *Caenorhabditis* species.

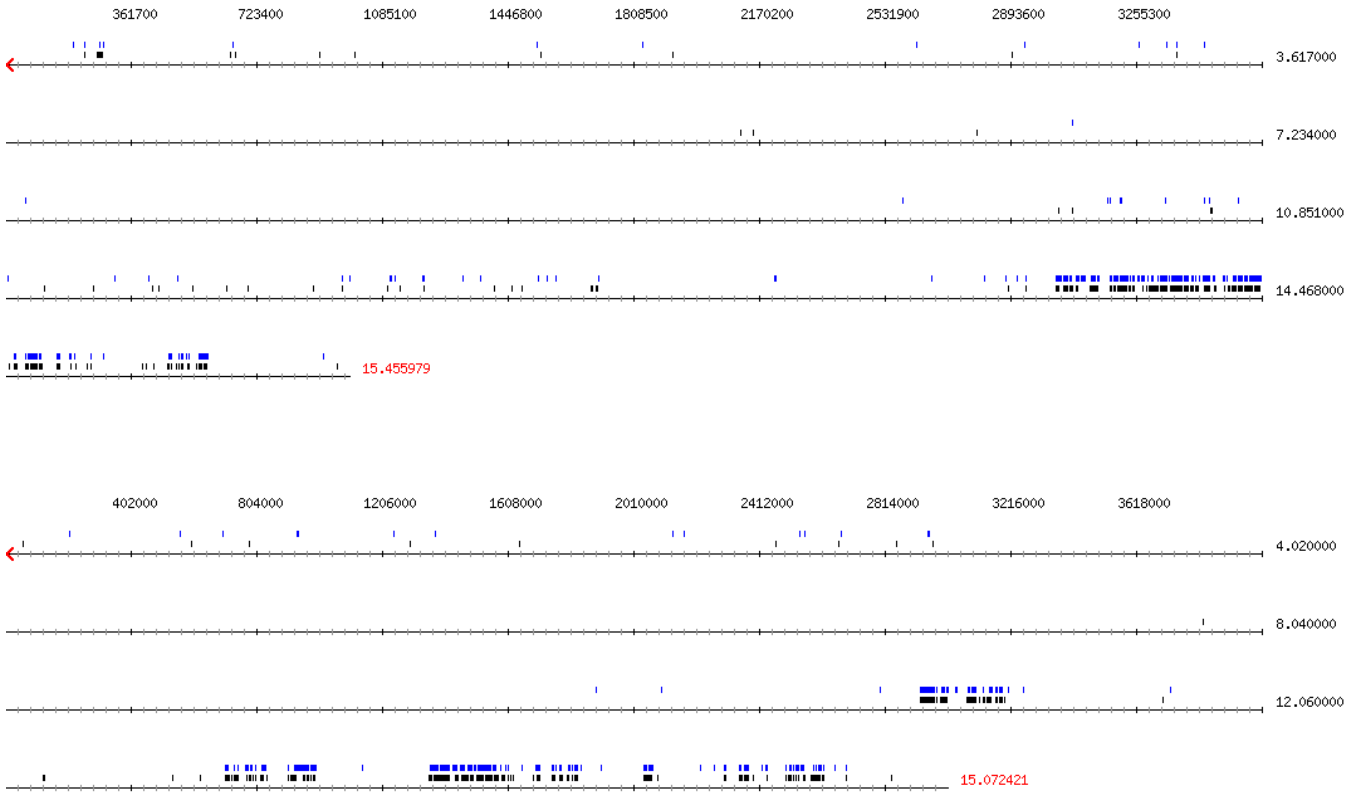

Figure S4. Distribution of the $(ACTACAA)_2$ sequence (black) and its reverse complement (blue) in chromosome I of *C. elegans* (lower frame) and *C. briggsae* (upper frame).

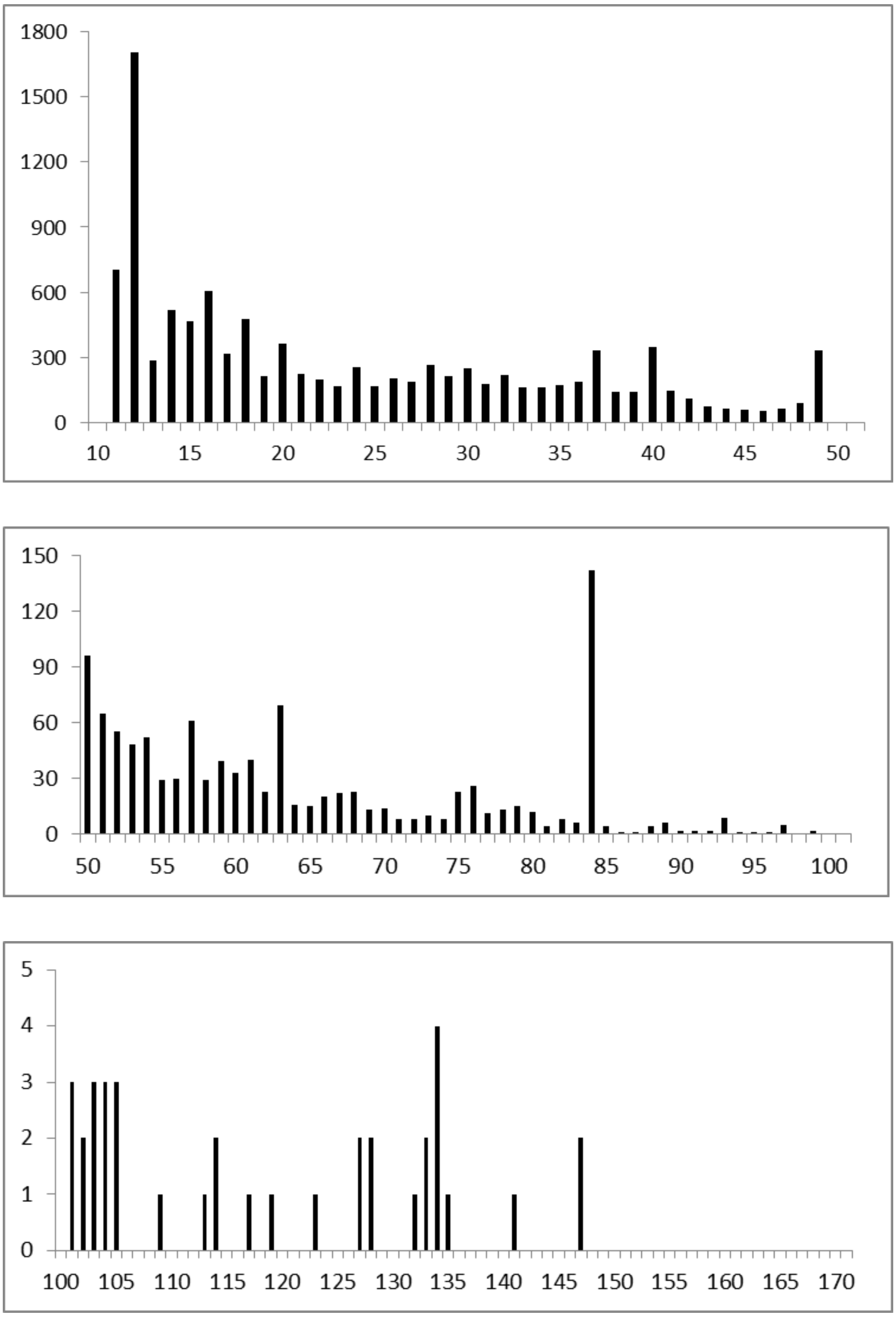

Figure S5. Histograms showing the number of human satellites as a function of the length of their repeats.
