# Supplementary material for: High evolutionary turnover of satellite families in Caenorhabditis
Source: BMC Evol Biol. 2015 Oct 5;15:218. doi: 10.1186/s12862-015-0495-x (PMC4595182; doi:10.1186/s12862-015-0495-x)
Supplement: Additional file 4: — Satellite families in Caenorhabditis and in M. hapla . (PDF 268 kb) [file 12862_2015_495_MOESM4_ESM.pdf]

“High evolutionary turnover of satellite families in *Caenorhabditis*”

JA Subirana, MM Albà and X Messeguer

Supplementary Tables

Table S1. Location of satellites in deletions found in natural isolates of *C. elegans*

Table S2. Satellite family members in the chromosomes of *C. elegans*

Table S3. CENP-A score of satellites with long repeats (>150 bases) in *C. elegans.*

Table S4. Chromosome positions of Cele_Fam_24_19_12: $(AT3)_{3\text{-}4}$AATTAAT

Table S5. Complete sequence of all the Cele_Fam_24_19_12 satellites

Table S6. Alignment of large satellite families in *Caenorhabditis*

Table S7. Satellite families shared by *M. hapla* and *Caenorhabditis*

Table S8. The microsatellites in *Caenorhabditis*

Table S1. Location of satellites in deletions found in natural isolates of *C. elegans*

Maydan et al. [28] have provided a catalogue of several indels in twelve natural isolates of *C. elegans*, which add up to about 3.2 Mb of the genome (excluding duplications). We have determined the position of satellites in all those indels. We would expect 56.6 satellites distributed throughout these regions, since there are 17.7 satellites/Mb in *C. elegans* (Table 2). Satellites were found only in some of them, with a total number of 58 satellites of different sizes and repeats. Therefore we can conclude that satellites are randomly distributed in the deletions found in the different strains studied by Maydan el al. In order to avoid redundancies, we do not show small indels present as a fraction of longer indels.

Chr.	Indel			Satellite			
	start	end	size	start	end	length	Repeat
1	11364959	11370480	5521	11363099	11366734	3635	22
1	13111296	13153623	42327	13149264	13151975	2711	13
1	15016450	15028313	11863	15025303	15028836	3533	21
2	885958	894076	8118	891132	892028	896	59
2	899953	912358	12405	898795	901439	2644	22
2	899953	912358	12405	902801	903323	522	17
2	1535351	1593891	58540	1578533	1579326	793	23
2	1750664	1822745	72081	1796659	1797045	386	34
2	1750664	1822745	72081	1812740	1812972	232	18
2	1750664	1822745	72081	1815685	1815917	232	26
2	1825029	1934453	109424	1892812	1893498	686	26
2	1995557	2022712	27155	2000886	2001208	322	12
2	1995557	2022712	27155	2002111	2002471	360	32
2	1995557	2022712	27155	2008284	2009472	1188	31
2	2063759	2138338	74579	2115413	2116408	995	11
2	2265489	2338778	73289	2329985	2330860	875	40
2	2344994	2353289	8295	2348338	2348687	349	33
2	2521667	2546368	24701	2525627	2526075	448	18
2	2521667	2546368	24701	2526938	2527351	413	12
2	2521667	2546368	24701	2529568	2530452	884	35
2	3196064	3249275	53211	3246628	3247181	553	20
2	3335696	3363341	27645	3361867	3363047	1180	43
2	3672299	3693900	21601	3691106	3692335	1229	31
2	13610426	13617819	7393	13612519	13612876	357	14
2	14982543	14985471	2928	14984063	14985128	1065	20
3	949229	973819	24590	951024	953927	2903	12
3	1295561	1307017	11456	1299749	1303117	3368	25
3	1311955	1316567	4612	1312378	1312777	399	21
3	1311955	1316567	4612	1314691	1315015	324	21
3	1346343	1409647	63304	1362848	1369996	7148	10
3	1893285	1895451	2166	1890358	1894009	3651	19
3	12320776	12322654	1878	12320572	12322233	1661	35
4	2563486	2594976	31490	2572290	2575957	3667	164

4	4124569	4166385	41816	4142135	4142511	376	12
4	17134566	17145165	10599	17138350	17140071	1721	184
4	17134566	17145165	10599	17140409	17141319	910	15
5	1274592	1274909	317	1273381	1274666	1285	35
5	3247858	3433564	185706	3373169	3373914	745	21
5	4783636	4788086	4450	4784583	4784785	202	16
5	15282820	15399718	116898	15295228	15296185	957	35
5	15282820	15399718	116898	15320755	15321325	570	40
5	15282820	15399718	116898	15321461	15322267	806	15
5	17238425	17347568	109143	17265086	17267237	2151	156
5	17238425	17347568	109143	17308688	17311507	2819	11
5	17371630	17489726	118096	17384209	17394484	10275	59
5	17371630	17489726	118096	17412041	17412354	313	19
5	17491246	17588401	97155	17587172	17587321	149	12
5	17801810	17820902	19092	17817696	17818236	540	34
5	18186552	18247813	61261	18190373	18190779	406	11
5	18678249	18684222	5973	18679671	18683869	4198	18
5	18770400	18776232	5832	18774110	18774554	444	11
5	19230781	19237263	6482	19229066	19231343	2277	12
5	19230781	19237263	6482	19232563	19232783	220	12
5	19390251	19469469	79218	19402218	19402465	247	17
5	19480251	19492283	12032	19488470	19489504	1034	17
5	19833649	19846181	12532	19836950	19837768	818	33
5	20435644	20439262	3618	20432075	20438121	6046	180
6	1752189	1778099	25910	1752274	1755174	2900	27

Table S2.
Satellite family members in the chromosomes of *C. elegans*

In this table the families are ordered by increasing length of the repeat.. Only families with more than 9 members are shown. The consensus sequence of each family is given in Additional file 2. Members with one asterisk are shared with other species. All other families are species specific in *C. elegans*.

Family	I	II	III	IV	V	X	Total number	Special features
**4_11**	16	19	13	12	17	5	**82**	Related to *Helitron 2_CE*
**18_11***	3	1	8	1	1	0	**14**	
**2_12***	50	48	40	14	47	4	**203**	Telomeric
**23_12***	5	4	2	1	0	1	**13**	Hexamer dimer
**11_14***	11	12	0	2	8	0	**33**	Heptamer dimer
**5_15**	8	8	8	24	11	6	**65**	Related to *Helitron Y4_CE*
**12_16**	1	9	5	9	5	0	**29**	Octamer dimer
**14_17***	6	2	0	6	4	0	**18**	
**15_17***	3	3	2	3	2	4	**17**	
**25_18**	0	0	0	0	10	1	**11**	
**6_19**	10	2	19	5	13	2	**51**	
**10_19**	5	2	10	5	9	5	**36**	
**16_19***	0	1	0	0	14	0	**15**	
**24_19**	1	3	0	3	0	5	**12**	Mixed with 23-mer
**17_20***	1	4	3	0	5	1	**14**	Found in *Helitrons*
**22_22***	1	5	3	4	0	0	**13**	
**8_25**	5	7	5	5	7	9	**38**	
**21_26**	3	1	1	1	2	5	**13**	
**26_31**	0	0	10	0	0	0	**10**	Pairing centers
**13_32**	0	0	0	0	20	2	**22**	Pairing centers
**1_35**	44	36	72	20	40	1	**213**	Most abundant
**3_34**	14	15	18	26	39	12	**124**	Found in *Helitrons*
**20_37**	2	1	7	2	0	1	**13**	Related to 3_34
**7_40**	9	5	6	7	8	12	**47**	Related to *Helitron Y4_CE*
**9_43**	9	10	9	2	6	0	**36**	Related to 1_35
**19_59**	1	3	4	1	4	0	**13**	Centromere-like
**Total in families**	208	201	245	153	272	76	**1155**	Families with more than 9 members
**Total in genome**	321	354	332	241	396	135	**1779**	

Table S3 . CENP-A score of satellites with long repeats (>150 bases) in *C. elegans.*

Satellites with a repeat of 59 bases and a high CENP-A score are also included in the Table.

Position	Satellite length	Number of repeats	Repeat length	CENP-A score
Chr I				
338563	2587	14	184	1.544
1026802	2177	12	182 (60)	1.073
9816009	3628	14	200 (26)	1.154
10851619	3760	23	163	0.976
13149264	2712	14	224 (13)	1.539
Chr II				
776994	1966	12	163	1.226
1094273	2358	11	161	0.809
2949871	2395	10	155	0.811
3159311	9176	55	162	1.070
6073584	3812	21	181	2.186
9807896	17344	292	59	1.503
11190043	2046	12	202 (90)	0.999
13428517	4784	24	165	1.076
14322264	9454	43	185	1.339
14333333	4159	25	166	1.142
Chr III				
99472	6101	35	178	2.302
448145	5086	21	164	2.201
1017321	3256	55	59	1.114
1362848	7149	44	162 (11)	1.182
2077622	4889	29	168	1.781
11593322	8828	48	184	1.705
11639888	7523	35	163	1.192
13074654	11986	206	59	1.297
13414618	2540	13	198 (64)	1.398
Chr IV				
2572290	3668	16	164	1.156
4822586	2633	16	163 (36)	1.011
6668156	8287	46	182	1.113
6682649	21927	125	172	1.114
6708756	22979	130	174	1.118
12169268	1954	24	59	1.021
15225055	2294	12	168	1.225
15510686	2554	12	168	1.119
17138350	1722	11	184	1.330
Chr V				
265076	2070	12	172	1.127
1201997	2418	13	185	1.326
1704637	1954	13	163	1.090
4244047	3476	18	200	0.972
9428478	6646	31	214 (32)	0.976
14584093	6934	32	162	0.999
16330572	2637	16	164	1.052
17265086	2152	12	156	1.431
17384209	10276	173	59	0.928
18512827	1516	10	151	0.749
20106050	1857	10	185	0.692
20432075	6047	28	180	1.736
Chr X				
2991962	3370	27	167	1.352
5048783	5263	24	226	0.846
10749197	1878	10	214	0.991
16931765	8401	47	151 (57)	1.150

The repeat length of some satellites has been corrected, since they have an internal repeat of the seed motif. In such cases the value given in parenthesis corresponds to the uncorrected value reported by SATFIND.

Table S4. Chromosome positions of Cele_Fam_24_19_12: $(AT3)_{3\text{-}4}$AATTAAT

Chromosome	Length (bases)	Position	Properties
I	165	4281275	Intergenic
I	840	12782330-r	Intergenic
II	842	4308301	Intergenic
II	604	9719108	Intergenic
II	353	9719725-r	Intergenic. Neighbor of previous satellite
IV	139	1639370	Intron
IV	180	2124726	Intron
IV	616	13461609	Intergenic. Contains inserted *Helitron*
V	229	4554501	Intergenic. Contains inverted satellite
X	560	2004045	Intergenic. Placed between two *Helitrons*
X	995	2008253	Intergenic. Contains inserted *Helitron*
X	286	5227453	Intergenic. Contains inverted satellite
X	1224	5230918-r	Intergenic. Neighbor of previous satellite. Contains inserted *Helitron*
X	580	9189061-r	Intron
X	564	12276204	Intergenic. Includes a short inversion

r indicates that it is a reverse complement sequence

Table S5. Complete sequence of all the Cele_Fam_24_19_12 satellites: $(ATTT)_{3-4}$AATTAAT (19-23 bases)

This satellite family is specific for *C. elegans*. Its repeat is completely absent in all other *Caenorhabditis*. The AATTAAT sequence is shown in blue in this Table. Point mutations are shown in red. Related repeated sequences in some satellites are separated by an empty line. At the beginning of the Table four short satellites are shown, which were not detected by SATFIND, due to their small size. We have also recovered a related satellite (Chromosome X-5230918), which had been rejected by SATFIND due to its irregular sequence.

The first step in the appearance of this satellite was probably a partial duplication of the motif with 23 bases, which gave rise to a longer motif with 42 bases (19 + 23). Subsequent duplications originated longer satellites. Inspection of the Table demonstrates that these satellites grew further by unequal crossing-over, as shown by the distribution of point mutations, usually of fragments of about 120-200 bases. This is clearly apparent in Cele.WS201_1:12782330-12783169 and in many other satellites. In some cases point mutations appeared before crossing-over, but in other cases the satellites grew before any apparent point mutation. If we classify the satellites by the number of point mutations and the relative distribution of motifs with either three or four ATTT units, it appears that individual satellites have different ages. Old satellites have acquired more point mutations than younger satellites.

Two clearly transposed sequences in the X chromosome are shown in green. The two satellites involved are separated by about 4 Kb which contain one *Helitron* (Y2_CE4) and a LINE sequence (RTE1). These two elements might have facilitated the local transposition detected. At the end we present a few related sequences which might correspond to degraded satellites.

> 4281275 chromosome I
GGCAAAA
ATTTATTTATTTATTTAATTAAT
ATGTATTTATTTAATTAAT
ATTTATTTATTTATTTAATTAAT
ATTTATTTATTTAATTAAT
ATTTATTTATTTATTTAATTAAT
ATTTATTTATTTAATTAAT
ATTTATTTCTTTAATTAAT
ATTTTCGATGCTCT

> 1639370 chromosome IV
ATTGAGGCCA
ATTTATTTAATTAAT
ATTTATTTATTTAATTAAT
ATGTATTTATTTAATTAATTAAT
ATTTATTTATTTAATTAAT
ATTTATTCATTTAATTAAT
AATTATTTATTTATTTAATTAAT
CAGAACATTTTA

> 2124726 chromosome IV reverse complement
AAT
ATTTATTTATTTAATTAAT
ATTTATTTATTTATTTAATTAAT
ATTTTTTTATTTAATTAAT
ATTTATTTATTTATTTAATTAAT
ATTTTTTTATTTAATTAAT
ATTTATTTATTTAATTAATTAAT
ATTTATTTATTTAATTAATTAAT
ATTTATTTATTTAATTAAT
ATTTATTTA

> 4554501 chromosome V. Contains inverted repeat
TTTACCGATTTAAATAAT
ATTTATTTATTTAATTAATTAAC
ATTTATTTATTTAATTAAT
ATTTAATTATTTATTTAATTAAT
ATAATTTATTTAATTAAT
ATTTATTTATTTAATTAAT
ATTTATTTATTTATTTAATTAAT
ATTTATTTA (Inverted)
AAATAAATAAATAAAT
ATTAATTAAATAAATAAATAAAT
ATTAATTAAATAAATAAAT
ATTAATC
ATTTACTATATA

> Rev.of:Cele.WS201_1:12782330-12783169

ATTTATTTATTTAATTAAT
ATTTATTTGTTTATTTAATTAAT
ATTTATTCATTTATTTAATTAAT
ATTTGTTTATTTATTTAATTAAT
ATTTATTTATTTAATTAAT
ATTTATTTATTTATCTAATTAAT
ATTTATTTATTTAATTAAT
ATTTATTTATTTAATTAAT
ATTTATTTATTTAATTAAT

ATTTATTTATTTATTTAATTAAT
ATTTATTTGTTTATTTAATTAAT
ATTTATTCATTTATTTAATTAAT
ATTTGTTTATTTATTTAATTAAT
ATTTATTTATTTAATTAAT
ATTTATTTATTTATCTAATTAAT
ATTTATTTATTTAATTAAT
ATTTATTTAATTAAT
TTTTATTTATTTAATTAAT

ATTTATTTATTTATTTAATTAAT
ATTTATTTATTTAATTAAT
ATTTATTTATTTATTTAATTAAT
ATTTATTTGTTTATTTAATTAAT
ATTTATTCATTTATTTAATTAAT
TTTTATTTATTTAATTAAT

ATTTATTTATTTATTTAATTAAT
ATTTATTTATTTAATTAAT
ATTTATTTATTTATTTAATTAAT
ATTTATTTGTTTATTTAATTAAT
ATTTATTCATTTATTTAATTAAT
ATTTATTTATTTAATTAAT

ATTTATTTATTTATTTAATTAAT
ATTTATTTATTTAATTAAT
ATTTATTTATTTATTTAATTAAT
ATTTATTTGTTTATTTAATTAAT
ATTTATTCATTTATTTAATTAAT
ATTTATTTATTTAATTAAT
ATTT

> Cele.WS201_2:4308301-4309142

TTT
ATTTAATTATTTAATTAAT
ATTTATTTATTTAATTAAT
ATTTATTTATTTAATTAAT
ATTTATTTATTTAATTAAT
ATTTCTTTATTTAATTAAT
ATTTATTTATTTAATTAAT
ATTTATTTATTTAATTAAT
AATTATTTATTTGATTAAT
ATTTATTTATTTAATTAAT
ATTTATTTATTTAATTAAT
ATTTATTTATTTAATTAAC
ATTTATTTATTTAATTAAT
ATTTATTTATTTAATTAAT
ATTTATTTATTTATTTAATTAAT
ATTTATTTATTTATTTAATTAAT
ATTTATTTATTTAATTAAT
ATTTATTTATTTATTTAAT
ATTTATTTATTTAATTAAT
ATTTATTTATTTAATTAAT
ATTTATTTATTTAATTAAT
ATTTATTTATTTAATTAAT
ATTTATTTATTTATTTAATTAAT
ATTTATTTTTTTAATTAAT
TTTTATTTATTTAATTAAT
ATTTATTTATTTAATTAAT
ATTTATTTATTTATTTATTTAATTAAC
ATTTATTTATTTAATTAAT
ATTTATTTATTTAATTAAT
ATTTATTTATTTATTTAATTAAT
ATTTATTTATTTATTTAATTAAT
ATTTATTTATTTAATTAAT
ATTTATTTATTTATTTAATTAAT
ATTTATTTATTTAATTAAT
ATTTATTTATTTAATTAAC
ATTTATTTATTTAATTAAT
ATTTATTTATTTAATTAAT
ATTTATTTATTTATTTAATTAAT
ATTTATTTATTTATTTAATTAAT
ATTTATTTTTTTAATTAAT
ATTTATTTATTTAATTAAT
ATTTATTTATTTATTTAATTAAT
ATTTATTTATTTAAT

> Cele.WS201_2:9719108-9719711

TTATTTAATTAAT

ATTTATTTATTTAATTAAT
ATTCATTTATTTATTTGATTAAT
ATTTCTTTATTTAATTAAT
ATTTATTTATTTAATTAAT
ATTTATTTATTTAATTAAT
ATTTATTTATTTATTTAATTAAT
ATTTATTTATTTAATTAAT

ATT-ATTTAATTAAT
ATTTATTTATTTATTTGATTAAT
ATTTCTTTATTTAATTAAT
ATTTATTTATTTAATTAAT
ATTTATTTATTTAATTAAT
ATTTATTTATTTATTTAATTAAT
ATTTATTTATGTAAATAAT
ATTTATTTATTTAATTAAT

ATTTATTTAATTAAT
ATTTATTTATTTAATTAAT
ATTTATTTATTTATTTAATTAAT
AATTATTTATGTAAATAAT
ATTTATTTATTTAATTAAT
ATTTATTTAATTATTTAATTAAT
ATTTATTTATTTAATTAAT
ATTTATTTATTTATTTAATTAAT
ATTTATTTATTTAATTAAT
ATTTATTTATGTAAATAAT
ATTTATTTATTTAATTAAT
ATTTATTTATTTATTTAATTAAT
ATTTAT

> Rev.of:Cele.WS201_2:9719725-9720077. Next to previous satellite.

TAAT
ATTTATTTATTTAATTAAT

ATTTATTTATTTAATTAAT
ATTTATTTATTTATTCAATTAAT
ATTTATATATTTAATTAAT
ATTTATTTATTTAATTAAT
ATTTATTTATTTATTTAATTAAA
ATTTATTTATTTAATTAAT
ATTTATTTATTTAATTAAT
-TGTTTTTATTTAATTAAT
ATTTATTTATTTATTTAATTAAT

ATTTATTTATTTAATTAAT
ATTTATTTATTTATTCAATTAAT
ATTTATATATTTAATTAAT
ATTTATTTATTTAATTAAT
ATTTATTTATTTATTTAATTAAT
ATTTATTTATTTAAATAAT
ATTTATTTATTTAATTAAT
ATTTATT

> Cele.WS201_4:13461609-13463683 With an inserted *Helitron*

AT
ATTTATTTATTTAATTAAT
ATTTATTTAATTAATTAAT
ATTTATTTATTTATTTATTTATTTAATTAATTAAT
ATTTATTTATTTATTTATTTATTTATTTATTTAATTAAT
ATTTATTTATTTAATTAAT
ATTTATTTAATTAATTAAT
ATTTATTTATTTATTTAATTAAT
ATTTATTTATTTATTTAATTAAT
ATTTATTTATTTATTTAATTAAT
ATTTATTTAATTGATTAAT
ATTTATTTATTTATTTAATTAAT
ATTTATTTATTTAGTTAAT
ATTTATTTATTTATTTAATTAAT
ATTTATTTAATTAATTAAT
ATTTATTTATTTATTTAATTAAT
ATTTGTTTATTTATTTAATTAAT
ATTTATTTATTTAATTAAT
ATTTATTTATTTATTTATTTAATTAAT
ATTTATTTATTTATTTAATTAAT
ATTTATTTATTTATTTAAT
ATTTATTTATTTATTTAATTAAT
ATTTATTTAATTATTCAAT
ATTTATTTATTTATTTAATTAAT
ATTTATTTATTTATTTAATTAAT
ATTTATTTATTTATTTAATTAAT
ATTTATTTATTTAATTAAT
ATTTATTTATTTATTTAAT
ATTTATTTAT
AACCTTCTGGGAATTTCTGGAAAACTCTAGAATGTTCCAGAACCTTCTGAAAAATTCTGAAAAGCTTTAGAACCTTCTGGAAA
ATTCTAAAAAATTCTGGAATGTTCCAGAACCTTCTGGAAAATTCGAAAAAATTCTGGATTGTTCTAGAACCTTCTGGAAAATT
AAAAAATGTTGCTGCGAGATCTTCGTGGCGAGACCCATCGTGGCGAGACCCACCGTGGTGAGACCCGTCGTGGTGAGACTCAT
CGTGGCGAGACCCATCGTGGTGAGACTCATCGTGGTGAGACCCATCGTGGCGAGACCCATCGTGGTGAGACCCATCGTGGTGA
GACCCATCGTGGTGATACCATCGTGGCGAGACCCATCGTGGTGAGACCCATCGTGGCGAGACCCATCGTGGTGAGACCCATCG
TGGCGAGACCCATCGTGGTGATACCCATCGTGGCGAGACCCATCGTGGTGATACCATCGTGGCGAGACCCATCGTGGTGAGAC
CCATCGTGGCGAGACCCACCGTGGTGAGACCCGTCATGGTGAGACCCATCGTGGTAAGACCCACCGTGGTGAGACCCGTCGTG
GTGAGACCCACCGTGGTGAGACCCACCGTGGTAATACCCAAAATTTTGGCGGGAAATTTAAATTTTCTGAGAAAAATATTTTG
GCGGGAATTTTTAATTTTCTGAGAAAAATATTTTGGCGGGAAATTTAAATTTTCTGTGAAAAATATTTTGGCGGGAAATTTAA
ATTTTCTGAAAAAAAAATTATTTTGGCGGGAAATTTAAATTTTCTGAAAAAAAAATTATTTTGGCGGGAAATTTAAATTTTCT
GAGAAAAATATTTTGGCGGGAAATTTAAATTTCCTGAGAAAAATATTTTGGCGGGAAATTTAAATTTTCTGTGAAAAATATTT
TGGCGGGAAATTTAAATTTTCTGTGAAAAATATTTTGGCGGGAAATTTAAATTTTCTGAGAAAAATATTTTGGCGGGAAATTT
AAATTTTCTGTGAAAAATATTTTGGCGGGAAATTTAAATTTTCTGTGAAAAATATTTTGGCGGGAAATTTAAATTTTCTGTGA
AAAATATTTTGGCGGGAAATTTAAATTTTCTGAGAAAAATATTTTGGAGAGAAATTTAAATTTTCTGTGAAAAATATTTTGGC
GGGAAATTTAAATTTTCTGTGAAAAATATTTTTGCGGGAAATTTAAATTTTCTGAGAAAAATATTTTGGAGAGAAATTTAAAT
TTTCTGTGAAAAATATTTTGGCGGGAAATTTAAATTTTCTGTGAAAAATATTTTTGCGGGAAATTTAAATTTTCTGAAAATCC
CAAAATTCTGGAAATCTAAATTAATTAATTAAT
ATTTATTTATTTATTTAATAAAT
ATTTATTTATTTATTTAATAAAT
ATTTATTTATTTATTTAATTAAT
ATTTATTTATTTATTTAAT
ATTTATTT

> Cele.WS201_6:2004045-2004604

AT
ATTTATTTATTTAATTAAT
ATTTATTTATTTAATTAAT
ATTTATTTATTTAATTTAT
ATTTATTTATTTATTTAATTAAT
ATTTATTTACTTAATTAAT
ATTTATTTATTTAATTAAT
ATTTATTTATTTACCTAATTAAT
ATTTATTTATTTAATTAAT
ATTTATTTATTTATTTAATTAAT
ATTTATTTATTTAATTAAT
ATTTATTTATTTAACTAAT
ATTTATTTATGTATTTAATTAAT
ATTTATTTATTTATTTCATTAAT
ATTTATTTATTCAATTAAT
ATTTATTTATTTAATTAAT
ATTTATTTATTTACTTAATTAAT
ATTCATTTATTTAATTAAT
ATTTATTTATTTAATTAAT
ATTTATTTATTTATTTAATTAAT
ATTTATTTAAGTAATTAAT
ATTTATTTATTTATTTAAT
ATTTATTTATTTATTTAAT
ATTTATTTATTTAATTAAT
ATTTATTTATTTAATTAAT
ATTTATTTATTTATTTAATTAAT
ATTTATTTATTTATTTAATAAAT
ATTTATTTATTTAATTAAT
ATTTATTT

> Cele.WS201_6:2008253-2010825 With an inserted *Helitron*

ATTTATTTAATTAAT
ATTTATTTATTTAATTAAT
ATTTATTTATTTAACTAAT
ATGTATTTATTTATTTAATTAAT
GTTTATTCATTTAATTAAT
ATTTATTTGTTTAATTAAT
ATTTATTTATTTAATTAAT
ATTTATTTATTGATTTAATTAAT
ATTTATTTATTTATTTAATTAAT
ATTTATTTATTTATTTGATTAAT
ATTTATTTATTTAATTAAT
ATTTATTTATTTATTTAATTAAT
ATTTATTTATTTAATTAATTAAT
ATTTATTTAATTAAT
ATTTATTTATTTAATTAAT
ATATATTTATTTAAATAAT
ATTTATTTATTTAATTAAA
TGTTTTTTTATTTATTTAATTAAT
ATTTATTTATTTAACTAAT
ATTTATTTATGTATTTAAATAAA
ATTTATT-ATTTATTTAATTAAT
ATTTTTTCATTTAATTAAT
ATTTTTTTATTTAATTAAT
ATTTATTTATTTAATTAAT
ATTTATTTATTTAATTAAT
ATGTATTTATTTATTTAATTAGT
ATTTATTCATTTAATTAAT
ATTTATTTATTTAATTAAT
ATTTATTTATTTATTTAATTAAT
ATTTATTTATTCAATTAAT
ATTTATTTATTTAATTAAT
ATTTATTTATTTACTTAATTAAT
ATTTATTTATTTAATTAAT
ATTTATTTATTTAATTAAT
ATTTATTTATTTATTTAATTAAT
ATTTATTTAAGTAATTAAT
ATTTATTTATTTATTTAAT
ATTTATTTATTTAATTAAT
ATTTATTTAAGTAATTAAT
ATTTATTTATTTATTTAAT
ATTTATTTATTTATTTAAT
ATTTATTTATTTAATTAAT
ATTTTTTTATTTAATTAAT
ATTTATTTATTTAATTAAT
ATTTTTTTATTTATTTAATAAAT
ATTTATTTATTTATTTAATAAAT
ATTTATTTATTTAATTAAT
TTTTATTTATTTAATTAAT
TTTTATTTATTTAATTAAT
ATTTACCAGCCGTGGACCGCACCTTCGGCGCGGCCCCCGACTTCTGGGGCTGAAAATTAGTTTTTTATGAAACTACCGTAACC
CTACAGTATTCCTACCGTACCACTATTGTACCACTACAGTACCCCTACTATATCCCTACACTAACCCTAACTCACTATCCCTC
CAGAAGCCAAAACTTCACAGACTACAAAGACTACATAGACTACAAACTATGGACACACAGAATAAGCGCTTTATATATAGTAA
ATGATG
ATTTATTTAATTAAT
ATTTATTTATTTATTTAATTAAT
ATTTATTTATT

> Cele.WS201_6:5227453-5227738 Contains short inverted sequence

ATTTATTTAATTAAT
ATTTATTTATTTGAATAAT
ATTTATTTATTTATTTAATTAAT
ATTTATTTATTTAATTAAT
TAATTTTTTTTTTGTTTAAT
ATTTATTTATTTATTTAATTATT
ATTTATTTATTTAATTAAT
ATTTATTTATTTAATTATT
ATTTATTTATTTAATTATT
ATTTATTTATTTGATTATT
ATTTATTTATTTAATTAAT
ATTTATTTCTTCATTTCATTAAT
ATTTATTTAATTCATTATT
ATTTACTTCTTTAAATAAT
ATTTATTTAAAATTAAATAAATAAATAAATATTAATTAAAT
AATTATTTAATTAAT
ATTTATTTTTCTAATTAAT
ATTTATTTATTTAATT

>5230918 chromosome X reverse complement. With an inserted *Helitron* sequence

TATTTAATTATT
ATTTATTTATTTAATTAAT
ATTTATTTATTTAATTAAT
ATTTATTTATTTATTTGATTAAT
ATTTATTTATTTAATTAATTAAT
ATTTATTTATTTATTTAAT
ATTTATTTATTTAAATATTTAAT
ATTTATTTATTTGTTTAAT
ATTTATTTATTAATTTAATTAAT
ATTTATTTATTTAATTAAT
ATTTATTTATTTAATTATT
ATTTATTTATTTAATTAAT
ACTTATTTTTTTAATTAAT
ATTTATTTATTTAATTATT
ATTTATTTATTTAATTAAT
ATTTATTTATTTAAATAAT
ATTTATTTATTTAATTAAT
ATTTATTTATTTATTTAATTAAT
ATTTATTTATTTATTTAATTAAT
ATTT-TTTATTTAATTAAT
ATTTATTTATTTAATTATT
ATTTATTTATTTAATTATT
ATTTATTTATTTAATTAAT
ATTTATTTATTTAATTAAT
ATTTATTTATTTAATTATT
AATTGTTTATTTAATTAAT
ATTTATTTAT
CATTACCTATATATAAAAAAATAGTGTCCGTTTGTTA
AGAAGTGACGTCATAGCTTACACAGGCAAGGCGTTTT
CGGCTGTTGGAGGGATATTAGTTTGGGGTATTTAATTATT
ATTTATTTATTTAATTAAT
ATTTATTTATTTAATTAAT
ATTTATTTATTTATTTGATTAAT
ATTTATTTATTTAATTAATTAAT
ATTTATTTATTTATTTAAT
ATTTATTTATTTAAATATTTAAT
ATTTATTTATTTGTTTAAT
ATTTATTTATTAATTTAATTAAT
ATTTATTTATTTAATTAAT
ATTTATTTATTTAATTATT
ATTTATTTATTTAATTAAT
ACTTATTTTTTTAATTAAT
ATTTATTTATTTAATTAAT
ATTTATTTATTTAATTGAT
ATTTATTTATTTAATTTAT
ATTGATTTATTTAATTAAT
ATTTATTTATTTATTTAATTAAT
ATTTATTTATTTATTTAATTAAT
ATTTATTTATTTATTTAATTAAT
ATTT-TTTATTTAATTAAT
ATTTATTTATTTAATTATT
ATTTATTTATTTAATTATT
ATTTATTTATTTAATTAAT
ATTT-TTTATTTAATTAAT
ATTTATTTATTTAATTATT
ATTTATTTATTTAATTAAT
ATTTATTTATTTAATTAAT
ATTTATTTATTTAATTATT
AATTGTTTATTTAATTAAT
ATTTATTTAT

> Rev.of:Cele.WS201_6:9189061-9189640

TTAATTAAT
ATTTATTT-TTTAATTAAT
ATTTATTTATTTAATTAATTAAT
ATTTATTTATT-AATTAAT
ATTTATTTATTTAATTAAT
ATTTATTTTTTTAATTAAT
ATTTATTTAATTAAT
ATTTATTTGTTTATTTAATTAAT
ATCTATTTATTTAATTAAT
ATTTATTTATTTAATTAAT
ATTTATTTATTTAATTAAT
ATTTATTTATTTAAT
ATTTATTTATTTAATTAATTAAT
ATTTATTTATTTAATTAAT
ATTTATTTATTTATTTAATTAAT
ATTTATTTATTTATTTAATTAAT
ATTTATTTATTTAAT
ATTTATTTATTTAATTAAT
ATTTATTTATTTAATTAAT
ACTTATTTATTTAATTAAT
ATTTATTTGTTCATTTAATTAAT
ATTTATTTATTTAATTAAT
ATTTATTTATTTAATTAAT
ATTCATTTATTTAATTAAT
ATTTATTTATTTAATTAAT
ATTTATTTATTTATTTAATTAAT
ATTTATTTATTTATTTAATTAAT
ATTTATTTATTTAATTAAT
ATTTATTTATTTAATTATT
ATTTATTTATTTAATTAAT
A

> Cele.WS201_6:12276204-12276767 Contains short inverted sequence

TTTAATTAAT
ATTTATTTATTTAATTAAT
ATTTATTTATTTAATTAAT
ATTTATTCATTTAATTAATTAAT
ATTTATTTATTTAATTAAT
ATTTATTTATTTAATTAAT
ATTTATTTATTTAATTAAT
ATTTATCTATTTATTTAATTAAT
ATTTATTTATTTAATTAAT
ATTTATTTATTCATTTAATTAAT
ATTTATTTATTTAATTAAT
ATTTATTTATTTAATTAAT
ATTTATTTATTTATTTAATTAAT
ATATATTTATTTATTTAATTAAT
ATATATTTATTTAATTAAT
ATTTATTTATTTATTTAAT
ACTTATCTATTTAATTAGT
ATTTATTTTTTTAATTAAT
ATTTATTTATTTAATTAAT
ATTTATTTATTCAATTGAT
ATTTATTTTTTCATTTAAT
ATTTATTTATTCAATTAAT
ATTTATTTATTTAATTAAT
ATTTATTTTTTTATTTAAT
AAATAAATATTAATTAAATA
AATTATTTATTTAATTAAT
ATTTATGTATTTAATTAAT
ATGAATTTATTTAATTAAT
ATTTATTTATTTAATTAAT

Sequences related to Cele_Fam_24_19_12

>ce6_dna range=chrII:11467601-11467800 strand=-
CAACGCGTGAGCTTCCAGATACTCGATGAGCTGGTCAACGATTGGAGGGA
TGTTTCCACCACAGTGAGACAGGATGCTGAAA
ATTTGTTTATCTTAATATTAATTA
ATTTATTTATTTAATTAAT
TTAGTTTATAAAAAT
CACGTTTGAATGAC**ATTT**CTGGGAAAACGTGGGAA**ATTT**ATTGGAAAAAAAAAATTCTAA

>ce6_dna range=chrIV:13456651-13456900 strand=-
CTCATATTGGGTCATTACTTTCCTT**ATTT**ACTGTACCTTT
ATTTATTTATTTATT**ATTT**GATTAAT
ATTTATTTATTTATTTAAT
ATTTATTTTATTA**ATTT**
ATTTTTATTA**ATTTATTTAATTAAT**
ATTTATTTATTTAATTAAT
ATTTATTTATTTATTTAATTAAT
ATTTATTTATTC**ATTTAATTAAT**
AATTAAATAATTAAATAAAAC**ATTT**CTGTAACTACGTGAAGCTTCTTGTACCTACTGG

>ce6_dna range=chrIV:13884851-13885050 strand=+
TGACGTCACAGACTACAAAGACTACATAGACT**ATTT**TTAAATTGAT
ATTTATTTATTTAATTAAT
T**AATTAAT**
ATTTT**TTTATTTATTTAATTAAT**
ATTTATTTATTTATTTAAATAAT
ATTTATTTATTTA
TC**ATTT**ACTATATATAAA
GCGCGTGTCCTTCTGTCCCTATGTAGTTTGATCTTTGATCAGAGCAACGA

>ce6_dna range=chrIV:13994001-13994200
TATTTTCACAACTTACTTACTAA**AATATTT**CAAGTTCAGTTTCAACGCGG
AACCAAGATTTTCCAAACTCTTTTTAGCTAGAAAAAGCTTGTACATTTTC
AAAGTATTGATTAGAGAGT
TGATATTTTTAA**ATTTATTTAATTA--ATTTATTTATTTAAT**GGGTCTCTTT
TAATCTTACGACTTTCAAAAAAAAAAAAACG

>ce6_dna range=chrV:4602651-4602850 strand=-
AAGAAGTTATG**ATTT**TTGACCCGGAACTT**ATTT**GGAGACCTAATAATTCAAAAACAAT
ATTTATTTTT**ATTT**TTT
TTTT**ATTTATTTAATTAAT**TA
ATTTTTTTT**ATTT**AATTGATG**AATTAAT**TCATTA**ATTT**TTTGGAAAATACTTA
ATTTGGAAAATACTCATTAGAACACAAG**ATTT**GTTACAAAATGTCTAGAGG

Table S6. Alignment of large satellite families in *Caenorhabditis*

```
>All.fam_Fam_1_20_5  Nr. of seq. 5 Alignment length(with gaps) = 21 Alignment score = 0.585714
Cbren.WS247_Fam_47_18_7                    AAATTC-CG-AAAATTCT-GA
Cele.WS201_Fam_321_19_1                    AAACTCGAG-AAAATTCT-GA
Cbren.WS247_Fam_33_20_9                    ANCTTCCAGAAAC-TTCTAGA
Rev.of:Cbrig.WS247_Fam_11_20_22            AAATTCCAG-AACATTCTAGA
Cele.WS201_Fam_319_20_1                    AAATTCCAG-AAGGTTCTAGT
                                           *   **  * **  **** *
Consensus:
                                           AaatTCcaGAAnaTTCTaGa

>All.fam_Fam_2_12_5  Nr. of seq. 5 Alignment length(with gaps) = 15 Alignment score = 0.737778
Cbren.WS247_Fam_8_12_37                    GC--TT-AGGCTTAG
Rev.of:Cbrig.WS247_Fam_108_12_2            GC--TT-AGGCTTAG
Rev.of:Cele.WS201_Fam_2_12_203             GC--TT-AGGCTTAG
Rev.of:Crema.WS247_Fam_22_12_15            GC--TT-AGGCTTAG
Cbren.WS247_Fam_389_15_1                   GCTTTTCAGGCTTAG
                                           **  ** ********
Consensus:
                                           GCTTAGGCTTAG (Telomere sequence)

>All.fam_Fam_5_24_4  Nr. of seq. 4 Alignment length(with gaps) = 24 Alignment score = 0.695602
Crema.WS247_Fam_73_21_4                    T-TGGGTCATGACCTAA-TTAAT-
Rev.of:Cbrig.WS247_Fam_3_22_70             TCTAGGTCATGACCTAA--TTTTT
Rev.of:Cbren.WS247_Fam_10_24_31            TCTAGGTCATGACCTAATTTTTTT
Cele.WS201_Fam_280_24_1                    AGTAGGTCATGACCTAATTTTTTA
                                             * *************  *  *
Consensus:
                                           tcTaGGTCATGACCTAAttTttTt

>All.fam_Fam_7_14_4  Nr. of seq. 4 Alignment length(with gaps) = 17 Alignment score = 0.643791
Cbrig.WS247_Fam_19_14_12                   TGGA-ATCT-GGA-ATC
Rev.of:Crema.WS247_Fam_13_14_26            CGGA-ATCC-GGA-ATC
Cbrig.WS247_Fam_4_15_56                    CGGA-ATCCNGGA-ATC
Cbren.WS247_Fam_15_16_22                   CGGACATCT-GGACATC
                                            *** ***  *** ***
Consensus:
                                           cGGAATCcGGAATC

>All.fam_Fam_8_14_4  Nr. of seq. 4 Alignment length(with gaps) = 14 Alignment score = 0.857143
Cbren.WS247_Fam_24_12_14                   AAG-CGGAAG-CGG
Cbren.WS247_Fam_21_14_15                   AAGTCGGAAGTCGG
Rev.of:Cbrig.WS247_Fam_32_14_5             AAGTCGGAAGTCGG
Rev.of:Crema.WS247_Fam_1_14_208            AAGTCGGAAGTCGG
                                           *** ****** ***
Consensus:
                                           AAGtCGGAAGtCGG

>All.fam_Fam_9_12_4  Nr. of seq. 4 Alignment length(with gaps) = 14 Alignment score = 0.750000
Cbren.WS247_Fam_400_12_1                   GCTCCGGCTCCG--
Rev.of:Cele.WS201_Fam_356_12_1             GCTCCAGCTCCA--
Rev.of:Crema.WS247_Fam_61_12_5             GCTCCAGCTCCA--
Cbrig.WS247_Fam_20_14_12                   GCTCCAGCTCCAGA
                                           ***** *****
Consensus:
                                           GCTCCaGCTCCa
```

```
>All.fam_Fam_10_14_4  Nr. of seq. 4 Alignment length(with gaps) = 14 Alignment score =
0.777778
Cbren.WS247_Fam_84_14_4                    TGTAGTTTGTAGTT
Rev.of:Cele.WS201_Fam_11_14_33             TGTAGTTTGTAGTT
Rev.of:Cbrig.WS247_Fam_266_14_1            CGTAGTATGTAGTT
Crema.WS247_Fam_146_14_2                   TGTAGTCTGTAATC
                                            ***** **** *
Consensus:
                                           tGTAGTtTGTAgTt

>All.fam_Fam_11_13_4  Nr. of seq. 4 Alignment length(with gaps) = 13 Alignment score =
0.602564
Cbren.WS247_Fam_190_10_2                   TTTTT-GAAAT--
Cele.WS201_Fam_40_12_7                     TTTTT-GAAATTT
Rev.of:Crema.WS247_Fam_43_13_7             TTTTTTCAAATTT
Cele.WS201_Fam_18_11_14                    TTTTTGCACAT--
                                           *****  * **
Consensus:
                                           TTTTTncAaATtt

>All.fam_Fam_12_11_4  Nr. of seq. 4 Alignment length(with gaps) = 12 Alignment score =
0.678241
Cbren.WS247_Fam_68_11_5                    -TCGATAAATTA
Rev.of:Cbrig.WS247_Fam_34_11_5             -CCGGTAAATTA
Crema.WS247_Fam_27_11_13                   -CCGGTAAATTA
Rev.of:Cele.WS201_Fam_359_11_1             TCCG-TTAATTA
                                             ** * *****
Consensus:
                                           cCGgTaAATTA

>All.fam_Fam_13_10_4  Nr. of seq. 4 Alignment length(with gaps) = 10 Alignment score =
0.822222
Cbren.WS247_Fam_1_10_97                    TAGAAGTTTC
Rev.of:Crema.WS247_Fam_18_10_16            TAGAAGTTTC
Rev.of:Cbrig.WS247_Fam_12_10_19            TGGAATTTTC
Rev.of:Cele.WS201_Fam_64_10_4              TGGAATTTTC
                                           * *** ****
Consensus:
                                           TaGAAgTTTC

>All.fam_Fam_23_26_3  Nr. of seq. 3 Alignment length(with gaps) = 28 Alignment score =
0.626984
Cbrig.WS247_Fam_59_24_3                    AATTCCCGCCAAAATATTT----TTTTG
Cbren.WS247_Fam_3_28_64                    AATTCCCGCCAAANTTTTTGAATTTTTG
Crema.WS247_Fam_123_26_2                   AATTCCCGCCAAAACGGAA-AA-TTTTG
                                           *************          *****
Consensus:
                                           AATTCCCGCCAAAatntttaaTTTTG

>All.fam_Fam_28_22_3  Nr. of seq. 3 Alignment length(with gaps) = 22 Alignment score =
0.621212
Crema.WS247_Fam_302_20_1                   GTCGATTTACGG-AGCCTCGG-
Crema.WS247_Fam_48_21_6                    GTNGATTTACGGAAGCTT-TGT
Rev.of:Cele.WS201_Fam_22_22_13             GTAGATTTACGGGAGTTTCTNT
                                           ** ********* **  *
Consensus:
                                           GTnGATTTACGGnAGctTctgt

>All.fam_Fam_31_19_3  Nr. of seq. 3 Alignment length(with gaps) = 21 Alignment score =
0.671958
Cbrig.WS247_Fam_68_19_3                    -TAGAGGTCCCAAAAAAAAT-
Crema.WS247_Fam_9_20_29                    TTAGACGTCCCAAAAAACAN-
Rev.of:Cbren.WS247_Fam_5_20_44             -TCGAGGACCCAAAAAAAAAA
                                            * ** * ********* *
Consensus:
                                           TaGAgGtCCCAAAAAAaAn
```

```
>All.fam_Fam_33_20_3  Nr. of seq. 3 Alignment length(with gaps) = 21 Alignment score =
0.650794
Cele.WS201_Fam_17_20_14                    TTTGAATTTTTNAGTCAAAA-
Rev.of:Crema.WS247_Fam_132_21_2            TTTGATATTTTCAGTCTAAAT
Crema.WS247_Fam_92_20_3                    ATTGACTTTTTGANTCGAAA-
                                            ****  **** * ** ***
Consensus:
                                           tTTGAntTTTTnAgTCnAAA

>All.fam_Fam_34_17_3  Nr. of seq. 3 Alignment length(with gaps) = 18 Alignment score =
0.660494
Cbrig.WS247_Fam_71_17_3                    T-CTCTTTTACTACCAAG
Rev.of:Cele.WS201_Fam_14_17_18             T-CTCTTTTACTACGGNN
Crema.WS247_Fam_140_18_2                   TCCTCATTTACTACCGAT
                                           * *** ********
Consensus:
                                           TCTCtTTTACTACcgan

>All.fam_Fam_35_17_3  Nr. of seq. 3 Alignment length(with gaps) = 17 Alignment score =
0.790850
Cbren.WS247_Fam_35_17_9                    CTCATTGACTACATTTA
Cele.WS201_Fam_15_17_17                    CTCATTGACTACCTTTG
Crema.WS247_Fam_329_17_1                   CTCTTTGACTACACTTG
                                           *** ********  **
Consensus:
                                           CTCaTTGACTACatTTg

>All.fam_Fam_36_16_3  Nr. of seq. 3 Alignment length(with gaps) = 16 Alignment score =
0.694444
Cbren.WS247_Fam_17_16_20                   TCTGTATGTCTGTATG
Rev.of:Crema.WS247_Fam_143_16_2            TCTGTCAGTCTGTGTG
Cbren.WS247_Fam_37_16_9                    TCTGTCTATCTGTCTA
                                           *****   ***** *
Consensus:
                                           TCTGTctgTCTGTnTg

>All.fam_Fam_37_16_3  Nr. of seq. 3 Alignment length(with gaps) = 16 Alignment score =
0.666667
Cbren.WS247_Fam_30_16_11                   CTCTCAGGCTCTCAGG
Cele.WS201_Fam_342_16_1                    CTCCTAGGCTCCTAGG
Rev.of:Crema.WS247_Fam_145_16_2            CTTTTAGGCTTTTAGG
                                           **   *****   ***
Consensus:
                                           CTcttAGGCTcttAGG

>All.fam_Fam_41_15_3  Nr. of seq. 3 Alignment length(with gaps) = 15 Alignment score =
0.940741
Cbren.WS247_Fam_16_15_20                   GAAACCGGACATTTC
Crema.WS247_Fam_33_15_9                    GAAACCGGACATTTC
Cbrig.WS247_Fam_14_15_16                   GAAACCAGACATTTC
                                           ****** ********
Consensus:
                                           GAAACCgGACATTTC

>All.fam_Fam_42_14_3  Nr. of seq. 3 Alignment length(with gaps) = 14 Alignment score =
0.682540
Cbrig.WS247_Fam_269_12_1                   G-CCCTAG-CCCTA
Rev.of:Crema.WS247_Fam_26_14_13            GACCCTAGACCCTA
Rev.of:Cele.WS201_Fam_349_14_1             GTCCCTAGTCCTTA
                                           * ****** ** **
Consensus:
                                           GnCCCTAGnCCcTA

>All.fam_Fam_74_23_2  Nr. of seq. 2 Alignment length(with gaps) = 23 Alignment score =
0.659420
Crema.WS247_Fam_15_21_19                   CTGAGGCTAACTGAGA-TTTTG-
Cele.WS201_Fam_123_22_2                    CTGAGGCTCTCT-AGATTTTTGG
```

```
                                            ********  ** *** *****
Consensus:
                                            CTGAGGCTaaCTgAGAtTTTTGg

>All.fam_Fam_85_22_2  Nr. of seq. 2 Alignment length(with gaps) = 22 Alignment score =
0.681818
Cbrig.WS247_Fam_5_21_47                     CATTTTTCATGATTTT-TGGNC
Cbren.WS247_Fam_55_22_6                     CATTTTTCAAGGTTTTCTAGGC
                                            ********* * **** * * *
Consensus:
                                            CATTTTTCAaGaTTTTcTaGgC

>All.fam_Fam_91_21_2  Nr. of seq. 2 Alignment length(with gaps) = 21 Alignment score =
0.682540
Cbrig.WS247_Fam_8_20_31                     AGCCTCAGTAAAAATTCC-TA
Rev.of:Cele.WS201_Fam_320_20_1              AG-CTCAGGAAAAATTCCAGA
                                            ** ***** *********  *
Consensus:
                                            AGcCTCAGgAAAAATTCCagA

>All.fam_Fam_108_19_2  Nr. of seq. 2 Alignment length(with gaps) = 19 Alignment score =
0.789474
Cbren.WS247_Fam_358_19_1                    GAAATGCGCCAAGACACAA
Cele.WS201_Fam_16_19_15                     GAATTGCGCCAAGGCACTA
                                            *** ********* *** *
Consensus:
                                            GAAaTGCGCCAAGaCACaA

>All.fam_Fam_126_16_2  Nr. of seq. 2 Alignment length(with gaps) = 16 Alignment score =
0.718750
Cbrig.WS247_Fam_6_14_46                     GGTCCT-GGGTCCTG-
Rev.of:Cbren.WS247_Fam_179_16_2             GGTTCTGGGGTCCTGG
                                            *** ** ********
Consensus:
                                            GGTcCTgGGGTCCTGg

>All.fam_Fam_133_16_2  Nr. of seq. 2 Alignment length(with gaps) = 16 Alignment score =
0.833333
Cbrig.WS247_Fam_21_16_12                    GATCCAGGGATCCAGG
Cele.WS201_Fam_87_16_3                      GTTCCAGGGTTCCAGG
                                            * ******* ******
Consensus:
                                            GaTCCAGGGaTCCAGG

>All.fam_Fam_139_14_2  Nr. of seq. 2 Alignment length(with gaps) = 14 Alignment score =
0.809524
Cbrig.WS247_Fam_10_14_28                    TCCTTATTCCTTAT
Rev.of:Crema.WS247_Fam_4_14_69              TCCTTCTTCCTTCT
                                            ***** ****** *
Consensus:
                                            TCCTTaTTCCTTaT

>All.fam_Fam_141_12_2  Nr. of seq. 2 Alignment length(with gaps) = 12 Alignment score =
0.666667
Cbren.WS247_Fam_11_10_27                    CC-GGT-CTGGT
Rev.of:Cele.WS201_Fam_352_12_1              CCTGGTCCTGGT
                                            ** *** *****
Consensus:
                                            CCtGGTcCTGGT

>All.fam_Fam_150_10_2  Nr. of seq. 2 Alignment length(with gaps) = 10 Alignment score =
0.733333
Cbren.WS247_Fam_31_10_10                    GACCAGCCTA
Cbrig.WS247_Fam_115_10_2                    GACCATTCTA
                                            *****  ***
Consensus:
                                            GACCAgcCTA
```

Table S7.
Satellite families shared by *M. hapla* and *Caenorhabditis*

```
>Allfam_Fam_2_10_5  Nr. of seq. 5 Alignment length(with gaps) = 15 Alignment score = 0.480000
Cbren.WS247_Fam_190_10_2                    TTTTT-GAAA--T--
Cele.WS201_Fam_40_12_7                      TTTTT-GAAA--TTT
Rev.of:Crema.WS247_Fam_43_13_7              TTTTTTCAAA--TTT
Mhapla.WS247_Fam_303_10_1                   TTTTC-GGAA--T--
Crema.WS247_Fam_7_12_32                     TTTTC-GAAAGAT--
                                            ****    **  *
Consensus:
                                            TTTTtgaAAT

>Allfam_Fam_43_12_3  Nr. of seq. 3 Alignment length(with gaps) = 14 Alignment score = 0.674603
Cele.WS201_Fam_33_12_8                      TAAA-AATAAAAA-
Crema.WS247_Fam_149_12_2                    TAAC-AATAAAAA-
Mhapla.WS247_Fam_4_14_16                    TAAANAATTAAAAT
                                            ***  *** ****
Consensus:
                                            TAAaAATaAAAA

>Allfam_Fam_44_10_3  Nr. of seq. 3 Alignment length(with gaps) = 13 Alignment score = 0.632479
Cbren.WS247_Fam_61_10_6                     A-AAAAAATCG--
Mhapla.WS247_Fam_301_11_1                   ATAAAAAATCG--
Cbrig.WS247_Fam_107_12_2                    A-AAAAATTCGAC
                                            * ***** ***
Consensus:
                                            AAAAAAaTCG

>Allfam_Fam_78_23_2  Nr. of seq. 2 Alignment length(with gaps) = 23 Alignment score = 0.652174
Cbren.WS247_Fam_335_21_1                    CA-AATTTTTTTCGGGA-TTTTT
Rev.of:Mhapla.WS247_Fam_103_23_2            CAGAATTTTTTTCCCAAGTTTTT
                                            ** **********   * *****
Consensus:
                                            CAgAATTTTTTTCccaAgTTTTT

>Allfam_Fam_87_22_2  Nr. of seq. 2 Alignment length(with gaps) = 22 Alignment score = 0.666667
Cbren.WS247_Fam_106_20_3                    GGCAANTTGCCAGAGAAAAC--
Rev.of:Mhapla.WS247_Fam_263_22_1            GGCAATTTGCGACAAAAAACCT
                                            ***** **** * * *****
Consensus:
                                            GGCAAtTTGCcAcAaAAAACct

>Allfam_Fam_98_22_2  Nr. of seq. 2 Alignment length(with gaps) = 22 Alignment score = 0.643939
Crema.WS247_Fam_291_21_1                    AGGTGAAAAATTCAAATTTAT-
Rev.of:Mhapla.WS247_Fam_262_22_1            AAGTTTAAACTTTAAATTTATT
                                            * **  *** ** ********
Consensus:
                                            AaGTgaAAAaTTcAAATTTATt

>Allfam_Fam_99_21_2  Nr. of seq. 2 Alignment length(with gaps) = 21 Alignment score = 0.650794
Cbrig.WS247_Fam_16_19_14                    AAATTCTG--GTCCTTAAAAA
Mhapla.WS247_Fam_112_21_2                   AAATTCTGATGTTTTTAAACA
                                            ********  **  ***** *
Consensus:
                                            AAATTCTGatGTccTTAAAaA

>Allfam_Fam_108_21_2  Nr. of seq. 2 Alignment length(with gaps) = 21 Alignment score = 0.682540
Cele.WS201_Fam_132_21_2                     CAACTACCACAACTACCGTAC
Rev.of:Mhapla.WS247_Fam_44_21_3             CTTCTACCACAACAACAGCAC
                                            *  ********** ** * **
Consensus:
                                            CaaCTACCACAACaACaGcAC

>Allfam_Fam_110_20_2  Nr. of seq. 2 Alignment length(with gaps) = 20 Alignment score = 0.683333
Mhapla.WS247_Fam_50_18_3                    NTTTTTA-TA-GGCCTAGAA
Rev.of:Cele.WS201_Fam_312_20_1              ATGTTTACTACGGCCTAGAA
                                             * **** ** *********
Consensus:
                                            aTgTTTAcTAcGGCCTAGAA
```

```
>Allfam_Fam_113_20_2  Nr. of seq. 2 Alignment length(with gaps) = 20 Alignment score = 0.641667
Cele.WS201_Fam_24_19_12                    TTTATTTATTTAATT-AATA
Mhapla.WS247_Fam_30_19_4                   TTTATTTTTATAATTCAAG-
                                           ******* * ***** **
Consensus:
                                           TTTATTTaTaTAATTcAAga

>Allfam_Fam_114_20_2  Nr. of seq. 2 Alignment length(with gaps) = 20 Alignment score = 0.633333
Mhapla.WS247_Fam_276_19_1                  TTTTAT-TATTCGTGCGCTC
Crema.WS247_Fam_94_20_3                    TTTTCTGTATTCTTGTGCAC
                                           **** * ***** ** ** *
Consensus:
                                           TTTTaTgTATTCgTGcGCaC

>Allfam_Fam_122_19_2  Nr. of seq. 2 Alignment length(with gaps) = 19 Alignment score = 0.754386
Cbrig.WS247_Fam_244_18_1                   TTTTGAAAAAAAAAATT-T
Mhapla.WS247_Fam_114_19_2                  TTTTGAAGAAAAGAATTAT
                                           ******* **** **** *
Consensus:
                                           TTTTGAAaAAAAaAATTaT

>Allfam_Fam_133_18_2  Nr. of seq. 2 Alignment length(with gaps) = 18 Alignment score = 0.629630
Cele.WS201_Fam_323_18_1                    TTGTAGATCAACGGGGTC
Rev.of:Mhapla.WS247_Fam_52_18_3            TTGTGGTTCAATGGTGGC
                                           **** * **** ** * *
Consensus:
                                           TTGTaGaTCAAcGGgGgC

>Allfam_Fam_145_16_2  Nr. of seq. 2 Alignment length(with gaps) = 16 Alignment score = 0.708333
Mhapla.WS247_Fam_57_15_3                   ATAATTTTAAGCTT-T
Cbrig.WS247_Fam_250_16_1                   AAAATTTTAGGCTTGT
                                           * ******* **** *
Consensus:
                                           AaAATTTTAaGCTTgT

>Allfam_Fam_151_16_2  Nr. of seq. 2 Alignment length(with gaps) = 16 Alignment score = 0.666667
Cele.WS201_Fam_88_16_3                     AATTCCGGCAATCGGC
Rev.of:Mhapla.WS247_Fam_282_16_1           AATTTCGGGAAACGGG
                                           **** *** ** ***
Consensus:
                                           AATTcCGGcAAaCGGc

>Allfam_Fam_152_15_2  Nr. of seq. 2 Alignment length(with gaps) = 15 Alignment score = 0.655556
Mhapla.WS247_Fam_299_13_1                  AATTTTTG-GC-ATT
Cbrig.WS247_Fam_263_14_1                   AATTTTTGTGCTAT-
                                           ******** ** **
Consensus:
                                           AATTTTTGtGCtATt

>Allfam_Fam_154_15_2  Nr. of seq. 2 Alignment length(with gaps) = 15 Alignment score = 0.655556
Cbren.WS247_Fam_181_14_2                   TCAATAATCAATAA-
Rev.of:Mhapla.WS247_Fam_292_15_1           TCGATAATAAAAAAG
                                           ** ***** ** **
Consensus:
                                           TCaATAATaAAaAAg

>Allfam_Fam_157_14_2  Nr. of seq. 2 Alignment length(with gaps) = 14 Alignment score = 0.642857
Cele.WS201_Fam_28_12_9                     CTACCTACCTAT--
Rev.of:Mhapla.WS247_Fam_295_14_1           CTACCTATATATTA
                                           *******  ***
Consensus:
                                           CTACCTAcaTATta

>Allfam_Fam_160_14_2  Nr. of seq. 2 Alignment length(with gaps) = 14 Alignment score = 0.714286
Cele.WS201_Fam_89_14_3                     AATTTTAAATTGGA
Rev.of:Mhapla.WS247_Fam_294_14_1           AATTACAAATTGTA
                                           ****  ****** *
Consensus:
                                           AATTacAAATTGgA
```

```
>Allfam_Fam_162_13_2  Nr. of seq. 2 Alignment length(with gaps) = 13 Alignment score = 0.641026
Cele.WS201_Fam_353_12_1                    AGTTATAT-AGTA
Rev.of:Mhapla.WS247_Fam_298_13_1           AGTTATATGAGGG
                                           ******** **
Consensus:
                                           AGTTATATgAGga

>Allfam_Fam_165_12_2  Nr. of seq. 2 Alignment length(with gaps) = 12 Alignment score = 0.694444
Crema.WS247_Fam_35_10_9                    TTAATTTTTA--
Rev.of:Mhapla.WS247_Fam_10_12_8            ATAATTTTTAAA
                                            *********
Consensus:
                                           aTAATTTTTAaa

>Allfam_Fam_166_12_2  Nr. of seq. 2 Alignment length(with gaps) = 12 Alignment score = 0.722222
Mhapla.WS247_Fam_302_11_1                  CAGAATATA-AC
Rev.of:Cele.WS201_Fam_143_12_2             CAGAATATATTC
                                           *********  *
Consensus:
                                           CAGAATATAtaC

>Allfam_Fam_168_11_2  Nr. of seq. 2 Alignment length(with gaps) = 11 Alignment score = 0.696970
Crema.WS247_Fam_347_10_1                   TCGC-TATCTT
Mhapla.WS247_Fam_126_11_2                  TCGCATAACTT
                                           **** ** ***
Consensus:
                                           TCGCaTAaCTT

>Allfam_Fam_170_11_2  Nr. of seq. 2 Alignment length(with gaps) = 11 Alignment score = 0.651515
Mhapla.WS247_Fam_304_10_1                  GCAAATATTT-
Cele.WS201_Fam_18_11_14                    GCACATTTTTT
                                           *** ** ***
Consensus:
                                           GCAaATaTTTt
```

Table S8. The microsatellites in *Caenorhabditis*

Species	A	AT	AC	AG	W	AAT	AAC	ATC	ACT	AAG	AGG	ACC	AGC	ACG	CCG	CG	C
C.elegans	50	255*	125*	185*	12097*	31	4	13	5	24	10	4	6	11	0	0	27
C.briggsae	8	118*	115*	1457*	3389*	25	7	15	1	220*	4	1	1	0	0	0	2
C.remanei	22	146*	19	182*	4274*	32*	17	4	2	72*	5	0	2	5	0	0	6
C.brenneri	59	127*	49*	1848*	2875*	14*	14	20	2	101*	13	3	3	3	0	0	7
M.hapla	23	9	0	0	101176*	6	1	0	0	4	1	0	0	0	0	0	8

Number of repeats longer than 24 bases found for each motif. Values in both strands are added. The superscript (*) indicates that the maximum length is longer than 95 nucleotides.
